# Expression pattern analysis of transcribed HERV sequences is complicated by *ex vivo *recombination

**DOI:** 10.1186/1742-4690-4-39

**Published:** 2007-06-06

**Authors:** Aline Flockerzi, Jochen Maydt, Oliver Frank, Alessia Ruggieri, Esther Maldener, Wolfgang Seifarth, Patrik Medstrand, Thomas Lengauer, Andreas Meyerhans, Christine Leib-Mösch, Eckart Meese, Jens Mayer

**Affiliations:** 1 Department of Human Genetics, Medical Faculty, University of Saarland, Homburg, Germany; 2 Max Planck-Institute for Informatics, Saarbruecken, Germany; 3 Medical Faculty Mannheim of the Ruprecht-Karls, University of Heidelberg, Germany; 4 Department of Experimental Medical Sciences, Lund University, Lund, Sweden; 5 Institute of Virology, Medical Faculty, University of Saarland, Homburg, Germany; 6 GSF – National Research Center for Environment and Health, Institute of Molecular Virology, Neuherberg, Germany

## Abstract

**Background:**

The human genome comprises numerous human endogenous retroviruses (HERVs) that formed millions of years ago in ancestral species. A number of loci of the HERV-K(HML-2) family are evolutionarily much younger. A recent study suggested an infectious HERV-K(HML-2) variant in humans and other primates. Isolating such a variant from human individuals would be a significant finding for human biology.

**Results:**

When investigating expression patterns of specific HML-2 proviruses we encountered HERV-K(HML-2) cDNA sequences without proviral homologues in the human genome, named HERV-KX, that could very well support recently suggested infectious HML-2 variants. However, detailed sequence analysis, using the software RECCO, suggested that HERV-KX sequences were produced by recombination, possibly arising *ex vivo*, between transcripts from different HML-2 proviral loci.

**Conclusion:**

As RT-PCR probably will be instrumental for isolating an infectious HERV-K(HML-2) variant, generation of "new" HERV-K(HML-2) sequences by *ex vivo *recombination seems inevitable. Further complicated by an unknown amount of allelic sequence variation in HERV-K(HML-2) proviruses, newly identified HERV-K(HML-2) variants should be interpreted very cautiously.

## Background

About 8% of the human genome is composed of sequences with retroviral origin. Among those, human endogenous retroviruses (HERVs) are remnants of ancient exogenous retroviruses that infected germ line genomes followed by fixation of proviruses in the population millions of years ago. It appears that most HERV families formed in ancestral species during relatively short time periods, and creation of new proviruses then ceased. Therefore, most HERV families' proviruses accumulated numerous nonsense mutations that rendered them defective both regarding coding capacity for retroviral proteins and transcriptional activity [1-3]. The so-called HERV-K(HML-2) family, in short, HML-2, is exceptional in several aspects. HML-2 is transcribed in a variety of human tissues [4]. In germ cell tumors HML-2 expression is highly upregulated [5]. Several HML-2 proviruses in the human genome encode functional Gag, Pro, Pol and/or Env proteins, or even appear fully intact [6-8]. Furthermore, an additional protein from an HML-2 splice product, Rec, may be involved in germ cell tumorigenesis [9].

Several HML-2 loci are evolutionarily old. Those so-called HERV-K(OLD) loci typically harbor a 96 bp insertion within the *gag *gene [10]. In contrast, a number of HML-2 loci appear evolutionarily much younger. They are present in the human genome but not in the chimpanzee or other apes' genomes, so that they very likely formed after the evolutionary split of human from chimpanzee [6,8,11,12]. Such young HML-2 loci either exist as proviruses or as solitary LTRs, the latter being the result of homologous recombination between the LTR's of a provirus, leaving one LTR behind.

The source of evolutionarily young and reasonably intact HML-2 sequences is hitherto unknown. Based on HML-2 sequence analysis, Belshaw et al. suggested an infectious pool of endogenous HML-2 proviruses that has persisted within the primate lineage throughout the past 30 million years. HML-2 sequences from that infectious pool sporadically reinfected and formed new proviruses in the human germ line, where some proviruses became fixed in the population. However, an infectious HML-2 variant has not been isolated so far, and it is unclear whether infectious HML-2 variants exist in humans to this day [13,14]. If it still existed, the exact nature of that variant, in terms of its actual sequence, remains unspecified. We report here analysis of mysterious HML-2-like sequences, named HERV-KX, that we isolated in the course of an HML-2 expression study. Employing the recently developed software RECCO [15], we revealed that HERV-KX sequences very likely are *ex vivo *recombination products between transcripts from different HML-2 proviruses.

Generation of recombinants in the course of reverse transcription of RNA and subsequent PCR is a well known phenomenon. Retroviral reverse transcriptase (RT) is known to switch templates both *in vivo *and *ex vivo *[16-20]. While template switches of retroviral RT's are critical for retrovirus biology, RT template switches occuring *ex vivo *and thus producing sequence artifacts in molecular biology experiments have been described [21-24]. PCR is also prone to generate chimeric products [25,26].

Our results underline that the pursuit for a replicating HML-2 variant requires caution as to the interpretation of newly identified HML-2 sequences. Such caution is not only required when studying transcribed HML-2 sequences, but also when studying transcripts from other HERV families and other repetitive sequences in general. Furthermore, since detection of recombination events in candidate sequences can be computationally difficult, and since other software tools in our hands failed to produce similar results for our dataset, RECCO is a valuable software tool for examining such new sequences.

## Results

### Nature of HERV-KX sequences

**Figure 1:**
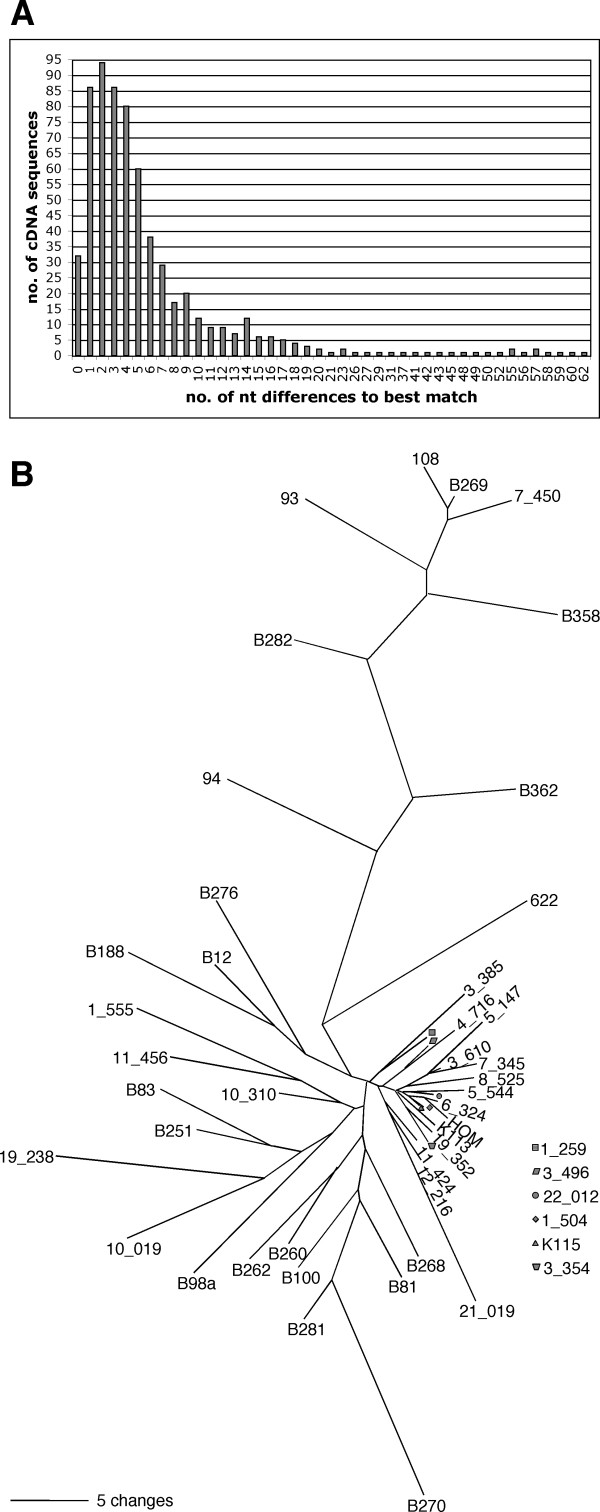
**Nature of HERV-KX sequences**. (A) Similarity of 642 HML-2 cDNA sequences and their best matching HML-2 loci in the human genome. The majority of cDNAs displayed between zero and a few differences to the best match, and were thus assignable to specific HML-2 loci. A minority of cDNAs displayed a greater number of dissimilarities to the best match and were thus not assignable with confidence to specific HML-2 loci. HERV-KX sequences were defined as displaying 18 or more nucleotide differences to the best matching HML-2 locus. (B) Sequence divergence of HERV-KX sequences in comparison to selected HML-2 reference sequences from the human genome, depicted as a neighbour joining-tree of the absolute number of nucleotide differences between sequences. For the sake of clarity, phylogenetically more distant HML-2 reference sequences were not included in the tree, as they were less similar to HERV-KX sequences than the reference sequences included in the tree. Proviral reference sequences are given as "xx_xxx" (see text). Positions with gaps were excluded in pairwise sequence comparisons.

In the course of an HERV-K(HML-2) expression study, we analysed, in total, 642 HML-2 cDNA sequences that were derived from various human tissues. About 95% of cDNA sequences could be unambiguously assigned to individual HML-2 proviral loci. Details for that part of the analysis will be reported elsewhere (Flockerzi et al., manuscript in preparation). In contrast, about 5% of cDNA sequences could not be definitely assigned to particular HML-2 loci in the human genome sequence. Their sequences were either similar to evolutionarily older HERV-K(OLD) proviruses, or to "modern" HERV-K(HML-2) proviruses, in that they displayed or lacked, respectively, a 96 bp sequence within the *gag *gene region [10]. We defined cDNA sequences as HERV-KX when they displayed in initial analysis 18 or more nt differences to the most similar HML-2 proviral locus. That number was based on observed nt differences of assignable cDNA sequences and should be taken as somewhat arbitrary due to the lack of defined criteria for HERV-KX sequences (see below). HERV-KX cDNA sequences displayed on average 37.5 (SD 16.2) nt differences from their most similar HML-2 locus. The most divergent HERV-KX sequence in our analysis displayed 62 (9%) nt differences to its best match. Compared with each other, HERV-KX sequences were heterogeneous in sequence as well (Figure 1). We did not identify an HERV-KX sequence twice. Thus, HERV-KX sequences comprised a diverse group of HML-2 (-like) sequences.

### RECCO analysis of test sequences

To characterize the nature of HERV-KX sequences, we investigated whether HERV-KX sequences showed signs of recombination events. Using the approach described in Materials and Methods, and in the Additional information [see Additional file 1], we first evaluated the power and sensitivity of the recombination detecting software RECCO [15] in our specific sequence context. For this purpose, we analysed HML-2 test sequences that were combined in silico from assignable cDNA sequences from different HML-2 proviruses, and thus generated one or two recombination events in a background of imperfect cDNA-provirus matches. For all those sequences RECCO was able to identify recombination events as well as regions where recombinations had "taken place" (Table 1). We then analysed assignable HML-2 cDNA sequences that very likely had not undergone recombination events. For all of those sequences, RECCO did not suggest recombination events (data not shown). Thus, RECCO detects recombination events in HML-2 sequences with high sensitivity, yet, does not falsely predict recombinations.

**Table 1:** Analysis of test sequences with Locus-Assigner and RECCO.

1	2	3	4	5	6	7	8	9
**artifical sequence**	**Locus-Assigner**	**# diff's**	**# recom's**	**loci involved**	**recom region**	**# diff's to locus**	**p value**	**# diff's after recom**

**B86_B107**	22_012	**37**	1	22_012 7_450	282 – 307	4 0	0,001	4
**B112_B131**	7_450	**34**	1	7_450 6_324/3_496	282 – 307	1 0	0,001	1
**B2_B15_B2**	4_742	**26**	2	4_742 10_019 4_742	317 – 384 583 – 603	0 0 0	0,003 0,001	0
**B443_B474**	10_019	**18**	2	10_019 3_385	388 – 436	3 2	0,001	**5**
**B86_B44**	22_012	**8**	1	10_310 1_504	59 – 191 503 – 639	0 4		**4**
**B86_B44**	22_012	**8**	0	22_012		8		**8**
**B44_B45_B187**	11_424/3_610/5 544/7_345	**6**	0	11_424/3_610/5_544/7_345		6		**6**

Test sequences were created in silico by combining sequence regions of assignable HML-2 cDNA sequences, with the aim to construct recombination events between transcripts from corresponding HML-2 loci. Pairwise comparisons, using the in-house Bio-Python script Locus-Assigner, were then performed for test and reference sequences. Column 1 denotes the cDNA sequence name. Column 2: best matching HML-2 proviral locus according to pairwise sequence comparisons with Locus-Assigner. The numbers given here disregard the PCR primer binding regions, and therefore are, for HERV-KX sequences B262, B12 and 93, below the <18 nt differences-threshold initially set for HERV-KX (see text). Due to the lack of a HERV taxonomy, and for the sake of simplicity, HML-2 proviral loci have been numbered artificially in this paper, with the first number indicating chromosomal location of the locus, and the second number being a consecutive number without further connotation. Column 3: nucleotide differences to best match. Column 4: number of recombinations according to RECCO. Column 5: proviral loci involved in recombination(s). More than one locus is given when different loci displayed identical (dis)similarities. Column 6: regions where recombination(s) occured, with the 5' end of the RT-PCR product being defined as nucleotide +1. Column 7: nucleotide differences to individual HML-2 loci. Column 8: p-values of particular predicted recombinations. Column 9: nucleotide differences remaining after considering recombination(s) (see text).

### Comparison of RECCO with other recombination detection software

We also tested other recombination detection software, e.g. RDP2 [27] and TOPALi [28]. We analyzed the artificial recombinant sequences "B86_B107" and "B112_B131" that showed particularly strong recombination signals in RECCO analysis (Table 1). TOPALi apparently did not correctly score gaps; it detected the 96 bp insertion between nucleotide 68 and 163 as a recombination for both sequences. Interestingly, TOPALi identified the true recombination breakpoint when we removed most sequences from the alignment that are not involved in the recombination and collapsed gap regions as much as possible. RDP2 could roughly identify the breakpoint location in the full alignment, but did not assign parental sequences correctly (data not shown). In summary, the size of the dataset and the number of gap regions made the analysis very difficult for current recombination detection programs.

### RECCO analysis of HERV-KX sequences

**Figure 2:**
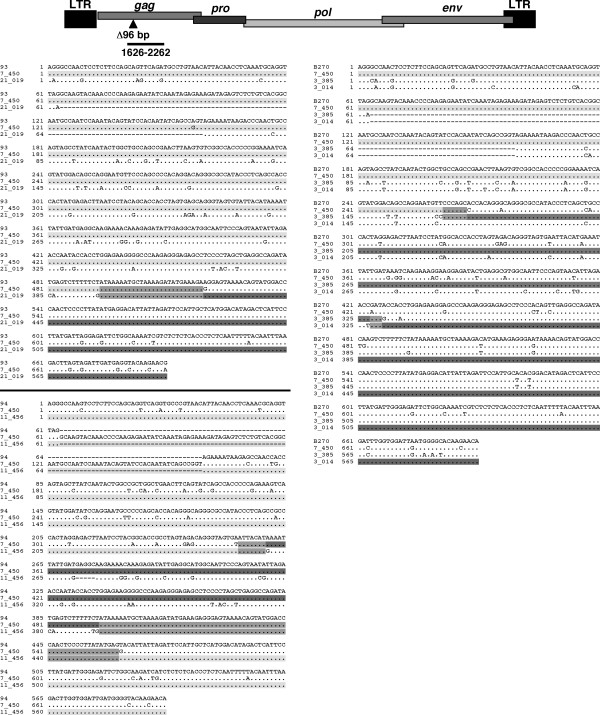
**RECCO-based explanation of HERV-KX sequences as recombination products**. Based on RECCO analysis, HERV-KX sequences can be explained as recombination products between transcripts from different HML-2 proviral loci. Three examples of HERV-KX sequences were multiply aligned with HML-2 proviral sequences, the transcripts of which served as template for recombination events. HERV-KX sequence "93" was generated by one recombination event involving two loci. HERV-KX sequence "B270" was generated by two recombination events involving three loci. HERV-KX sequence "94" was generated by two recombination events involving two different proviral templates. Probable recombination regions are indicated by medium grey background. Light and dark grey background indicates proviral templates. Localization of the RT-PCR amplicon and the 96 bp sequence (see text) within the HML-2 gag gene/provirus is depicted above the sequence comparisons.

**Figure 3:**
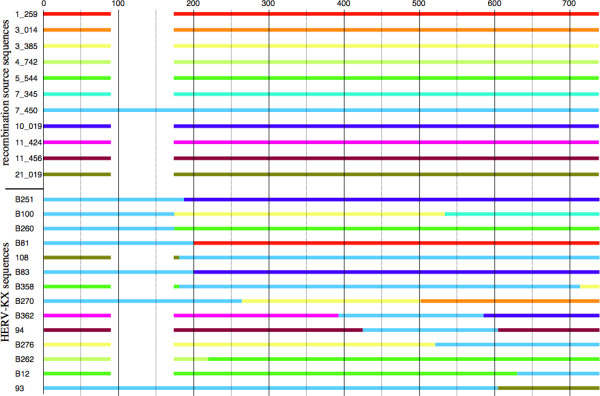
**Graphical representation of HERV-KX sequences as recombination products**. Recombination source sequences and HERV-X sequences are depicted graphically in the upper and lower part, respectively. Colors depict composition of HERV-KX sequences from the various source sequences. Thus, color changes indicate regions of predicted recombination events (see Table 2). Lack of a 96 bp sequence (see text) is indicated by a gap.

Using RECCO, we analysed HERV-KX sequences in comparison to respective HML-2 proviral sequences in the human genome. All HERV-KX sequences were predicted by RECCO to consist of two to three different HML-2 proviral sequences, thus having undergone one or two, respectively, recombination events. For two sequences, alternative predictions, further reducing the number of nucleotide differences, involved four different proviruses, thus involving three recombination events (Table 2). Recombinations within three exemplary cDNA sequences can be portrayed as follows. HERV-KX sequence "B270", displaying 39 nt differences to its best match, was predicted to be comprised of sequences from a provirus on chromosome 7 and two different proviruses on chromosome 3. Recombinations were predicted to have occured between nt 267 and 273 and between nt 583 to 712 of the cDNA, resulting in an explanation with zero nt differences. HERV-KX sequence "94", displaying 26 nt differences to its best matching provirus on human chromosome 11, was predicted to be comprised of sequences from HML-2 proviruses on chromosomes 7 and 11. Recombination events were predicted to have occured between nt 415 and 422, and between nt 583 and 651, resulting in an explanation with zero nt differences. Finally, HERV-KX sequence "93", displaying 13 nt differences to its best match, was predicted to be comprised of proviruses on human chromosome 7 and 21, with a recombination between nt 583–610, and resulting in an explanation with 1 nt difference remaining (Figures 2 and 3). For all HERV- KX sequences, on average 35 (SD 15.5) nt differences could be reduced to 2.6 (SD 3.4) nt differences when considering RECCO predictions for recombinations. Those numbers are in ranges typically observed in our analysis for best matches of assignable sequences, where, on average, 4.5 (SD 3.7) nt differences remained between cDNA and best match. Of further note, because we defined HERV-KX sequences as displaying 18 or more nt differences to the best match, it is possible that there are further recombined cDNAs with corresponding signals among the sequences with <18 nt differences.

**Table 2:** Analysis of HERV-KX sequences with Locus Assigner and RECCO.

1	2	3	4	5	6	7	8	9
**HERV-KX sequence**	**Locus- Assigner**	**# diff's**	**# recom's**	**loci involved**	**recom region**	**# diff's to locus**	**p value**	**# diff's after recom**

B251 (*)	7_450	**56**	3	3_610/5_147/7_345	59–67	0	0,059	
				7_450	182–191	0	0,001	
				10_019 1_504/K115/19_352	385–436	3 1	0,023	**4**
B251 (*)	7_450	**56**	2	7_450	182–191	6	0,001	
				10_019	385–436	3	0,023	
				1_504/K1_15/19_352		1		**10**
B251 (*)	7_450	**56**	1	7_450	182–191	6	0,001	
				10_019		14		**20**
**B100**	7_450	**54**	2	7_450	164–181	0	0,001	
				3_385 7_345	510–551	0 0	0,004	**0**
**B260**	7_450	**53**	1	7_450	164–181	0	0,001	
				5_544		4		**4**
**B81**	7_450	**52**	1	7_450	192–205	1	0,001	
				1_259		6		**7**
108 (**)	3_610	**51**	1	21_019	68–181	0	0,001	
				7_450		0		**0**
B83 (*)	7_450	**51**	2	7_450	192–209	3	0,001	
				10_019	437–466	2	0,201	
				1_504/19_352/K115		2		**7**
B83 (*)	7_450	**51**	1	7_450	192–209	3	0,001	
				10_019		13		**16**
B358 (**)	11_424/3_354/1_504/5_544/K115	**50**	2	5_544	68–181	2	0,001	
				7_450	727–752	0	0,004	
				3_385		0		**2**
B269 (**)	3_610	**48**	1	3_610/7_345/5_147	68–181	0	0,001	
				7_450		0		**0**
**B281**	7_450	**43**	1	7_450	238–242	0	0,001	
				1_504/1_259		4		**4**
B268 (*)	7_450	**41**	2	7_450	164–181	0	0,001	
				1_504/22_012/6_324	583–712	0	0,122	
				7_450		0		**0**
B268 (*)	7_450	**41**	1	7_450 1_504/22_012/6_324	164–181	0 6	0,001	
**B270**	7_450	**39**	2	7_450	267–273	0	0,001	
				3_385	502–505	0	0,001	
				3_014		0		**0**
**B282**	1_504	**36**	2	3_354/1_504	238–242	1	0,001	
				7_450	583–712	0	0,004	
				3_385		0		**1**
B362 (*)	1_259	**31**	2	11_424	389–401	2	0,001	
				7_450	583–624	0	0,102	
				10_019		0		**2**
B362 (*)	1_259	**31**	1	11_424	389–401	2	0,001	
				7_450		9		**11**
**94**	11_456	**26**	2	11_456	415–422	0	0,001	
				7_450	583–651	0	0,003	
				11_456		0		**0**
**B98a**	3_496/7_345	**20**	1	10_019	214–263	2	0,001	
				7_345/1_504		8		**10**
622 (*)	3_014	**19**	2	HOM/6_324	316–388	0	0,001	
				3_014	676–764	0	0,330	
				3_385		0		**0**
622 (*)	3_014	**19**	1	HOM/6_324	316–388	0	0,001	
				3_014		5		**5**
B276 (*)	1_504	**18**	2	3_354/1_504	192–306	0	0,021	
				3_385	521–553	0	0,001	
				7_450		0		**0**
B276 (*)	1_504	**18**	1	3_385	521–553	5	0,001	
				7_450		0		**5**
**B188**	11_424	**18**	3	1_259/11_424	256–265	0	0,006	
				7_450	404–414	0	0,006	
				(***)	583–610	1	0,002	
				7_450		0		**1**
**B262**	3_496	**17**	1	4_742	209–226	0	0,001	
				5_544		4		**4**
**B12**	5_544	**14**	1	5 544	583–673	7	0,001	
				7_450		0		**7**
**93**	7_450	**13**	1	7_450	583–610	1	0,001	
				21 019		0		**1**

Designation of columns is the same as in Table 1.

(*) RECCO provided alternative explanations with different p-values. The lowest p-value did not neccessarily correspond to the explanation with the least nt differences. By visual inspection of sequences, another explanation may be favored despite a higher p-value.

(**) HERV-KX sequences without 96 bp sequence. RECCO predicted recombination with loci having the 96 bp sequence (see text), and predicted a recombination involving the 96 bp sequence. Regions of recombination events may therefore be shorter then the ones given by RECCO.

(***) Among 10 different proviral loci, RECCO does not specify a particular locus because all 10 loci are very similar or identical in sequence to each other in the concerned region. By visual inspection, a more optimal explanation among the 10 loci may be specified.

Taken together, RECCO analysis indicates that HERV-KX sequences are due to recombination events and that HERV-KX sequences are not represented in the human genome assembly because of that.

## Discussion

The nature of HML-2 sequences having formed evolutionarily young HML-2 proviruses in the human genome remains speculative. Based on HML-2 sequence analysis, Belshaw et al. put forward a replication-competent, infectious variant that occasionally (re-)infects humans and thus forms new proviruses, some of which became fixed in the population [13]. However, the sequence of such a variant and its current host(s) remains unknown. As reported just recently, engineered HML-2 consensus sequences, named Phoenix and HERV-K_CON, are infectious and form new proviruses. However, as long as a naturally occuring infectious variant has not been identified, it is not clear how similar in sequence Phoenix or HERV-K_CON would be to that variant [29,30]. Eventually, it will be essential to demonstrate by molecular genetic means the naturally occurring variant.

In the course of an investigation of expression patterns of HERV-K(HML-2) proviruses in various human tissues, we encountered among all sequenced cDNA's about 5% of so-called HERV-KX cDNA sequences displaying high similarities to HML-2, yet, without definite proviral homologues in the human genome. In comparison to each other, HERV-KX sequences were heterogeneous.

In principle, HERV-KX sequences might corroborate the hypothesis of a replication-competent HML-2 variant. HERV-KX sequences were not isolated from every individual and might represent rare allelic variants or newly formed proviruses in the investigated tissue specimen. And, HERV-KX sequence heterogeneity might imply a quasispecies-like nature of infectious HML-2 variants. Some HML-2 loci indeed exist as allelic variants, consisting of tandem proviruses or full-length versus solitary LTRs, or being present at, or absent from a genomic locus [31-34]. However, it is very unlikely that as many HML-2 proviruses as represented by HERV-KX sequences went unnoticed so far. Alternatively, HERV-KX sequences could also stem from a new, recombined, transcriptionally active provirus that had formed after reverse transcription (including template switches *in vivo*) of HML-2 RNAs within an HML-2-encoded retroviral particle. If so, one would expect to repeatedly isolate a new, defined cDNA sequence from that provirus, just as we repeatedly isolated transcripts from known HML-2 proviruses. However, HERV-KX sequences were heterogeneous among each other and we did not identify a new cDNA population from a new HML-2 provirus (see Fig. 1A). Also, we isolated HERV-KX sequences from RNA from brain, that does not express detectable amounts of HML-2 Gag protein, and thus retroviral particles (M. Sauter, unpublished results).

Of greater significance, closer inspection of HERV-KX sequences with the recombination-detecting software RECCO strongly indicates that HERV-KX sequences are mosaics between different HML-2 proviruses and therefore can be interpreted as recombinants between different transcribed proviruses and with varying recombination breakpoints--thus explaining HERV-KX sequence heterogeneity. Even though it is possible to argue for a few HERV-KX sequences about weak recombination signals that only save a few differences between cDNA and proviral sequences, nearly all analyzed HERV-KX sequences show a clear signal for at least a single recombination event.

Our study does not formally exclude recombinations between HML-2 sequences occuring *in vivo*. However, because of above described findings, we conclude that isolated HERV-KX sequences are very unlikely to stem from allelic polymorphisms of HML-2 loci, or from new, recombined proviruses that formed in the investigated tissue sample. Recombinations are very likely due to *ex vivo *recombination.

There are two opportunities for *ex vivo *recombination in the course of the experiment. First, during cDNA generation, reverse transcriptase could have switched from an RNA template from one HML-2 provirus to an RNA template from another HML-2 provirus, forming a chimera between two different HML-2 proviruses. Second, during PCR, incompletely polymerised DNA single-strands could have reannealed with an incomplete DNA single-strand from another HML-2 provirus and were then polymerised until the end, likewise forming a chimera. Both mechanisms of *ex vivo *recombination have been studied in detail recently, and are known to produce chimeric sequences [16-21,25,26]. HERV-KX sequences could have been produced either by one, or by a combination of both mechanisms. In this context, we were able to produce 10.5% of recombinant *gag *sequences in a separate *ex vivo *experiment by *in vitro *transcribing different HML-2 *gag *sequences, followed by RT-PCR on mixed RNA, cloning of RT-PCR products, and screening for recombinants utilizing restriction enzymes sites present in one or the other *gag *sequence [see Additional file 1].

Our findings are significant for the identification of an infectious HML-2 variant. Generation of *ex vivo *recombination products in analogous experimental studies seems almost inevitable.

Results from such studies should therefore be interpreted very cautiously concerning isolation of new HML-2 (-like) sequences. That is, it will be difficult to nambiguously demonstrate an infectious HML-2 variant just by RT-PCR. In addition, after taking recombination into account, remaining nucleotide differences between experimental and reference sequences, as observed for a few HERV-KX sequences, are no strong arguments for having isolated an infectious HML-2 variant. First, RT, PCR and sequencing errors must be considered. Second, recent work from our group demonstrated for the HERV-K(HML-2.HOM) provirus several haplotypes on the single nucleotide level that severely affected coding capacity of proviral *gag*, *pro *and *pol *genes [33]. Similar findings were reported for HML-2 *env *sequences [35]. It seems reasonable to assume that other HML-2 proviruses likewise comprise numerous alleles on the DNA sequence level, thus contributing to the observed remaining nucleotide differences.

Taken together, since there is strong evidence that HERV-KX sequences are due to *ex vivo *recombination events, it seems unlikely that HERV-KX sequences are indeed portions of a replication-competent, infectious HML-2 variant.

In the light of computational difficulties of detecting recombination events in biological sequences, our study furthermore demonstrates that RECCO is a valuable software tool to detect or to potentially exclude such *ex vivo *recombination events. This is not only true for studies on the HML-2 family but also for other HERV families, ERVs in other species and other classes of repetitive elements.

## Conclusion

RT and PCR experiments will probably be instrumental to examine presence of a recently proposed replicating and infectious HERV-K(HML-2) variant in human individuals. Both RT and PCR generate *ex vivo *recombination products that may not be recognized as such, thus pretending new HML-2 variants that falsely corroborate replicating and infectious HML-2 variants. Using the software RECCO, recombination products may be discerned from true sequences with less difficulty. As for HML-2 sequences without recombination signals, allelic variation of HML-2 loci on the nucleotide level should be taken into account before treating them as instances of replicating and infectious HML-2 variants. Besides, our findings may be taken as a reminder of potential pitfalls when investigating repetitive sequences.

## Methods

### Generation of HML-2 cDNA sequences

Generation and analysis of HML-2 cDNA sequences is described in more detail elsewhere (Flockerzi et al., manuscript in preparation). In brief, cDNA sequences were amplified by RT-PCR from total RNA isolated from human tissue specimens. PCR primers encompassed a *gag *gene region of 637 bp, with respect to the previously published HERV-K(HML-2.HOM) sequence [10]. That *gag *gene region also includes a 96 bp sequence that is missing in evolutionarily young HML-2 loci but is present in evolutionarily older, so-called HERV-K(OLD) loci [10]. RT-PCR products were cloned into pGEM T-Easy (Promega), and inserts from a number of positive clones were sequenced using vector-specific primers. Poor quality sequence reads were not further analysed. Using the in-house Bio-Python script Locus-Assigner, that basically performs pairwise sequence comparisons and catalogues numbers of differences between cDNA and reference sequences (described in more detail in Flockerzi et al., manuscript in preparation), we assigned cDNA sequences to their corresponding HML-2 provirus utilizing nucleotide differences between the various HML-2 proviruses. HERV-KX candidate sequences, that could not be assigned to individual HML-2 loci, were further substantiated by sequencing both strands and generating a consensus sequence for each.

HERV-KX sequences reported in this study were isolated from RNA from different human specimens, such as human brain and germ cell tumor tissue.

### Characterization of HERV-KX sequences and analysis with RECCO

We analysed HERV-KX sequences for various aspects, such as sequence divergence from known HML-2 sequences and between each other, length of open reading frames, nucleotide substitution patterns, and potential recombination events. Eventually, only results from the latter analysis proved to be meaningful, and are presented here. We employed the software RECCO [15], version 0.92, to examine each HERV-KX sequence for potential recombination events. As reference sequences for recombination analysis we used corresponding *gag *gene regions from the HML-2 proviruses published in the human genome reference sequence. A previously described allelic HML-2 provirus, HERV-K113 [32], that is not present in the human genome reference sequence, was also included. A manually optimized multiple alignment of those HML-2 sequences served as reference sequence dataset for RECCO. To derive p-values for predicted recombinations we used the DNA mutation cost setting of RECCO and performed 1000 permutations.

Test sequences for RECCO analysis were generated by combining in silico sequence portions from assignable cDNAs from different HML-2 proviruses, thus resembling HERV-KX-like recombination products.

## Competing interests

The author(s) declare that they have no competing interests.

## Authors' contributions

AF, OF, AR and EMa carried out the molecular genetic studies. AF and JMaydt performed sequence analysis and wrote and adopted software. PM participated in the computer analysis. WS, TL, AM, CLM, EMe, and JMayer conceived of the study, participated in its design and financed it. JMayer drafted the manuscript. All authors read and approved the final manuscript.

## Supplementary Material

Additional file 1 Additional information for HERV-KX sequence analysis. Additional information on analysis of HERV-KX sequences, including. (i) recombination analysis of HERV-KX sequences with RECCO, (ii) description of treatment of gaps in RECCO analysis, (iii) original RECCO output for HERV-KX sequences "B270", "93" and "94", (iv) multiple alignment of HERV-KX and HML-2 reference sequences, (v) reproduction of HERV-K(HML-2) *gag *sequence recombinants Click here for file
