# Supplementary material for: Expression pattern analysis of transcribed HERV sequences is complicated by ex vivo recombination
Source: Retrovirology. 2007 Jun 6;4:39. doi: 10.1186/1742-4690-4-39 (PMC1904241; doi:10.1186/1742-4690-4-39)
Supplement: Additional file 1 — Additional information for HERV-KX sequence analysis. Additional information on analysis of HERV-KX sequences, including. (i) recombination analysis of HERV-KX sequences with RECCO, (ii) description of treatment of gaps in RECCO analysis, (iii) original RECCO output for HERV-KX sequences "B270", "93" and "94", (iv) multiple alignment of HERV-KX and HML-2 reference sequences, (v) reproduction of HERV-K(HML-2) gag sequence recombinants [file 1742-4690-4-39-S1.pdf]

Supplementary information

Expression pattern analysis of transcribed HERV sequences is complicated by *ex vivo* recombination

Flockerzi et al.

Recombination analysis of HERV-KX sequences with RECCO

RECCO [1] is a novel method for analyzing sequences subject to recombination. Given costs for recombination and for mutation events, RECCO finds all cost-optimal explanations of the putative recombinant sequence in terms of recombination and mutation of a given set of aligned sequences. A high recombination cost results in an explanation that only uses the single, most related reference sequence without any recombination events. As the recombination cost decreases, more and more recombination events are introduced in the explanation in favor of mutation events. The first recombination event reduces the number of mutations needed for an explanation by the largest factor. RECCO builds a list of recombination events and displays the amount of mutation cost saved by each recombination – the so called "savings" of a recombination. RECCO also computes the total mutation cost of the explanation that includes this recombination event (see Table 2). True recombinant sequences usually display a strong reduction in mutation cost (i.e. a high savings value) for the first few recombination events introduced.

To quantify the statistical significance of each recombination event, RECCO generates sets of alignments by permuting the columns of the alignment. As a result, the permuted alignments do not contain any recombination signal, but have the same diversity as the original alignment. P-values are then estimated by computing the probability of obtaining higher savings than observed in the given alignment purely by chance, based on the analysis of the set of permuted alignments. We report the p-values for the query sequence here, as our goal was to analyze the recombination signal for the query sequence only.

Treatment of gaps in RECCO analysis

Treating gaps correctly was critical for the analysis of the HERV-KX sequences, as the multiple alignment contained two long gaps and several small gaps. Recently published recombination detection methods usually implement one of the three following options: (i) discard sites that contain a gap character, (ii) treat each gap character as a fifth nucleotide state or (iii) treat each consecutive run of columns containing gaps as a large polymorphism (Geneconv [2]). The first option results in an unacceptable loss of information, in our case. For example, the 96 bp indel differentiates between evolutionarily young and old HERV-KX sequences [3]. The second option may lead to an artificially high similarity or dissimilarity between sequences in gap regions and eventually produces spurious recombination events. The third option prohibits recombinations in any run of columns containing a gap, such that a sequence containing a long gap may confound recombinations that involve other sequences. It is also difficult to choose an adequate scoring term for the resulting large polymorphisms. In conclusion, all existing approaches for treating gaps either discard a lot of information and thus miss recombination events or may infer spurious recombination events solely based on gap information.

We decided to implement an approach that discriminates between possibly spurious recombination events based on gap information and recombination events based on polymorphisms. First, it is important to realize that gaps in the query sequence have a different quality than gaps in the sequences used for an explanation. If gaps in the query sequence are matched with nucleotides in the explanation, the involved sequence in the explanation represents irrelevant information. Hence, gaps in the query sequence are assigned zero cost, such that all columns with a gap in the putative recombinant are effectively removed from the alignment. The situation is totally different if nucleotides in the query sequence are matched with a gap in the explanation. In this case, there is missing information as the query sequence is not fully explained by the other sequences. Consequently, we have chosen to penalize gaps in the explanation.

To capture the effect of the gap penalty on inferred recombination events, we executed RECCO [1] for all gap penalties in the set {0.016, 0.02, 0.04, 0.07, 0.1, 0.2, 0.4, 0.6, 0.8}. As gaps were strongly preferred for gap penalties below 0.016 and resulted in spurious recombination events, we did not consider smaller gap penalties than 0.016. The results were then preprocessed as follows: recombination events with a p-value above 0.05 were discarded as not significant. If a recombination event with the same start and end position was detected as significant for all gap penalties, it was accepted as correct. Other recombination events were

manually examined and consolidated. In the tables displaying recombination events, we only report the sequence p-value for the default gap penalty of 0.2.

References

1. Maydt J, Lengauer T: **Recco: recombination analysis using cost optimization.** *Bioinformatics* 2006, **22:**1064-1071.
2. Sawyer S: **Statistical tests for detecting gene conversion.** *Mol Biol Evol* 1989, **6:**526-538.
3. Mayer J, Meese E, Mueller-Lantzsch N: **Human endogenous retrovirus K homologous sequences and their coding capacity in Old World primates.** *J Virol* 1998, **72:**1870-1875.

Following pages:

Original RECCO output for HERV-KX sequences "B270", "93" and "94"

Multiple alignment of HERV-KX and HML-2 reference sequences, provided in fasta format.

Reproduction of HERV-K(HML-2) *gag* sequence recombinants

```
           1   5    10   15   20   25   30   35   40   45   50   55   60   65   70   75   80   85   90   95   100  105  110  115
10_019     AGGACC---AAGTCCTCTTCCAGTAGGTCAGATGCCCGTAACATTACAACCTCAAACGCAGGTTAG-------------------------------------------------
10_310     AGGGCC---AAGTCCTCTTCCAGCAGGTCAGGTGCCCGTAACACTACAACCTCAAACGCAGGTTAA-------------------------------------------------
11_233     -------------------------------------------------------------------------------------------------------------------
11_277     ATGGCC---AACTCCTCTTCCAGCGGTTCAGATGCCTGTAACATTACAACCTCAAATGCAGGTTAGACAAGTACAAACCCCAAGAGAATATCAAATAGAGAG--------TCTCT
11_424     AGGCAC---AAGTCCTCTTCCAGCAGGTCAGGTGCCCGTAAGATTACAACCTCAAACGCAGGTTAA-------------------------------------------------
11_456     AGGGCC---AAGTCCTCTTCCAGCAGGTCAGGTGCCCGTAACATTACAACCTCAAACGCAGGTTAG-------------------------------------------------
12_216     AGGCAC---AAGTCCTCTTCCAGCAGGTCAGGTGCCCGTAACATTACAACCTCAAACGCAGGTTAA-------------------------------------------------
12_419     -------------------------------------------------------------------------------------------------------------------
14_023     ATGGCCAACAACTCCTTCTCCCACGGTTCAGATGCCTGTGACATTACAACCTCAAA-----------------------------------------------------------
16_010     ATGGC---CAACTCCTCCTCCCGTGGTTCAGATACCTGTAACATTACAACCTCAAATGCAGGTTAGACAAGTACAAACCCCAAGAGAATATCAAATAGAAAAGGATAG--TCTCT
17_029     -------------------------------------------------------------------------------------------------------------------
19_157     ATGGCC---AACTCCTCTTCCAGCAGTTCAGATGCCTGTAACATTACAACCTCAAATGCAGGTTAGACAAGTACAAACCTGAAGAGAATAACAAATAGAGAAAGATAGAGTATCT
19_238     AGGGCC---ATGTCCTCTTCCAGCAGGTCAGATGCCCGTAATATTACAACCTCAAACGCAGTTTAG-------------------------------------------------
19_352     AGGCAC---AAGTCGTCTTCCAGCAGGTCAGGTGCCCGTAACATTACAACCTCAAACGCAGGTTAA-------------------------------------------------
19_407     ATGGCC---AACTCCTCTCCCAGCAGTTCAGATGCCTGTAACATTACAACCTCAAATGCAGGTTAGACAAGTACAAACCCCAAAAGAATATCAAATAGGGAAAGATAGAGTCTCT
19_639     -------------------------------------------------------------------------------------------------------------------
1_059      ATGGCCATCAACTCCTCCTCCAGTGGTTCAGATGCCTGTAACATTACAACCTCAAATGCAGGTTAGACAAGTGCAAACCCCAAGAGAATATCAAGTAGAAAAGGATAGAGTCTCT
1_061      ATGGCCATCAACTCCTCCTCCAGCGGTTCAGATGCCTGTAACATTACAACCTCAAATGCAGGTTAGACAAGTGCAAACCCCAAGAGAATATCATGTAGAAAAGGATAGAGTCTCT
1_259      AGGCAC---AAGTCCTCTTCCAGCAGGTCAGGTGCCCGTAAGATTACAACCTCAAACGCAGGTTAA-------------------------------------------------
1_504      AGGCAC---AAGTCATCTTCCAGCAGGTCAGGTGCCCGTAACATTACAACCTCAAAAGCAGGTTAA-------------------------------------------------
1_555      AGGGCC---AAGTCCTCTTTCAGCAGGTCAGGTGACCGTAACATTACAACCTCAAGCGCAGGTTAG-------------------------------------------------
20_078     ATGGCC---AACTCCTCCTCCCACAGTTCAGATGCCCGTAACATTACAACCTCAAATGCAGGTTAGACAAGTACAAACCCCAAGAGAATATCAGATAGAAAAGGATAG--TCTCT
21_019     AAGGCC---AAGTCCTCTTCCAGCAAGTCAGGTGCCTGTAACATTACAACCTCAAACGCAGGTTAA-------------------------------------------------
22_012     AGGGAC---AAGTCCTCTTCCAGCAGGTCAGGTGCCCGTAACATTACAACCTCAAAAGCAGGTTAA-------------------------------------------------
22_041     ATCGCCATCAACTCCTCCTCCCGCGGTTCAGATGCCTGTAACATTACAACCTCAAACGCAGGTTAGACAAGCACAAACCCCAAGAGAAAATCAAGTAGAAAGGGACAGAGTCTCT
2_Tan      AGGCAC---AAGTCCTCTTCCAGCAGGTCAGGTGCCTGTAACATTACAACCTCAAAAGCAGGTTAA-------------------------------------------------
3_014      AGGGAC---AAGTCCTCTTCCAGCAGGTCAGATGCCCGTAACATTACAACCTCAAACACAGGTTAG-------------------------------------------------
3_209      ATGGCCACCATCTCCTTCTCCCGCGGTTCAGATACCTGTGACATCACAATCTCAAA-----------------------------------------------------------
3_294      AGGGCC---AAGTCCTCTTCCAGCAGGTCAGGTGCCCGTAACATTACAACCTCAAACGCAGGTTAA-------------------------------------------------
3_354      AGGCAC---AAGTCATCTTCCAGCAGGTCAGGTGCCCGTAACATTACAACCTCAAAAGCAGGTTAA-------------------------------------------------
3_385      AGGCAC---AAGTCCTCTTCCAGCAGGTCAGGTGCCTGTAACATTACAACCTCAAATGCAGGTTAA-------------------------------------------------
3_496      AGGGCC---AAGTCCTCTTCCAGCAGGTCAGGTGCCCGTAACATTACAACCTCAAAAGCAGATTAA-------------------------------------------------
3_610      AGGCAC---AAGTCCTCTTCCAGCAGGTCAGGTGCCTGTAACATTACAACCTCAAACGCAGGTTAA-------------------------------------------------
4_008      -------------------------------------------------------------------------------------------------------------------
4_032      ATGGCCACCAACCCCTTCTCCCGCGGTTCAGATGCCTGTGATGTCACAATCTCAAA-----------------------------------------------------------
4_052      ATGGCCACCAACTCCTTCTCCCGCGGTTCAGATGCCTTGATGTCACAATCTCAAA-----------------------------------------------------------
4_068      ATGGCCACCAACCCCTTCTCCCGCGGTTCAGATGCCTGTGATGTCACAATCTCAAA-----------------------------------------------------------
4_716      AGGGCC---AAGTCCTCTTCCAGCAGGTCAGGTGCCCGTAACATTACAACCTCAAAAGCAGGTTAA-------------------------------------------------
4_742      AGAGCC---AAGTCCTTTTCTAGCAGGTCAGATGCCCATAACATTACAACCTCAAACGCAGGTTAG-------------------------------------------------
5_147      AGGCAC---AAGTCCTCTTCCAGCAGGTCAGGTGCCTGTAACATTACAACCTCAAACGCAGGTTAA-------------------------------------------------
5_259      AGCACC---AAGCCCTCTTCCAGCAGGTGAGATGCCTGGAACATTACAGTCTCCAATGCAGGTTAG-------------------------------------------------
5_535      ACGGCCATCAACTCCTCCTCCTGCGGTTCAGATGCCTGTGACATTACAACCTCAAATGCAGGTTAGACAAGTACAAACTGCAAGAGAATATCAAGTAGAAAAGGATAGAGTCTCT
5_544      AGGCAC---AAGTCCTCTTCCAGCAGGTCAGGTGCCCGTAAGATTACAACCTCAAAAGCAGGTTAA-------------------------------------------------
6_098      ATGGCC---AACTCCTCTTCCAGCAGTTCAGATGCCTGTAACATTGCAACCTCAAATGCAGGTTAGACAAGTACAAACCCCAGAAGAATATCAAATAGAAAAAGATAAAGTCTCT
6_178      ATGGCCATCAA---CTCCTCCCGTGGTTCAGATACCTGTAACATTACAACCTCAAAGGGAGGTTAGACAAGTACAAACCCCAAGAGAATATCAAGTAGAAAAGGATAGAGTCTCT
6_240      -------------------------------------------------------------------------------------------------------------------
6_324      AGGCAC---AAGTCCTCTTCCAGCAGGTCAGGTGCCTGTAACATTACAACCTCAAAAGCAGGTTAA-------------------------------------------------
7_345      AGGCAC---AAGTCCTCTTCCAGCAGGTCAGGTGCCTGTAACATTACAACCTCAAACGCAGGTTAA-------------------------------------------------
7_450      AGGGCC---AACTCCTCTTCCAGCAGTTCAGATGCCTGTAACATTACAACCTCAAATGCAGGTTAGGCAAGTACAAACCCCAAGAGAATATCAAATAGAGAAAGATAGAGTCTCT
8_047      ATGGCCACCAACTCCTTCTCCCGCGGTTCAGATGCCTGTGATGTCACAATCTCAAA-----------------------------------------------------------
8_074      ATGGCCACCAACTCCTTCTCCCGCGGTTCAGATGCCTGTGATGTCACAATCTCAAA-----------------------------------------------------------
8_094      ATGGCCACCAACTCCTTCTCCCGCGGTTCAGATGCCTGTGATGTCACAATCTCAAA-----------------------------------------------------------
8_121      -------------------------------------------------------------------------------------------------------------------
8_235      -------------------------------------------------------------------------------------------------------------------
8_525      AGGCAC---AAGTCCTCTTCCAGCAGGTCAGGTGCCCGTAACATTACAACCTCAAAAGCAGGTTAA-------------------------------------------------
8_559      ATGGCCACCAACTCCTTCTCCTGTGGTTCAGATGCCTGTGACATTACAACCTCAAA-----------------------------------------------------------
9_307      -------------------------------------------------------------------------------------------------------------------
9_340      ATGGCCATCAACTCCTCCTCCCGCGGTTCAGATGCCTGTAACATTACAACCTCAAATGCAGGTTAGACAAGTACAAACCCCAAGAGAATATCAAATAGAAAAGGAAAGAGTCTCT
HML2_Rep   AGGCAC---AAGTCCTCTTCCAGCAGGTCAGGTGCCTGTAACATTACAACCTCAAAAGCAGGTTAA-------------------------------------------------
K113       AGGCAC---AAGTCCTCTTCCAGCAGGTCAGGTGCCCGTAACATTACAACCTCAAAAGCAGGTTAA-------------------------------------------------
K115       AGGCAC---AAGTCCTCTTCCAGCAGGTCAGGTGCCCGTAACATTACAACCTCAAAAGCAGGTTAA-------------------------------------------------
X_540      -------------------------------------------------------------------------------------------------------------------
Y_026      ATGGCCACCAACTCCTTCTCCCGCGGTTCAGATGCCTGTGACATTACAACCTCAAA-----------------------------------------------------------
B270       AGGGCC---AACTCCTCTTCCAGCAGTTCAGATGCCTGTAACATTACAACCTCAAATGCAGGTTAGGCAAGTACAAACCCCAAGAGAATATCAAATAGAGAAAGATAGAGTCTCT
```

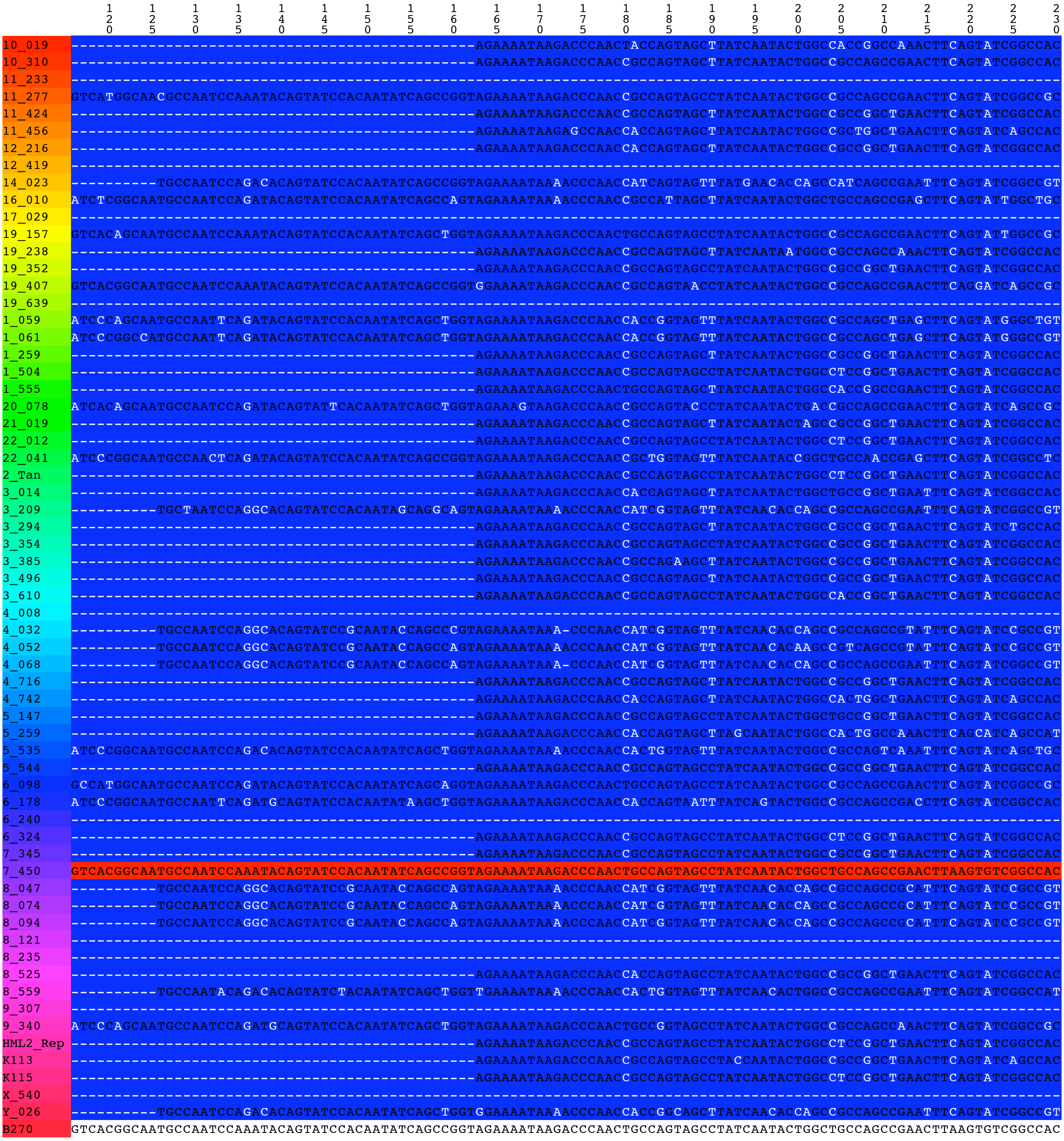
10_019
10_310
11_233
11_277
11_424
11_456
12_216
12_419
14_023
16_010
17_029
19_157
19_238
19_352
19_407
19_639
1_059
1_061
1_259
1_504
1_555
20_078
21_019
22_012
22_041
2_Tan
3_014
3_209
3_294
3_354
3_385
3_496
3_610
4_008
4_032
4_052
4_068
4_716
4_742
5_147
5_259
5_535
5_544
6_098
6_178
6_240
6_324
7_345
7_450
8_047
8_074
8_094
8_121
8_235
8_525
8_559
9_307
9_340
HML2_Rep
K113
K115
X_540
Y_026
B270

```
Position    235  240  245  250  255  260  265  270  275  280  285  290  295  300  305  310  315  320  325  330  335  340  345
10_019     CCCC-AGAAAGTCAGTATGGATATCCAGGAACGCTCCCAGCACCACAGGGCAG----GGCGCCATACCCTCAGCCACCCACTAGCAGAC--------------------------
10_310     CCCC-AGAAAGTCAGTATGGATATCCAGGAATGCCCCCAGCACCACAGGGCAG----GGCGCCATACCCTCAGCCGCCCACTAGGAGAC--------------------------
11_233     -------------------------------------------------------------------------------------------------------------------
11_277     CCCC-AGAAAATCAGTATGGACAGCCAGGAGTGTTTCCAGCACCACAGGGCAG----GGCGCTATAGCCTCAGCCGCCCACTATAAGAC--------------------------
11_424     CCCC-AGAAAGTCAGTATGGATATCCAGGAATGCCCCCAGCACCACAGGGCAG----GGCGCCATACCCTCAGCCACCCACTAGGAGAC--------------------------
11_456     CCCC-AGAAAGTCAGTATGGATATCCAGGAATGCCCCCAGCACCACAGGGCAG----GGCGCCATACCCTCAGCCGCCCACTAGGAGAC--------------------------
12_216     CCCC-AGAAAGTCAGTATGGATATCCAGGAATGCCCCCAGCACCACAGGGCAG----GGCGCCATACCCTCAGCCGCCCACTAGGAGAC--------------------------
12_419     -------------------------------------------------------------------------------------------------------------------
14_023     CTCC-AGTGGTTCAGTATAGATCCCAGGTGTTGTGTCCTGTGCCAAATAGCAG----GGCAGTATAGCAACAACCCATGGCGATGGTGT--------------------------
16_010     CTCC-AGAGGTCCAATACAGACCTCAAGTGGTGTGTGCCATGCCAAATTGCAC----GGCATCGTACAAGCAACCCATGGCGGTGGTGTTTAATACGTCAGCACCACAGGGCGCG
17_029     -------------------------------------------------------------------------------------------------------------------
19_157     CCCT-AGAAAATCAGCATGGACAGCCAGGAATGTTTCCAGCACCACAGGGCAG----GGCACTATATCCGCAGCCTCCCACTATGAGAC--------------------------
19_238     CCCC-AGAAAGTCAGTATGGATATCCAGGAACGCTCCCAGCACCACAGGGCAG----GGCGCCATACCCTCAGCCACCCACTAGGAGAC--------------------------
19_352     CCCT-AGAAAGTCAGTATGGATATCCAGGAATGCCCCCAGCACCACAGGGCAG----GGCGCCATACCCTCAGCCGCCCACTAGGAGAC--------------------------
19_407     CCCC-AGAAAATCAGTATGGACAGCCAGGAATGTTTCCAGCACCACAGGGCAG----GGCGCTATATCCTCAGCAGCCCACTATGAGAT--------------------------
19_639     -------------------------------------------------------------------------------------------------------------------
1_059      CTCC-GGAGGTTCAATACAGACCTCAAGCTGTGTGTCCCGTGCCACATAGCAC----GGCACCTCACCAGCAACCCA-----GTGGTGT--------------------------
1_061      CTCC-GGAGGTTCAATACAGACCTCAAGCTGTGTGTCCCGTGCCACACAGCAC----GGCACCTCACCAGCAACCCA-----GCGGTGT--------------------------
1_259      CCCC-AGAAAGTCAGTATGGATATCCAGGAATGCCCCCAGCACCACAGGGCAG----GGCGCCATACCCTCAGCTGCCCACTAGGAGAC--------------------------
1_504      CCCC-AGAAAGTCAGTATGGATATCCAGGAATGCCCCCAGCACCACAGGGCAG----GGCGCCATACCCTCAGCCGCCCACTAGGAGAC--------------------------
1_555      CCCC-AGAAAGTCAGTATGGATATCTAGGAATGCCCCCAGCACCACAGGGCAG----GGAGCCATACCCTCAGCCGCCCACTAGGAGAC--------------------------
20_078     CCCCCAGAAAATCAGTATGGACAGCCAGGAACGTTTCCAGCACCACAGGGCACGCACGGCGATATAGCCTCAGCTGCCCACTATGAGAC--------------------------
21_019     CCCC-AGAAAGTCAGTATGGATATCCAAGAATGCCCCCAGCACCACAGGGCAG----GGCGCCATACCCTCAGCCGCCCACTAGGAGAC--------------------------
22_012     CCCC-AGAAAGTCAGTATGGATATCCAGGAATGCCCCCAGCACCACAGGGCAG----GGCGCCATACCCTCAGCCGCCCACTAGGAGAC--------------------------
22_041     CTTC-AGAGGTTCAATACAGACCTCAAGCGGTGTGTCCTGTGCCAAATAGCAC----GGCACCATACCAGCAACCCACAGCGATGGCGTCTAATTCACCAGCAACACAGGACGCG
2_Tan      CCCC-AGAAAGTCAGTATGGATATCCAGGAATGCCCCCAGCACCACAGGGCAG----GGCGCCATACCCTCAGCCGCCCACTAGGAGAC--------------------------
3_014      CCCC-AGAAAGTCAGTATGGACATCCAGGAATGCTCCCAGCACCACAGGGCAG----GGCGCCATACCCTCAGCCGCCCACTATGAGAC--------------------------
3_209      CTCC-AGAGGTTCAGTATGGATCTCAGGTGGTGCGTCCTGTGCTAAATAGCAA----GACACTATATCAACAACCCACGGCGATGGCGT--------------------------
3_294      CCCC-AGAAAGTCAGTATGGATATCCAGGAATGCCCCCAGCACTACAGGGCAG----GGCGCCATATCCTCAGCCGCCCACTGTGAGAC--------------------------
3_354      CCCC-AGAAAGTCAGTATGGATATCCAGGAATGCCCCCAGCACCACAGGGCAG----GGCGCCATACCCTCAGCCACCCACTAGGAGAC--------------------------
3_385      CCCC-AGAAAGTCAGTATGGATATCCAGGAATGCCCCCAGCACCACAGGGCAG----GGCGCCATACCCTCAGCTGCCCACTAGGAGAC--------------------------
3_496      CCCC-AGAAAGTCAGTATGGATATCCAGGAATGCCCCCAGCACCACAGGGCAG----GGCGCCATACCCTCAGCCGCCCACTAGGAGAC--------------------------
3_610      CCCC-AGAAAGTCAGTATGGATATCCAGGAATGCCCCCAGCACCACAGGGCAG----GGCGCCATACCCTCAGCCGCCCACTAGGAGAC--------------------------
4_008      -------------------------------------------------------------------CAGCAACCCACGGCGGTGGCGC--------------------------
4_032      CTCC-AGAGGTTCAGTATGGATCTCAGGCGGTGCGTCCTGTGCCAAATAGCAA----GGCACTATATCAACAACCCACGGCGATGGCGT--------------------------
4_052      CTCC-AGAGGTTCAGTATGGATCTCAGGCGGTGCGTCCTGTGCCAAATAGCAA----GGCACTATATCAACAACCCAAGGCGATGGCGT--------------------------
4_068      CTCC-AGAGGTTCAGTATGGATCTCAGGAGGTGCGTCCTGTGCCAAATAGCAA----GGCACTATATCAACAACCCACGGCGATGGCGT--------------------------
4_716      CCCT-AGAAAGTCAGTGTGGATATCCAGGAATGCCCCCAGCACCACAGGGCAG----GGCGCCATACCCTCAGCCGCCCACTAGGAGAC--------------------------
4_742      CCCC-AGAAAGTCAGTATGGACATCCAGGAATGCTGCCAGCACTACAGAATAG----GGTGCCACACCCTCAGCCGCCCACTTTGAGAC--------------------------
5_147      CCCC-AGAAAGTCAGTATGGATATCCAGGAATACCCCCAGCACCACAGGGCAG----GGTGCCATACCCTCAGCCGCCCACTAGGAGAC--------------------------
5_259      CCCC-AGAAAGTCAGTATGGACATTCAGGAATGCTCCCAGCACCACAGGGCAG----GGAGCCATACCCTCAGCCACCCACTATGACAC--------------------------
5_535      TTCC-AGAGGTTCAGTATAGATCTCAGGCGGGGTGTCCTGTCCCAAATAGCAG----GGCACTATATCAGCAACCCACAGCGATGGCGT--------------------------
5_544      CCCC-AGAAAGTCAGTATGGATATCCAGGAATGCCCCCAGCACCACAGGGCAG----GGCGCCATACCATCAGCCGCCCACTAGGAGAC--------------------------
6_098      CCCC-AGAAAATCAGTATGGACACCCAGGAATGTTTCCAGCACCACAGGGCAG----GGCTCTAGATCCTCAGCCGTCCACTGTGAGAC--------------------------
6_178      CTCC-AGAGGTTAAATACAGACCTCAAGTGGTGTGTCCTGTGCCAAATAGCAC----GGCACCATACCAGCAACCCACGGCAGTGGTGT--------------------------
6_240      -------------------------------------------------------------------------------------------------------------------
6_324      CCCC-AGAAAGTCAGTATGGATATCCAGGAATGCCCCCAGCACCACAGGGCAG----GGCGCCATACCCTCAGCCGCCCACTAGGAGAC--------------------------
7_345      CCCC-AGAAAGTTAGTATGGATATCCAGGAATGCCCCCAGCACCACAGGGCAG----GGCGCCATACCCTCAGCCGCCCACTAGGAGAC--------------------------
7_450      CCCC-GGAAAATCAGTATGGACAGCCAGGAATGTTCCCAGCCCCACAGGACAG----GGCGCCATACCCTCAGCCACCCACTATGAGAC--------------------------
8_047      CTCC-AGAGGTTCAGTATGGATCTCAGGCGGTGCGTCCTGTGCCAAATAGCAA----GGCAGTATATCAACAACCCACGGCGATGGCGT--------------------------
8_074      CTCC-AGAGGTTCAGTATGGATCTCAGGCGGTGCGTCCTGTGCCAAATAGCAA----GGCACTATATCAA---CCCACGGCGATGGCGT--------------------------
8_094      CTCC-AGAGGTTCAGTATGGATCTCAGGCGGTGCGTCCTGTGGCAAATAGCAA----GGCACTATATCAACAACCCACGGCGATGGCGT--------------------------
8_121      -------------------------------------------------------------------------------------------------------------------
8_235      -------------------------------------------------------------------------------------------------------------------
8_525      CCCC-AGAAAGTCAGTATGGATATCCAGGAATGCCCCCAGCACCACAGGGCAG----GGCGCCATACCCTCAGCCGCCCACTAGGAGAC--------------------------
8_559      CTCC-AGAGGTGCAGTATAGATCCCAGATGGGA--TCCTGTGCCAAATAGTAG----GGCACTATATCAACAACCCACGGCGATGGTGT--------------------------
9_307      -------------------------------------------------------------------------------------------------------------------
9_340      CCCC-AGAGTATCAGTATGGATGGCCAGGA-TGTTTCCAGCACCGCAGGGCAG----GGCGCTATAGCCGCAGCCGCCCACTATGAGAC--------------------------
HML2_Rep   CCCC-AGAAAGTCAGTATGGATATCCAGGAATGCCCCCAGCACCACAGGGCAG----GGCGCCATACCCTCAGCCGCCCACTAGGAGAC--------------------------
K113       CCCC-AGAAAGTCAGTATGGATATCCAGGAATGCCCCCAGCACCACAGGGCAG----GGCGCCATACCCTCAGCCGCCCACTAGGAGAC--------------------------
K115       CCCC-AGAAAGTCAGTATGGATATCCAGGAATGCCCCCAGCACCACAGGGCAG----GGAGCCATACCCTCAGCCGCCCACTAGGAGAC--------------------------
X_540      -------------------------------------------------------------------------------------------------------------------
Y_026      CTCC-AGAGGTTCAGTATAGAACTCAGGTGGTGCGCCCAAC-CCAAATAGCAG----GGCACTATATCAACATCCCACGGCAATGGTGT--------------------------
B270       CCCC-GGAAAATCAGTATGGACAGCCAGGAATGTTCCCAGCACCACAGGGCAG----GGCGCCATACCCTCAGCTGCCCACTAGGAGAC--------------------------
```

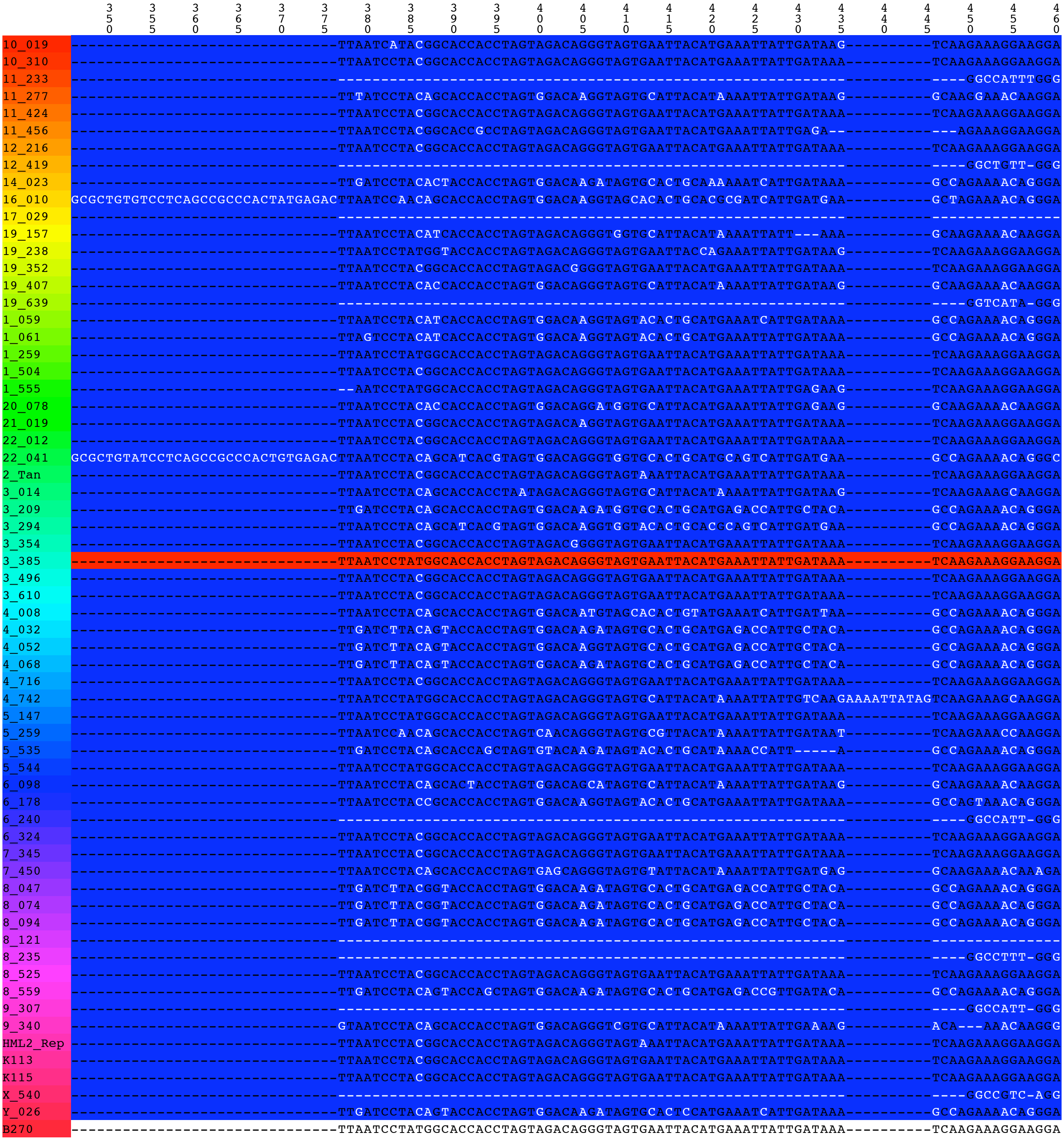

10_019
10_310
11_233
11_277
11_424
11_456
12_216
12_419
14_023
16_010
17_029
19_157
19_238
19_352
19_407
19_639
1_059
1_061
1_259
1_504
1_555
20_078
21_019
22_012
22_041
2_Tan
3_014
3_209
3_294
3_354
3_385
3_496
3_610
4_008
4_032
4_052
4_068
4_716
4_742
5_147
5_259
5_535
5_544
6_098
6_178
6_240
6_324
7_345
7_450
8_047
8_074
8_094
8_121
8_235
8_525
8_559
9_307
9_340
HML2_Rep
K113
K115
X_540
Y_026
B270

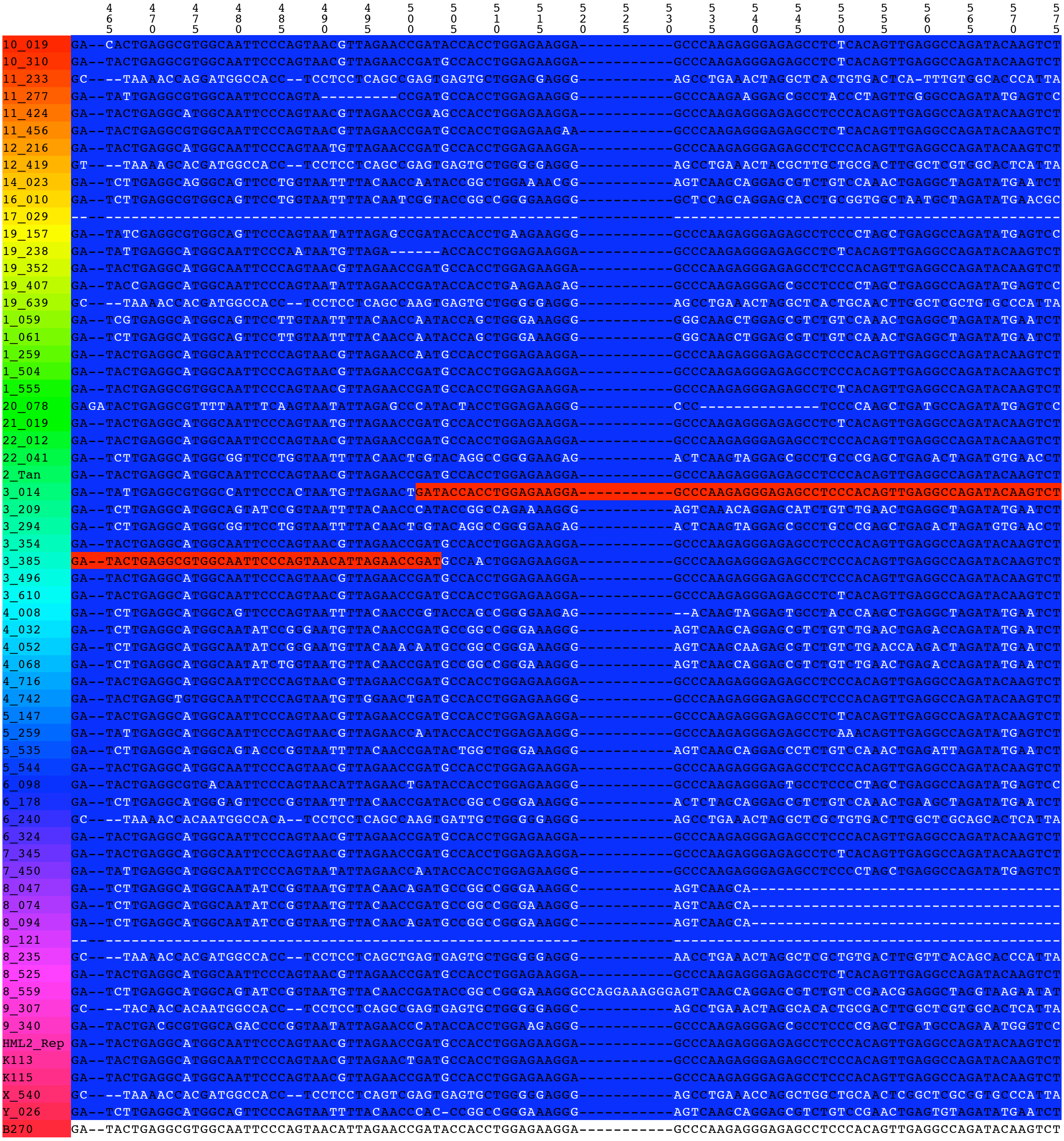
10_019
10_310
11_233
11_277
11_424
11_456
12_216
12_419
14_023
16_010
17_029
19_157
19_238
19_352
19_407
19_639
1_059
1_061
1_259
1_504
1_555
20_078
21_019
22_012
22_041
2_Tan
3_014
3_209
3_294
3_354
3_385
3_496
3_610
4_008
4_032
4_052
4_068
4_716
4_742
5_147
5_259
5_535
5_544
6_098
6_178
6_240
6_324
7_345
7_450
8_047
8_074
8_094
8_121
8_235
8_525
8_559
9_307
9_340
HML2_Rep
K113
K115
X_540
Y_026
B270

```
          580  585  590  595  600  605  610  615  620  625  630  635  640  645  650  655  660  665  670  675  680  685  690
10_019    TTTTCGATAAAAATGCT-AAAAGATATGAAAGAGGGAGTAA--AACACTATGGACCCAACTCCCCTTATATGAGGACATTATTAGATTCCATTGCTCATGGACATCATAGACTCA
10_310    TTTTCGATAAAAATGCT-AAAAGATATGAAAGAGGGAGTAA--AACAGTATGGACCCAAATCCCCTTATATGAGGACATTATTAGATTCCATTGCTCATGGACAT---AGACTCA
11_233    TTGCCCAACCTGCAGTT-----CACTATGGTGAAGGAGCAA--TTCAGACTCACCCTGCAGCATCCGTCTGGGTCAAACAGTGG---CTGCTCCCTAAGGAATAGTTGGGGGCG
11_277    TTTTCTATAAAAATGTT-AAAAGATATGAAAGAGGGAGTAA--AACAGTATGGACCCAACTCCCCTTATATGAGAACATTATTAGGTTCCACTGCTCATGGACAT---AGACTCA
11_424    TTTTCGATAAAAATGCT-AAAAGATATGAAAGAAGGAGTAA--AACAGTATGGACCCAACTCCCCTTATATGAGGACATTATTAGATTCCATTGCTCATGGACAT---AGACTCA
11_456    TTTTTGATAAAAATGCT-AAAAGATATGAAAGAGGGAGTAA--AACAGTATGGACCCAACTCCCCTTATATGAGTACATTATTAGATTCCATTGCTCATGGACAT---AGACTCA
12_216    TTTTCAATAAAAATGCT-AAAAGATATGAAAGAAGGAGTAA--AACAGTATGGACCCAACTCCCCTTATATGAGGACATTATTAGATTCCATTGCTCATGGACAT---AGACTCA
12_419    TTGCCCAACCTGCAGTT-----CACTACGGTGAAGGAGCAA--TTCAGACTTGCCCTGCAGCTTCCTGTATGGGTCAAAGAGTGG---CCGCTCTCTAAGGAAAAGTTGGGGGTG
14_023    TTCACCATGAAAATGTT-AAAAGATATGAAGGAGGCAGTTA--AACAATATGGACCCAACTCTCCTTATATGAGAACATTATTAGATTCCATTGCTAATGGGAAT---AGACTTA
16_010    TTCACCATGAAAATGTT-AAAAGACATGAAGGAAGGAGTTA--AACAATATGGACCCAACTCACCTTCTATGAGAACATTATTAGATTCCATTGCTCCAGCAGG-----------
17_029    -------------------------------------------GTCAGTATGGTCCCAATTCGCCTTTCATCCGATCGCTATTGCAATCTGTGGCTCAGAATAAG---CTATTGA
19_157    TTTTCTATAAAAATGCT-AAAAGATGTGAAAGAGGGAGTAA--AA-AGTATGGACTCAACTCCCCTTATTTGAGGACATTATTAATTCCATTGCTCATGGACAT---AGACTCA
19_238    TTTTCGATAAAAATGCT-AAAAGATATGAAAGAGGGAGTAA--AACAGTATGGACCCAACTCTCCTTATATGAGGACATTATTAGATTCCATTGCTCATGGACAT---AGACTCA
19_352    TTTTCGATAAAAATGCT-AAAAGATATGAAAGAGGGAGTAA--AACAGTATGGACCCAACTCCCCTTATATGAGGACATTATTAGATTCCATTGCTCATGGACAT---AGACTCA
19_407    TTTTCCATAAAAATGCT-AAAAGATATGAAAGAGGGAGTAA--AACAGTATGGACCCAACTCCCCTTATATGAGGACATTATTAGATTCCATTGCTCATGGACAT---AGACTCA
19_639    TTGCCCAACTTACAGTT-----CATTATGGTGAAGGAGCAA--TTCAGACTTGCCTTGCAGCATCCTGTCTGAGTCAAACAGTGG---CTGCTCCCTAAGGAAAATTTGGGGGCG
1_059     TTCACCATGAAAATGTT-AAAAGATATGAAGGAAGGAGTTA--AACAATATGGACCCAACTCCCCTTATATGAGAACTTTATTAGATTCCATTGCTCATGGAAAT---AGACTTA
1_061     TTCACCATGAAAATGTT-AAAAGATATGAAGGAAGGAGTTA--AACAATATGGACCCAACTCCCCTTATATGAGAACTTTATTAGATCCCATTGCTCATGGAAAT---AGACTTA
1_259     TTTTCGATAAAAATGCT-AAAAGATATGAAAGAGGGAGTAA--AACAGTATGGACCCAACTCTCCTTATATGAGGACATTATTAGATTCCATTGCTCATGGACAT---AGACTCA
1_504     TTTTCGATAAAAATGCT-AAAAGATATGAAAGAGGGAGTAA--AACAGTATGGACCCAACTCCCCTTATATGAGGACATTATTAGATTCCATTGCTCATGGACAT---AGACTCA
1_555     TTTTAGATAAAAATGCT-AAAAGATATGAAAGAGGGAGTAA--AACAGTATGGACCCAACTCCCCTTATATGAGGACATTATTAGATTCCATTGCTCATGGACAT---AGACTCA
20_078    TTTTCTATAAAAATGCT-AAAAGACATGAAAGAGGGAGTAA--AACAGTATGGACCCAATTCTCCTTATATGAGAACATTATTAGATTCCATTGCTCATGGAAAT---AGACTTA
21_019    TTTTCGATAAAAATGCT-AAAAGATATGAAAGAAGGAGTAA--AACAGTATGGACCCAACTCCCCTTATATGAGGACATTATTAGATTCCATTGCTCATGGACAT---AGACTCA
22_012    TTTTCGATAAAAATGCT-AAAAGATATGAAAGAGGGAGTAA--AACAGTATGGACCCAACTCCCCTTATATGAGGACATTATTAGATTCCATTGCTTATGGACAT---AGACTCA
22_041    TTCACCATGAAAATGTT-AAAAGATATAAAGGAAGGAGTTA--AACAATATGGATCCAACTCCCCTTATATAAGAACATTATTAGATTCCATTGCTCATGGAAAT---AGACTTA
2_Tan     TTTTCGATAAAAAAGCT-AAAAGATATGAAAGAGGGAGTAA--AACAGTATGGACCCAACTCCCCTTATATGAGGACATTATTAGATTCCATTGCTCATGGACAT---AGACTCA
3_014     TTTTCTATAAAAATGCT-AAAAGACATGAAAGAGGGAATAA--AACAGTATGGACCCAACTCCCCTTATATGAGGACATTATTAGATTCCATTGCACACGGACAT---AGACTCA
3_209     TTCACCATGAAAATGTT-AAAAAATTTAAAGGAAGGATTTA--AACAATATGGACCCAACTGTCCTTATATGAGAACATTATTAGATTCCATTGCTCATGGAAAT---AGACTTA
3_294     TTCACCATGAAAATGTT-AAAAGATATAAAGGAAGGAGTTA--AACAATATGGATCCAACTCCCCTTATATAAGAACATTATTAGATTCCATTGCTCATGGAAAT---AGACTTA
3_354     TTTTCGATAAAAATGCT-AAAAGATATGAAAGAGGGAGTAA--AACAGTATGGACCCAACTCCCCTTATATGAGGACATTATTAGATTCCATTGCTCATGGACAT---AAACTCA
3_385     TTTTCGATAAAAATGCT-AAAAGATATGAAAGAGGGAGTAA--AACAGTATGGACCCAACTCCCCTTATATGAGGACATTATTAGATTCCATTGCTCATGGACAT---AGACTCA
3_496     TTTTCGATAAAAATACT-AAAAGATATGAAAGAGGGAGTAA--AACAGTATGGACCCAACTCCCCTTATATGAGGACATTATTAGATTCCATTGCTCATGGACAT---AGACTCA
3_610     TTTTCGATAAAAATGCT-AAAAGATATGAAAGAGGGAGTAA--AACAGTATGGACCCAACTCCCCTTATATGAGGACATTATTAGATTCCATTGCTCATGGACAT---AGACTCA
4_008     TTCACCTTGAAAATGTT-AAAAGATATGAAGAAAGGAGTTATAAACAATATGGACCCAACTCCCCTTACATGAGAACATTATTAGATTCCATTGTTCATGGAAAT---AGACTTA
4_032     TTCACCACAAAAATGTT-AAAAGATATGAAGGAAGGAGTTA--AACAATATGGACCTAACTCTCCTTATATGAGAACATTATTAGATTCCATTGCTCATGGAAAT---AGACTTA
4_052     ATCACCATAAAAAAATTTAAAAGATATGAAGGAAGGAGTTA--AACAATATGGACCTAACTCTCCTTATATGAGAACACTATTAGATTCCATTGCTCATGGAAAT---AGACTTA
4_068     TTCACCACAAAAATGTT-AAAAGATATGAAGGAAGGAGTTA--AACAATATGGACCTAACTCTCCTTATATGAGAACATTATTAGATTCCATTGCTCATGGAAAT---AGACTTA
4_716     TTTTCGATAAAAATGCT-AAAAGATATGAAAGAGGGAGTAA--AACAGTATGGACCCAACTCCCCTTATATGAGGACATTATTAGATTCCATTGCTCATAGACAT---AGACTCA
4_742     TTTTCTATAAAAATGCT-AAAAGATAGGAAAGACGGAGTCA--AACAGTATGGACCCAACTCCCCTTACATGAGGACATTATTAGATTCCATTGCTCATGGACAT---AGACTCA
5_147     TTTTCGATAAAAATGCT-AAAAGATATGAAAGAGGGAGTAA--AACAGTATGGACCCAACTCCCCTTATATGAGGACATTATTAGATTCCATTGCTCATGGACAT---AGACTCA
5_259     TTTTCTATAAAAATGCT-GAAAGATATGAAAGAGGGAGTAA--AACAGTATGGACCCAACTTCCCTTATATGAGGACATTATTAGACTCCATTGCTCATGGTCAT---AGACTCA
5_535     TTCACCCTGAAAATGTT-AAAAGATATGAAGGAAGGAGTTA--AACAATATGGACCCAACTCTCCTTATATGAGAACATTATTAGATTCCATTGCTCATGGAAAT---AGACTTA
5_544     TTTTCGATAAAAATGCT-AAAAGATATGAAAGAGGGAGTAA--AACAGTATGGACCCAACTCCCCTTATATGAGGACATTATTAGATTCCATTGCTTATGGACAT---AGACTCA
6_098     TTTTCTATAAAAATGCT-AAAAGATATGAAAGAGGGAGTAA--AACAGTATGGACCCAGCTCCCCTTATATGAAATATTATTAGATTCCATTGCTCATGGACAG---AGACTCA
6_178     TTCACCATGAAAATGTT-AAAAGATATGAAGGAAGGAGTT------AATATGGACCCAACTCCCCTTATGTGAGAACATTATTAGATTCCATTGTTCATGGAAAT---AGACTTA
6_240     TTGCCCAACCTACAGCT-----CACTACAATGAAGGAGCAA--TTCAGACTCACCCTGCAGTGTCCTGTATGGGTCAAACAGTGG---CCGCTCTCTAAGGAAAAGTTGGGTGTG
6_324     TTTTCGATAAAAATACT-AAAAGATATGAAAGAGGGAGTAA--AACAGTATGGACCCAACTCCCCTTATATGAGGACATTATTAGATTCCATTGCTCATGGACAT---AGACTCA
7_345     TTTTCGATAAAAATGCT-AAAAGATATGAAAGAGAGAGTAA--AACAGTATGGACCCAACTCCCCTTATATGAGGACATTATTAGATTCCATTGCTCATGGACAT---AGACTCA
7_450     TTTTCTATAAAAATGCT-AAAAGATATGAAAGAGGGAGTAA--AACAGTATGGACCCAACTCCCCTTATATGAGGACATTATTAGATTCCATTGCTCATGGACAT---AGACTCA
8_047     ----------------------------------GGAGTTA--AACAATATGGACCTAACTCTCCTTATATGAGAACATTATTAATTCCATTGCTCATGGAAAT---AGACTTA
8_074     ----------------------------------GGAGTTA--AACAATATGGACCTAACTCTCCTTATACGAGAATATTATTAATTCCATTGCTCATGGAAAT---AGACTTA
8_094     ----------------------------------GGAGTTA--AACAATATGGACCTAACTCTCCTTATATGAGAATATTATTAATTCCATTGCTCATGGAAAT---AGACTTA
8_121     -------------------------------------------GTCAGTAAGGTCCCAATTCGCCTTCTGTCCAGTCACTATTACACTCAGTAACTCAGGACAAG---CGATTGG
8_235     TTGCCCAACCTGCAGTT-----CACTATGGTGAAGGAGCAA--TTCAGACTCACCCTGCAGTGTCTTGTATGGGTCAAACAGTGA---CCACTCCCTAAGGAAAAGTTGGGGGCG
8_525     TTTTCGATAAAAATGCT-AAAAGATATGAAAGAGGGAGTAA--AACAGTATGGACCCAACTCCCCTTATATGAGGACATTATTAGATTCCATTGCTCATGGACAT---AGACTCA
8_559     TTCACCATGAAAATGTT-AAAAGACATAAAGGAAGGAGCTA--AACAATATGGACCCAACTCTCCTTATATGAGAACGTTATTAGATTCCATTGCTCATGGAAAT---AGACTTA
9_307     TTGCCCAACCTACAGTT-----CACTACAGTGAAGGAGCAA--TTCAGACTCGCCCTGCAGTGTCCTGTATGGGTCAAACAGTGG---CCACTCTCTAAGGAAAAGTTGGGGGCG
9_340     TTTTCTATGAAAATGCT-AAAAGACATGAAAGAGGGAGTAA--AACAGTATGGACCCAACTCCCCTTATATAAGAACATTATTAGATTCCACTGCTCATGGACAT---AGACTCA
HML2_Rep  TTTTCGATAAAAAAGCT-AAAAGATATGAAAGAGGGAGTAA--AACAGTATGGACCCAACTCCCCTTATATGAGGACATTATTAGATTCCATTGCTCATGGACAT---AGACTCA
K113      TTTTCGATAAAAATGCT-AAAAGATATGAAAGAGGGAGTAA--AACAGTATGGACCCAACTCCCCTTATATGAGGACATTATTAGATTCCATTGCTCATGGACAT---AGACTCA
K115      TTTTCGATAAAAATGCT-AAAAGATATGAAAGAGGGAGTAA--AACAGTATGGACCCAACTCCCCTTATATGAGGACATTATTAGATTCCATTGCTCATGGACAT---AGACTCA
X_540     TTGCCCAACCGGCAGTT-----CACTGCGGTGAAGGAGCAA--TTCAGACTCGCCCTGTAGCATCCTGTCTGGGTCAAACAGTGG---CCGCTCCCTAAGGAAAAGTTAGGGGCG
Y_026     TTCACCATGAAAATGTT-AAAAGATATGAAGGAAGGAGTTA--AACAATATGGACCCAACTCTCCTTGTATGAGAACATTATTAGATTCCATTGCTCTTGGAAAT---AGACTTA
B270      TTTTCTATAAAAATGCT-AAAAGACATGAAAGAGGGAATAA--AACAGTATGGACCCAACTCCCCTTATATGAGGACATTATTAGATTCCATTGCACACGGACAT---AGACTCA
```

```
             6    7    7    7    7    7    7    7    7    7    7    7    7    7    7    7    7    7    7    7    7    8    8
             9    0    0    1    1    2    2    3    3    4    4    5    5    6    6    7    7    8    8    9    9    0    0
             5    0    5    0    5    0    5    0    5    0    5    0    5    0    5    0    5    0    5    0    5    0    5
10_019   TTCC-TTA--TGATTGGGAGA-TTCTGGCAAAATCGTCTCT-CTCACCCTC-TCAATTTTTACAATTTAAGACTTGGTG-----------GATTGATGGG-----GCACAAGAAC
10_310   TTCC-TTA--TGATTGGGAGA-TTCTGGCAAAATCGTCTCT-CTCACCCTC-TCAATTTTTACAATTTAAGACTTGGTG-----------GATTGATGGG-----GTACAAGAAC
11_233   CTACATAAAGTAGTTAAAAA-CTATTGTAAAAAGGACATGTTTCACCAATG-----------------------------------------------------------------
11_277   TTCC-TTA--TGATTGGGAGA-TTTTGGCCAAATCATCACT-CTCACTGTC-TCAATTTTTACAATTTAAGAGTTGGTG-----------GATTGATGGG-----GCACAAGAAC
11_424   TTCC-TTA--TGATTGGGAGA-TTCTGGCAAAATCGTCTCT-CTCACCCTC-TCAATTTTTACAATTTAAGACTTGGTG-----------GATTGATGGG-----GTACAAGAAC
11_456   TTCC-TTA--TGATTGGGAGA-TTCTGGCAAGATCATCTCT-CTCACCCTC-TCAATTTTTACAATTTAAGACTTGGTG-----------GATTGATGGG-----GTACAAGAAC
12_216   TTCC-TTA--TGATTGGGAGA-TTCTGGCAAAATCGTCTCT-CTTACCCTC-TCAATTTTTACAATTTAAGACTTGGTG-----------GATTGATGGG-----GTACAAGAAC
12_419   TTACATAAATAGTTAAAAAA-CTATTTAAATAGGATGTGTTCCACCCACT------------------------------------------------------------------
14_023   TTCC-TTA--TGATTGGGAA-TTTTGGCTAAATCTTCTCT-TTCACCCTC-TCAGTATCTACAATTTAAAACCTGGTG-----------GATTGACGGA-----GTACAAGAAC
16_010   ---------------------------------------------------------------------------------------------------------------------
17_029   CTCC-GTG--TGATTGGGAGA-TTTTAACGAAAGTTAACT-TTCGCCCTC-CCAATTTCTTCAGTTTAAGACTTGGTG-----------GACCGACCAG-----GCTCAAAATC
19_157   TTCC-TTT--TGATTGGGAGA-TTTTGGCCAAATCATCACT-CTCACCCTC-T-ATCTTTTACAATTTAAGACTTTGTG-----------GATTGATGGG-----GTACAAGAAC
19_238   TTCC-TTA--TGATTGGGAGA-TTCTGGCAAAATCGTCTCT-CTCACCCTC-TCAATTTTTACAATTTAAGACTTGGTG-----------GATTGATGGG-----GCACAAGAAC
19_352   TTCC-TTA--TGATTGGGAGA-TTCTGGCAAAATCGTCTCT-CTCACCCTC-TCAATTTTTACAATTTAAGACTTGGTG-----------GATTGATGGG-----GTACAAGAAC
19_407   TTCT-TTA--TGATTGGGAGA-TTTTGGCCAAATGATCGCT-CTCACCCTC-TCAATGTTTACAATTTAAGACTTGGTGAAGACTTGGTGGATTGATGGG-----GCACAAGAAC
19_639   CTACATAAAATAGTTAAAAAA-CTACTTAAAAA-GGACACGTTTCACCCACT------------------------------------------------------------------
1_059    TTCC-CTA--TGATTGGGAA-CGTTGGCTAAATCTTCCCT-TTCACCCTC-TCAGTTCCTACAATTTAAAACCTGGTG-----------GATTGATGGA-----GTACAAGAAC
1_061    TTCC-CTA--TGATTGGGAA-TGTTGGCTAAATCTTCCCT-TTCACCCTC-TCAGTTCCTACAATTTAAAACCTGGTG-----------GATTGATGGA-----GTACAAGAAC
1_259    TTCC-TTA--TGATTGGGAGA-TTCTGGCAAAATCGTCTCT-CTCACCCTC-TCAATTTTTACAATTTAAGACTTGGTG-----------GATTGATGGG-----GTACAAGAAC
1_504    TTCC-TTA--TGATTGGGAGA-TTCTGGCAAAATCGTCTCT-CTCACCCTC-TCAATTTTTACAATTTAAGACTTGGTG-----------GATTGATGGG-----GTACAAGAAC
1_555    TTCC-TTA--TGATTGGGAGA-TTCTGGCAAAATCATCTCT-CTCACCCTC-TCAATTTTTACAATTTAAGACTTGGTG-----------AATTGATGGG-----GCACAAGAAC
20_078   TTCC-TTA--TGACTGGGAA-TTTTGGCCAAATCATCACT-CTCACCTTC-TCAATTTTTACAATTTAAGACCTGGTG-----------GATTGATGGG-----GTACAACAAC
21_019   TTCC-TTA--TGATTAGGAGA-TTCTGGCAAAATCGTCTCT-CTCACCCTC-TCAATTTTTACAATTTAAGACTTAGTA-----------GATTGATCAG-----GTACAAGAAC
22_012   TTCC-TTA--TGATTGGGAGA-TTCTGGCAAAATCGTCTCT-CTCACCCTC-TCAATTTTTACAATTTAAGACTTGGTG-----------GATTGATGGG-----GTACAAGAAC
22_041   CTCC-TTA--TGACTGGGAA-TTTTGGCCAAATCTTCCCT-TTCATCCTC-TCAGTATCTACAGTTTAAAACCTGGTG-----------GATTGATGGA-----GTACAAGAAC
2_Tan    TTCC-TTA--TGATTGGGAGA-TTCTGGCAAAATCGTCTCT-CTCACCCTC-TCAATTTTTACAATTTAAGACTTGGTG-----------GATTGATGGG-----GTACAAGAAC
3_014    TTCC-TTA--TGATTGGGAGA-TTCTGGCAAAATCGTCTCT-CTCACCCTC-TCAATTTTTACAATTTAAGATTTGGTG-----------GATTAATGGG-----GCACAAGAAC
3_209    TTCC-TTA--AGATTGGGAA-TTTTGGTTAAATCTTCCCT-CTCACCCTC-TCAGTATCTACAGTTTAAAACCTGGTG-----------GATTGATGGG-----GTACAAGAAC
3_294    CTCC-TTA--TGACTGGGAA-GTTTGGCCAAATCTTCCCT-TTCATCCTC-TCAGTATCTACAGTTTAAAACCTGGTG-----------GATTGATGGA-----GTACAAGAAC
3_354    TTCC-TTA--TGATTGGGAGA-TTCTGGCAAAATCGTCTCT-CTCACCCTC-TCAATTTTTACAATTTAAGACTTGGTG-----------GATTGATGGG-----GTACAAGAAC
3_385    TTCC-TTA--TGATTGGGAGA-TTCTGGCAAAATCGTCTCT-CTCACCCTC-TCAATTTTTACAATTTAAGACTTGGTG-----------GATTGATAGA-----GTACAAGAAC
3_496    TTCC-TTA--TGATTGGGAGA-TTCTGGCAAAATCGTCTCT-CTCACCCTC-TCAATTTTTACAATTTAAGACTTGGTG-----------GATTGATGGG-----GTACAAGAAC
3_610    TTCC-TTA--TGATTGGGAGA-TTCTGGCAAAATCGTCTCT-CTCACCCTC-TCAATTTTTACAATTTAAGACTTGGTG-----------GATTGATGGG-----GTACAAGAAC
4_008    TTCC-TTA--TGATTGGGAA-TTTTGGCTAAATCTTCCCT-TTCACCCTC-TCA---TTTACAGTTTAAAACCTGGTG-----------GATTGATGGAATGGAGTACAAGAAC
4_032    TTCC-TTA--TAATTGGGAA-TTTTGGCTAAATCTTCCCT-TTCACCCTC-TCAGTATCTACAGTTTAAAACCTGGTG-----------GATTGATGGG-----GTACAAGAAC
4_052    TTTC-TTA--TGATTGGGAA-TTTTGGCTAAATCTTCCCT-TTCACCCTC-TCAGTATCTACAGTTTAAAACCTGGTG-----------GAATGATGGG-----GTACAAGAAC
4_068    TTCC-TTA--TAATTGGGAA-TTTTGGCTAAATCTTCCCT-TTCACCCTC-TCAGTATCTACAGTTTAAAACCTGGTG-----------GATTGATGGG-----GTACATGAAC
4_716    TTCC-TTA--TGATTGGGAGA-TTCTGGCAAAATCGTCTCT-CTTACCCTC-TCAATTTTTACAATTTAAGACTTGGTG-----------GATTGATGGG-----GTACAAGAAC
4_742    TTCC-TTA--TGATTGGGAGA-TTCTGGCAAAATCGTCTTT-CTCACCCTC-TCAATTTGTACAATTTAAGACTTGGTG-----------GATTGATAGG-----ACACAAGAAC
5_147    TTCC-TTA--TGATTGGGAGA-TTCTGGCAAAATCGTCTCT-CTCACCCTC-TCTATTTTTACAATTTAAGACTTGGTG-----------GATTGATGGG-----GTACAAGAAC
5_259    TTCC-TTA--TGATTGGGAGA-TTTTGGCAAAATCGTCTTC--TCACCCTC-TCAATTTTTACAATTTAAGACTTGGTG-----------GATTGATGGG-----TAACAAGAAC
5_535    TTCC-TTA--TGATTGGGAA-TTTTGGCTAAGTCTTCCCT-TTCACCCTC-TCAGTTTCTACAGTTTAAAACCTGGTG-----------GATTGATGGA-----GTACAAGAAC
5_544    TTCC-TTA--TGATTGGGAGA-TTCTGGCAAAATCGTCTCT-CTCACCCTC-TCAATTTTTACAATTTAAGACTTGGTG-----------GATTGATGGG-----GTACAAGAAC
6_098    TTCC-TTA--TGATTGGGAGA-TTTTGGCCAAATCATCACT-CTCATCCTC-TCAATTTTTACAATTTAAGACTTGGTG-----------AATTGATGGG-----GCACAAGAAC
6_178    TTCC-TTA--TGACTGGGAA-TTTTGGCTAAATCTTCCCT-TTCACCCTC-TCAGTTTCTACAGTTTAAACCGTGGTG-----------GATCGATGGA-----GTACAAGAAC
6_240    CTACATAAAATAGTTAAAAAA-CTATTTAAAAAGGACATGTTTCACACACT------------------------------------------------------------------
6_324    TTCC-TTA--TGATTGGGAGA-TTCTGGCAAAATCGTCTCT-CTCACCCTC-TCAATTTTTACAATTTAAGACTTGGTG-----------GATTGATGGG-----GTACAAGAAC
7_345    TTCC-TTA--TGATTGGGAGA-TTCTGGCAAAATCGTCTCT-CTCACCCTC-TCAATTTTTACAATTTAAGACTTGGTG-----------GATTGATGGG-----GTTCAAGAAC
7_450    TTCC-TTA--TGATTGGGAGG-TTCTGGCCAAATTGTCTCT-CTCACCCTC-TCAATTTTAACAATTTAAGACTTGGTG-----------GATTGATGGG-----GCACAACAAC
8_047    TTTC-TTA--TGATTGGGAA-TTCTGGCTAAATCTTCCCT-TTCACCCTC-TCAGTATCTCCAGTTTAAAACCTGGTG-----------GATTGATGGG-----GTACAAGAAC
8_074    TTTC-TTA--TGATTGGGAA-TTCTGGCTATATCTTCCCT-TTCACCCTC-TCAGTATCTCCAGTTTAAAACCTGGTG-----------GATTGATGGG-----GTACAAGAAC
8_094    TTTC-TTA--TGATTGGGAA-TTCTGGCTATATCTTCCCT-TTCACCCTC-TCAGTATCTCCAGTTTAAAACCTGGTG-----------GATTGATGGG-----GTACAAGAAC
8_121    CCCC-GGA--CGATTGGGAGA-TTTTAGCAAAAGCTACTCT-TTCGCCCTC-CCAATTTCTTCAATTTAAAAGTTGGTG-----------GACCGATCAG-----GCTCAAAATC
8_235    CTGCATAAAATAGTTAAAAA-CTATTTAAAAAGGACATGTTTCACTCACT-------------------------------------------------------------------
8_525    TTCC-TTA--CAATTGGGAGA-TTCTGGAAAAATCGTCTCT-CTCACCCTC-TCAATTTTTACAATTTAAGACTTGGTG-----------GATTGATGGG-----GTACAAGAAC
8_559    TTCC-TTA--TGATTGGGAA-TTTTACCTAAATCTTCCCT-TTCACCCTC-TCAGTATCTACAGTTTAAAACCTGGTG-----------GATTGATGGA-----GTACAAGAAC
9_307    CTACATAAAATAGTTAAAAAA-CTATTTAAAAAGGACATGTTTCACCCACT------------------------------------------------------------------
9_340    TTCC-TTA--TGATTGGGAGA-TTTTGGCCAAATCATCACT-CTCACC---TCAATTTTTACAATTTAAGACCTGGTG-----------GATTGATGGG-----ACACAAGAAC
HML2_Rep TTCC-TTA--TGATTGGGAGA-TTCTGGCAAAATCGTCTCT-CTCACCCTC-TCAATTTTTACAATTTAAGACTTGGTG-----------GATTGATGGG-----GTACAAGAAC
K113     TTCC-TTA--TGATTGGGAGA-TTCTGGCAAAATCGTCTCT-CTCACCCTC-TCAATTTTTACAATTTAAGACTTGGTG-----------GATTGATGGG-----GTACAAGAAC
K115     TTCC-TTA--TGATTGGGAGA-TTCTGGCAAAATCGTCTCT-CTCACCCTC-TCAATTTTTACAATTTAAGACTTGGTG-----------GATTGATGGG-----GTACAAGAAC
X_540    CTACATAAAATAGTTAAAAAACTATTTAAAAAGGACATGTTTCACCCACT-------------------------------------------------------------------
Y_026    TTCC-TTA--GGATTGGGAA-TTTTGGCTAAATCTTCCCT-TTCACCCTC-TCAATTCCTACAGTTTAAAACCTGGTA-----------GATTGATGGA-----GTACAAGAAT
B270     TTCC-TTA--TGATTGGGAGA-TTCTGGCAAAATCGTCTCT-CTCACCCTC-TCAATTTTTACAATTTAAGATTTGGTG-----------GATTAATGGG-----GCACAAGAAC
```

10_019	A
10_310	A
11_233	-
11_277	A
11_424	A
11_456	A
12_216	A
12_419	-
14_023	A
16_010	-
17_029	A
19_157	A
19_238	A
19_352	A
19_407	A
19_639	-
1_059	A
1_061	A
1_259	A
1_504	A
1_555	A
20_078	A
21_019	G
22_012	A
22_041	A
2_Tan	A
3_014	A
3_209	A
3_294	A
3_354	A
3_385	A
3_496	A
3_610	A
4_008	A
4_032	A
4_052	A
4_068	A
4_716	A
4_742	A
5_147	A
5_259	A
5_535	A
5_544	A
6_098	A
6_178	A
6_240	-
6_324	A
7_345	A
7_450	A
8_047	A
8_074	A
8_094	A
8_121	A
8_235	-
8_525	A
8_559	A
9_307	-
9_340	A
HML2_Rep	A
K113	A
K115	A
X_540	-
Y_026	A
B270	A

```
          1   5    10   15   20   25   30   35   40   45   50   55   60   65   70   75   80   85   90   95   100  105  110  115
10_019    AGGACC---AAGTCCTCTTCCAGTAGGTCAGATGCCCGTAACATTACAACCTCAAACGCAGGTTAG-------------------------------------------------
10_310    AGGGCC---AAGTCCTCTTCCAGCAGGTCAGGTGCCCGTAACACTACAACCTCAAACGCAGGTTAA-------------------------------------------------
11_233    -------------------------------------------------------------------------------------------------------------------
11_277    ATGGCC---AACTCCTCTTCCAGCGGTTCAGATGCCTGTAACATTACAACCTCAAATGCAGGTTAGACAAGTACAAACCCCAAGAGAATATCAAATAGAGAG--------TCTCT
11_424    AGGCAC---AAGTCCTCTTCCAGCAGGTCAGGTGCCCGTAAGATTACAACCTCAAACGCAGGTTAA-------------------------------------------------
11_456    AGGGCC---AAGTCCTCTTCCAGCAGGTCAGGTGCCCGTAACATTACAACCTCAAACGCAGGTTAG-------------------------------------------------
12_216    AGGCAC---AAGTCCTCTTCCAGCAGGTCAGGTGCCCGTAACATTACAACCTCAAACGCAGGTTAA-------------------------------------------------
12_419    -------------------------------------------------------------------------------------------------------------------
14_023    ATGGCCAACAACTCCTTCTCCCACGGTTCAGATGCCTGTGACATTACAACCTCAAA-----------------------------------------------------------
16_010    ATGGC---CAACTCCTCCTCCCGTGGTTCAGATACCTGTAACATTACAACCTCAAATGCAGGTTAGACAAGTACAAACCCCAAGAGAATATCAAATAGAAAAGGATAG--TCTCT
17_029    -------------------------------------------------------------------------------------------------------------------
19_157    ATGGCC---AACTCCTCTTCCAGCAGTTCAGATGCCTGTAACATTACAACCTCAAATGCAGGTTAGACAAGTACAAACCTGAAGAGAATAACAAATAGAGAAAGATAGAGTATCT
19_238    AGGGCC---ATGTCCTCTTCCAGCAGGTCAGATGCCCGTAATATTACAACCTCAAACGCAGTTTAG-------------------------------------------------
19_352    AGGCAC---AAGTCGTCTTCCAGCAGGTCAGGTGCCCGTAACATTACAACCTCAAACGCAGGTTAA-------------------------------------------------
19_407    ATGGCC---AACTCCTCTCCCAGCAGTTCAGATGCCTGTAACATTACAACCTCAAATGCAGGTTAGACAAGTACAAACCCCAAAAGAATATCAAATAGGGAAAGATAGAGTCTCT
19_639    -------------------------------------------------------------------------------------------------------------------
1_059     ATGGCCATCAACTCCTCCTCCAGTGGTTCAGATGCCTGTAACATTACAACCTCAAATGCAGGTTAGACAAGTGCAAACCCCAAGAGAATATCAAGTAGAAAAGGATAGAGTCTCT
1_061     ATGGCCATCAACTCCTCCTCCAGCGGTTCAGATGCCTGTAACATTACAACCTCAAATGCAGGTTAGACAAGTGCAAACCCCAAGAGAATATCATGTAGAAAAGGATAGAGTCTCT
1_259     AGGCAC---AAGTCCTCTTCCAGCAGGTCAGGTGCCCGTAAGATTACAACCTCAAACGCAGGTTAA-------------------------------------------------
1_504     AGGCAC---AAGTCATCTTCCAGCAGGTCAGGTGCCCGTAACATTACAACCTCAAAAGCAGGTTAA-------------------------------------------------
1_555     AGGGCC---AAGTCCTCTTTCAGCAGGTCAGGTGACCGTAACATTACAACCTCAAGCGCAGGTTAG-------------------------------------------------
20_078    ATGGCC---AACTCCTCCTCCCACAGTTCAGATGCCCGTAACATTACAACCTCAAATGCAGGTTAGACAAGTACAAACCCCAAGAGAATATCAGATAGAAAAGGATAG--TCTCT
21_019    AAGGCC---AAGTCCTCTTCCAGCAAGTCAGGTGCCTGTAACATTACAACCTCAAACGCAGGTTAA-------------------------------------------------
22_012    AGGGAC---AAGTCCTCTTCCAGCAGGTCAGGTGCCCGTAACATTACAACCTCAAAAGCAGGTTAA-------------------------------------------------
22_041    ATCGCCATCAACTCCTCCTCCCGCGGTTCAGATGCCTGTAACATTACAACCTCAAACGCAGGTTAGACAAGCACAAACCCCAAGAGAAAATCAAGTAGAAAGGGACAGAGTCTCT
2_Tan     AGGCAC---AAGTCCTCTTCCAGCAGGTCAGGTGCCTGTAACATTACAACCTCAAAAGCAGGTTAA-------------------------------------------------
3_014     AGGGAC---AAGTCCTCTTCCAGCAGGTCAGATGCCCGTAACATTACAACCTCAAACACAGGTTAG-------------------------------------------------
3_209     ATGGCCACCATCTCCTTCTCCCGCGGTTCAGATACCTGTGACATCACAATCTCAAA-----------------------------------------------------------
3_294     AGGGCC---AAGTCCTCTTCCAGCAGGTCAGGTGCCCGTAACATTACAACCTCAAACGCAGGTTAA-------------------------------------------------
3_354     AGGCAC---AAGTCATCTTCCAGCAGGTCAGGTGCCCGTAACATTACAACCTCAAAAGCAGGTTAA-------------------------------------------------
3_385     AGGCAC---AAGTCCTCTTCCAGCAGGTCAGGTGCCTGTAACATTACAACCTCAAATGCAGGTTAA-------------------------------------------------
3_496     AGGGCC---AAGTCCTCTTCCAGCAGGTCAGGTGCCCGTAACATTACAACCTCAAAAGCAGATTAA-------------------------------------------------
3_610     AGGCAC---AAGTCCTCTTCCAGCAGGTCAGGTGCCTGTAACATTACAACCTCAAACGCAGGTTAA-------------------------------------------------
4_008     -------------------------------------------------------------------------------------------------------------------
4_032     ATGGCCACCAACCCCTTCTCCCGCGGTTCAGATGCCTGTGATGTCACAATCTCAAA-----------------------------------------------------------
4_052     ATGGCCACCAACTCCTTCTCCCGCGGTTCAGATGCCTTTGATGTCACAATCTCAAA-----------------------------------------------------------
4_068     ATGGCCACCAACCCCTTCTCCCGCGGTTCAGATGCCTGTGATGTCACAATCTCAAA-----------------------------------------------------------
4_716     AGGGCC---AAGTCCTCTTCCAGCAGGTCAGGTGCCCGTAACATTACAACCTCAAAAGCAGGTTAA-------------------------------------------------
4_742     AGAGCC---AAGTCCTTTTCTAGCAGGTCAGATGCCCATAACATTACAACCTCAAACGCAGGTTAG-------------------------------------------------
5_147     AGGCAC---AAGTCCTCTTCCAGCAGGTCAGGTGCCTGTAACATTACAACCTCAAACGCAGGTTAA-------------------------------------------------
5_259     AGCACC---AAGCCCTCTTCCAGCAGGTGAGATGCCTGGAACATTACAGTCTCCAATGCAGGTTAG-------------------------------------------------
5_535     ACGGCCATCAACTCCTCCTCCTGCGGTTCAGATGCCTGTGACATTACAACCTCAAATGCAGGTTAGACAAGTACAAACTGCAAGAGAATATCAAGTAGAAAAGGATAGAGTCTCT
5_544     AGGCAC---AAGTCCTCTTCCAGCAGGTCAGGTGCCCGTAAGATTACAACCTCAAAAGCAGGTTAA-------------------------------------------------
6_098     ATGGCC---AACTCCTCTTCCAGCAGTTCAGATGCCTGTAACATTGCAACCTCAAATGCAGGTTAGACAAGTACAAACCCCAGAAGAATATCAAATAGAAAAAGATAAAGTCTCT
6_178     ATGGCCATCAA---CTCCTCCCGTGGTTCAGATACCTGTAACATTACAACCTCAAAGGGAGGTTAGACAAGTACAAACCCCAAGAGAATATCAAGTAGAAAAGGATAGAGTCTCT
6_240     -------------------------------------------------------------------------------------------------------------------
6_324     AGGCAC---AAGTCCTCTTCCAGCAGGTCAGGTGCCTGTAACATTACAACCTCAAAAGCAGGTTAA-------------------------------------------------
7_345     AGGCAC---AAGTCCTCTTCCAGCAGGTCAGGTGCCTGTAACATTACAACCTCAAACGCAGGTTAA-------------------------------------------------
7_450     AGGGCC---AACTCCTCTTCCAGCAGTTCAGATGCCTGTAACATTACAACCTCAAATGCAGGTTAGGCAAGTACAAACCCCAAGAGAATATCAAATAGAGAAAGATAGAGTCTCT
8_047     ATGGCCACCAACTCCTTCTCCCGCGGTTCAGATGCCTGTGATGTCACAATCTCAAA-----------------------------------------------------------
8_074     ATGGCCACCAACTCCTTCTCCCGCGGTTCAGATGCCTGTGATGTCACAATCTCAAA-----------------------------------------------------------
8_094     ATGGCCACCAACTCCTTCTCCCGCGGTTCAGATGCCTGTGATGTCACAATCTCAAA-----------------------------------------------------------
8_121     -------------------------------------------------------------------------------------------------------------------
8_235     -------------------------------------------------------------------------------------------------------------------
8_525     AGGCAC---AAGTCCTCTTCCAGCAGGTCAGGTGCCCGTAACATTACAACCTCAAAAGCAGGTTAA-------------------------------------------------
8_559     ATGGCCACCAACTCCTTCTCCTGTGGTTCAGATGCCTGTGACATTACAACCTCAAA-----------------------------------------------------------
9_307     -------------------------------------------------------------------------------------------------------------------
9_340     ATGGCCATCAACTCCTCCTCCCGCGGTTCAGATGCCTGTAACATTACAACCTCAAATGCAGGTTAGACAAGTACAAACCCCAAGAGAATATCAAATAGAAAAGGAAAGAGTCTCT
HML2_Rep  AGGCAC---AAGTCCTCTTCCAGCAGGTCAGGTGCCTGTAACATTACAACCTCAAAAGCAGGTTAA-------------------------------------------------
K113      AGGCAC---AAGTCCTCTTCCAGCAGGTCAGGTGCCCGTAACATTACAACCTCAAAAGCAGGTTAA-------------------------------------------------
K115      AGGCAC---AAGTCCTCTTCCAGCAGGTCAGGTGCCCGTAACATTACAACCTCAAAAGCAGGTTAA-------------------------------------------------
X_540     -------------------------------------------------------------------------------------------------------------------
Y_026     ATGGCCACCAACTCCTTCTCCCGCGGTTCAGATGCCTGTGACATTACAACCTCAAA-----------------------------------------------------------
93        AGGGCC---AACTCCTCTTCCAGCAGTTCAGATGCCTGTAACATTACAACCTCAAATGCAGGTTAGGCAAGTACAAACCCCAAGAGAATATCAAATAGAGAAAGATAGAGTCTCT
```

```
          120  125  130  135  140  145  150  155  160  165  170  175  180  185  190  195  200  205  210  215  220  225  230
10_019    -----------------------------------------------AGAAAATAAGACCCAACTACCAGTAGCTTATCAATACTGGCCACCGGCCAAACTTCAGTATCGGCCAC
10_310    -----------------------------------------------AGAAAATAAGACCCAACCGCCAGTAGCTTATCAATACTGGCCGCCAGCCGAACTTCAGTATCGGCCAC
11_233    -------------------------------------------------------------------------------------------------------------------
11_277    GTCATGGCAACGCCAATCCAAATACAGTATCCACAATATCAGCCGGTAGAAAATAAGACCCAACCGCCAGTAGCCTATCAATACTGGCCGCCAGCCGAACTTCAGTATCGGCCGC
11_424    -----------------------------------------------AGAAAATAAGACCCAACCGCCAGTAGCTTATCAATACTGGCCGCCGGCTGAACTTCAGTATCGGCCAC
11_456    -----------------------------------------------AGAAAATAAGAGCCAACCACCAGTAGCTTATCAATACTGGCCGCTGGCTGAACTTCAGTATCAGCCAC
12_216    -----------------------------------------------AGAAAATAAGACCCAACCACCAGTAGCTTATCAATACTGGCCGCCGGCTGAACTTCAGTATCGGCCAC
12_419    -------------------------------------------------------------------------------------------------------------------
14_023    ----------TGCCAATCCAGACACAGTATCCACAATATCAGCCGGTAGAAAATAAAACCCAACCATCAGTAGTTTATGAACACCAGCCATCAGCCGAATTTCAGTATCGGCCGT
16_010    ATCTCGGCAATGCCAATCCAGATACAGTATCCACAATATCAGCCAGTAGAAAATAAAACCCAACCGCCATTAGCTTATCAATACTGGCTGCCAGCCGAGCTTCAGTATTGGCTGC
17_029    -------------------------------------------------------------------------------------------------------------------
19_157    GTCACAGCAATGCCAATCCAAATACAGTATCCACAATATCAGCTGGTAGAAAATAAGACCCAACTGCCAGTAGCCTATCAATACTGGCCGCCAGCCGAACTTCAGTATTGGCCGC
19_238    -----------------------------------------------AGAAAATAAGACCCAACCGCCAGTAGCTTATCAATAATGGCCGCCAGCCAAACTTCAGTATCGGCCAC
19_352    -----------------------------------------------AGAAAATAAGACCCAACCGCCAGTAGCCTATCAATACTGGCCGCCGGCTGAACTTCAGTATCGGCCAC
19_407    GTCACGGCAATGCCAATCCAAATACAGTATCCACAATATCAGCCGGTGGAAAATAAGACCCAACCGCCAGTAACCTATCAATACTGGCCGCCAGCCGAACTTCAGGATCAGCCGC
19_639    -------------------------------------------------------------------------------------------------------------------
1_059     ATCCCAGCAATGCCAATTCAGATACAGTATCCACAATATCAGCTGGTAGAAAATAAGACCCAACCACCGGTAGTTTATCAATACTGGCCGCCAGCTGAGCTTCAGTATGGGCTGT
1_061     ATCCCGGCCATGCCAATTCAGATACAGTATCCACAATATCAGCTGGTAGAAAATAAGACCCAACCACCGGTAGTTTATCAATACTGGCCGCCAGCTGAGCTTCAGTATGGGCCGT
1_259     -----------------------------------------------AGAAAATAAGACCCAACCGCCAGTAGCTTATCAATACTGGCCGCCGGCTGAACTTCAGTATCGGCCAC
1_504     -----------------------------------------------AGAAAATAAGACCCAACCGCCAGTAGCCTATCAATACTGGCCTCCGGCTGAACTTCAGTATCGGCCAC
1_555     -----------------------------------------------AGAAAATAAGACCCAACTGCCAGTAGCTTATCAATACTGGCCACCGGCCGAACTTCAGTATCGGCCAC
20_078    ATCACAGCAATGCCAATCCAGATACAGTATTCACAATATCAGCTGGTAGAAAGTAAGACCCAACCGCCAGTACCCTATCAATACTGACCGCCAGCCGAACTTCAGTATCAGCCGC
21_019    -----------------------------------------------AGAAAATAAGACCCAACCGCCAGTAGCTTATCAATACTAGCCGCCGGCTGAACTTCAGTATCGGCCAC
22_012    -----------------------------------------------AGAAAATAAGACCCAACCGCCAGTAGCCTATCAATACTGGCCTCCGGCTGAACTTCAGTATCGGCCAC
22_041    ATCCCGGCAATGCCAACTCAGATACAGTATCCACAATATCAGCCGGTAGAAAATAAGACCCAACCGCTGGTAGTTTATCAATACCGGCTGCCAACCGAGCTTCAGTATCGGCCTC
2_Tan     -----------------------------------------------AGAAAATAAGACCCAACCGCCAGTAGCCTATCAATACTGGCCTCCGGCTGAACTTCAGTATCGGCCAC
3_014     -----------------------------------------------AGAAAATAAGACCCAACCACCAGTAGCTTATCAATACTGGCTGCCGGCTGAATTTCAGTATCGGCCAC
3_209     ----------TGCTAATCCAGGCACAGTATCCACAATAGCAGGCAGTAGAAAATAAAACCCAACCATCGGTAGTTTATCAACACCAGCCGCCAGCCGAATTTCAGTATCGGCCGT
3_294     -----------------------------------------------AGAAAATAAGACCCAACCGCCAGTAGCTTATCAATACTGGCCGCCGGCTGAACTTCAGTATCTGCCAC
3_354     -----------------------------------------------AGAAAATAAGACCCAACCGCCAGTAGCCTATCAATACTGGCCGCCGGCTGAACTTCAGTATCGGCCAC
3_385     -----------------------------------------------AGAAAATAAGACCCAACCGCCAGAAGCTTATCAATACTGGCCGCCGGCTGAACTTCAGTATCGGCCAC
3_496     -----------------------------------------------AGAAAATAAGACCCAACCGCCAGTAGCTTATCAATACTGGCCGCCGGCTGAACTTCAGTATCGGCCAC
3_610     -----------------------------------------------AGAAAATAAGACCCAACCGCCAGTAGCCTATCAATACTGGCCACCGGCTGAACTTCAGTATCGGCCAC
4_008     -------------------------------------------------------------------------------------------------------------------
4_032     ----------TGCCAATCCAGGCACAGTATCCGCAATACCAGCCCGTAGAAAATAAA-CCCAACCATCGGTAGTTTATCAACACCAGCCGCCAGCCGTATTTCAGTATCCGCCGT
4_052     ----------TGCCAATCCAGGCACAGTATCCGCAATACCAGCCAGTAGAAAATAAAACCCAACCATCGGTAGTTTATCAACACAAGCCGTCAGCCGTATTTCAGTATCCGCCGT
4_068     ----------TGCCAATCCAGGCACAGTATCCGCAATACCAGCCAGTAGAAAATAAA-CCCAACCATCGGTAGTTTATCAACACCAGCCGCCAGCCGAATTTCAGTATCGGCCGT
4_716     -----------------------------------------------AGAAAATAAGACCCAACCGCCAGTAGCTTATCAATACTGGCCGCCGGCTGAACTTCAGTATCGGCCAC
4_742     -----------------------------------------------AGAAAATAAGACCCAACCACCAGTAGCTTATCAATACTGGCCACTGGCTGAACTTCAGTATCAGCCAC
5_147     -----------------------------------------------AGAAAATAAGACCCAACCGCCAGTAGCCTATCAATACTGGCTGCCGGCTGAACTTCAGTATCGGCCAC
5_259     -----------------------------------------------AGAAAATAAGACCCAACCACCAGTAGCTTAGCAATACTGGCCACTGGCCAAACTTCAGCATCAGCCAT
5_535     ATCCCGGCAATGCCAATCCAGACACAGTATCCACAATATCAGCTGGTAGAAAATAAAACCCAACCACTGGTAGTTTATCAATACTGGCCGCCAGTCAAATTTCAGTATCAGCTGC
5_544     -----------------------------------------------AGAAAATAAGACCCAACCGCCAGTAGCCTATCAATACTGGCCGCCGGCTGAACTTCAGTATCGGCCAC
6_098     GCCATGGCAATGCCAATCCAGATACAGTATCCACAATATCAGCAGGTAGAAAATAAGACCCAACTGCCAGTAGCCTATCAATACTGGCCGCCAGCCGAACTTCAGTATCGGCCGC
6_178     ATCCCGGCAATGCCAATTCAGATGCAGTATCCACAATATAAGCTGGTAGAAAATAAGACCCAACCACCAGTAATTTATCAGTACTGGCCGCCAGCCGACCTTCAGTATCGGCCAC
6_240     -------------------------------------------------------------------------------------------------------------------
6_324     -----------------------------------------------AGAAAATAAGACCCAACCGCCAGTAGCCTATCAATACTGGCCTCCGGCTGAACTTCAGTATCGGCCAC
7_345     -----------------------------------------------AGAAAATAAGACCCAACCGCCAGTAGCCTATCAATACTGGCCGCCGGCTGAACTTCAGTATCGGCCAC
7_450     GTCACGGCAATGCCAATCCAAATACAGTATCCACAATATCAGCCGGTAGAAAATAAGACCCAACTGCCAGTAGCCTATCAATACTGGCTGCCAGCCGAACTTAAGTGTCGGCCAC
8_047     ----------TGCCAATCCAGGCACAGTATCCGCAATACCAGCCAGTAGAAAATAAAACCCAACCATCGGTAGTTTATCAACACCAGCCGCCAGCCGCATTTCAGTATCCGCCGT
8_074     ----------TGCCAATCCAGGCACAGTATCCGCAATACCAGCCAGTAGAAAATAAAACCCAACCATCGGTAGTTTATCAACACCAGCCGCCAGCCGCATTTCAGTATCCGCCGT
8_094     ----------TGCCAATCCAGGCACAGTATCCGCAATACCAGCCAGTAGAAAATAAAACCCAACCATCGGTAGTTTATCAACACCAGCCGCCAGCCGCATTTCAGTATCCGCCGT
8_121     -------------------------------------------------------------------------------------------------------------------
8_235     -------------------------------------------------------------------------------------------------------------------
8_525     -----------------------------------------------AGAAAATAAGACCCAACCACCAGTAGCCTATCAATACTGGCCGCCGGCTGAACTTCAGTATCGGCCAC
8_559     ----------TGCCAATACAGACACAGTATCTACAATATCAGCTGGTTGAAAATAAAACCCAACCACTGGTAGTTTATCAACACTGGCCGCCAGCCGAATTTCAGTATCGGCCAT
9_307     -------------------------------------------------------------------------------------------------------------------
9_340     ATCCCAGCAATGCCAATCCAGATGCAGTATCCACAATATCAGCTGGTAGAAAATAAGACCCAACTGCCGGTAGCCTATCAATACTGGCCGCCAGCCAAACTTCAGTATCGGCCGC
HML2_Rep  -----------------------------------------------AGAAAATAAGACCCAACCGCCAGTAGCCTATCAATACTGGCCTCCGGCTGAACTTCAGTATCGGCCAC
K113      -----------------------------------------------AGAAAATAAGACCCAACCGCCAGTAGCCTACCAATACTGGCCGCCGGCTGAACTTCAGTATCAGCCAC
K115      -----------------------------------------------AGAAAATAAGACCCAACCGCCAGTAGCCTATCAATACTGGCCTCCGGCTGAACTTCAGTATCGGCCAC
X_540     -------------------------------------------------------------------------------------------------------------------
Y_026     ----------TGCCAATCCAGACACAGTATCCACAATATCAGCTGGTGGAAAATAAAACCCAACCACCGGCAGCTTATCAACACCAGCCGCCAGCCGAATTTCAGTATCGGCCGT
93        GTCACGGCAATGCCAATCCAAATACAGTATCCACAATATCAGCCAGTAGAAAATAAGACCCAACTGCCAGTAGCCTATCAATACTGGCTGCCAGCCGAACTTAAGTGTCGGCCAC
```

```
          235 240 245 250 255 260 265 270 275 280 285 290 295 300 305 310 315 320 325 330 335 340 345
10_019    CCCC-AGAAAGTCAGTATGGATATCCAGGAACGCTCCCAGCACCACAGGGCAG----GGCGCCATACCCTCAGCCACCCACTAGCAGAC--------------------------
10_310    CCCC-AGAAAGTCAGTATGGATATCCAGGAATGCCCCCAGCACCACAGGGCAG----GGCGCCATACCCTCAGCCGCCCACTAGGAGAC--------------------------
11_233    -------------------------------------------------------------------------------------------------------------------
11_277    CCCC-AGAAAATCAGTATGGACAGCCAGGAGTGTTTCCAGCACCACAGGGCAG----GGCGCTATAGCCTCAGCCGCCCACTATAAGAC--------------------------
11_424    CCCC-AGAAAGTCAGTATGGATATCCAGGAATGCCCCCAGCACCACAGGGCAG----GGCGCCATACCCTCAGCCACCCACTAGGAGAC--------------------------
11_456    CCCC-AGAAAGTCAGTATGGATATCCAGGAATGCCCCCAGCACCACAGGGCAG----GGCGCCATACCCTCAGCCGCCCACTAGGAGAC--------------------------
12_216    CCCC-AGAAAGTCAGTATGGATATCCAGGAATGCCCCCAGCACCACAGGGCAG----GGCGCCATACCCTCAGCCGCCCACTAGGAGAC--------------------------
12_419    -------------------------------------------------------------------------------------------------------------------
14_023    CTCC-AGTGGTTCAGTATAGATCCCAGGTGTTGTGTCCTGTGCCAAATAGCAG----GGCAGTATAGCAACAACCCATGGCGATGGTGT--------------------------
16_010    CTCC-AGAGGTCCAATACAGACCTCAAGTGGTGTGTGCCATGCCAAATTGCAC----GGCATCGTACAAGCAACCCATGGCGGTGGTGTTTAATACGTCAGCACCACAGGGCGCG
17_029    -------------------------------------------------------------------------------------------------------------------
19_157    CCCT-AGAAAATCAGCATGGACAGCCAGGAATGTTTCCAGCACCACAGGGCAG----GGCACTATATCCGCAGCCTCCCACTATGAGAC--------------------------
19_238    CCCC-AGAAAGTCAGTATGGATATCCAGGAACGCTCCCAGCACCACAGGGCAG----GGCGCCATACCCTCAGCCACCCACTAGGAGAC--------------------------
19_352    CCCT-AGAAAGTCAGTATGGATATCCAGGAATGCCCCCAGCACCACAGGGCAG----GGCGCCATACCCTCAGCCGCCCACTAGGAGAC--------------------------
19_407    CCCC-AGAAAATCAGTATGGACAGCCAGGAATGTTTCCAGCACCACAGGGCAG----GGCGCTATATCCTCAGCAGCCCACTATGAGAT--------------------------
19_639    -------------------------------------------------------------------------------------------------------------------
1_059     CTCC-GGAGGTTCAATACAGACCTCAAGCTGTGTGTCCCGTGCCACATAGCAC----GGCACCTCACCAGCAACCCA-----GTGGTGT--------------------------
1_061     CTCC-GGAGGTTCAATACAGACCTCAAGCTGTGTGTCCCGTGCCACACAGCAC----GGCACCTCACCAGCAACCCA-----GCGGTGT--------------------------
1_259     CCCC-AGAAAGTCAGTATGGATATCCAGGAATGCCCCCAGCACCACAGGGCAG----GGCGCCATACCCTCAGCTGCCCACTAGGAGAC--------------------------
1_504     CCCC-AGAAAGTCAGTATGGATATCCAGGAATGCCCCCAGCACCACAGGGCAG----GGCGCCATACCCTCAGCCGCCCACTAGGAGAC--------------------------
1_555     CCCC-AGAAAGTCAGTATGGATATCTAGGAATGCCCCCAGCACCACAGGGCAG----GGAGCCATACCCTCAGCCGCCCACTAGGAGAC--------------------------
20_078    CCCCCAGAAAATCAGTATGGACAGCCAGGAACGTTTCCAGCACCACAGGGCACGCACGGCGATATAGCCTCAGCTGCCCACTATGAGAC--------------------------
21_019    CCCC-AGAAAGTCAGTATGGATATCCAAGAATGCCCCCAGCACCACAGGGCAG----GGCGCCATACCCTCAGCCGCCCACTAGGAGAC--------------------------
22_012    CCCC-AGAAAGTCAGTATGGATATCCAGGAATGCCCCCAGCACCACAGGGCAG----GGCGCCATACCCTCAGCCGCCCACTAGGAGAC--------------------------
22_041    CTTC-AGAGGTTCAATACAGACCTCAAGCGGTGTGTCCTGTGCCAAATAGCAC----GGCACCATACCAGCAACCCACAGCGATGGCGTCTAATTCACCAGCAACACAGGACGCG
2_Tan     CCCC-AGAAAGTCAGTATGGATATCCAGGAATGCCCCCAGCACCACAGGGCAG----GGCGCCATACCCTCAGCCGCCCACTAGGAGAC--------------------------
3_014     CCCC-AGAAAGTCAGTATGGACATCCAGGAATGCTCCCAGCACCACAGGGCAG----GGCGCCATACCCTCAGCCGCCCACTATGAGAC--------------------------
3_209     CTCC-AGAGGTTCAGTATGGATCTCAGGTGGTGCGTCCTGTGCTAAATAGCAA----GACACTATATCAACAACCCACGGCGATGGCGT--------------------------
3_294     CCCC-AGAAAGTCAGTATGGATATCCAGGAATGCCCCCAGCACTACAGGGCAG----GGCGCCATATCCTCAGCCGCCCACTGTGAGAC--------------------------
3_354     CCCC-AGAAAGTCAGTATGGATATCCAGGAATGCCCCCAGCACCACAGGGCAG----GGCGCCATACCCTCAGCCACCCACTAGGAGAC--------------------------
3_385     CCCC-AGAAAGTCAGTATGGATATCCAGGAATGCCCCCAGCACCACAGGGCAG----GGCGCCATACCCTCAGCTGCCCACTAGGAGAC--------------------------
3_496     CCCC-AGAAAGTCAGTATGGATATCCAGGAATGCCCCCAGCACCACAGGGCAG----GGCGCCATACCCTCAGCCGCCCACTAGGAGAC--------------------------
3_610     CCCC-AGAAAGTCAGTATGGATATCCAGGAATGCCCCCAGCACCACAGGGCAG----GGCGCCATACCCTCAGCCGCCCACTAGGAGAC--------------------------
4_008     -------------------------------------------------------------------CAGCAACCCACGGCGGTGGCGC--------------------------
4_032     CTCC-AGAGGTTCAGTATGGATCTCAGGCGGTGCGTCCTGTGCCAAATAGCAA----GGCACTATATCAACAACCCACGGCGATGGCGT--------------------------
4_052     CTCC-AGAGGTTCAGTATGGATCTCAGGCGGTGCGTCCTGTGCCAAATAGCAA----GGCACTATATCAACAACCCAAGGCGATGGCGT--------------------------
4_068     CTCC-AGAGGTTCAGTATGGATCTCAGGAGGTGCGTCCTGTGCCAAATAGCAA----GGCACTATATCAACAACCCACGGCGATGGCGT--------------------------
4_716     CCCT-AGAAAGTCAGTGTGGATATCCAGGAATGCCCCCAGCACCACAGGGCAG----GGCGCCATACCCTCAGCCGCCCACTAGGAGAC--------------------------
4_742     CCCC-AGAAAGTCAGTATGGACATCCAGGAATGCTGCCAGCACTACAGAATAG----GGTGCCACACCCTCAGCCGCCCACTTTGAGAC--------------------------
5_147     CCCC-AGAAAGTCAGTATGGATATCCAGGAATACCCCCAGCACCACAGGGCAG----GGTGCCATACCCTCAGCCGCCCACTAGGAGAC--------------------------
5_259     CCCC-AGAAAGTCAGTATGGACATTCAGGAATGCTCCCAGCACCACAGGGCAG----GGAGCCATACCCTCAGCCACCCACTATGACAC--------------------------
5_535     TTCC-AGAGGTTCAGTATAGATCTCAGGCGGGGTGTCCTGTCCCAAATAGCAG----GGCACTATATCAGCAACCCACAGCGATGGCGT--------------------------
5_544     CCCC-AGAAAGTCAGTATGGATATCCAGGAATGCCCCCAGCACCACAGGGCAG----GGCGCCATACCATCAGCCGCCCACTAGGAGAC--------------------------
6_098     CCCC-AGAAAATCAGTATGGACACCCAGGAATGTTTCCAGCACCACAGGGCAG----GGCTCTAGATCCTCAGCCGTCCACTGTGAGAC--------------------------
6_178     CTCC-AGAGGTTAAATACAGACCTCAAGTGGTGTGTCCTGTGCCAAATAGCAC----GGCACCATACCAGCAACCCACGGCAGTGGTGT--------------------------
6_240     -------------------------------------------------------------------------------------------------------------------
6_324     CCCC-AGAAAGTCAGTATGGATATCCAGGAATGCCCCCAGCACCACAGGGCAG----GGCGCCATACCCTCAGCCGCCCACTAGGAGAC--------------------------
7_345     CCCC-AGAAAGTTAGTATGGATATCCAGGAATGCCCCCAGCACCACAGGGCAG----GGCGCCATACCCTCAGCCGCCCACTAGGAGAC--------------------------
7_450     CCCC-GGAAAATCAGTATGGACAGCCAGGAATGTTCCCAGCCCCACAGGACAG----GGCGCCATACCCTCAGCCACCCACTATGAGAC--------------------------
8_047     CTCC-AGAGGTTCAGTATGGATCTCAGGCGGTGCGTCCTGTGCCAAATAGCAA----GGCAGTATATCAACAACCCACGGCGATGGCGT--------------------------
8_074     CTCC-AGAGGTTCAGTATGGATCTCAGGCGGTGCGTCCTGTGCCAAATAGCAA----GGCACTATATCAA---CCCACGGCGATGGCGT--------------------------
8_094     CTCC-AGAGGTTCAGTATGGATCTCAGGCGGTGCGTCCTGTGGCAAATAGCAA----GGCACTATATCAACAACCCACGGCGATGGCGT--------------------------
8_121     -------------------------------------------------------------------------------------------------------------------
8_235     -------------------------------------------------------------------------------------------------------------------
8_525     CCCC-AGAAAGTCAGTATGGATATCCAGGAATGCCCCCAGCACCACAGGGCAG----GGCGCCATACCCTCAGCCGCCCACTAGGAGAC--------------------------
8_559     CTCC-AGAGGTGCAGTATAGATCCCAGATGGGA--TCCTGTGCCAAATAGTAG----GGCACTATATCAACAACCCACGGCGATGGTGT--------------------------
9_307     -------------------------------------------------------------------------------------------------------------------
9_340     CCCC-AGAGTATCAGTATGGATGGCCAGGA-TGTTTCCAGCACCGCAGGGCAG----GGCGCTATAGCCGCAGCCGCCCACTATGAGAC--------------------------
HML2_Rep  CCCC-AGAAAGTCAGTATGGATATCCAGGAATGCCCCCAGCACCACAGGGCAG----GGCGCCATACCCTCAGCCGCCCACTAGGAGAC--------------------------
K113      CCCC-AGAAAGTCAGTATGGATATCCAGGAATGCCCCCAGCACCACAGGGCAG----GGCGCCATACCCTCAGCCGCCCACTAGGAGAC--------------------------
K115      CCCC-AGAAAGTCAGTATGGATATCCAGGAATGCCCCCAGCACCACAGGGCAG----GGAGCCATACCCTCAGCCGCCCACTAGGAGAC--------------------------
X_540     -------------------------------------------------------------------------------------------------------------------
Y_026     CTCC-AGAGGTTCAGTATAGAACTCAGGTGGTGCGCCCAAC-CCAAATAGCAG----GGCACTATATCAACATCCCACGGCAATGGTGT--------------------------
93        CCCC-GGAAAATCAGTATGGACAGCCAGGAATGTTCCCAGCCCCACAGGACAG----GGCGCCATACCCTCAGCCACCCACTATGAGAC--------------------------
```

```
           350  355  360  365  370  375  380  385  390  395  400  405  410  415  420  425  430  435  440  445  450  455  460
10_019    -------------------------------TTAATCATACGGCACCACCTAGTAGACAGGGTAGTGAATTACATGAAATTATTGATAAG----------TCAAGAAAGGAAGGA
10_310    -------------------------------TTAATCCTACGGCACCACCTAGTAGACAGGGTAGTGAATTACATGAAATTATTGATAAA----------TCAAGAAAGGAAGGA
11_233    --------------------------------------------------------------------------------------------------------GGCCATTTGGG
11_277    -------------------------------TTTATCCTACAGCACCACCTAGTGGACAAGGTAGTGCATTACATAAAATTATTGATAAG----------GCAAGGAAACAAGGA
11_424    -------------------------------TTAATCCTACGGCACCACCTAGTAGACAGGGTAGTGAATTACATGAAATTATTGATAAA----------TCAAGAAAGGAAGGA
11_456    -------------------------------TTAATCCTACGGCACCGCCTAGTAGACAGGGTAGTGAATTACATGAAATTATTGAGA------------------AGAAAGGAAGGA
12_216    -------------------------------TTAATCCTACGGCACCACCTAGTAGACAGGGTAGTGAATTACATGAAATTATTGATAAA----------TCAAGAAAGGAAGGA
12_419    --------------------------------------------------------------------------------------------------------GGCTGTT-GGG
14_023    -------------------------------TTGATCCTACACTACCACCTAGTGGACAAGATAGTGCACTGCAAAAAATCATTGATAAA----------GCCAGAAAACAGGGA
16_010    GCGCTGTGTCCTCAGCCGCCCACTATGAGACTTAATCCAACAGCACCACCTAGTGGACAAGGTAGCACACTGCACGCGATCATTGATGAA----------GCTAGAAAACAGGGA
17_029    -------------------------------------------------------------------------------------------------------------------
19_157    -------------------------------TTAATCCTACATCACCACCTAGTAGACAGGGTGGTGCATTACATAAAATTATT---AAA----------GCAAGAAAACAAGGA
19_238    -------------------------------TTAATCCTATGGTACCACCTAGTAGACAGGGTAGTGAATTACCAGAAATTATTGATAAG----------TCAAGAAAGGAAGGA
19_352    -------------------------------TTAATCCTACGGCACCACCTAGTAGACGGGGTAGTGAATTACATGAAATTATTGATAAA----------TCAAGAAAGGAAGGA
19_407    -------------------------------TTAATCCTACACCACCACCTAGTGGACAGGGTAGTGCATTACATAAAATTATTGATAAG----------GCAAGAAAACAAGGA
19_639    --------------------------------------------------------------------------------------------------------GGTCATA-GGG
1_059     -------------------------------TTAATCCTACATCACCACCTAGTGGACAAGGTAGTACACTGCATGAAATCATTGATAAA----------GCCAGAAAACAGGGA
1_061     -------------------------------TTAGTCCTACATCACCACCTAGTGGACAAGGTAGTACACTGCATGAAATTATTGATAAA----------GCCAGAAAACAGGGA
1_259     -------------------------------TTAATCCTATGGCACCACCTAGTAGACAGGGTAGTGAATTACATGAAATTATTGATAAA----------TCAAGAAAGGAAGGA
1_504     -------------------------------TTAATCCTACGGCACCACCTAGTAGACAGGGTAGTGAATTACATGAAATTATTGATAAA----------TCAAGAAAGGAAGGA
1_555     ---------------------------------AATCCTATGGCACCACCTAGTAGACAGGGTAGTGAATTACATGAAATTATTGAGAAG----------TCAAGAAAGGAAGGA
20_078    -------------------------------TTAATCCTACACCACCACCTAGTGGACAGGATGGTGCATTACATGAAATTATTGAGAAG----------GCAAGAAAACAAGGA
21_019    -------------------------------TTAATCCTACGGCACCACCTAGTAGACAAGGTAGTGAATTACATGAAATTATTGATAAA----------TCAAGAAAGGAAGGA
22_012    -------------------------------TTAATCCTACGGCACCACCTAGTAGACAGGGTAGTGAATTACATGAAATTATTGATAAA----------TCAAGAAAGGAAGGA
22_041    GCGCTGTATCCTCAGCCGCCCACTGTGAGACTTAATCCTACAGCATCACGTAGTGGACAGGGTGGTGCACTGCATGCAGTCATTGATGAA----------GCCAGAAAACAGGGC
2_Tan     -------------------------------TTAATCCTACGGCACCACCTAGTAGACAGGGTAGTAAATTACATGAAATTATTGATAAA----------TCAAGAAAGGAAGGA
3_014     -------------------------------TTAATCCTACAGCACCACCTAATAGACAGGGTAGTGCATTACATAAAATTATTGATAAG----------TCAAGAAAGCAAGGA
3_209     -------------------------------TTGATCCTACAGCACCACCTAGTGGACAAGATGGTGCACTGCATGAGACCATTGCTACA----------GCCAGAAAACAGGGA
3_294     -------------------------------TTAATCCTACAGCATCACGTAGTGGACAAGGTGGTACACTGCACGCAGTCATTGATGAA----------GCCAGAAAACAGGGA
3_354     -------------------------------TTAATCCTACGGCACCACCTAGTAGACGGGGTAGTGAATTACATGAAATTATTGATAAA----------TCAAGAAAGGAAGGA
3_385     -------------------------------TTAATCCTATGGCACCACCTAGTAGACAGGGTAGTGAATTACATGAAATTATTGATAAA----------TCAAGAAAGGAAGGA
3_496     -------------------------------TTAATCCTACGGCACCACCTAGTAGACAGGGTAGTGAATTACATGAAATTATTGATAAA----------TCAAGAAAGGAAGGA
3_610     -------------------------------TTAATCCTACGGCACCACCTAGTAGACAGGGTAGTGAATTACATGAAATTATTGATAAA----------TCAAGAAAGGAAGGA
4_008     -------------------------------TTAATCCTACAGCACCACCTAGTGGACAATGTAGCACACTGTATGAAATCATTGATTAA----------GCCAGAAAACAGGGA
4_032     -------------------------------TTGATCCTACAGTACCACCTAGTGGACAAGATAGTGCACTGCATGAGACCATTGCTACA----------GCCAGAAAACAGGGA
4_052     -------------------------------TTGATCTTACAGTACCACCTAGTGGACAAGGTAGTGCACTGCATGAGACCATTGCTACA----------GCCAGAAAACAGGGA
4_068     -------------------------------TTGATCTTACAGTACCACCTAGTGGACAAGATAGTGCACTGCATGAGACCATTGCTACA----------GCCAGAAAACAGGGA
4_716     -------------------------------TTAATCCTACGGCACCACCTAGTAGACAGGGTAGTGAATTACATGAAATTATTGATAAA----------TCAAGAAAGGAAGGA
4_742     -------------------------------TTAATCCTATGGCACCACCTAGTAGACAGGGTAGTGCATTACATAAAATTATTGTCAAGAAAATTATAGTCAAGAAAGCAAGGA
5_147     -------------------------------TTAATCCTATGGCACCACCTAGTAGACAGGGTAGTGAATTACATGAAATTATTGATAAA----------TCAAGAAAGGAAGGA
5_259     -------------------------------TTAATCCAACAGCACCACCTAGTCAACAGGGTAGTGCGTTACATAAAATTATTGATAAT----------TCAAGAAACCAAGGA
5_535     -------------------------------TTGATCCTACAGCACCAGCTAGTGTACAAGATAGTACACTGCATAAAACCATT-----A----------GCCAGAAAACAGGGA
5_544     -------------------------------TTAATCCTATGGCACCACCTAGTAGACAGGGTAGTGAATTACATGAAATTATTGATAAA----------TCAAGAAAGGAAGGA
6_098     -------------------------------TTAATCCTACAGCACTACCTAGTGGACAGCATAGTGCATTACATAAAATTATTGATAAG----------GCAAGAAAACAAGGA
6_178     -------------------------------TTAATCCTACCGCACCACCTAGTGGACAAGGTAGTACACTGCATGAAATTATTGATAAA----------GCCAGTAAACAGGGA
6_240     --------------------------------------------------------------------------------------------------------GGCCATT-GGG
6_324     -------------------------------TTAATCCTACGGCACCACCTAGTAGACAGGGTAGTGAATTACATGAAATTATTGATAAA----------TCAAGAAAGGAAGGA
7_345     -------------------------------TTAATCCTACGGCACCACCTAGTAGACAGGGTAGTGAATTACATGAAATTATTGATAAA----------TCAAGAAAGGAAGGA
7_450     -------------------------------TTAATCCTACAGCACCACCTAGTGAGCAGGGTAGTGTATTACATAAAATTATTGATGAG----------GCAAGAAAACAAAGA
8_047     -------------------------------TTGATCTTACGGTACCACCTAGTGGACAAGATAGTGCACTGCATGAGACCATTGCTACA----------GCCAGAAAACAGGGA
8_074     -------------------------------TTGATCTTACGGTACCACCTAGTGGACAAGATAGTGCACTGCATGAGACCATTGCTACA----------GCCAGAAAACAGGGA
8_094     -------------------------------TTGATCTTACGGTACCACCTAGTGGACAAGATAGTGCACTGCATGAGACCATTGCTACA----------GCCAGAAAACAGGGA
8_121     -------------------------------------------------------------------------------------------------------------------
8_235     --------------------------------------------------------------------------------------------------------GGCCTTT-GGG
8_525     -------------------------------TTAATCCTACGGCACCACCTAGTAGACAGGGTAGTGAATTACATGAAATTATTGATAAA----------TCAAGAAAGGAAGGA
8_559     -------------------------------TTGATCCTACACTACCAGCTAGTGGACAACATAGTGCACTGCATGAGACCGTTGATACA----------GCCAGAAAACAGGGA
9_307     --------------------------------------------------------------------------------------------------------GGCCATT-GGG
9_340     -------------------------------GTAATCCTACAGCACCACCTAGTGGACAGGGTCGTGCATTACATAAAATTATTGAAAAG----------ACA---AAACAAGGG
HML2_Rep  -------------------------------TTAATCCTACGGCACCACCTAGTAGACAGGGTAGTAAATTACATGAAATTATTGATAAA----------TCAAGAAAGGAAGGA
K113      -------------------------------TTAATCCTACGGCACCACCTAGTAGACAGGGTAGTGAATTACATGAAATTATTGATAAA----------TCAAGAAAGGAAGGA
K115      -------------------------------TTAATCCTACGGCACCACCTAGTAGACAGGGTAGTGAATTACATGAAATTATTGATAAA----------TCAAGAAAGGAAGGA
X_540     --------------------------------------------------------------------------------------------------------GGCCGTC-AGG
Y_026     -------------------------------TTGATCCTACAGTACCACCTAGTGGACAACATAGTGCACTCCATGAAATCATTGATAAA----------GCCAGAAAACAGGGA
93        -------------------------------TTAATCCTACAGCACCACCTAGTGAGCAGGGTAGTGTATTACATAAAATTATTGATGAG----------GCAAGAAAACAAAGA
```

```
           4    4    4    4    4    4    4    5    5    5    5    5    5    5    5    5    5    5    5    5    5    5    5
           6    7    7    8    8    9    9    0    0    1    1    2    2    3    3    4    4    5    5    6    6    7    7
           5    0    5    0    5    0    5    0    5    0    5    0    5    0    5    0    5    0    5    0    5    0    5
10_019    GA--CACTGAGGCGTGGCAATTCCCAGTAACGTTAGAACCGATACCACCTGGAGAAGGA-----------GCCCAAGAGGGAGAGCCTCTCACAGTTGAGGCCAGATACAAGTCT
10_310    GA--TACTGAGGCGTGGCAATTCCCAGTAACGTTAGAACCGATGCCACCTGGAGAAGGA-----------GCCCAAGAGGGAGAGCCTCTCACAGTTGAGGCCAGATACAAGTCT
11_233    GC----TAAAACCAGGATGGCCACC--TCCTCCTCAGCCGAGTGAGTGCTGGAGGAGGG-----------AGCCTGAAACTAGGCTCACTGTGACTCA-TTTGTGGCACCCATTA
11_277    GA--TATTGAGGCGTGGCAATTCCCAGTA---------CCGATGCCACCTGGAGAAGGG-----------GCCCAAGAAGGAGCGCCTACCCTAGTTGGGGCCAGATATGAGTCC
11_424    GA--TACTGAGGCATGGCAATTCCCAGTAACGTTAGAACCGAAGCCACCTGGAGAAGGA-----------GCCCAAGAGGGAGAGCCTCCCACAGTTGAGGCCAGATACAAGTCT
11_456    GA--TACTGAGGCGTGGCAATTCCCAGTAACGTTAGAACCGATGCCACCTGGAGAAGAA-----------GCCCAAGAGGGAGAGCCTCTCACAGTTGAGGCCAGATACAAGTCT
12_216    GA--TACTGAGGCATGGCAATTCCCAGTAATGTTAGAACCGATGCCACCTGGAGAAGGA-----------GCCCAAGAGGGAGAGCCTCCCACAGTTGAGGCCAGATACAAGTCT
12_419    GT----TAAAGCACGATGGCCACC--TCCTCCTCAGCCGAGTGAGTGCTGGGGGAGGG-----------AGCCTGAAACTACGCTTGCTGCGACTTGGCTCGTGGCACTCATTA
14_023    GA--TCTTGAGGCAGGGCAGTTCCTGGTAATTTTACAACCAATACCGGCTGGAAAACGG-----------AGTCAAGCAGGAGCGTCTGTCCAAACTGAGGCTAGATATGAATCT
16_010    GA--TCTTGAGGCGTGGCAGTTCCTGGTAATTTTACAATCGGTACCGGCCGGGGAAGGG-----------GCTCCAGCAGGAGCACCTGCGGTGGCTAATGCTAGATATGAACGC
17_029    -------------------------------------------------------------------------------------------------------------------
19_157    GA--TATCGAGGCGTGGCAGTTCCCAGTAATATTAGAGCCGATACCACCTGAAGAAGGG-----------GCCCAAGAGGGAGAGCCTCCCCTAGCTGAGGCCAGATATGAGTCC
19_238    GA--TATTGAGGCATGGCAATTCCCAATAATGTTAGA------ACCACCTGGAGAAGGA-----------GCCCAAGAGGGAGAGCCTCTCACAGTTGAGGCCAGATACAAGTCT
19_352    GA--TACTGAGGCATGGCAATTCCCAGTAACGTTAGAACCGATGCCACCTGGAGAAGGA-----------GCCCAAGAGGGAGAGCCTCCCACAGTTGAGGCCAGATACAAGTCT
19_407    GA--TACCGAGGCATGGCAATTCCCAGTAATATTAGAACCGATACCACCTGAAGAAGAG-----------GCCCAAGAGGGAGCGCCTCCCCTAGCTGAGGCCAGATATGAGTCC
19_639    GC----TAAAACCACGATGGCCACC--TCCTCCTCAGCCAAGTGAGTGCTGGGGGAGGG-----------AGCCTGAAACTAGGCTCACTGCAACTTGGCTCGCTGTGCCCATTA
1_059     GA--TCGTGAGGCATGGCAGTTCCTTGTAATTTTACAACCAATACCAGCTGGGAAAGGG-----------GGGCAAGCTGGAGCGTCTGTCCAAACTGAGGCTAGATATGAATCT
1_061     GA--TCTTGAGGCATGGCAGTTCCTTGTAATTTTACAACCAATACCAGCTGGGAAAGGG-----------GGGCAAGCTGGAGCGTCTGTCCAAACTGAGGCTAGATATGAATCT
1_259     GA--TACTGAGGCATGGCAATTCCCAGTAACGTTAGAACCAATGCCACCTGGAGAAGGA-----------GCCCAAGAGGGAGAGCCTCCCACAGTTGAGGCCAGATACAAGTCT
1_504     GA--TACTGAGGCATGGCAATTCCCAGTAACGTTAGAACCGATGCCACCTGGAGAAGGA-----------GCCCAAGAGGGAGAGCCTCCCACAGTTGAGGCCAGATACAAGTCT
1_555     GA--TACTGAGGCGTGGCAATTCCCAGTAACGTTAGAACCGATGCCACCTGGAGAAGGA-----------GCCCAAGAGGGAGAGCCTCTCACAGTTGAGGCCAGATACAAGTCT
20_078    GAGATACTGAGGCGTTTTAATTTCAAGTAATATTAGAGCCCATACTACCTGGAGAAGGG-----------CCC--------------TCCCCAAGCTGATGCCAGATATGAGTCC
21_019    GA--TACTGAGGCATGGCAATTCCCAGTAATGTTAGAACCGATGCCACCTGGAGAAGGA-----------GCCCAAGAGGGAGAGCCTCTCACAGTTGAGGCCAGATACAAGTCT
22_012    GA--TACTGAGGCATGGCAATTCCCAGTAACGTTAGAACCGATGCCACCTGGAGAAGGA-----------GCCCAAGAGGGAGAGCCTCCCACAGTTGAGGCCAGATACAAGTCT
22_041    GA--TCTTGAGGCATGGCGGTTCCTGGTAATTTTACAACTGGTACAGGCCGGGGAAGAG-----------ACTCAAGTAGGAGCGCCTGCCCGAGCTGAGACTAGATGTGAACCT
2_Tan     GA--TACTGAGGCATGGCAATTCCCAGTAACGTTAGAACCGATGCCACCTGGAGAAGGA-----------GCCCAAGAGGGAGAGCCTCCCACAGTTGAGGCCAGATACAAGTCT
3_014     GA--TATTGAGGCGTGGCCATTCCCACTAATGTTAGAACTGATACCACCTGGAGAAGGA-----------GCCCAAGAGGGAGAGCCTCCCACAGTTGAGGCCAGATACAAGTCT
3_209     GA--TCTTGAGGCATGGCAGTATCCGGTAATTTTACAACCCATACCGGCCAGAAAAGGG-----------AGTCAAACAGGAGCATCTGTCTGAACTGAGGCTAGATATGAATCT
3_294     GA--TCTTGAGGCATGGCGGTTCCTGGTAATTTTACAACTGGTACAGGCCGGGGAAGAG-----------ACTCAAGTAGGAGCGCCTGCCCGAGCTGAGACTAGATGTGAACCT
3_354     GA--TACTGAGGCATGGCAATTCCCAGTAACGTTAGAACCGATGCCACCTGGAGAAGGA-----------GCCCAAGAGGGAGAGCCTCCCACAGTTGAGGCCAGATACAAGTCT
3_385     GA--TACTGAGGCGTGGCAATTCCCAGTAACATTAGAACCGATGCCAACTGGAGAAGGA-----------GCCCAAGAGGGAGAGCCTCCCACAGTTGAGGCCAGATACAAGTCT
3_496     GA--TACTGAGGCATGGCAATTCCCAGTAACGTTAGAACCGATGCCACCTGGAGAAGGA-----------GCCCAAGAGGGAGAGCCTCCCACAGTTGAGGCCAGATACAAGTCT
3_610     GA--TACTGAGGCATGGCAATTCCCAGTAACGTTAGAACCGATGCCACCTGGAGAAGGA-----------GCCCAAGAGGGAGAGCCTCTCACAGTTGAGGCCAGATACAAGTCT
4_008     GA--TCTTGAGGCATGGCAGTTCCCAGTAATTTTACAACCGGTACCAGCCGGGGAAGAG-------------ACAAGTAGGAGTGCCTACCCAAGCTGAGGCTAGATATGAATCT
4_032     GA--TCTTGAGGCATGGCAATATCCGGGAATGTTACAACCGATGCCGGCCGGGAAAGGG-----------AGTCAAGCAGGAGCGTCTGTCTGAACTGAGACCAGATATGAATCT
4_052     GA--TCTTGAGGCATGGCAATATCCGGGAATGTTACAAACAATGCCGGCCGGGAAAGGG-----------AGTCAAGCAAGAGCGTCTGTCTGAACCAAGACTAGATATGAATCT
4_068     GA--TCTTGAGGCATGGCAATATCTGGTAATGTTACAACCGATGCCGGCCGGGAAAGGG-----------AGTCAAGCAGGAGCGTCTGTCTGAACTGAGACCAGATATGAATCT
4_716     GA--TACTGAGGCATGGCAATTCCCAGTAACGTTAGAACCGATGCCACCTGGAGAAGGA-----------GCCCAAGAGGGAGAGCCTCCCACAGTTGAGGCCAGATACAAGTCT
4_742     GA--TACTGAGGTGTGGCAATTCCCAGTAATGTTGGAACTGATGCCACCTGGAGAAGGG-----------GCCCAAGAGGGAGAGCCTCCCACAGTTGAGGCCAGATACAAGTCT
5_147     GA--TACTGAGGCATGGCAATTCCCAGTAACGTTAGAACCGATGCCACCTGGAGAAGGA-----------GCCCAAGAGGGAGAGCCTCTCACAGTTGAGGCCAGATACAAGTCT
5_259     GA--TATTGAGGCATGGCAATTCCCAGTAACGTTAGAACCAATACCACCTGGAGAAGGG-----------GCCCAAGAGGGAGAGCCTCAAACAGTTGAGGCCAGATATGAGTCT
5_535     GA--TCTTGAGGCATGGCAGTACCCGGTAATTTTACAACCGATACTGGCTGGGAAAGGG-----------AGTCAAGCAGGAGCCTCTGTCCAAACTGAGATTAGATATGAATCT
5_544     GA--TACTGAGGCATGGCAATTCCCAGTAACGTTAGAACCGATGCCACCTGGAGAAGGA-----------GCCCAAGAGGGAGAGCCTCCCACAGTTGAGGCCAGATACAAGTCT
6_098     GA--TACTGAGGCGTGACAATTCCCAGTAACATTAGAACTGATACCACCTGGAGAAGGG-----------GCCCAAGAGGGAGTGCCTCCCCTAGCTGAGGCCAGATATGAGTCC
6_178     GA--TCTTGAGGCATGGGAGTTCCCGGTAATTTTACAACCGATACCGGCCGGGAAAGGG-----------ACTCTAGCAGGAGCGTCTGTCCAAACTGAAGCTAGATATGAATCT
6_240     GC----TAAAACCACAATGGCCACA--TCCTCCTCAGCCAAGTGATTGCTGGGGGAGGG-----------AGCCTGAAACTAGGCTCGCTGTGACTTGGCTCGCAGCACTCATTA
6_324     GA--TACTGAGGCATGGCAATTCCCAGTAACGTTAGAACCGATGCCACCTGGAGAAGGA-----------GCCCAAGAGGGAGAGCCTCCCACAGTTGAGGCCAGATACAAGTCT
7_345     GA--TACTGAGGCATGGCAATTCCCAGTAACGTTAGAACCGATGCCACCTGGAGAAGGA-----------GCCCAAGAGGGAGAGCCTCTCACAGTTGAGGCCAGATACAAGTCT
7_450     GA--TATTGAGGCATGGCAATTCCCAGTAATATTAGAACCAATACCACCTGGAGAAGGG-----------GCCCAAGAGGGAGAGCCTCCCCTAGCTGAGGCCAGATATGAGTCT
8_047     GA--TCTTGAGGCATGGCAATATCCGGTAATGTTACAACAGATGCCGGCCGGGAAAGGC-----------AGTCAAGCA------------------------------------
8_074     GA--TCTTGAGGCATGGCAATATCCGGTAATGTTACAACCGATGCCGGCCGGGAAAGGG-----------AGTCAAGCA------------------------------------
8_094     GA--TCTTGAGGCATGGCAATATCCGGTAATGTTACAACAGATGCCGGCCGGGAAAGGC-----------AGTCAAGCA------------------------------------
8_121     -------------------------------------------------------------------------------------------------------------------
8_235     GC----TAAAACCACGATGGCCACC--TCCTCCTCAGCTGAGTGAGTGCTGGGGGAGGG-----------AACCTGAAACTAGGCTCGCTGTGACTTGGTTCACAGCACCCATTA
8_525     GA--TACTGAGGCATGGCAATTCCCAGTAACGTTAGAACCGATGCCACCTGGAGAAGGA-----------GCCCAAGAGGGAGAGCCTCTCACAGTTGAGGCCAGATACAAGTCT
8_559     GA--TCTTGAGGCATGGCAGTATCCGGTAATGTTACAACCGATACCGGCCGGGAAAGGGCCAGGAAAGGGAGTCAAGCAGGAGCGTCTGTCCGAACGGAGGCTAGGTAAGAATAT
9_307     GC----TACAACCACAATGGCCACC--TCCTCCTCAGCCGAGTGAGTGCTGGGGGAGGC-----------AGCCTGAAACTAGGCACACTGCGACTTGGCTCGTGGCACTCATTA
9_340     GA--TACTGACGCGTGGCAGACCCCGGTAATATTAGAACCCATACCACCTGGAAGAGGG-----------GCCCAAGAGGGAGCGCCTCCCCGAGCTGATGCCAGAAATGGGTCC
HML2_Rep  GA--TACTGAGGCATGGCAATTCCCAGTAACGTTAGAACCGATGCCACCTGGAGAAGGA-----------GCCCAAGAGGGAGAGCCTCCCACAGTTGAGGCCAGATACAAGTCT
K113      GA--TACTGAGGCATGGCAATTCCCAGTAACGTTAGAACTGATGCCACCTGGAGAAGGA-----------GCCCAAGAGGGAGAGCCTCCCACAGTTGAGGCCAGATACAAGTCT
K115      GA--TACTGAGGCATGGCAATTCCCAGTAACGTTAGAACCGATGCCACCTGGAGAAGGA-----------GCCCAAGAGGGAGAGCCTCCCACAGTTGAGGCCAGATACAAGTCT
X_540     GC----TAAAACCACGATGGCCACC--TCCTCCTCAGTCGAGTGAGTGCTGGGGGAGGG-----------AGCCTGAAACCAGGCTGGCTGCAACTCGGCTCGCGGTGCCCATTA
Y_026     GA--TCTTGAGGCATGGCAGTTCCCAGTAATTTTACAACCCAC-CCGGCCGGGAAAGGG-----------AGTCAAGCAGGAGCGTCTGTCCGAACTGAGTGTAGATATGAATCT
93        GA--TATTGAGGCATGGCAATTCCCAGTAATATTAGAACCAATACCACCTGGAGAAGGG-----------GCCCAAGAGGGAGAGCCTCCCCTAGCTGAGGCCAGATATGAGTCT
```

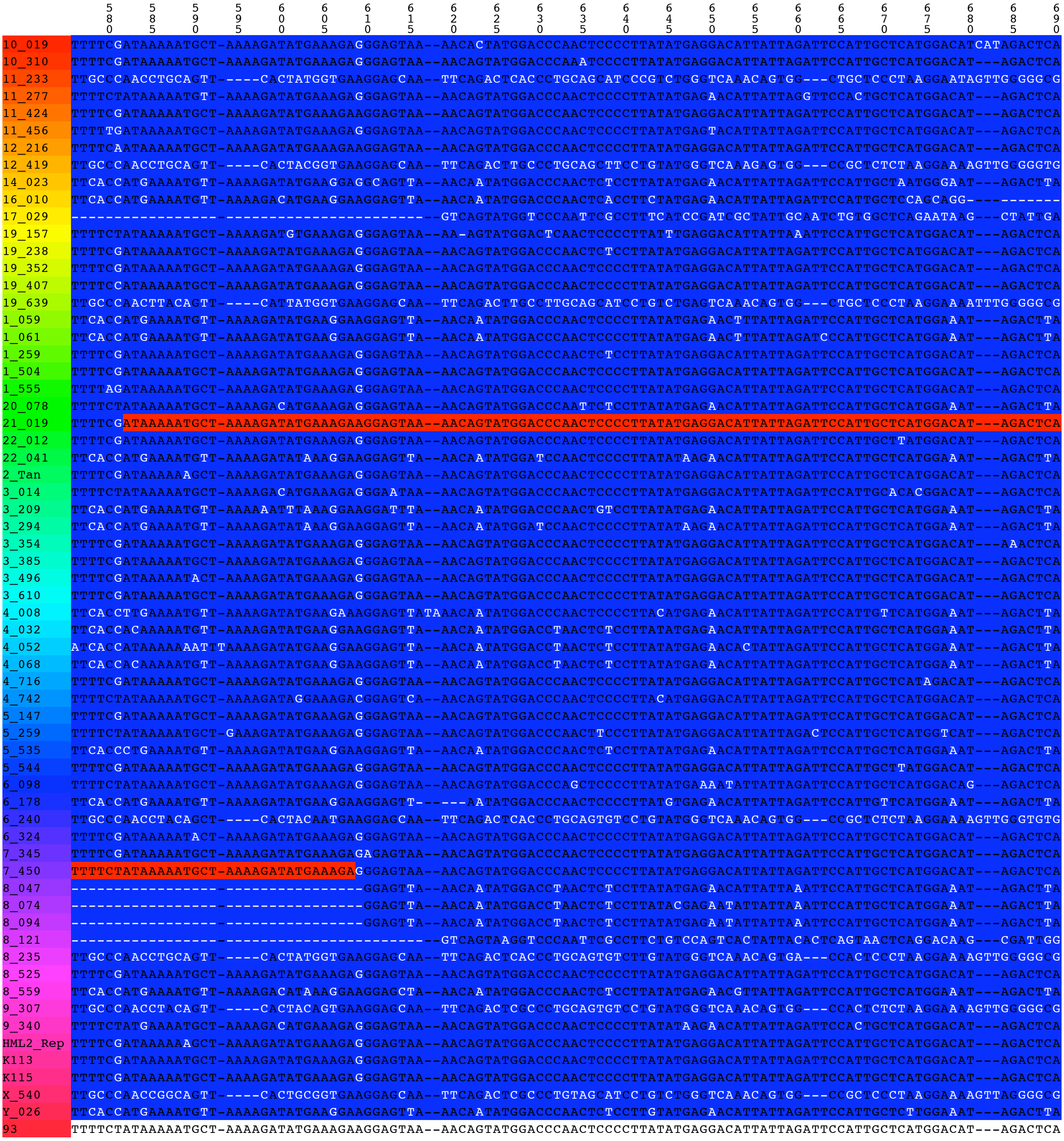
580 585 590 595 600 605 610 615 620 625 630 635 640 645 650 655 660 665 670 675 680 685 690
10_019
10_310
11_233
11_277
11_424
11_456
12_216
12_419
14_023
16_010
17_029
19_157
19_238
19_352
19_407
19_639
1_059
1_061
1_259
1_504
1_555
20_078
21_019
22_012
22_041
2_Tan
3_014
3_209
3_294
3_354
3_385
3_496
3_610
4_008
4_032
4_052
4_068
4_716
4_742
5_147
5_259
5_535
5_544
6_098
6_178
6_240
6_324
7_345
7_450
8_047
8_074
8_094
8_121
8_235
8_525
8_559
9_307
9_340
HML2_Rep
K113
K115
X_540
Y_026
93

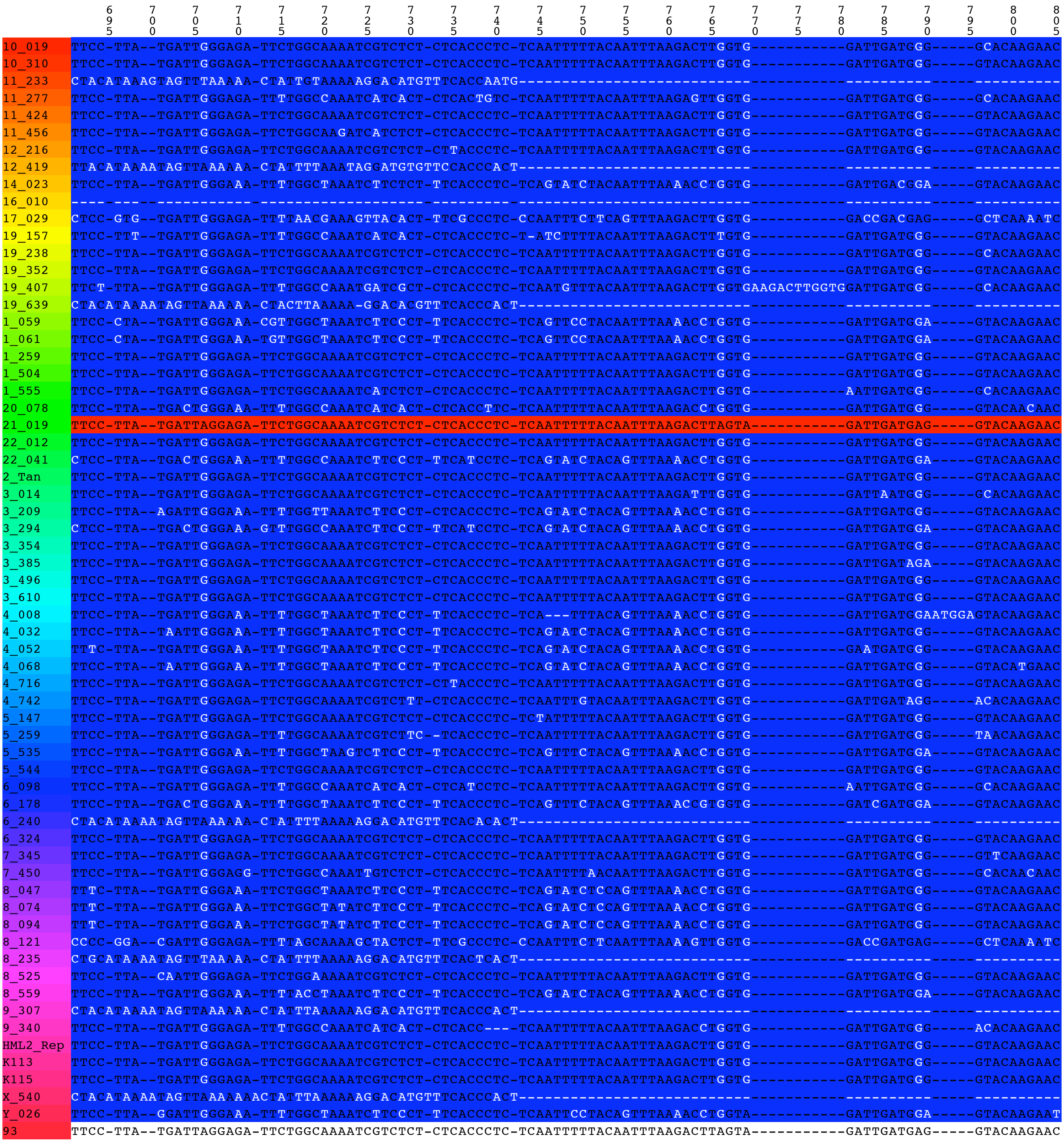
695 700 705 710 715 720 725 730 735 740 745 750 755 760 765 770 775 780 785 790 795 800 805
10_019
10_310
11_233
11_277
11_424
11_456
12_216
12_419
14_023
16_010
17_029
19_157
19_238
19_352
19_407
19_639
1_059
1_061
1_259
1_504
1_555
20_078
21_019
22_012
22_041
2_Tan
3_014
3_209
3_294
3_354
3_385
3_496
3_610
4_008
4_032
4_052
4_068
4_716
4_742
5_147
5_259
5_535
5_544
6_098
6_178
6_240
6_324
7_345
7_450
8_047
8_074
8_094
8_121
8_235
8_525
8_559
9_307
9_340
HML2_Rep
K113
K115
X_540
Y_026
93

10_019	A
10_310	A
11_233	-
11_277	A
11_424	A
11_456	A
12_216	A
12_419	-
14_023	A
16_010	-
17_029	A
19_157	A
19_238	A
19_352	A
19_407	A
19_639	-
1_059	A
1_061	A
1_259	A
1_504	A
1_555	A
20_078	A
21_019	G
22_012	A
22_041	A
2_Tan	A
3_014	A
3_209	A
3_294	A
3_354	A
3_385	A
3_496	A
3_610	A
4_008	A
4_032	A
4_052	A
4_068	A
4_716	A
4_742	A
5_147	A
5_259	A
5_535	A
5_544	A
6_098	A
6_178	A
6_240	-
6_324	A
7_345	A
7_450	A
8_047	A
8_074	A
8_094	A
8_121	A
8_235	-
8_525	A
8_559	A
9_307	-
9_340	A
HML2_Rep	A
K113	A
K115	A
X_540	-
Y_026	A
93	G

```
          1   5    10   15   20   25   30   35   40   45   50   55   60   65   70   75   80   85   90   95   100  105  110  115
10_019    AGGACC---AAGTCCTCTTCCAGTAGGTCAGATGCCCGTAACATTACAACCTCAAACGCAGGTTAG-------------------------------------------------
10_310    AGGGCC---AAGTCCTCTTCCAGCAGGTCAGGTGCCCGTAACACTACAACCTCAAACGCAGGTTAA-------------------------------------------------
11_233    -------------------------------------------------------------------------------------------------------------------
11_277    ATGGCC---AACTCCTCTTCCAGCGGTTCAGATGCCTGTAACATTACAACCTCAAATGCAGGTTAGACAAGTACAAACCCCAAGAGAATATCAAATAGAGAG--------TCTCT
11_424    AGGCAC---AAGTCCTCTTCCAGCAGGTCAGGTGCCCGTAAGATTACAACCTCAAACGCAGGTTAA-------------------------------------------------
11_456    AGGGCC---AAGTCCTCTTCCAGCAGGTCAGGTGCCCGTAACATTACAACCTCAAACGCAGGTTAG-------------------------------------------------
12_216    AGGCAC---AAGTCCTCTTCCAGCAGGTCAGGTGCCCGTAACATTACAACCTCAAACGCAGGTTAA-------------------------------------------------
12_419    -------------------------------------------------------------------------------------------------------------------
14_023    ATGGCCAACAACTCCTTCTCCCACGGTTCAGATGCCTGTGACATTACAACCTCAAA-----------------------------------------------------------
16_010    ATGGC---CAACTCCTCCTCCCGTGGTTCAGATACCTGTAACATTACAACCTCAAATGCAGGTTAGACAAGTACAAACCCCAAGAGAATATCAAATAGAAAAGGATAG--TCTCT
17_029    -------------------------------------------------------------------------------------------------------------------
19_157    ATGGCC---AACTCCTCTTCCAGCAGTTCAGATGCCTGTAACATTACAACCTCAAATGCAGGTTAGACAAGTACAAACCTGAAGAGAATAACAAATAGAGAAAGATAGAGTATCT
19_238    AGGGCC---ATGTCCTCTTCCAGCAGGTCAGATGCCCGTAATATTACAACCTCAAACGCAGTTTAG-------------------------------------------------
19_352    AGGCAC---AAGTCGTCTTCCAGCAGGTCAGGTGCCCGTAACATTACAACCTCAAACGCAGGTTAA-------------------------------------------------
19_407    ATGGCC---AACTCCTCTCCCAGCAGTTCAGATGCCTGTAACATTACAACCTCAAATGCAGGTTAGACAAGTACAAACCCCAAAAGAATATCAAATAGGGAAAGATAGAGTCTCT
19_639    -------------------------------------------------------------------------------------------------------------------
1_059     ATGGCCATCAACTCCTCCTCCAGTGGTTCAGATGCCTGTAACATTACAACCTCAAATGCAGGTTAGACAAGTGCAAACCCCAAGAGAATATCAAGTAGAAAAGGATAGAGTCTCT
1_061     ATGGCCATCAACTCCTCCTCCAGCGGTTCAGATGCCTGTAACATTACAACCTCAAATGCAGGTTAGACAAGTGCAAACCCCAAGAGAATATCATGTAGAAAAGGATAGAGTCTCT
1_259     AGGCAC---AAGTCCTCTTCCAGCAGGTCAGGTGCCCGTAAGATTACAACCTCAAACGCAGGTTAA-------------------------------------------------
1_504     AGGCAC---AAGTCATCTTCCAGCAGGTCAGGTGCCCGTAACATTACAACCTCAAAAGCAGGTTAA-------------------------------------------------
1_555     AGGGCC---AAGTCCTCTTTCAGCAGGTCAGGTGACCGTAACATTACAACCTCAAGCGCAGGTTAG-------------------------------------------------
20_078    ATGGCC---AACTCCTCCTCCCACAGTTCAGATGCCCGTAACATTACAACCTCAAATGCAGGTTAGACAAGTACAAACCCCAAGAGAATATCAGATAGAAAAGGATAG--TCTCT
21_019    AAGGCC---AAGTCCTCTTCCAGCAAGTCAGGTGCCTGTAACATTACAACCTCAAACGCAGGTTAA-------------------------------------------------
22_012    AGGGAC---AAGTCCTCTTCCAGCAGGTCAGGTGCCCGTAACATTACAACCTCAAAAGCAGGTTAA-------------------------------------------------
22_041    ATCGCCATCAACTCCTCCTCCCGCGGTTCAGATGCCTGTAACATTACAACCTCAAACGCAGGTTAGACAAGCACAAACCCCAAGAGAAAATCAAGTAGAAAGGGACAGAGTCTCT
2_Tan     AGGCAC---AAGTCCTCTTCCAGCAGGTCAGGTGCCTGTAACATTACAACCTCAAAAGCAGGTTAA-------------------------------------------------
3_014     AGGGAC---AAGTCCTCTTCCAGCAGGTCAGATGCCCGTAACATTACAACCTCAAACACAGGTTAG-------------------------------------------------
3_209     ATGGCCACCATCTCCTTCTCCCGCGGTTCAGATACCTGTGACATCACAATCTCAAA-----------------------------------------------------------
3_294     AGGGCC---AAGTCCTCTTCCAGCAGGTCAGGTGCCCGTAACATTACAACCTCAAACGCAGGTTAA-------------------------------------------------
3_354     AGGCAC---AAGTCATCTTCCAGCAGGTCAGGTGCCCGTAACATTACAACCTCAAAAGCAGGTTAA-------------------------------------------------
3_385     AGGCAC---AAGTCCTCTTCCAGCAGGTCAGGTGCCTGTAACATTACAACCTCAAATGCAGGTTAA-------------------------------------------------
3_496     AGGGCC---AAGTCCTCTTCCAGCAGGTCAGGTGCCCGTAACATTACAACCTCAAAAGCAGATTAA-------------------------------------------------
3_610     AGGCAC---AAGTCCTCTTCCAGCAGGTCAGGTGCCTGTAACATTACAACCTCAAACGCAGGTTAA-------------------------------------------------
4_008     -------------------------------------------------------------------------------------------------------------------
4_032     ATGGCCACCAACCCCTTCTCCCGCGGTTCAGATGCCTGTGATGTCACAATCTCAAA-----------------------------------------------------------
4_052     ATGGCCACCAACTCCTTCTCCCGCGGTTCAGATGCCTTTGATGTCACAATCTCAAA-----------------------------------------------------------
4_068     ATGGCCACCAACCCCTTCTCCCGCGGTTCAGATGCCTGTGATGTCACAATCTCAAA-----------------------------------------------------------
4_716     AGGGCC---AAGTCCTCTTCCAGCAGGTCAGGTGCCCGTAACATTACAACCTCAAAAGCAGGTTAA-------------------------------------------------
4_742     AGAGCC---AAGTCCTTTTCTAGCAGGTCAGATGCCCATAACATTACAACCTCAAACGCAGGTTAG-------------------------------------------------
5_147     AGGCAC---AAGTCCTCTTCCAGCAGGTCAGGTGCCTGTAACATTACAACCTCAAACGCAGGTTAA-------------------------------------------------
5_259     AGCACC---AAGCCCTCTTCCAGCAGGTGAGATGCCTGGAACATTACAGTCTCCAATGCAGGTTAG-------------------------------------------------
5_535     ACGGCCATCAACTCCTCCTCCTGCGGTTCAGATGCCTGTGACATTACAACCTCAAATGCAGGTTAGACAAGTACAAACTGCAAGAGAATATCAAGTAGAAAAGGATAGAGTCTCT
5_544     AGGCAC---AAGTCCTCTTCCAGCAGGTCAGGTGCCCGTAAGATTACAACCTCAAAAGCAGGTTAA-------------------------------------------------
6_098     ATGGCC---AACTCCTCTTCCAGCAGTTCAGATGCCTGTAACATTGCAACCTCAAATGCAGGTTAGACAAGTACAAACCCCAGAAGAATATCAAATAGAAAAAGATAAAGTCTCT
6_178     ATGGCCATCAA---CTCCTCCCGTGGTTCAGATACCTGTAACATTACAACCTCAAAGGGAGGTTAGACAAGTACAAACCCCAAGAGAATATCAAGTAGAAAAGGATAGAGTCTCT
6_240     -------------------------------------------------------------------------------------------------------------------
6_324     AGGCAC---AAGTCCTCTTCCAGCAGGTCAGGTGCCTGTAACATTACAACCTCAAAAGCAGGTTAA-------------------------------------------------
7_345     AGGCAC---AAGTCCTCTTCCAGCAGGTCAGGTGCCTGTAACATTACAACCTCAAACGCAGGTTAA-------------------------------------------------
7_450     AGGGCC---AACTCCTCTTCCAGCAGTTCAGATGCCTGTAACATTACAACCTCAAATGCAGGTTAGGCAAGTACAAACCCCAAGAGAATATCAAATAGAGAAAGATAGAGTCTCT
8_047     ATGGCCACCAACTCCTTCTCCCGCGGTTCAGATGCCTGTGATGTCACAATCTCAAA-----------------------------------------------------------
8_074     ATGGCCACCAACTCCTTCTCCCGCGGTTCAGATGCCTGTGATGTCACAATCTCAAA-----------------------------------------------------------
8_094     ATGGCCACCAACTCCTTCTCCCGCGGTTCAGATGCCTGTGATGTCACAATCTCAAA-----------------------------------------------------------
8_121     -------------------------------------------------------------------------------------------------------------------
8_235     -------------------------------------------------------------------------------------------------------------------
8_525     AGGCAC---AAGTCCTCTTCCAGCAGGTCAGGTGCCCGTAACATTACAACCTCAAAAGCAGGTTAA-------------------------------------------------
8_559     ATGGCCACCAACTCCTTCTCCTGTGGTTCAGATGCCTGTGACATTACAACCTCAAA-----------------------------------------------------------
9_307     -------------------------------------------------------------------------------------------------------------------
9_340     ATGGCCATCAACTCCTCCTCCCGCGGTTCAGATGCCTGTAACATTACAACCTCAAATGCAGGTTAGACAAGTACAAACCCCAAGAGAATATCAAATAGAAAAGGAAAGAGTCTCT
HML2_Rep  AGGCAC---AAGTCCTCTTCCAGCAGGTCAGGTGCCTGTAACATTACAACCTCAAAAGCAGGTTAA-------------------------------------------------
K113      AGGCAC---AAGTCCTCTTCCAGCAGGTCAGGTGCCCGTAACATTACAACCTCAAAAGCAGGTTAA-------------------------------------------------
K115      AGGCAC---AAGTCCTCTTCCAGCAGGTCAGGTGCCCGTAACATTACAACCTCAAAAGCAGGTTAA-------------------------------------------------
X_540     -------------------------------------------------------------------------------------------------------------------
Y_026     ATGGCCACCAACTCCTTCTCCCGCGGTTCAGATGCCTGTGACATTACAACCTCAAA-----------------------------------------------------------
94        AGGGCC---AAGTCCTCTTCCAGCAGGTCAGGTGCCCGTAACATTACAACCTCAAACGCAGGTTAG-------------------------------------------------
```

```
          120  125  130  135  140  145  150  155  160  165  170  175  180  185  190  195  200  205  210  215  220  225  230
10_019   -----------------------------------------------AGAAAATAAGACCCAACTACCAGTAGCTTATCAATACTGGCCACCGGCCAAACTTCAGTATCGGCCAC
10_310   -----------------------------------------------AGAAAATAAGACCCAACCGCCAGTAGCTTATCAATACTGGCCGCCAGCCGAACTTCAGTATCGGCCAC
11_233   -------------------------------------------------------------------------------------------------------------------
11_277   GTCATGGCAACGCCAATCCAAATACAGTATCCACAATATCAGCCGGTAGAAAATAAGACCCAACCGCCAGTAGCCTATCAATACTGGCCGCCAGCCGAACTTCAGTATCGGCCGC
11_424   -----------------------------------------------AGAAAATAAGACCCAACCGCCAGTAGCTTATCAATACTGGCCGCCGGCTGAACTTCAGTATCGGCCAC
11_456   -----------------------------------------------AGAAAATAAGAGCCAACCACCAGTAGCTTATCAATACTGGCCGCTGGCTGAACTTCAGTATCAGCCAC
12_216   -----------------------------------------------AGAAAATAAGACCCAACCACCAGTAGCTTATCAATACTGGCCGCCGGCTGAACTTCAGTATCGGCCAC
12_419   -------------------------------------------------------------------------------------------------------------------
14_023   ----------TGCCAATCCAGACACAGTATCCACAATATCAGCCGGTAGAAAATAAAACCCAACCATCAGTAGTTTATGAACACCAGCCATCAGCCGAATTTCAGTATCGGCCGT
16_010   ATCTCGGCAATGCCAATCCAGATACAGTATCCACAATATCAGCCAGTAGAAAATAAAACCCAACCGCCATTAGCTTATCAATACTGGCTGCCAGCCGAGCTTCAGTATTGGCTGC
17_029   -------------------------------------------------------------------------------------------------------------------
19_157   GTCACAGCAATGCCAATCCAAATACAGTATCCACAATATCAGCTGGTAGAAAATAAGACCCAACTGCCAGTAGCCTATCAATACTGGCCGCCAGCCGAACTTCAGTATTGGCCGC
19_238   -----------------------------------------------AGAAAATAAGACCCAACCGCCAGTAGCTTATCAATAATGGCCGCCAGCCAAACTTCAGTATCGGCCAC
19_352   -----------------------------------------------AGAAAATAAGACCCAACCGCCAGTAGCCTATCAATACTGGCCGCCGGCTGAACTTCAGTATCGGCCAC
19_407   GTCACGGCAATGCCAATCCAAATACAGTATCCACAATATCAGCCGGTGGAAAATAAGACCCAACCGCCAGTAACCTATCAATACTGGCCGCCAGCCGAACTTCAGGATCAGCCGC
19_639   -------------------------------------------------------------------------------------------------------------------
1_059    ATCCCAGCAATGCCAATTCAGATACAGTATCCACAATATCAGCTGGTAGAAAATAAGACCCAACCACCGGTAGTTTATCAATACTGGCCGCCAGCTGAGCTTCAGTATGGGCTGT
1_061    ATCCCGGCCATGCCAATTCAGATACAGTATCCACAATATCAGCTGGTAGAAAATAAGACCCAACCACCGGTAGTTTATCAATACTGGCCGCCAGCTGAGCTTCAGTATGGGCCGT
1_259    -----------------------------------------------AGAAAATAAGACCCAACCGCCAGTAGCTTATCAATACTGGCCGCCGGCTGAACTTCAGTATCGGCCAC
1_504    -----------------------------------------------AGAAAATAAGACCCAACCGCCAGTAGCCTATCAATACTGGCCTCCGGCTGAACTTCAGTATCGGCCAC
1_555    -----------------------------------------------AGAAAATAAGACCCAACTGCCAGTAGCTTATCAATACTGGCCACCGGCCGAACTTCAGTATCGGCCAC
20_078   ATCACAGCAATGCCAATCCAGATACAGTATTCACAATATCAGCTGGTAGAAAGTAAGACCCAACCGCCAGTACCCTATCAATACTGACCGCCAGCCGAACTTCAGTATCAGCCGC
21_019   -----------------------------------------------AGAAAATAAGACCCAACCGCCAGTAGCTTATCAATACTAGCCGCCGGCTGAACTTCAGTATCGGCCAC
22_012   -----------------------------------------------AGAAAATAAGACCCAACCGCCAGTAGCCTATCAATACTGGCCTCCGGCTGAACTTCAGTATCGGCCAC
22_041   ATCCCGGCAATGCCAACTCAGATACAGTATCCACAATATCAGCCGGTAGAAAATAAGACCCAACCGCTGGTAGTTTATCAATACCGGCTGCCAACCGAGCTTCAGTATCGGCCTC
2_Tan    -----------------------------------------------AGAAAATAAGACCCAACCGCCAGTAGCCTATCAATACTGGCCTCCGGCTGAACTTCAGTATCGGCCAC
3_014    -----------------------------------------------AGAAAATAAGACCCAACCACCAGTAGCTTATCAATACTGGCTGCCGGCTGAATTTCAGTATCGGCCAC
3_209    ----------TGCTAATCCAGGCACAGTATCCACAATAGCAGGCAGTAGAAAATAAAACCCAACCATCGGTAGTTTATCAACACCAGCCGCCAGCCGAATTTCAGTATCGGCCGT
3_294    -----------------------------------------------AGAAAATAAGACCCAACCGCCAGTAGCTTATCAATACTGGCCGCCGGCTGAACTTCAGTATCTGCCAC
3_354    -----------------------------------------------AGAAAATAAGACCCAACCGCCAGTAGCCTATCAATACTGGCCGCCGGCTGAACTTCAGTATCGGCCAC
3_385    -----------------------------------------------AGAAAATAAGACCCAACCGCCAGAAGCTTATCAATACTGGCCGCCGGCTGAACTTCAGTATCGGCCAC
3_496    -----------------------------------------------AGAAAATAAGACCCAACCGCCAGTAGCTTATCAATACTGGCCGCCGGCTGAACTTCAGTATCGGCCAC
3_610    -----------------------------------------------AGAAAATAAGACCCAACCGCCAGTAGCCTATCAATACTGGCCACCGGCTGAACTTCAGTATCGGCCAC
4_008    -------------------------------------------------------------------------------------------------------------------
4_032    ----------TGCCAATCCAGGCACAGTATCCGCAATACCAGCCCGTAGAAAATAAA-CCCAACCATCGGTAGTTTATCAACACCAGCCGCCAGCCGTATTTCAGTATCCGCCGT
4_052    ----------TGCCAATCCAGGCACAGTATCCGCAATACCAGCCAGTAGAAAATAAAACCCAACCATCGGTAGTTTATCAACACAAGCCGTCAGCCGTATTTCAGTATCCGCCGT
4_068    ----------TGCCAATCCAGGCACAGTATCCGCAATACCAGCCAGTAGAAAATAAA-CCCAACCATCGGTAGTTTATCAACACCAGCCGCCAGCCGAATTTCAGTATCGGCCGT
4_716    -----------------------------------------------AGAAAATAAGACCCAACCGCCAGTAGCTTATCAATACTGGCCGCCGGCTGAACTTCAGTATCGGCCAC
4_742    -----------------------------------------------AGAAAATAAGACCCAACCACCAGTAGCTTATCAATACTGGCCACTGGCTGAACTTCAGTATCAGCCAC
5_147    -----------------------------------------------AGAAAATAAGACCCAACCGCCAGTAGCCTATCAATACTGGCTGCCGGCTGAACTTCAGTATCGGCCAC
5_259    -----------------------------------------------AGAAAATAAGACCCAACCACCAGTAGCTTAGCAATACTGGCCACTGGCCAAACTTCAGCATCAGCCAT
5_535    ATCCCGGCAATGCCAATCCAGACACAGTATCCACAATATCAGCTGGTAGAAAATAAAACCCAACCACTGGTAGTTTATCAATACTGGCCGCCAGTCAAATTTCAGTATCAGCTGC
5_544    -----------------------------------------------AGAAAATAAGACCCAACCGCCAGTAGCCTATCAATACTGGCCGCCGGCTGAACTTCAGTATCGGCCAC
6_098    GCCATGGCAATGCCAATCCAGATACAGTATCCACAATATCAGCAGGTAGAAAATAAGACCCAACTGCCAGTAGCCTATCAATACTGGCCGCCAGCCGAACTTCAGTATCGGCCGC
6_178    ATCCCGGCAATGCCAATTCAGATGCAGTATCCACAATATAAGCTGGTAGAAAATAAGACCCAACCACCAGTAATTTATCAGTACTGGCCGCCAGCCGACCTTCAGTATCGGCCAC
6_240    -------------------------------------------------------------------------------------------------------------------
6_324    -----------------------------------------------AGAAAATAAGACCCAACCGCCAGTAGCCTATCAATACTGGCCTCCGGCTGAACTTCAGTATCGGCCAC
7_345    -----------------------------------------------AGAAAATAAGACCCAACCGCCAGTAGCCTATCAATACTGGCCGCCGGCTGAACTTCAGTATCGGCCAC
7_450    GTCACGGCAATGCCAATCCAAATACAGTATCCACAATATCAGCCGGTAGAAAATAAGACCCAACTGCCAGTAGCCTATCAATACTGGCTGCCAGCCGAACTTAAGTGTCGGCCAC
8_047    ----------TGCCAATCCAGGCACAGTATCCGCAATACCAGCCAGTAGAAAATAAAACCCAACCATCGGTAGTTTATCAACACCAGCCGCCAGCCGCATTTCAGTATCCGCCGT
8_074    ----------TGCCAATCCAGGCACAGTATCCGCAATACCAGCCAGTAGAAAATAAAACCCAACCATCGGTAGTTTATCAACACCAGCCGCCAGCCGCATTTCAGTATCCGCCGT
8_094    ----------TGCCAATCCAGGCACAGTATCCGCAATACCAGCCAGTAGAAAATAAAACCCAACCATCGGTAGTTTATCAACACCAGCCGCCAGCCGCATTTCAGTATCCGCCGT
8_121    -------------------------------------------------------------------------------------------------------------------
8_235    -------------------------------------------------------------------------------------------------------------------
8_525    -----------------------------------------------AGAAAATAAGACCCAACCACCAGTAGCCTATCAATACTGGCCGCCGGCTGAACTTCAGTATCGGCCAC
8_559    ----------TGCCAATACAGACACAGTATCTACAATATCAGCTGGTTGAAAATAAAACCCAACCACTGGTAGTTTATCAACACTGGCCGCCAGCCGAATTTCAGTATCGGCCAT
9_307    -------------------------------------------------------------------------------------------------------------------
9_340    ATCCCAGCAATGCCAATCCAGATGCAGTATCCACAATATCAGCTGGTAGAAAATAAGACCCAACTGCCGGTAGCCTATCAATACTGGCCGCCAGCCAAACTTCAGTATCGGCCGC
HML2_Rep -----------------------------------------------AGAAAATAAGACCCAACCGCCAGTAGCCTATCAATACTGGCCTCCGGCTGAACTTCAGTATCGGCCAC
K113     -----------------------------------------------AGAAAATAAGACCCAACCGCCAGTAGCCTACCAATACTGGCCGCCGGCTGAACTTCAGTATCAGCCAC
K115     -----------------------------------------------AGAAAATAAGACCCAACCGCCAGTAGCCTATCAATACTGGCCTCCGGCTGAACTTCAGTATCGGCCAC
X_540    -------------------------------------------------------------------------------------------------------------------
Y_026    ----------TGCCAATCCAGACACAGTATCCACAATATCAGCTGGTGGAAAATAAAACCCAACCACCGGCAGCTTATCAACACCAGCCGCCAGCCGAATTTCAGTATCGGCCGT
94       -----------------------------------------------AGAAAATAAGAGCCAACCACCAGTAGCTTATCAATACTGGCCGCTGGCTGAACTTCAGTATCAGCCAC
```

```
          235  240  245  250  255  260  265  270  275  280  285  290  295  300  305  310  315  320  325  330  335  340  345
10_019    CCCC-AGAAAGTCAGTATGGATATCCAGGAACGCTCCCAGCACCACAGGGCAG----GGCGCCATACCCTCAGCCACCCACTAGCAGAC--------------------------
10_310    CCCC-AGAAAGTCAGTATGGATATCCAGGAATGCCCCCAGCACCACAGGGCAG----GGCGCCATACCCTCAGCCGCCCACTAGGAGAC--------------------------
11_233    -------------------------------------------------------------------------------------------------------------------
11_277    CCCC-AGAAAATCAGTATGGACAGCCAGGAGTGTTTCCAGCACCACAGGGCAG----GGCGCTATAGCCTCAGCCGCCCACTATAAGAC--------------------------
11_424    CCCC-AGAAAGTCAGTATGGATATCCAGGAATGCCCCCAGCACCACAGGGCAG----GGCGCCATACCCTCAGCCACCCACTAGGAGAC--------------------------
11_456    CCCC-AGAAAGTCAGTATGGATATCCAGGAATGCCCCCAGCACCACAGGGCAG----GGCGCCATACCCTCAGCCGCCCACTAGGAGAC--------------------------
12_216    CCCC-AGAAAGTCAGTATGGATATCCAGGAATGCCCCCAGCACCACAGGGCAG----GGCGCCATACCCTCAGCCGCCCACTAGGAGAC--------------------------
12_419    -------------------------------------------------------------------------------------------------------------------
14_023    CTCC-AGTGGTTCAGTATAGATCCCAGGTGTTGTGTCCTGTGCCAAATAGCAG----GGCAGTATAGCAACAACCCATGGCGATGGTGT--------------------------
16_010    CTCC-AGAGGTCCAATACAGACCTCAAGTGGTGTGTGCCATGCCAAATTGCAC----GGCATCGTACAAGCAACCCATGGCGGTGGTGTTTAATACGTCAGCACCACAGGGCGCG
17_029    -------------------------------------------------------------------------------------------------------------------
19_157    CCCT-AGAAAATCAGCATGGACAGCCAGGAATGTTTCCAGCACCACAGGGCAG----GGCACTATATCCGCAGCCTCCCACTATGAGAC--------------------------
19_238    CCCC-AGAAAGTCAGTATGGATATCCAGGAACGCTCCCAGCACCACAGGGCAG----GGCGCCATACCCTCAGCCACCCACTAGGAGAC--------------------------
19_352    CCCT-AGAAAGTCAGTATGGATATCCAGGAATGCCCCCAGCACCACAGGGCAG----GGCGCCATACCCTCAGCCGCCCACTAGGAGAC--------------------------
19_407    CCCC-AGAAAATCAGTATGGACAGCCAGGAATGTTTCCAGCACCACAGGGCAG----GGCGCTATATCCTCAGCAGCCCACTATGAGAT--------------------------
19_639    -------------------------------------------------------------------------------------------------------------------
1_059     CTCC-GGAGGTTCAATACAGACCTCAAGCTGTGTGTCCCGTGCCACATAGCAC----GGCACCTCACCAGCAACCCA-----GTGGTGT--------------------------
1_061     CTCC-GGAGGTTCAATACAGACCTCAAGCTGTGTGTCCCGTGCCACACAGCAC----GGCACCTCACCAGCAACCCA-----GCGGTGT--------------------------
1_259     CCCC-AGAAAGTCAGTATGGATATCCAGGAATGCCCCCAGCACCACAGGGCAG----GGCGCCATACCCTCAGCTGCCCACTAGGAGAC--------------------------
1_504     CCCC-AGAAAGTCAGTATGGATATCCAGGAATGCCCCCAGCACCACAGGGCAG----GGCGCCATACCCTCAGCCGCCCACTAGGAGAC--------------------------
1_555     CCCC-AGAAAGTCAGTATGGATATCTAGGAATGCCCCCAGCACCACAGGGCAG----GGAGCCATACCCTCAGCCGCCCACTAGGAGAC--------------------------
20_078    CCCCCAGAAAATCAGTATGGACAGCCAGGAACGTTTCCAGCACCACAGGGCACGCACGGCGATATAGCCTCAGCTGCCCACTATGAGAC--------------------------
21_019    CCCC-AGAAAGTCAGTATGGATATCCAAGAATGCCCCCAGCACCACAGGGCAG----GGCGCCATACCCTCAGCCGCCCACTAGGAGAC--------------------------
22_012    CCCC-AGAAAGTCAGTATGGATATCCAGGAATGCCCCCAGCACCACAGGGCAG----GGCGCCATACCCTCAGCCGCCCACTAGGAGAC--------------------------
22_041    CTTC-AGAGGTTCAATACAGACCTCAAGCGGTGTGTCCTGTGCCAAATAGCAC----GGCACCATACCAGCAACCCACAGCGATGGCGTCTAATTCACCAGCAACACAGGACGCG
2_Tan     CCCC-AGAAAGTCAGTATGGATATCCAGGAATGCCCCCAGCACCACAGGGCAG----GGCGCCATACCCTCAGCCGCCCACTAGGAGAC--------------------------
3_014     CCCC-AGAAAGTCAGTATGGACATCCAGGAATGCTCCCAGCACCACAGGGCAG----GGCGCCATACCCTCAGCCGCCCACTATGAGAC--------------------------
3_209     CTCC-AGAGGTTCAGTATGGATCTCAGGTGGTGCGTCCTGTGCTAAATAGCAA----GACACTATATCAACAACCCACGGCGATGGCGT--------------------------
3_294     CCCC-AGAAAGTCAGTATGGATATCCAGGAATGCCCCCAGCACTACAGGGCAG----GGCGCCATATCCTCAGCCGCCCACTGTGAGAC--------------------------
3_354     CCCC-AGAAAGTCAGTATGGATATCCAGGAATGCCCCCAGCACCACAGGGCAG----GGCGCCATACCCTCAGCCACCCACTAGGAGAC--------------------------
3_385     CCCC-AGAAAGTCAGTATGGATATCCAGGAATGCCCCCAGCACCACAGGGCAG----GGCGCCATACCCTCAGCTGCCCACTAGGAGAC--------------------------
3_496     CCCC-AGAAAGTCAGTATGGATATCCAGGAATGCCCCCAGCACCACAGGGCAG----GGCGCCATACCCTCAGCCGCCCACTAGGAGAC--------------------------
3_610     CCCC-AGAAAGTCAGTATGGATATCCAGGAATGCCCCCAGCACCACAGGGCAG----GGCGCCATACCCTCAGCCGCCCACTAGGAGAC--------------------------
4_008     -------------------------------------------------------------------CAGCAACCCACGGCGGTGGCGC--------------------------
4_032     CTCC-AGAGGTTCAGTATGGATCTCAGGCGGTGCGTCCTGTGCCAAATAGCAA----GGCACTATATCAACAACCCACGGCGATGGCGT--------------------------
4_052     CTCC-AGAGGTTCAGTATGGATCTCAGGCGGTGCGTCCTGTGCCAAATAGCAA----GGCACTATATCAACAACCCAAGGCGATGGCGT--------------------------
4_068     CTCC-AGAGGTTCAGTATGGATCTCAGGAGGTGCGTCCTGTGCCAAATAGCAA----GGCACTATATCAACAACCCACGGCGATGGCGT--------------------------
4_716     CCCT-AGAAAGTCAGTGTGGATATCCAGGAATGCCCCCAGCACCACAGGGCAG----GGCGCCATACCCTCAGCCGCCCACTAGGAGAC--------------------------
4_742     CCCC-AGAAAGTCAGTATGGACATCCAGGAATGCTGCCAGCACTACAGAATAG----GGTGCCACACCCTCAGCCGCCCACTTTGAGAC--------------------------
5_147     CCCC-AGAAAGTCAGTATGGATATCCAGGAATACCCCCAGCACCACAGGGCAG----GGTGCCATACCCTCAGCCGCCCACTAGGAGAC--------------------------
5_259     CCCC-AGAAAGTCAGTATGGACATTCAGGAATGCTCCCAGCACCACAGGGCAG----GGAGCCATACCCTCAGCCACCCACTATGACAC--------------------------
5_535     TTCC-AGAGGTTCAGTATAGATCTCAGGCGGGGTGTCCTGTCCCAAATAGCAG----GGCACTATATCAGCAACCCACAGCGATGGCGT--------------------------
5_544     CCCC-AGAAAGTCAGTATGGATATCCAGGAATGCCCCCAGCACCACAGGGCAG----GGCGCCATACCATCAGCCGCCCACTAGGAGAC--------------------------
6_098     CCCC-AGAAAATCAGTATGGACACCCAGGAATGTTTCCAGCACCACAGGGCAG----GGCTCTAGATCCTCAGCCGTCCACTGTGAGAC--------------------------
6_178     CTCC-AGAGGTTAAATACAGACCTCAAGTGGTGTGTCCTGTGCCAAATAGCAC----GGCACCATACCAGCAACCCACGGCAGTGGTGT--------------------------
6_240     -------------------------------------------------------------------------------------------------------------------
6_324     CCCC-AGAAAGTCAGTATGGATATCCAGGAATGCCCCCAGCACCACAGGGCAG----GGCGCCATACCCTCAGCCGCCCACTAGGAGAC--------------------------
7_345     CCCC-AGAAAGTTAGTATGGATATCCAGGAATGCCCCCAGCACCACAGGGCAG----GGCGCCATACCCTCAGCCGCCCACTAGGAGAC--------------------------
7_450     CCCC-GGAAAATCAGTATGGACAGCCAGGAATGTTCCCAGCCCCACAGGACAG----GGCGCCATACCCTCAGCCACCCACTATGAGAC--------------------------
8_047     CTCC-AGAGGTTCAGTATGGATCTCAGGCGGTGCGTCCTGTGCCAAATAGCAA----GGCAGTATATCAACAACCCACGGCGATGGCGT--------------------------
8_074     CTCC-AGAGGTTCAGTATGGATCTCAGGCGGTGCGTCCTGTGCCAAATAGCAA----GGCACTATATCAA---CCCACGGCGATGGCGT--------------------------
8_094     CTCC-AGAGGTTCAGTATGGATCTCAGGCGGTGCGTCCTGTGGCAAATAGCAA----GGCACTATATCAACAACCCACGGCGATGGCGT--------------------------
8_121     -------------------------------------------------------------------------------------------------------------------
8_235     -------------------------------------------------------------------------------------------------------------------
8_525     CCCC-AGAAAGTCAGTATGGATATCCAGGAATGCCCCCAGCACCACAGGGCAG----GGCGCCATACCCTCAGCCGCCCACTAGGAGAC--------------------------
8_559     CTCC-AGAGGTGCAGTATAGATCCCAGATGGGA--TCCTGTGCCAAATAGTAG----GGCACTATATCAACAACCCACGGCGATGGTGT--------------------------
9_307     -------------------------------------------------------------------------------------------------------------------
9_340     CCCC-AGAGTATCAGTATGGATGGCCAGGA-TGTTTCCAGCACCGCAGGGCAG----GGCGCTATAGCCGCAGCCGCCCACTATGAGAC--------------------------
HML2_Rep  CCCC-AGAAAGTCAGTATGGATATCCAGGAATGCCCCCAGCACCACAGGGCAG----GGCGCCATACCCTCAGCCGCCCACTAGGAGAC--------------------------
K113      CCCC-AGAAAGTCAGTATGGATATCCAGGAATGCCCCCAGCACCACAGGGCAG----GGCGCCATACCCTCAGCCGCCCACTAGGAGAC--------------------------
K115      CCCC-AGAAAGTCAGTATGGATATCCAGGAATGCCCCCAGCACCACAGGGCAG----GGAGCCATACCCTCAGCCGCCCACTAGGAGAC--------------------------
X_540     -------------------------------------------------------------------------------------------------------------------
Y_026     CTCC-AGAGGTTCAGTATAGAACTCAGGTGGTGCGCCCAAC-CCAAATAGCAG----GGCACTATATCAACATCCCACGGCAATGGTGT--------------------------
94        CCCC-AGAAAGTCAGTATGGATATCCAGGAATGCCCCCAGCACCACAGGGCAG----GGCGCCATACCCTCAGCCGCCCACTAGGAGAC--------------------------
```

```
           350  355  360  365  370  375  380  385  390  395  400  405  410  415  420  425  430  435  440  445  450  455  460
10_019     -------------------------------TTAATCATACGGCACCACCTAGTAGACAGGGTAGTGAATTACATGAAATTATTGATAAG----------TCAAGAAAGGAAGGA
10_310     -------------------------------TTAATCCTACGGCACCACCTAGTAGACAGGGTAGTGAATTACATGAAATTATTGATAAA----------TCAAGAAAGGAAGGA
11_233     --------------------------------------------------------------------------------------------------------GGCCATTTGGG
11_277     -------------------------------TTTATCCTACAGCACCACCTAGTGGACAAGGTAGTGCATTACATAAAATTATTGATAAG----------GCAAGGAAACAAGGA
11_424     -------------------------------TTAATCCTACGGCACCACCTAGTAGACAGGGTAGTGAATTACATGAAATTATTGATAAA----------TCAAGAAAGGAAGGA
11_456     -------------------------------TTAATCCTACGGCACCGCCTAGTAGACAGGGTAGTGAATTACATGAAATTATTGAGA---------------AGAAAGGAAGGA
12_216     -------------------------------TTAATCCTACGGCACCACCTAGTAGACAGGGTAGTGAATTACATGAAATTATTGATAAA----------TCAAGAAAGGAAGGA
12_419     --------------------------------------------------------------------------------------------------------GGCTGTT-GGG
14_023     -------------------------------TTGATCCTACACTACCACCTAGTGGACAAGATAGTGCACTGCAAAAAATCATTGATAAA----------GCCAGAAAACAGGGA
16_010     GCGCTGTGTCCTCAGCCGCCCACTATGAGACTTAATCCAACAGCACCACCTAGTGGACAAGGTAGCACACTGCACGCGATCATTGATGAA----------GCTAGAAAACAGGGA
17_029     -------------------------------------------------------------------------------------------------------------------
19_157     -------------------------------TTAATCCTACATCACCACCTAGTAGACAGGGTGGTGCATTACATAAAATTATT---AAA----------GCAAGAAAACAAGGA
19_238     -------------------------------TTAATCCTATGGTACCACCTAGTAGACAGGGTAGTGAATTACCAGAAATTATTGATAAG----------TCAAGAAAGGAAGGA
19_352     -------------------------------TTAATCCTACGGCACCACCTAGTAGACGGGGTAGTGAATTACATGAAATTATTGATAAA----------TCAAGAAAGGAAGGA
19_407     -------------------------------TTAATCCTACACCACCACCTAGTGGACAGGGTAGTGCATTACATAAAATTATTGATAAG----------GCAAGAAAACAAGGA
19_639     --------------------------------------------------------------------------------------------------------GGTCATA-GGG
1_059      -------------------------------TTAATCCTACATCACCACCTAGTGGACAAGGTAGTACACTGCATGAAATCATTGATAAA----------GCCAGAAAACAGGGA
1_061      -------------------------------TTAGTCCTACATCACCACCTAGTGGACAAGGTAGTACACTGCATGAAATTATTGATAAA----------GCCAGAAAACAGGGA
1_259      -------------------------------TTAATCCTATGGCACCACCTAGTAGACAGGGTAGTGAATTACATGAAATTATTGATAAA----------TCAAGAAAGGAAGGA
1_504      -------------------------------TTAATCCTACGGCACCACCTAGTAGACAGGGTAGTGAATTACATGAAATTATTGATAAA----------TCAAGAAAGGAAGGA
1_555      -------------------------------
--AATCCTATGGCACCACCTAGTAGACAGGGTAGTGAATTACATGAAATTATTGAGAAG----------TCAAGAAAGGAAGGA
20_078     -------------------------------TTAATCCTACACCACCACCTAGTGGACAGGATGGTGCATTACATGAAATTATTGAGAAG----------GCAAGAAAACAAGGA
21_019     -------------------------------TTAATCCTACGGCACCACCTAGTAGACAAGGTAGTGAATTACATGAAATTATTGATAAA----------TCAAGAAAGGAAGGA
22_012     -------------------------------TTAATCCTACGGCACCACCTAGTAGACAGGGTAGTGAATTACATGAAATTATTGATAAA----------TCAAGAAAGGAAGGA
22_041     GCGCTGTATCCTCAGCCGCCCACTGTGAGACTTAATCCTACAGCATCACGTAGTGGACAGGGTGGTGCACTGCATGCAGTCATTGATGAA----------GCCAGAAAACAGGGC
2_Tan      -------------------------------TTAATCCTACGGCACCACCTAGTAGACAGGGTAGTAAATTACATGAAATTATTGATAAA----------TCAAGAAAGGAAGGA
3_014      -------------------------------TTAATCCTACAGCACCACCTAATAGACAGGGTAGTGCATTACATAAAATTATTGATAAG----------TCAAGAAAGCAAGGA
3_209      -------------------------------TTGATCCTACAGCACCACCTAGTGGACAAGATGGTGCACTGCATGAGACCATTGCTACA----------GCCAGAAAACAGGGA
3_294      -------------------------------TTAATCCTACAGCATCACGTAGTGGACAAGGTGGTACACTGCACGCAGTCATTGATGAA----------GCCAGAAAACAGGGA
3_354      -------------------------------TTAATCCTACGGCACCACCTAGTAGACGGGGTAGTGAATTACATGAAATTATTGATAAA----------TCAAGAAAGGAAGGA
3_385      -------------------------------TTAATCCTATGGCACCACCTAGTAGACAGGGTAGTGAATTACATGAAATTATTGATAAA----------TCAAGAAAGGAAGGA
3_496      -------------------------------TTAATCCTACGGCACCACCTAGTAGACAGGGTAGTGAATTACATGAAATTATTGATAAA----------TCAAGAAAGGAAGGA
3_610      -------------------------------TTAATCCTACGGCACCACCTAGTAGACAGGGTAGTGAATTACATGAAATTATTGATAAA----------TCAAGAAAGGAAGGA
4_008      -------------------------------TTAATCCTACAGCACCACCTAGTGGACAATGTAGCACACTGTATGAAATCATTGATTAA----------GCCAGAAAACAGGGA
4_032      -------------------------------TTGATCTTACAGTACCACCTAGTGGACAAGATAGTGCACTGCATGAGACCATTGCTACA----------GCCAGAAAACAGGGA
4_052      -------------------------------TTGATCTTACAGTACCACCTAGTGGACAAGGTAGTGCACTGCATGAGACCATTGCTACA----------GCCAGAAAACAGGGA
4_068      -------------------------------TTGATCTTACAGTACCACCTAGTGGACAAGATAGTGCACTGCATGAGACCATTGCTACA----------GCCAGAAAACAGGGA
4_716      -------------------------------TTAATCCTACGGCACCACCTAGTAGACAGGGTAGTGAATTACATGAAATTATTGATAAA----------TCAAGAAAGGAAGGA
4_742      -------------------------------TTAATCCTATGGCACCACCTAGTAGACAGGGTAGTGCATTACATAAAATTATTGTCAAGAAAATTATAGTCAAGAAAGCAAGGA
5_147      -------------------------------TTAATCCTATGGCACCACCTAGTAGACAGGGTAGTGAATTACATGAAATTATTGATAAA----------TCAAGAAAGGAAGGA
5_259      -------------------------------TTAATCCAACAGCACCACCTAGTCAACAGGGTAGTGCGTTACATAAAATTATTGATAAT----------TCAAGAAACCAAGGA
5_535      -------------------------------TTGATCCTACAGCACCAGCTAGTGTACAAGATAGTACACTGCATAAAACCATT-----A----------GCCAGAAAACAGGGA
5_544      -------------------------------TTAATCCTATGGCACCACCTAGTAGACAGGGTAGTGAATTACATGAAATTATTGATAAA----------TCAAGAAAGGAAGGA
6_098      -------------------------------TTAATCCTACAGCACTACCTAGTGGACAGCATAGTGCATTACATAAAATTATTGATAAG----------GCAAGAAAACAAGGA
6_178      -------------------------------TTAATCCTACCGCACCACCTAGTGGACAAGGTAGTACACTGCATGAAATTATTGATAAA----------GCCAGTAAACAGGGA
6_240      --------------------------------------------------------------------------------------------------------GGCCATT-GGG
6_324      -------------------------------TTAATCCTACGGCACCACCTAGTAGACAGGGTAGTGAATTACATGAAATTATTGATAAA----------TCAAGAAAGGAAGGA
7_345      -------------------------------TTAATCCTACGGCACCACCTAGTAGACAGGGTAGTGAATTACATGAAATTATTGATAAA----------TCAAGAAAGGAAGGA
7_450      -------------------------------TTAATCCTACAGCACCACCTAGTGAGCAGGGTAGTGTATTACATAAAATTATTGATGAG----------GCAAGAAAACAAAGA
8_047      -------------------------------TTGATCTTACGGTACCACCTAGTGGACAAGATAGTGCACTGCATGAGACCATTGCTACA----------GCCAGAAAACAGGGA
8_074      -------------------------------TTGATCTTACGGTACCACCTAGTGGACAAGATAGTGCACTGCATGAGACCATTGCTACA----------GCCAGAAAACAGGGA
8_094      -------------------------------TTGATCTTACGGTACCACCTAGTGGACAAGATAGTGCACTGCATGAGACCATTGCTACA----------GCCAGAAAACAGGGA
8_121      -------------------------------------------------------------------------------------------------------------------
8_235      --------------------------------------------------------------------------------------------------------GGCCTTT-GGG
8_525      -------------------------------TTAATCCTACGGCACCACCTAGTAGACAGGGTAGTGAATTACATGAAATTATTGATAAA----------TCAAGAAAGGAAGGA
8_559      -------------------------------TTGATCCTACAGTACCAGCTAGTGGACAAGATAGTGCACTGCATGAGACCGTTGATACA----------GCCAGAAAACAGGGA
9_307      --------------------------------------------------------------------------------------------------------GGCCATT-GGG
9_340      -------------------------------GTAATCCTACAGCACCACCTAGTGGACAGGGTCGTGCATTACATAAAATTATTGAAAG----------ACA---AAACAAGGG
HML2_Rep   -------------------------------TTAATCCTACGGCACCACCTAGTAGACAGGGTAGTAAATTACATGAAATTATTGATAAA----------TCAAGAAAGGAAGGA
K113       -------------------------------TTAATCCTACGGCACCACCTAGTAGACAGGGTAGTGAATTACATGAAATTATTGATAAA----------TCAAGAAAGGAAGGA
K115       -------------------------------TTAATCCTACGGCACCACCTAGTAGACAGGGTAGTGAATTACATGAAATTATTGATAAA----------TCAAGAAAGGAAGGA
X_540      --------------------------------------------------------------------------------------------------------GGCCGTC-AGG
Y_026      -------------------------------TTGATCCTACAGTACCACCTAGTGGACAAGATAGTGCACTCCATGAAATCATTGATAAA----------GCCAGAAAACAGGGA
94         -------------------------------TTAATCCTACGGCACCGCCTAGTAGACAGGGTAGTGAATTACATAAAATTATTGATGAG----------GCAAGAAAACAAAGA
```

```
           465  470  475  480  485  490  495  500  505  510  515  520  525  530  535  540  545  550  555  560  565  570  575
10_019     GA--CACTGAGGCGTGGCAATTCCCAGTAACGTTAGAACCGATACCACCTGGAGAAGGA-----------GCCCAAGAGGGAGAGCCTCTCACAGTTGAGGCCAGATACAAGTCT
10_310     GA--TACTGAGGCGTGGCAATTCCCAGTAACGTTAGAACCGATGCCACCTGGAGAAGGA-----------GCCCAAGAGGGAGAGCCTCTCACAGTTGAGGCCAGATACAAGTCT
11_233     GC----TAAAACCAGGATGGCCACC--TCCTCCTCAGCCGAGTGAGTGCTGGAGGAGGG-----------AGCCTGAAACTAGGCTCACTGTGACTCA-TTTGTGGCACCCATTA
11_277     GA--TATTGAGGCGTGGCAATTCCCAGTA---------CCGATGCCACCTGGAGAAGGG-----------GCCCAAGAAGGAGCGCCTACCCTAGTTGGGGCCAGATATGAGTCC
11_424     GA--TACTGAGGCATGGCAATTCCCAGTAACGTTAGAACCGAAGCCACCTGGAGAAGGA-----------GCCCAAGAGGGAGAGCCTCCCACAGTTGAGGCCAGATACAAGTCT
11_456     GA--TACTGAGGCGTGGCAATTCCCAGTAACGTTAGAACCGATGCCACCTGGAGAAGAA-----------GCCCAAGAGGGAGAGCCTCTCACAGTTGAGGCCAGATACAAGTCT
12_216     GA--TACTGAGGCATGGCAATTCCCAGTAATGTTAGAACCGATGCCACCTGGAGAAGGA-----------GCCCAAGAGGGAGAGCCTCCCACAGTTGAGGCCAGATACAAGTCT
12_419     GT----TAAAAGCACGATGGCCACC--TCCTCCTCAGCCGAGTGAGTGCTGGGGGAGGG-----------AGCCTGAAACTACGCTTGCTGCGACTTGGCTCGTGGCACTCATTA
14_023     GA--TCTTGAGGCAGGGCAGTTCCTGGTAATTTTACAACCAATACCGGCTGGAAAACGG-----------AGTCAAGCAGGAGCGTCTGTCCAAACTGAGGCTAGATATGAATCT
16_010     GA--TCTTGAGGCGTGGCAGTTCCTGGTAATTTTACAATCGGTACCGGCCGGGGAAGGG-----------GCTCCAGCAGGAGCACCTGCGGTGGCTAATGCTAGATATGAACGC
17_029     -------------------------------------------------------------------------------------------------------------------
19_157     GA--TATCGAGGCGTGGCAGTTCCCAGTAATATTAGAGCCGATACCACCTGAAGAAGGG-----------GCCCAAGAGGGAGAGCCTCCCCTAGCTGAGGCCAGATATGAGTCC
19_238     GA--TATTGAGGCATGGCAATTCCCAATAATGTTAGA------ACCACCTGGAGAAGGA-----------GCCCAAGAGGGAGAGCCTCTCACAGTTGAGGCCAGATACAAGTCT
19_352     GA--TACTGAGGCATGGCAATTCCCAGTAACGTTAGAACCGATGCCACCTGGAGAAGGA-----------GCCCAAGAGGGAGAGCCTCCCACAGTTGAGGCCAGATACAAGTCT
19_407     GA--TACCGAGGCATGGCAATTCCCAGTAATATTAGAACCGATACCACCTGAAGAAGAG-----------GCCCAAGAGGGAGCGCCTCCCCTAGCTGAGGCCAGATATGAGTCC
19_639     GC----TAAAACCACGATGGCCACC--TCCTCCTCAGCCAAGTGAGTGCTGGGGGAGGG-----------AGCCTGAAACTAGGCTCACTGCAACTTGGCTCGCTGTGCCCATTA
1_059      GA--TCGTGAGGCATGGCAGTTCCTTGTAATTTTACAACCAATACCAGCTGGGAAAGGG-----------GGGCAAGCTGGAGCGTCTGTCCAAACTGAGGCTAGATATGAATCT
1_061      GA--TCTTGAGGCATGGCAGTTCCTTGTAATTTTACAACCAATACCAGCTGGGAAAGGG-----------GGGCAAGCTGGAGCGTCTGTCCAAACTGAGGCTAGATATGAATCT
1_259      GA--TACTGAGGCATGGCAATTCCCAGTAACGTTAGAACCAATGCCACCTGGAGAAGGA-----------GCCCAAGAGGGAGAGCCTCCCACAGTTGAGGCCAGATACAAGTCT
1_504      GA--TACTGAGGCATGGCAATTCCCAGTAACGTTAGAACCGATGCCACCTGGAGAAGGA-----------GCCCAAGAGGGAGAGCCTCCCACAGTTGAGGCCAGATACAAGTCT
1_555      GA--TACTGAGGCGTGGCAATTCCCAGTAACGTTAGAACCGATGCCACCTGGAGAAGGA-----------GCCCAAGAGGGAGAGCCTCTCACAGTTGAGGCCAGATACAAGTCT
20_078     GAGATACTGAGGCGTTTTAATTTCAAGTAATATTAGAGCCCATACTACCTGGAGAAGGG-----------CCC--------------TCCCCAAGCTGATGCCAGATATGAGTCC
21_019     GA--TACTGAGGCATGGCAATTCCCAGTAATGTTAGAACCGATGCCACCTGGAGAAGGA-----------GCCCAAGAGGGAGAGCCTCTCACAGTTGAGGCCAGATACAAGTCT
22_012     GA--TACTGAGGCATGGCAATTCCCAGTAACGTTAGAACCGATGCCACCTGGAGAAGGA-----------GCCCAAGAGGGAGAGCCTCCCACAGTTGAGGCCAGATACAAGTCT
22_041     GA--TCTTGAGGCATGGCGGTTCCTGGTAATTTTACAACTGGTACAGGCCGGGGAAGAG-----------ACTCAAGTAGGAGCGCCTGCCCGAGCTGAGACTAGATGTGAACCT
2_Tan      GA--TACTGAGGCATGGCAATTCCCAGTAACGTTAGAACCGATGCCACCTGGAGAAGGA-----------GCCCAAGAGGGAGAGCCTCCCACAGTTGAGGCCAGATACAAGTCT
3_014      GA--TATTGAGGCGTGGCCATTCCCACTAATGTTAGAACTGATACCACCTGGAGAAGGA-----------GCCCAAGAGGGAGAGCCTCCCACAGTTGAGGCCAGATACAAGTCT
3_209      GA--TCTTGAGGCATGGCAGTATCCGGTAATTTTACAACCCATACCGGCCAGAAAAGGG-----------AGTCAAACAGGAGCATCTGTCTGAACTGAGGCTAGATATGAATCT
3_294      GA--TCTTGAGGCATGGCGGTTCCTGGTAATTTTACAACTGGTACAGGCCGGGGAAGAG-----------ACTCAAGTAGGAGCGCCTGCCCGAGCTGAGACTAGATGTGAACCT
3_354      GA--TACTGAGGCATGGCAATTCCCAGTAACGTTAGAACCGATGCCACCTGGAGAAGGA-----------GCCCAAGAGGGAGAGCCTCCCACAGTTGAGGCCAGATACAAGTCT
3_385      GA--TACTGAGGCGTGGCAATTCCCAGTAACATTAGAACCGATGCCAACTGGAGAAGGA-----------GCCCAAGAGGGAGAGCCTCCCACAGTTGAGGCCAGATACAAGTCT
3_496      GA--TACTGAGGCATGGCAATTCCCAGTAACGTTAGAACCGATGCCACCTGGAGAAGGA-----------GCCCAAGAGGGAGAGCCTCCCACAGTTGAGGCCAGATACAAGTCT
3_610      GA--TACTGAGGCATGGCAATTCCCAGTAACGTTAGAACCGATGCCACCTGGAGAAGGA-----------GCCCAAGAGGGAGAGCCTCTCACAGTTGAGGCCAGATACAAGTCT
4_008      GA--TCTTGAGGCATGGCAGTTCCCAGTAATTTTACAACCGGTACCAGCCGGGGAAGAG-------------ACAAGTAGGAGTGCCTACCCAAGCTGAGGCTAGATATGAATCT
4_032      GA--TCTTGAGGCATGGCAATATCCGGGAATGTTACAACCGATGCCGGCCGGGAAAGGG-----------AGTCAAGCAGGAGCGTCTGTCTGAACTGAGACCAGATATGAATCT
4_052      GA--TCTTGAGGCATGGCAATATCCGGGAATGTTACAAACAATGCCGGCCGGGAAAGGG-----------AGTCAAGCAAGAGCGTCTGTCTGAACCAAGACTAGATATGAATCT
4_068      GA--TCTTGAGGCATGGCAATATCTGGTAATGTTACAACCGATGCCGGCCGGGAAAGGG-----------AGTCAAGCAGGAGCGTCTGTCTGAACTGAGACCAGATATGAATCT
4_716      GA--TACTGAGGCATGGCAATTCCCAGTAACGTTAGAACCGATGCCACCTGGAGAAGGA-----------GCCCAAGAGGGAGAGCCTCCCACAGTTGAGGCCAGATACAAGTCT
4_742      GA--TACTGAGGTGTGGCAATTCCCAGTAATGTTGGAACTGATGCCACCTGGAGAAGGG-----------GCCCAAGAGGGAGAGCCTCCCACAGTTGAGGCCAGATACAAGTCT
5_147      GA--TACTGAGGCATGGCAATTCCCAGTAACGTTAGAACCGATGCCACCTGGAGAAGGA-----------GCCCAAGAGGGAGAGCCTCTCACAGTTGAGGCCAGATACAAGTCT
5_259      GA--TATTGAGGCATGGCAATTCCCAGTAACGTTAGAACCAATACCACCTGGAGAAGGG-----------GCCCAAGAGGGAGAGCCTCAAACAGTTGAGGCCAGATATGAGTCT
5_535      GA--TCTTGAGGCATGGCAGTACCCGGTAATTTTACAACCGATACTGGCTGGGAAAGGG-----------AGTCAAGCAGGAGCCTCTGTCCAAACTGAGATTAGATATGAATCT
5_544      GA--TACTGAGGCATGGCAATTCCCAGTAACGTTAGAACCGATGCCACCTGGAGAAGGA-----------GCCCAAGAGGGAGAGCCTCCCACAGTTGAGGCCAGATACAAGTCT
6_098      GA--TACTGAGGCGTGACAATTCCCAGTAACATTAGAACTGATACCACCTGGAGAAGGG-----------GCCCAAGAGGGAGTGCCTCCCCTAGCTGAGGCCAGATATGAGTCC
6_178      GA--TCTTGAGGCATGGGAGTTCCCGGTAATTTTACAACCGATACCGGCCGGGAAAGGG-----------ACTCTAGCAGGAGCGTCTGTCCAAACTGAAGCTAGATATGAATCT
6_240      GC----TAAAACCACAATGGCCACA--TCCTCCTCAGCCAAGTGATTGCTGGGGGAGGG-----------AGCCTGAAACTAGGCTCGCTGTGACTTGGCTCGCAGCACTCATTA
6_324      GA--TACTGAGGCATGGCAATTCCCAGTAACGTTAGAACCGATGCCACCTGGAGAAGGA-----------GCCCAAGAGGGAGAGCCTCCCACAGTTGAGGCCAGATACAAGTCT
7_345      GA--TACTGAGGCATGGCAATTCCCAGTAACGTTAGAACCGATGCCACCTGGAGAAGGA-----------GCCCAAGAGGGAGAGCCTCTCACAGTTGAGGCCAGATACAAGTCT
7_450      GA--TATTGAGGCATGGCAATTCCCAGTAATATTAGAACCAATACCACCTGGAGAAGGG-----------GCCCAAGAGGGAGAGCCTCCCCTAGCTGAGGCCAGATATGAGTCT
8_047      GA--TCTTGAGGCATGGCAATATCCGGTAATGTTACAACAGATGCCGGCCGGGAAAGGC-----------AGTCAAGCA------------------------------------
8_074      GA--TCTTGAGGCATGGCAATATCCGGTAATGTTACAACCGATGCCGGCCGGGAAAGGG-----------AGTCAAGCA------------------------------------
8_094      GA--TCTTGAGGCATGGCAATATCCGGTAATGTTACAACAGATGCCGGCCGGGAAAGGC-----------AGTCAAGCA------------------------------------
8_121      -------------------------------------------------------------------------------------------------------------------
8_235      GC----TAAAACCACGATGGCCACC--TCCTCCTCAGCTGAGTGAGTGCTGGGGGAGGG-----------AACCTGAAACTAGGCTCGCTGTGACTTGGTTCACAGCACCCATTA
8_525      GA--TACTGAGGCATGGCAATTCCCAGTAACGTTAGAACCGATGCCACCTGGAGAAGGA-----------GCCCAAGAGGGAGAGCCTCTCACAGTTGAGGCCAGATACAAGTCT
8_559      GA--TCTTGAGGCATGGCAGTATCCGGTAATGTTACAACCGATACCGGCCGGGAAAGGGCCAGGAAAGGGAGTCAAGCAGGAGCGTCTGTCCGAACGGAGGCTAGGTAAGAATAT
9_307      GC----TACAACCACAATGGCCACC--TCCTCCTCAGCCGAGTGAGTGCTGGGGGAGGC-----------AGCCTGAAACTAGGCACACTGCGACTTGGCTCGTGGCACTCATTA
9_340      GA--TACTGACGCGTGGCAGACCCCGGTAATATTAGAACCCATACCACCTGGAAGAGGG-----------GCCCAAGAGGGAGCGCCTCCCCGAGCTGATGCCAGAAATGGGTCC
HML2_Rep   GA--TACTGAGGCATGGCAATTCCCAGTAACGTTAGAACCGATGCCACCTGGAGAAGGA-----------GCCCAAGAGGGAGAGCCTCCCACAGTTGAGGCCAGATACAAGTCT
K113       GA--TACTGAGGCATGGCAATTCCCAGTAACGTTAGAACTGATGCCACCTGGAGAAGGA-----------GCCCAAGAGGGAGAGCCTCCCACAGTTGAGGCCAGATACAAGTCT
K115       GA--TACTGAGGCATGGCAATTCCCAGTAACGTTAGAACCGATGCCACCTGGAGAAGGA-----------GCCCAAGAGGGAGAGCCTCCCACAGTTGAGGCCAGATACAAGTCT
X_540      GC----TAAAACCACGATGGCCACC--TCCTCCTCAGTCGAGTGAGTGCTGGGGGAGGG-----------AGCCTGAAACCAGGCTGGCTGCAACTCGGCTCGCGGTGCCCATTA
Y_026      GA--TCTTGAGGCATGGCAGTTCCCAGTAATTTTACAACCCAC-CCGGCCGGGAAAGGG-----------AGTCAAGCAGGAGCGTCTGTCCGAACTGAGTGTAGATATGAATCT
94         GA--TATTGAGGCATGGCAATTCCCAGTAATATTAGAACCAATACCACCTGGAGAAGGG-----------GCCCAAGAGGGAGAGCCTCCCCTAGCTGAGGCCAGATATGAGTCT
```

```
           580  585  590  595  600  605  610  615  620  625  630  635  640  645  650  655  660  665  670  675  680  685  690
10_019    TTTTCGATAAAAATGCT-AAAAGATATGAAAGAGGGAGTAA--AACACTATGGACCCAACTCCCCTTATATGAGGACATTATTAGATTCCATTGCTCATGGACATCATAGACTCA
10_310    TTTTCGATAAAAATGCT-AAAAGATATGAAAGAGGGAGTAA--AACAGTATGGACCCAAATCCCCTTATATGAGGACATTATTAGATTCCATTGCTCATGGACAT---AGACTCA
11_233    TTGCCCAACCTGCAGTT-----CACTATGGTGAAGGAGCAA--TTCAGACTCACCCTGCAGCATCCGTCTGGGTCAAACAGTGG---CTGCTCCCTAAGGAATAGTTGGGGGCG
11_277    TTTTCTATAAAAATGTT-AAAAGATATGAAAGAGGGAGTAA--AACAGTATGGACCCAACTCCCCTTATATGAGAACATTATTAGGTTCCACTGCTCATGGACAT---AGACTCA
11_424    TTTTCGATAAAAATGCT-AAAAGATATGAAAGAAGGAGTAA--AACAGTATGGACCCAACTCCCCTTATATGAGGACATTATTAGATTCCATTGCTCATGGACAT---AGACTCA
11_456    TTTTTGATAAAAATGCT-AAAAGATATGAAAGAGGGAGTAA--AACAGTATGGACCCAACTCCCCTTATATGAGTACATTATTAGATTCCATTGCTCATGGACAT---AGACTCA
12_216    TTTTCAATAAAAATGCT-AAAAGATATGAAAGAAGGAGTAA--AACAGTATGGACCCAACTCCCCTTATATGAGGACATTATTAGATTCCATTGCTCATGGACAT---AGACTCA
12_419    TTGCCCAACCTGCAGTT-----CACTACGGTGAAGGAGCAA--TTCAGACTTGCCCTGCAGCTTCCTGTATGGGTCAAAGAGTGG---CCGCTCTCTAAGGAAAAGTTGGGGGTG
14_023    TTCACCATGAAAATGTT-AAAAGATATGAAGGAGGCAGTTA--AACAATATGGACCCAACTCTCCTTATATGAGAACATTATTAGATTCCATTGCTAATGGGAAT---AGACTTA
16_010    TTCACCATGAAAATGTT-AAAAGACATGAAGGAAGGAGTTA--AACAATATGGACCCAACTCACCTTCTATGAGAACATTATTAGATTCCATTGCTCCAGCAGG----------
17_029    ------------------------------------------GTCAGTATGGTCCCAATTCGCCTTTCATCCGATCGCTATTGCAATCTGTGGCTCAGAATAAG---CTATTGA
19_157    TTTTCTATAAAAATGCT-AAAAGATGTGAAAGAGGGAGTAA--AA-AGTATGGACTCAACTCCCCTTATTTGAGGACATTATTAATTCCATTGCTCATGGACAT---AGACTCA
19_238    TTTTCGATAAAAATGCT-AAAAGATATGAAAGAGGGAGTAA--AACAGTATGGACCCAACTCTCCTTATATGAGGACATTATTAGATTCCATTGCTCATGGACAT---AGACTCA
19_352    TTTTCGATAAAAATGCT-AAAAGATATGAAAGAGGGAGTAA--AACAGTATGGACCCAACTCCCCTTATATGAGGACATTATTAGATTCCATTGCTCATGGACAT---AGACTCA
19_407    TTTTCCATAAAAATGCT-AAAAGATATGAAAGAGGGAGTAA--AACAGTATGGACCCAACTCCCCTTATATGAGGACATTATTAGATTCCATTGCTCATGGACAT---AGACTCA
19_639    TTGCCCAACTTACAGTT-----CATTATGGTGAAGGAGCAA--TTCAGACTTGCCTTGCAGCATCCTGTCTGAGTCAAACAGTGG---CTGCTCCCTAAGGAAAATTTGGGGGCG
1_059     TTCACCATGAAAATGTT-AAAAGATATGAAGGAAGGAGTTA--AACAATATGGACCCAACTCCCCTTATATGAGAACTTTATTAGATTCCATTGCTCATGGAAAT---AGACTTA
1_061     TTCACCATGAAAATGTT-AAAAGATATGAAGGAAGGAGTTA--AACAATATGGACCCAACTCCCCTTATATGAGAACTTTATTAGATCCCATTGCTCATGGAAAT---AGACTTA
1_259     TTTTCGATAAAAATGCT-AAAAGATATGAAAGAGGGAGTAA--AACAGTATGGACCCAACTCTCCTTATATGAGGACATTATTAGATTCCATTGCTCATGGACAT---AGACTCA
1_504     TTTTCGATAAAAATGCT-AAAAGATATGAAAGAGGGAGTAA--AACAGTATGGACCCAACTCCCCTTATATGAGGACATTATTAGATTCCATTGCTCATGGACAT---AGACTCA
1_555     TTTTAGATAAAAATGCT-AAAAGATATGAAAGAGGGAGTAA--AACAGTATGGACCCAACTCCCCTTATATGAGGACATTATTAGATTCCATTGCTCATGGACAT---AGACTCA
20_078    TTTTCTATAAAAATGCT-AAAAGACATGAAAGAGGGAGTAA--AACAGTATGGACCCAATTCTCCTTATATGAGAACATTATTAGATTCCATTGCTCATGGAAAT---AGACTTA
21_019    TTTTCGATAAAAATGCT-AAAAGATATGAAAGAAGGAGTAA--AACAGTATGGACCCAACTCCCCTTATATGAGGACATTATTAGATTCCATTGCTCATGGACAT---AGACTCA
22_012    TTTTCGATAAAAATGCT-AAAAGATATGAAAGAGGGAGTAA--AACAGTATGGACCCAACTCCCCTTATATGAGGACATTATTAGATTCCATTGCTTATGGACAT---AGACTCA
22_041    TTCACCATGAAAATGTT-AAAAGATATAAAGGAAGGAGTTA--AACAATATGGATCCAACTCCCCTTATATAAGAACATTATTAGATTCCATTGCTCATGGAAAT---AGACTTA
2_Tan     TTTTCGATAAAAAAGCT-AAAAGATATGAAAGAGGGAGTAA--AACAGTATGGACCCAACTCCCCTTATATGAGGACATTATTAGATTCCATTGCTCATGGACAT---AGACTCA
3_014     TTTTCTATAAAAATGCT-AAAAGACATGAAAGAGGGAATAA--AACAGTATGGACCCAACTCCCCTTATATGAGGACATTATTAGATTCCATTGCACACGGACAT---AGACTCA
3_209     TTCACCATGAAAATGTT-AAAAAATTTAAAGGAAGGATTTA--AACAATATGGACCCAACTGTCCTTATATGAGAACATTATTAGATTCCATTGCTCATGGAAAT---AGACTTA
3_294     TTCACCATGAAAATGTT-AAAAGATATAAAGGAAGGAGTTA--AACAATATGGATCCAACTCCCCTTATATAAGAACATTATTAGATTCCATTGCTCATGGAAAT---AGACTTA
3_354     TTTTCGATAAAAATGCT-AAAAGATATGAAAGAGGGAGTAA--AACAGTATGGACCCAACTCCCCTTATATGAGGACATTATTAGATTCCATTGCTCATGGACAT---AAACTCA
3_385     TTTTCGATAAAAATGCT-AAAAGATATGAAAGAGGGAGTAA--AACAGTATGGACCCAACTCCCCTTATATGAGGACATTATTAGATTCCATTGCTCATGGACAT---AGACTCA
3_496     TTTTCGATAAAAATACT-AAAAGATATGAAAGAGGGAGTAA--AACAGTATGGACCCAACTCCCCTTATATGAGGACATTATTAGATTCCATTGCTCATGGACAT---AGACTCA
3_610     TTTTCGATAAAAATGCT-AAAAGATATGAAAGAGGGAGTAA--AACAGTATGGACCCAACTCCCCTTATATGAGGACATTATTAGATTCCATTGCTCATGGACAT---AGACTCA
4_008     TTCACCTTGAAAATGTT-AAAAGATATGAAGAAAGGAGTTATAAACAATATGGACCCAACTCCCCTTACATGAGAACATTATTAGATTCCATTGTTCATGGAAAT---AGACTTA
4_032     TTCACCACAAAAATGTT-AAAAGATATGAAGGAAGGAGTTA--AACAATATGGACCTAACTCTCCTTATATGAGAACATTATTAGATTCCATTGCTCATGGAAAT---AGACTTA
4_052     ATCACCATAAAAAAATTTAAAAGATATGAAGGAAGGAGTTA--AACAATATGGACCTAACTCTCCTTATATGAGAACACTATTAGATTCCATTGCTCATGGAAAT---AGACTTA
4_068     TTCACCACAAAAATGTT-AAAAGATATGAAGGAAGGAGTTA--AACAATATGGACCTAACTCTCCTTATATGAGAACATTATTAGATTCCATTGCTCATGGAAAT---AGACTTA
4_716     TTTTCGATAAAAATGCT-AAAAGATATGAAAGAGGGAGTAA--AACAGTATGGACCCAACTCCCCTTATATGAGGACATTATTAGATTCCATTGCTCATAGACAT---AGACTCA
4_742     TTTTCTATAAAAATGCT-AAAAGATAGGAAAGACGGAGTCA--AACAGTATGGACCCAACTCCCCTTACATGAGGACATTATTAGATTCCATTGCTCATGGACAT---AGACTCA
5_147     TTTTCGATAAAAATGCT-AAAAGATATGAAAGAGGGAGTAA--AACAGTATGGACCCAACTCCCCTTATATGAGGACATTATTAGATTCCATTGCTCATGGACAT---AGACTCA
5_259     TTTTCTATAAAAATGCT-GAAAGATATGAAAGAGGGAGTAA--AACAGTATGGACCCAACTTCCCTTATATGAGGACATTATTAGACTCCATTGCTCATGGTCAT---AGACTCA
5_535     TTCACCCTGAAAATGTT-AAAAGATATGAAGGAAGGAGTTA--AACAATATGGACCCAACTCTCCTTATATGAGAACATTATTAGATTCCATTGCTCATGGAAAT---AGACTTA
5_544     TTTTCGATAAAAATGCT-AAAAGATATGAAAGAGGGAGTAA--AACAGTATGGACCCAACTCCCCTTATATGAGGACATTATTAGATTCCATTGCTTATGGACAT---AGACTCA
6_098     TTTTCTATAAAAATGCT-AAAAGATATGAAAGAGGGAGTAA--AACAGTATGGACCCAGCTCCCCTTATATGAAAATATTATTAGATTCCATTGCTCATGGACAG---AGACTCA
6_178     TTCACCATGAAAATGTT-AAAAGATATGAAGGAAGGAGTT------AATATGGACCCAACTCCCCTTATGTGAGAACATTATTAGATTCCATTGTTCATGGAAAT---AGACTTA
6_240     TTGCCCAACCTACAGCT-----CACTACAATGAAGGAGCAA--TTCAGACTCACCCTGCAGTGTCCTGTATGGGTCAAACAGTGG---CCGCTCTCTAAGGAAAAGTTGGGTGTG
6_324     TTTTCGATAAAAATACT-AAAAGATATGAAAGAGGGAGTAA--AACAGTATGGACCCAACTCCCCTTATATGAGGACATTATTAGATTCCATTGCTCATGGACAT---AGACTCA
7_345     TTTTCGATAAAAATGCT-AAAAGATATGAAAGAGAGAGTAA--AACAGTATGGACCCAACTCCCCTTATATGAGGACATTATTAGATTCCATTGCTCATGGACAT---AGACTCA
7_450     TTTTCTATAAAAATGCT-AAAAGATATGAAAGAGGGAGTAA--AACAGTATGGACCCAACTCCCCTTATATGAGGACATTATTAGATTCCATTGCTCATGGACAT---AGACTCA
8_047     ----------------------------------GGAGTTA--AACAATATGGACCTAACTCTCCTTATATGAGAACATTATTAATTCCATTGCTCATGGAAAT---AGACTTA
8_074     ----------------------------------GGAGTTA--AACAATATGGACCTAACTCTCCTTATACGAGAATATTATTAATTCCATTGCTCATGGAAAT---AGACTTA
8_094     ----------------------------------GGAGTTA--AACAATATGGACCTAACTCTCCTTATATGAGAATATTATTAATTCCATTGCTCATGGAAAT---AGACTTA
8_121     ------------------------------------------GTCAGTAAGGTCCCAATTCGCCTTCTGTCCAGTCACTATTACACTCAGTAACTCAGGACAAG---CGATTGG
8_235     TTGCCCAACCTGCAGTT-----CACTATGGTGAAGGAGCAA--TTCAGACTCACCCTGCAGTGTCTTGTATGGGTCAAACAGTGA---CCACTCCCTAAGGAAAAGTTGGGGGCG
8_525     TTTTCGATAAAAATGCT-AAAAGATATGAAAGAGGGAGTAA--AACAGTATGGACCCAACTCCCCTTATATGAGGACATTATTAGATTCCATTGCTCATGGACAT---AGACTCA
8_559     TTCACCATGAAAATGTT-AAAAGACATAAAGGAAGGAGCTA--AACAATATGGACCCAACTCTCCTTATATGAGAACGTTATTAGATTCCATTGCTCATGGAAAT---AGACTTA
9_307     TTGCCCAACCTACAGTT-----CACTACAGTGAAGGAGCAA--TTCAGACTCGCCCTGCAGTGTCCTGTATGGGTCAAACAGTGG---CCACTCTCTAAGGAAAAGTTGGGGGCG
9_340     TTTTCTATGAAAATGCT-AAAAGACATGAAAGAGGGAGTAA--AACAGTATGGACCCAACTCCCCTTATATAAGAACATTATTAGATTCCACTGCTCATGGACAT---AGACTCA
HML2_Rep  TTTTCGATAAAAAAGCT-AAAAGATATGAAAGAGGGAGTAA--AACAGTATGGACCCAACTCCCCTTATATGAGGACATTATTAGATTCCATTGCTCATGGACAT---AGACTCA
K113      TTTTCGATAAAAATGCT-AAAAGATATGAAAGAGGGAGTAA--AACAGTATGGACCCAACTCCCCTTATATGAGGACATTATTAGATTCCATTGCTCATGGACAT---AGACTCA
K115      TTTTCGATAAAAATGCT-AAAAGATATGAAAGAGGGAGTAA--AACAGTATGGACCCAACTCCCCTTATATGAGGACATTATTAGATTCCATTGCTCATGGACAT---AGACTCA
X_540     TTGCCCAACCGGCAGTT-----CACTGCGGTGAAGGAGCAA--TTCAGACTCGCCCTGTAGCATCCTGTCTGGGTCAAACAGTGG---CCGCTCCCTAAGGAAAAGTTAGGGGCG
Y_026     TTCACCATGAAAATGTT-AAAAGATATGAAGGAAGGAGTTA--AACAATATGGACCCAACTCTCCTTGTATGAGAACATTATTAGATTCCATTGCTCTTGGAAAT---AGACTTA
94        TTTTCTATAAAAATGCT-AAAAGATATGAAAGAGGGAGTAA--AACAGTATGGACCCAACTCCCCTTATATGAGTACATTATTAGATTCCATTGCTCATGGACAT---AGACTCA
```

```
          695  700  705  710  715  720  725  730  735  740  745  750  755  760  765  770  775  780  785  790  795  800  805
10_019    TTCC-TTA--TGATTGGGAGA-TTCTGGCAAAATCGTCTCT-CTCACCCTC-TCAATTTTTACAATTTAAGACTTGGTG-----------GATTGATGGG-----GCACAAGAAC
10_310    TTCC-TTA--TGATTGGGAGA-TTCTGGCAAAATCGTCTCT-CTCACCCTC-TCAATTTTTACAATTTAAGACTTGGTG-----------GATTGATGGG-----GTACAAGAAC
11_233    CTACATAAAGTAGTTTAAAAA-CTATTGTAAAAAGGACATGTTTCACCAATG---------------------------------------------------------------
11_277    TTCC-TTA--TGATTGGGAGA-TTTTGGCCAAATCATCACT-CTCACTGTC-TCAATTTTTACAATTTAAGAGTTGGTG-----------GATTGATGGG-----GCACAAGAAC
11_424    TTCC-TTA--TGATTGGGAGA-TTCTGGCAAAATCGTCTCT-CTCACCCTC-TCAATTTTTACAATTTAAGACTTGGTG-----------GATTGATGGG-----GTACAAGAAC
11_456    TTCC-TTA--TGATTGGGAGA-TTCTGGCAAGATCATCTCT-CTCACCCTC-TCAATTTTTACAATTTAAGACTTGGTG-----------GATTGATGGG-----GTACAAGAAC
12_216    TTCC-TTA--TGATTGGGAGA-TTCTGGCAAAATCGTCTCT-CTTACCCTC-TCAATTTTTACAATTTAAGACTTGGTG-----------GATTGATGGG-----GTACAAGAAC
12_419    TTACATAAAATAGTTAAAAAA-CTATTTTAAATAGGATGTGTTCCACCCACT---------------------------------------------------------------
14_023    TTCC-TTA--TGATTGGGAAA-TTTTGGCTAAATCTTCTCT-TTCACCCTC-TCAGTATCTACAATTTAAAACCTGGTG-----------GATTGACGGA-----GTACAAGAAC
16_010    -------------------------------------------------------------------------------------------------------------------
17_029    CTCC-GTG--TGATTGGGAGA-TTTTAACGAAAGTTACACT-TTCGCCCTC-CCAATTTCTTCAGTTTAAGACTTGGTG-----------GACCGACGAG-----GCTCAAAATC
19_157    TTCC-TTT--TGATTGGGAGA-TTTTGGCCAAATCATCACT-CTCACCCTC-T-ATCTTTTACAATTTAAGACTTTGTG-----------GATTGATGGG-----GTACAAGAAC
19_238    TTCC-TTA--TGATTGGGAGA-TTCTGGCAAAATCGTCTCT-CTCACCCTC-TCAATTTTTACAATTTAAGACTTGGTG-----------GATTGATGGG-----GCACAAGAAC
19_352    TTCC-TTA--TGATTGGGAGA-TTCTGGCAAAATCGTCTCT-CTCACCCTC-TCAATTTTTACAATTTAAGACTTGGTG-----------GATTGATGGG-----GTACAAGAAC
19_407    TTCT-TTA--TGATTGGGAGA-TTTTGGCCAAATGATCGCT-CTCACCCTC-TCAATGTTTACAATTTAAGACTTGGTGAAGACTTGGTGGATTGATGGG-----GCACAAGAAC
19_639    CTACATAAAATAGTTAAAAAA-CTACTTAAAAA-GGACACGTTTCACCCACT---------------------------------------------------------------
1_059     TTCC-CTA--TGATTGGGAAA-CGTTGGCTAAATCTTCCCT-TTCACCCTC-TCAGTTCCTACAATTTAAAACCTGGTG-----------GATTGATGGA-----GTACAAGAAC
1_061     TTCC-CTA--TGATTGGGAAA-TGTTGGCTAAATCTTCCCT-TTCACCCTC-TCAGTTCCTACAATTTAAAACCTGGTG-----------GATTGATGGA-----GTACAAGAAC
1_259     TTCC-TTA--TGATTGGGAGA-TTCTGGCAAAATCGTCTCT-CTCACCCTC-TCAATTTTTACAATTTAAGACTTGGTG-----------GATTGATGGG-----GTACAAGAAC
1_504     TTCC-TTA--TGATTGGGAGA-TTCTGGCAAAATCGTCTCT-CTCACCCTC-TCAATTTTTACAATTTAAGACTTGGTG-----------GATTGATGGG-----GTACAAGAAC
1_555     TTCC-TTA--TGATTGGGAGA-TTCTGGCAAAATCATCTCT-CTCACCCTC-TCAATTTTTACAATTTAAGACTTGGTG----------AATTGATGGG-----GCACAAGAAC
20_078    TTCC-TTA--TGACTGGGAAA-TTTTGGCCAAATCATCACT-CTCACCTTC-TCAATTTTTACAATTTAAGACCTGGTG-----------GATTGATGGG-----GTACAACAAC
21_019    TTCC-TTA--TGATTAGGAGA-TTCTGGCAAAATCGTCTCT-CTCACCCTC-TCAATTTTTACAATTTAAGACTTAGTA-----------GATTGATGAG-----GTACAAGAAC
22_012    TTCC-TTA--TGATTGGGAGA-TTCTGGCAAAATCGTCTCT-CTCACCCTC-TCAATTTTTACAATTTAAGACTTGGTG-----------GATTGATGGG-----GTACAAGAAC
22_041    CTCC-TTA--TGACTGGGAAA-TTTTGGCCAAATCTTCCCT-TTCATCCTC-TCAGTATCTACAGTTTAAAACCTGGTG-----------GATTGATGGA-----GTACAAGAAC
2_Tan     TTCC-TTA--TGATTGGGAGA-TTCTGGCAAAATCGTCTCT-CTCACCCTC-TCAATTTTTACAATTTAAGACTTGGTG-----------GATTGATGGG-----GTACAAGAAC
3_014     TTCC-TTA--TGATTGGGAGA-TTCTGGCAAAATCGTCTCT-CTCACCCTC-TCAATTTTTACAATTTAAGATTTGGTG-----------GATTAATGGG-----GCACAAGAAC
3_209     TTCC-TTA--AGATTGGGAAA-TTTTGGTTAAATCTTCCCT-CTCACCCTC-TCAGTATCTACAGTTTAAAACCTGGTG-----------GATTGATGGG-----GTACAAGAAC
3_294     CTCC-TTA--TGACTGGGAAA-GTTTGGCCAAATCTTCCCT-TTCATCCTC-TCAGTATCTACAGTTTAAAACCTGGTG-----------GATTGATGGA-----GTACAAGAAC
3_354     TTCC-TTA--TGATTGGGAGA-TTCTGGCAAAATCGTCTCT-CTCACCCTC-TCAATTTTTACAATTTAAGACTTGGTG-----------GATTGATGGG-----GTACAAGAAC
3_385     TTCC-TTA--TGATTGGGAGA-TTCTGGCAAAATCGTCTCT-CTCACCCTC-TCAATTTTTACAATTTAAGACTTGGTG-----------GATTGATAGA-----GTACAAGAAC
3_496     TTCC-TTA--TGATTGGGAGA-TTCTGGCAAAATCGTCTCT-CTCACCCTC-TCAATTTTTACAATTTAAGACTTGGTG-----------GATTGATGGG-----GTACAAGAAC
3_610     TTCC-TTA--TGATTGGGAGA-TTCTGGCAAAATCGTCTCT-CTCACCCTC-TCAATTTTTACAATTTAAGACTTGGTG-----------GATTGATGGG-----GTACAAGAAC
4_008     TTCC-TTA--TGATTGGGAAA-TTTTGGCTAAATCTTCCCT-TTCACCCTC-TCA---TTTACAGTTTAAAACCTGGTG-----------GATTGATGGAATGGAGTACAAGAAC
4_032     TTCC-TTA--TAATTGGGAAA-TTTTGGCTAAATCTTCCCT-TTCACCCTC-TCAGTATCTACAGTTTAAAACCTGGTG-----------GATTGATGGG-----GTACAAGAAC
4_052     TTTC-TTA--TGATTGGGAAA-TTTTGGCTAAATCTTCCCT-TTCACCCTC-TCAGTATCTACAGTTTAAAACCTGGTG-----------GAATGATGGG-----GTACAAGAAC
4_068     TTCC-TTA--TAATTGGGAAA-TTTTGGCTAAATCTTCCCT-TTCACCCTC-TCAGTATCTACAGTTTAAAACCTGGTG-----------GATTGATGGG-----GTACATGAAC
4_716     TTCC-TTA--TGATTGGGAGA-TTCTGGCAAAATCGTCTCT-CTTACCCTC-TCAATTTTTACAATTTAAGACTTGGTG-----------GATTGATGGG-----GTACAAGAAC
4_742     TTCC-TTA--TGATTGGGAGA-TTCTGGCAAAATCGTCTTT-CTCACCCTC-TCAATTTGTACAATTTAAGACTTGGTG-----------GATTGATAGG-----ACACAAGAAC
5_147     TTCC-TTA--TGATTGGGAGA-TTCTGGCAAAATCGTCTCT-CTCACCCTC-TCTATTTTTACAATTTAAGACTTGGTG-----------GATTGATGGG-----GTACAAGAAC
5_259     TTCC-TTA--TGATTGGGAGA-TTTTGGCAAAATCGTCTTC--TCACCCTC-TCAATTTTTACAATTTAAGACTTGGTG-----------GATTGATGGG-----TAACAAGAAC
5_535     TTCC-TTA--TGATTGGGAAA-TTTTGGCTAAGTCTTCCCT-TTCACCCTC-TCAGTTTCTACAGTTTAAAACCTGGTG-----------GATTGATGGA-----GTACAAGAAC
5_544     TTCC-TTA--TGATTGGGAGA-TTCTGGCAAAATCGTCTCT-CTCACCCTC-TCAATTTTTACAATTTAAGACTTGGTG-----------GATTGATGGG-----GTACAAGAAC
6_098     TTCC-TTA--TGATTGGGAGA-TTTTGGCCAAATCATCACT-CTCATCCTC-TCAATTTTTACAATTTAAGACTTGGTG----------AATTGATGGG-----GCACAAGAAC
6_178     TTCC-TTA--TGACTGGGAAA-TTTTGGCTAAATCTTCCCT-TTCACCCTC-TCAGTTTCTACAGTTTAAACCGTGGTG-----------GATCGATGGA-----GTACAAGAAC
6_240     CTACATAAAATAGTTAAAAAA-CTATTTTAAAAAGGACATGTTTCACACACT---------------------------------------------------------------
6_324     TTCC-TTA--TGATTGGGAGA-TTCTGGCAAAATCGTCTCT-CTCACCCTC-TCAATTTTTACAATTTAAGACTTGGTG-----------GATTGATGGG-----GTACAAGAAC
7_345     TTCC-TTA--TGATTGGGAGA-TTCTGGCAAAATCGTCTCT-CTCACCCTC-TCAATTTTTACAATTTAAGACTTGGTG-----------GATTGATGGG-----GTTCAAGAAC
7_450     TTCC-TTA--TGATTGGGAGG-TTCTGGCCAAATTGTCTCT-CTCACCCTC-TCAATTTTAACAATTTAAGACTTGGTG-----------GATTGATGGG-----GCACAACAAC
8_047     TTTC-TTA--TGATTGGGAAA-TTCTGGCTAAATCTTCCCT-TTCACCCTC-TCAGTATCTCCAGTTTAAAACCTGGTG-----------GATTGATGGG-----GTACAAGAAC
8_074     TTTC-TTA--TGATTGGGAAA-TTCTGGCTATATCTTCCCT-TTCACCCTC-TCAGTATCTCCAGTTTAAAACCTGGTG-----------GATTGATGGG-----GTACAAGAAC
8_094     TTTC-TTA--TGATTGGGAAA-TTCTGGCTATATCTTCCCT-TTCACCCTC-TCAGTATCTCCAGTTTAAAACCTGGTG-----------GATTGATGGG-----GTACAAGAAC
8_121     CCCC-GGA--CGATTGGGAGA-TTTTAGCAAAAGCTACTCT-TTCGCCCTC-CCAATTTCTTCAATTTAAAAGTTGGTG-----------GACCGATGAG-----GCTCAAAATC
8_235     CTGCATAAAATAGTTTAAAAA-CTATTTTAAAAAGGACATGTTTCACTCACT---------------------------------------------------------------
8_525     TTCC-TTA--CAATTGGGAGA-TTCTGGAAAAATCGTCTCT-CTCACCCTC-TCAATTTTTACAATTTAAGACTTGGTG-----------GATTGATGGG-----GTACAAGAAC
8_559     TTCC-TTA--TGATTGGGAAA-TTTTACCTAAATCTTCCCT-TTCACCCTC-TCAGTATCTACAGTTTAAAACCTGGTG-----------GATTGATGGA-----GTACAAGAAC
9_307     CTACATAAAATAGTTAAAAAA-CTATTTAAAAAAGGACATGTTTCACCCACT---------------------------------------------------------------
9_340     TTCC-TTA--TGATTGGGAGA-TTTTGGCCAAATCATCACT-CTCACC----TCAATTTTTACAATTTAAGACCTGGTG-----------GATTGATGGG-----ACACAAGAAC
HML2_Rep  TTCC-TTA--TGATTGGGAGA-TTCTGGCAAAATCGTCTCT-CTCACCCTC-TCAATTTTTACAATTTAAGACTTGGTG-----------GATTGATGGG-----GTACAAGAAC
K113      TTCC-TTA--TGATTGGGAGA-TTCTGGCAAAATCGTCTCT-CTCACCCTC-TCAATTTTTACAATTTAAGACTTGGTG-----------GATTGATGGG-----GTACAAGAAC
K115      TTCC-TTA--TGATTGGGAGA-TTCTGGCAAAATCGTCTCT-CTCACCCTC-TCAATTTTTACAATTTAAGACTTGGTG-----------GATTGATGGG-----GTACAAGAAC
X_540     CTACATAAAATAGTTAAAAAAACTATTTAAAAAAGGACATGTTTCACCCACT---------------------------------------------------------------
Y_026     TTCC-TTA--GGATTGGGAAA-TTTTGGCTAAATCTTCCCT-TTCACCCTC-TCAATTCCTACAGTTTAAAACCTGGTA-----------GATTGATGGA-----GTACAAGAAT
94        TTCC-TTA--TGATTGGGAGA-TTCTGGCAAGATCATCTCT-CTCACCCTC-TCAATTTTTACAATTTAAGACTTGGTG-----------GATTGATGGG-----GTACAAGAAC
```

10_019	A
10_310	A
11_233	-
11_277	A
11_424	A
11_456	A
12_216	A
12_419	-
14_023	A
16_010	-
17_029	A
19_157	A
19_238	A
19_352	A
19_407	A
19_639	-
1_059	A
1_061	A
1_259	A
1_504	A
1_555	A
20_078	A
21_019	G
22_012	A
22_041	A
2_Tan	A
3_014	A
3_209	A
3_294	A
3_354	A
3_385	A
3_496	A
3_610	A
4_008	A
4_032	A
4_052	A
4_068	A
4_716	A
4_742	A
5_147	A
5_259	A
5_535	A
5_544	A
6_098	A
6_178	A
6_240	-
6_324	A
7_345	A
7_450	A
8_047	A
8_074	A
8_094	A
8_121	A
8_235	-
8_525	A
8_559	A
9_307	-
9_340	A
HML2_Rep	A
K113	A
K115	A
X_540	-
Y_026	A
94	A

```
>1_059
A-TGGCCATCAACTCCTCCTCCAGTGGTTCAGATGCCTGTAACATTACAACCTCAAATGCAGGTTAGACAAGTGCAAACC
CCAAGAGAATATCAAGTAGAAAAGGATAGAGTCTCTATCCCAGCAATGCCAATTCAGATACAGTATCCACAATATCAGCT
GGTAGAAAATAAGACCCAACCACCGGTAGTTTATCAATACTGGCCGCCAGCTGAGCTTCAGTATGGGCTGTCTCC-GGAG
GTTCAATACAGACCTCAAGCTGTGTGTCCCGTGCCACATAGCAC----GGCACCTCACCAGCAACCCA-----GTGGTGT
---------------------------------------------------------TTAATCCTACATCACCACCTAGT
GGACAAGGTAGTACACTGCATGAAATCATTGATAAA----------GCCAGAAAACAGGGAGA--TCGTGAGGCATGGCA
GTTCCTTGTAATTTTACAACCAATACCAGCTGGGAAAGGG-----------GGGCAAGCTGGAGCGTCTGTCCAAACTGA
GGCTAGATATGAATCTTTCACCATGAAAATGTT-AAAAGATATGAAGGAAGGAGTTA--AACAATATGGACCCAACTCCC
CTTATATGAGAACTTTATTAGATTCCATTGCTCATGGAAAT---AGACTTATTCC-CTA--TGATTGGGAAA-CGTTGGC
TAAATCTTCCCT-TTCACCCTC-TCAGTTCCTACAATTTAAAACCTGGTG-----------GATTGATGGA-----GTAC
AAGAACA
>1_061
A-TGGCCATCAACTCCTCCTCCAGCGGTTCAGATGCCTGTAACATTACAACCTCAAATGCAGGTTAGACAAGTGCAAACC
CCAAGAGAATATCATGTAGAAAAGGATAGAGTCTCTATCCCGGCCATGCCAATTCAGATACAGTATCCACAATATCAGCT
GGTAGAAAATAAGACCCAACCACCGGTAGTTTATCAATACTGGCCGCCAGCTGAGCTTCAGTATGGGCCGTCTCC-GGAG
GTTCAATACAGACCTCAAGCTGTGTGTCCCGTGCCACACAGCAC----GGCACCTCACCAGCAACCCA-----GCGGTGT
---------------------------------------------------------TTAGTCCTACATCACCACCTAGT
GGACAAGGTAGTACACTGCATGAAATTATTGATAAA----------GCCAGAAAACAGGGAGA--TCTTGAGGCATGGCA
GTTCCTTGTAATTTTACAACCAATACCAGCTGGGAAAGGG-----------GGGCAAGCTGGAGCGTCTGTCCAAACTGA
GGCTAGATATGAATCTTTCACCATGAAAATGTT-AAAAGATATGAAGGAAGGAGTTA--AACAATATGGACCCAACTCCC
CTTATATGAGAACTTTATTAGATCCCATTGCTCATGGAAAT---AGACTTATTCC-CTA--TGATTGGGAAA-TGTTGGC
TAAATCTTCCCT-TTCACCCTC-TCAGTTCCTACAATTTAAAACCTGGTG-----------GATTGATGGA-----GTAC
AAGAACA
>1_259
A-GGCAC---AAGTCCTCTTCCAGCAGGTCAGGTGCCCGTAAGATTACAACCTCAAACGCAGGTTAA-------------
--------------------------------------------------------------------------------
---AGAAAATAAGACCCAACCGCCAGTAGCTTATCAATACTGGCCGCCGGCTGAACTTCAGTATCGGCCACCCCC-AGAA
AGTCAGTATGGATATCCAGGAATGCCCCCAGCACCACAGGGCAG----GGCGCCATACCCTCAGCTGCCCACTAGGAGAC
---------------------------------------------------------TTAATCCTATGGCACCACCTAGT
AGACAGGGTAGTGAATTACATGAAATTATTGATAAA----------TCAAGAAAGGAAGGAGA--TACTGAGGCATGGCA
ATTCCCAGTAACGTTAGAACCAATGCCACCTGGAGAAGGA-----------GCCCAAGAGGGAGAGCCTCCCACAGTTGA
GGCCAGATACAAGTCTTTTTCGATAAAAATGCT-AAAAGATATGAAAGAGGGAGTAA--AACAGTATGGACCCAACTCTC
CTTATATGAGGACATTATTAGATTCCATTGCTCATGGACAT---AGACTCATTCC-TTA--TGATTGGGAGA-TTCTGGC
AAAATCGTCTCT-CTCACCCTC-TCAATTTTTACAATTTAAGACTTGGTG-----------GATTGATGGG-----GTAC
AAGAACA
>1_504
A-GGCAC---AAGTCATCTTCCAGCAGGTCAGGTGCCCGTAACATTACAACCTCAAAAGCAGGTTAA-------------
--------------------------------------------------------------------------------
---AGAAAATAAGACCCAACCGCCAGTAGCCTATCAATACTGGCCTCCGGCTGAACTTCAGTATCGGCCACCCCC-AGAA
AGTCAGTATGGATATCCAGGAATGCCCCCAGCACCACAGGGCAG----GGCGCCATACCCTCAGCCGCCCACTAGGAGAC
---------------------------------------------------------TTAATCCTACGGCACCACCTAGT
AGACAGGGTAGTGAATTACATGAAATTATTGATAAA----------TCAAGAAAGGAAGGAGA--TACTGAGGCATGGCA
ATTCCCAGTAACGTTAGAACCGATGCCACCTGGAGAAGGA-----------GCCCAAGAGGGAGAGCCTCCCACAGTTGA
GGCCAGATACAAGTCTTTTTCGATAAAAATGCT-AAAAGATATGAAAGAGGGAGTAA--AACAGTATGGACCCAACTCCC
CTTATATGAGGACATTATTAGATTCCATTGCTCATGGACAT---AGACTCATTCC-TTA--TGATTGGGAGA-TTCTGGC
AAAATCGTCTCT-CTCACCCTC-TCAATTTTTACAATTTAAGACTTGGTG-----------GATTGATGGG-----GTAC
AAGAACA
>1_555
A-GGGCC---AAGTCCTCTTTCAGCAGGTCAGGTGACCGTAACATTACAACCTCAAGCGCAGGTTAG-------------
--------------------------------------------------------------------------------
---AGAAAATAAGACCCAACTGCCAGTAGCTTATCAATACTGGCCACCGGCCGAACTTCAGTATCGGCCACCCCC-AGAA
AGTCAGTATGGATATCTAGGAATGCCCCCAGCACCACAGGGCAG----GGAGCCATACCCTCAGCCGCCCACTAGGAGAC
-----------------------------------------------------------AATCCTATGGCACCACCTAGT
AGACAGGGTAGTGAATTACATGAAATTATTGAGAAG----------TCAAGAAAGGAAGGAGA--TACTGAGGCGTGGCA
ATTCCCAGTAACGTTAGAACCGATGCCACCTGGAGAAGGA-----------GCCCAAGAGGGAGAGCCTCTCACAGTTGA
GGCCAGATACAAGTCTTTTTAGATAAAAATGCT-AAAAGATATGAAAGAGGGAGTAA--AACAGTATGGACCCAACTCCC
CTTATATGAGGACATTATTAGATTCCATTGCTCATGGACAT---AGACTCATTCC-TTA--TGATTGGGAGA-TTCTGGC
AAAATCATCTCT-CTCACCCTC-TCAATTTTTACAATTTAAGACTTGGTG-----------AATTGATGGG-----GCAC
AAGAACA
>2_Tan
A-GGCAC---AAGTCCTCTTCCAGCAGGTCAGGTGCCTGTAACATTACAACCTCAAAAGCAGGTTAA-------------
--------------------------------------------------------------------------------
---AGAAAATAAGACCCAACCGCCAGTAGCCTATCAATACTGGCCTCCGGCTGAACTTCAGTATCGGCCACCCCC-AGAA
AGTCAGTATGGATATCCAGGAATGCCCCCAGCACCACAGGGCAG----GGCGCCATACCCTCAGCCGCCCACTAGGAGAC
---------------------------------------------------------TTAATCCTACGGCACCACCTAGT
AGACAGGGTAGTAAATTACATGAAATTATTGATAAA----------TCAAGAAAGGAAGGAGA--TACTGAGGCATGGCA
ATTCCCAGTAACGTTAGAACCGATGCCACCTGGAGAAGGA-----------GCCCAAGAGGGAGAGCCTCCCACAGTTGA
GGCCAGATACAAGTCTTTTTCGATAAAAAAGCT-AAAAGATATGAAAGAGGGAGTAA--AACAGTATGGACCCAACTCCC
CTTATATGAGGACATTATTAGATTCCATTGCTCATGGACAT---AGACTCATTCC-TTA--TGATTGGGAGA-TTCTGGC
AAAATCGTCTCT-CTCACCCTC-TCAATTTTTACAATTTAAGACTTGGTG-----------GATTGATGGG-----GTAC
AAGAACA
>3_014
A-GGGAC---AAGTCCTCTTCCAGCAGGTCAGATGCCCGTAACATTACAACCTCAAACACAGGTTAG-------------
--------------------------------------------------------------------------------
---AGAAAATAAGACCCAACCACCAGTAGCTTATCAATACTGGCTGCCGGCTGAATTTCAGTATCGGCCACCCCC-AGAA
```

AGTCAGTATGGACATCCAGGAATGCTCCCAGCACCACAGGGCAG----GGCGCCATACCCTCAGCCGCCCACTATGAGAC
---TTAATCCTACAGCACCACCTAAT
AGACAGGGTAGTGCATTACATAAAATTATTGATAAG----------TCAAGAAAGCAAGGAGA--TATTGAGGCGTGGCC
ATTCCCACTAATGTTAGAACTGATACCACCTGGAGAAGGA-----------GCCCAAGAGGGAGAGCCTCCCACAGTTGA
GGCCAGATACAAGTCTTTTTCTATAAAAATGCT-AAAAGACATGAAAGAGGGAATAA--AACAGTATGGACCCAACTCCC
CTTATATGAGGACATTATTAGATTCCATTGCACACGGACAT---AGACTCATTCC-TTA--TGATTGGGAGA-TTCTGGC
AAAATCGTCTCT-CTCACCCTC-TCAATTTTTACAATTTAAGATTTGGTG-----------GATTAATGGG-----GCAC
AAGAACA
>3_209
A-TGGCCACCATCTCCTTCTCCCGCGGTTCAGATACCTGTGACATCACAATCTCAAA-----------------------
--TGCTAATCCAGGCACAGTATCCACAATAGCAGGC
AGTAGAAAATAAAACCCAACCATCGGTAGTTTATCAACACCAGCCGCCAGCCGAATTTCAGTATCGGCCGTCTCC-AGAG
GTTCAGTATGGATCTCAGGTGGTGCGTCCTGTGCTAAATAGCAA----GACACTATATCAACAACCCACGGCGATGGCGT
---TTGATCCTACAGCACCACCTAGT
GGACAAGATGGTGCACTGCATGAGACCATTGCTACA----------GCCAGAAAACAGGGAGA--TCTTGAGGCATGGCA
GTATCCGGTAATTTTACAACCCATACCGGCCAGAAAAGGG-----------AGTCAAACAGGAGCATCTGTCTGAACTGA
GGCTAGATATGAATCTTTCACCATGAAAATGTT-AAAAAATTTAAAGGAAGGATTTA--AACAATATGGACCCAACTGTC
CTTATATGAGAACATTATTAGATTCCATTGCTCATGGAAAT---AGACTTATTCC-TTA--AGATTGGGAAA-TTTTGGT
TAAATCTTCCCT-CTCACCCTC-TCAGTATCTACAGTTTAAAACCTGGTG-----------GATTGATGGG-----GTAC
AAGAACA
>3_294
A-GGGCC---AAGTCCTCTTCCAGCAGGTCAGGTGCCCGTAACATTACAACCTCAAACGCAGGTTAA-------------
----
---AGAAAATAAGACCCAACCGCCAGTAGCTTATCAATACTGGCCGCCGGCTGAACTTCAGTATCTGCCACCCCC-AGAA
AGTCAGTATGGATATCCAGGAATGCCCCCAGCACTACAGGGCAG----GGCGCCATATCCTCAGCCGCCCACTGTGAGAC
---TTAATCCTACAGCATCACGTAGT
GGACAAGGTGGTACACTGCACGCAGTCATTGATGAA----------GCCAGAAAACAGGGAGA--TCTTGAGGCATGGCG
GTTCCTGGTAATTTTACAACTGGTACAGGCCGGGGAAGAG-----------ACTCAAGTAGGAGCGCCTGCCCGAGCTGA
GACTAGATGTGAACCTTTCACCATGAAAATGTT-AAAAGATATAAAGGAAGGAGTTA--AACAATATGGATCCAACTCCC
CTTATATAAGAACATTATTAGATTCCATTGCTCATGGAAAT---AGACTTACTCC-TTA--TGACTGGGAAA-GTTTGGC
CAAATCTTCCCT-TTCATCCTC-TCAGTATCTACAGTTTAAAACCTGGTG-----------GATTGATGGA-----GTAC
AAGAACA
>3_354
A-GGCAC---AAGTCATCTTCCAGCAGGTCAGGTGCCCGTAACATTACAACCTCAAAAGCAGGTTAA-------------
----
---AGAAAATAAGACCCAACCGCCAGTAGCCTATCAATACTGGCCGCCGGCTGAACTTCAGTATCGGCCACCCCC-AGAA
AGTCAGTATGGATATCCAGGAATGCCCCCAGCACCACAGGGCAG----GGCGCCATACCCTCAGCCACCCACTAGGAGAC
---TTAATCCTACGGCACCACCTAGT
AGACGGGGTAGTGAATTACATGAAATTATTGATAAA----------TCAAGAAAGGAAGGAGA--TACTGAGGCATGGCA
ATTCCCAGTAACGTTAGAACCGATGCCACCTGGAGAAGGA-----------GCCCAAGAGGGAGAGCCTCCCACAGTTGA
GGCCAGATACAAGTCTTTTTCGATAAAAATGCT-AAAAGATATGAAAGAGGGAGTAA--AACAGTATGGACCCAACTCCC
CTTATATGAGGACATTATTAGATTCCATTGCTCATGGACAT---AAACTCATTCC-TTA--TGATTGGGAGA-TTCTGGC
AAAATCGTCTCT-CTCACCCTC-TCAATTTTTACAATTTAAGACTTGGTG-----------GATTGATGGG-----GTAC
AAGAACA
>3_385
A-GGCAC---AAGTCCTCTTCCAGCAGGTCAGGTGCCTGTAACATTACAACCTCAAATGCAGGTTAA-------------
----
---AGAAAATAAGACCCAACCGCCAGAAGCTTATCAATACTGGCCGCCGGCTGAACTTCAGTATCGGCCACCCCC-AGAA
AGTCAGTATGGATATCCAGGAATGCCCCCAGCACCACAGGGCAG----GGCGCCATACCCTCAGCTGCCCACTAGGAGAC
---TTAATCCTATGGCACCACCTAGT
AGACAGGGTAGTGAATTACATGAAATTATTGATAAA----------TCAAGAAAGGAAGGAGA--TACTGAGGCGTGGCA
ATTCCCAGTAACATTAGAACCGATGCCAACTGGAGAAGGA-----------GCCCAAGAGGGAGAGCCTCCCACAGTTGA
GGCCAGATACAAGTCTTTTTCGATAAAAATGCT-AAAAGATATGAAAGAGGGAGTAA--AACAGTATGGACCCAACTCCC
CTTATATGAGGACATTATTAGATTCCATTGCTCATGGACAT---AGACTCATTCC-TTA--TGATTGGGAGA-TTCTGGC
AAAATCGTCTCT-CTCACCCTC-TCAATTTTTACAATTTAAGACTTGGTG-----------GATTGATAGA-----GTAC
AAGAACA
>3_496
A-GGGCC---AAGTCCTCTTCCAGCAGGTCAGGTGCCCGTAACATTACAACCTCAAAAGCAGATTAA-------------
----
---AGAAAATAAGACCCAACCGCCAGTAGCTTATCAATACTGGCCGCCGGCTGAACTTCAGTATCGGCCACCCCC-AGAA
AGTCAGTATGGATATCCAGGAATGCCCCCAGCACCACAGGGCAG----GGCGCCATACCCTCAGCCGCCCACTAGGAGAC
---TTAATCCTACGGCACCACCTAGT
AGACAGGGTAGTGAATTACATGAAATTATTGATAAA----------TCAAGAAAGGAAGGAGA--TACTGAGGCATGGCA
ATTCCCAGTAACGTTAGAACCGATGCCACCTGGAGAAGGA-----------GCCCAAGAGGGAGAGCCTCCCACAGTTGA
GGCCAGATACAAGTCTTTTTCGATAAAAATACT-AAAAGATATGAAAGAGGGAGTAA--AACAGTATGGACCCAACTCCC
CTTATATGAGGACATTATTAGATTCCATTGCTCATGGACAT---AGACTCATTCC-TTA--TGATTGGGAGA-TTCTGGC
AAAATCGTCTCT-CTCACCCTC-TCAATTTTTACAATTTAAGACTTGGTG-----------GATTGATGGG-----GTAC
AAGAACA
>3_610
A-GGCAC---AAGTCCTCTTCCAGCAGGTCAGGTGCCTGTAACATTACAACCTCAAACGCAGGTTAA-------------
----
---AGAAAATAAGACCCAACCGCCAGTAGCCTATCAATACTGGCCACCGGCTGAACTTCAGTATCGGCCACCCCC-AGAA
AGTCAGTATGGATATCCAGGAATGCCCCCAGCACCACAGGGCAG----GGCGCCATACCCTCAGCCGCCCACTAGGAGAC
---TTAATCCTACGGCACCACCTAGT
AGACAGGGTAGTGAATTACATGAAATTATTGATAAA----------TCAAGAAAGGAAGGAGA--TACTGAGGCATGGCA
ATTCCCAGTAACGTTAGAACCGATGCCACCTGGAGAAGGA-----------GCCCAAGAGGGAGAGCCTCTCACAGTTGA

```
GGCCAGATACAAGTCTTTTTCGATAAAAATGCT-AAAAGATATGAAAGAGGGAGTAA--AACAGTATGGACCCAACTCCC
CTTATATGAGGACATTATTAGATTCCATTGCTCATGGACAT---AGACTCATTCC-TTA--TGATTGGGAGA-TTCTGGC
AAAATCGTCTCT-CTCACCCTC-TCAATTTTTACAATTTAAGACTTGGTG-----------GATTGATGGG-----GTAC
AAGAACA
>4_008
--------------------------------------------------------------------------------
--------------------------------------------------------------------------------
--------------------------------------------------------------------------------
----------------------------------------------------------CAGCAACCCACGGCGGTGGCGC
---------------------------------------------------------TTAATCCTACAGCACCACCTAGT
GGACAATGTAGCACACTGTATGAAATCATTGATTAA----------GCCAGAAAACAGGGAGA--TCTTGAGGCATGGCA
GTTCCCAGTAATTTTACAACCGGTACCAGCCGGGGAAGAG-------------ACAAGTAGGAGTGCCTACCCAAGCTGA
GGCTAGATATGAATCTTTCACCTTGAAAATGTT-AAAAGATATGAAGAAAGGAGTTATAAACAATATGGACCCAACTCCC
CTTACATGAGAACATTATTAGATTCCATTGTTCATGGAAAT---AGACTTATTCC-TTA--TGATTGGGAAA-TTTTGGC
TAAATCTTCCCT-TTCACCCTC-TCA---TTTACAGTTTAAAACCTGGTG-----------GATTGATGGAATGGAGTAC
AAGAACA
>4_032
A-TGGCCACCAACCCCTTCTCCCGCGGTTCAGATGCCTGTGATGTCACAATCTCAAA-----------------------
----------------------------------------------TGCCAATCCAGGCACAGTATCCGCAATACCAGCC
CGTAGAAAATAAA-CCCAACCATCGGTAGTTTATCAACACCAGCCGCCAGCCGTATTTCAGTATCCGCCGTCTCC-AGAG
GTTCAGTATGGATCTCAGGCGGTGCGTCCTGTGCCAAATAGCAA----GGCACTATATCAACAACCCACGGCGATGGCGT
---------------------------------------------------------TTGATCTTACAGTACCACCTAGT
GGACAAGATAGTGCACTGCATGAGACCATTGCTACA----------GCCAGAAAACAGGGAGA--TCTTGAGGCATGGCA
ATATCCGGGAATGTTACAACCGATGCCGGCCGGGAAAGGG-----------AGTCAAGCAGGAGCGTCTGTCTGAACTGA
GACCAGATATGAATCTTTCACCACAAAAATGTT-AAAAGATATGAAGGAAGGAGTTA--AACAATATGGACCTAACTCTC
CTTATATGAGAACATTATTAGATTCCATTGCTCATGGAAAT---AGACTTATTCC-TTA--TAATTGGGAAA-TTTTGGC
TAAATCTTCCCT-TTCACCCTC-TCAGTATCTACAGTTTAAAACCTGGTG-----------GATTGATGGG-----GTAC
AAGAACA
>4_052
A-TGGCCACCAACTCCTTCTCCCGCGGTTCAGATGCCTTTGATGTCACAATCTCAAA-----------------------
----------------------------------------------TGCCAATCCAGGCACAGTATCCGCAATACCAGCC
AGTAGAAAATAAAACCCAACCATCGGTAGTTTATCAACACAAGCCGTCAGCCGTATTTCAGTATCCGCCGTCTCC-AGAG
GTTCAGTATGGATCTCAGGCGGTGCGTCCTGTGCCAAATAGCAA----GGCACTATATCAACAACCCAAGGCGATGGCGT
---------------------------------------------------------TTGATCTTACAGTACCACCTAGT
GGACAAGGTAGTGCACTGCATGAGACCATTGCTACA----------GCCAGAAAACAGGGAGA--TCTTGAGGCATGGCA
ATATCCGGGAATGTTACAAACAATGCCGGCCGGGAAAGGG-----------AGTCAAGCAAGAGCGTCTGTCTGAACCAA
GACTAGATATGAATCTATCACCATAAAAAAATTTAAAAGATATGAAGGAAGGAGTTA--AACAATATGGACCTAACTCTC
CTTATATGAGAACACTATTAGATTCCATTGCTCATGGAAAT---AGACTTATTTC-TTA--TGATTGGGAAA-TTTTGGC
TAAATCTTCCCT-TTCACCCTC-TCAGTATCTACAGTTTAAAACCTGGTG-----------GAATGATGGG-----GTAC
AAGAACA
>4_068
A-TGGCCACCAACCCCTTCTCCCGCGGTTCAGATGCCTGTGATGTCACAATCTCAAA-----------------------
----------------------------------------------TGCCAATCCAGGCACAGTATCCGCAATACCAGCC
AGTAGAAAATAAA-CCCAACCATCGGTAGTTTATCAACACCAGCCGCCAGCCGAATTTCAGTATCGGCCGTCTCC-AGAG
GTTCAGTATGGATCTCAGGAGGTGCGTCCTGTGCCAAATAGCAA----GGCACTATATCAACAACCCACGGCGATGGCGT
---------------------------------------------------------TTGATCTTACAGTACCACCTAGT
GGACAAGATAGTGCACTGCATGAGACCATTGCTACA----------GCCAGAAAACAGGGAGA--TCTTGAGGCATGGCA
ATATCTGGTAATGTTACAACCGATGCCGGCCGGGAAAGGG-----------AGTCAAGCAGGAGCGTCTGTCTGAACTGA
GACCAGATATGAATCTTTCACCACAAAAATGTT-AAAAGATATGAAGGAAGGAGTTA--AACAATATGGACCTAACTCTC
CTTATATGAGAACATTATTAGATTCCATTGCTCATGGAAAT---AGACTTATTCC-TTA--TAATTGGGAAA-TTTTGGC
TAAATCTTCCCT-TTCACCCTC-TCAGTATCTACAGTTTAAAACCTGGTG-----------GATTGATGGG-----GTAC
ATGAACA
>4_716
A-GGGCC---AAGTCCTCTTCCAGCAGGTCAGGTGCCCGTAACATTACAACCTCAAAAGCAGGTTAA-------------
--------------------------------------------------------------------------------
---AGAAAATAAGACCCAACCGCCAGTAGCTTATCAATACTGGCCGCCGGCTGAACTTCAGTATCGGCCACCCCT-AGAA
AGTCAGTGTGGATATCCAGGAATGCCCCCAGCACCACAGGGCAG----GGCGCCATACCCTCAGCCGCCCACTAGGAGAC
---------------------------------------------------------TTAATCCTACGGCACCACCTAGT
AGACAGGGTAGTGAATTACATGAAATTATTGATAAA----------TCAAGAAAGGAAGGAGA--TACTGAGGCATGGCA
ATTCCCAGTAACGTTAGAACCGATGCCACCTGGAGAAGGA-----------GCCCAAGAGGGAGAGCCTCCCACAGTTGA
GGCCAGATACAAGTCTTTTTCGATAAAAATGCT-AAAAGATATGAAAGAGGGAGTAA--AACAGTATGGACCCAACTCCC
CTTATATGAGGACATTATTAGATTCCATTGCTCATAGACAT---AGACTCATTCC-TTA--TGATTGGGAGA-TTCTGGC
AAAATCGTCTCT-CTTACCCTC-TCAATTTTTACAATTTAAGACTTGGTG-----------GATTGATGGG-----GTAC
AAGAACA
>4_742
A-GAGCC---AAGTCCTTTTCTAGCAGGTCAGATGCCCATAACATTACAACCTCAAACGCAGGTTAG-------------
--------------------------------------------------------------------------------
---AGAAAATAAGACCCAACCACCAGTAGCTTATCAATACTGGCCACTGGCTGAACTTCAGTATCAGCCACCCCC-AGAA
AGTCAGTATGGACATCCAGGAATGCTGCCAGCACTACAGAATAG----GGTGCCACACCCTCAGCCGCCCACTTTGAGAC
---------------------------------------------------------TTAATCCTATGGCACCACCTAGT
AGACAGGGTAGTGCATTACATAAAATTATTGTCAAGAAAATTATAGTCAAGAAAGCAAGGAGA--TACTGAGGTGTGGCA
ATTCCCAGTAATGTTGGAACTGATGCCACCTGGAGAAGGG-----------GCCCAAGAGGGAGAGCCTCCCACAGTTGA
GGCCAGATACAAGTCTTTTTCTATAAAAATGCT-AAAAGATAGGAAAGACGGAGTCA--AACAGTATGGACCCAACTCCC
CTTACATGAGGACATTATTAGATTCCATTGCTCATGGACAT---AGACTCATTCC-TTA--TGATTGGGAGA-TTCTGGC
AAAATCGTCTTT-CTCACCCTC-TCAATTTGTACAATTTAAGACTTGGTG-----------GATTGATAGG-----ACAC
AAGAACA
```

```
>5_147
A-GGCAC---AAGTCCTCTTCCAGCAGGTCAGGTGCCTGTAACATTACAACCTCAAACGCAGGTTAA-------------
--------------------------------------------------------------------------------
---AGAAAATAAGACCCAACCGCCAGTAGCCTATCAATACTGGCTGCCGGCTGAACTTCAGTATCGGCCACCCCC-AGAA
AGTCAGTATGGATATCCAGGAATACCCCCAGCACCACAGGGCAG----GGTGCCATACCCTCAGCCGCCCACTAGGAGAC
---------------------------------------------------------TTAATCCTATGGCACCACCTAGT
AGACAGGGTAGTGAATTACATGAAATTATTGATAAA----------TCAAGAAAGGAAGGAGA--TACTGAGGCATGGCA
ATTCCCAGTAACGTTAGAACCGATGCCACCTGGAGAAGGA-----------GCCCAAGAGGGAGAGCCTCTCACAGTTGA
GGCCAGATACAAGTCTTTTTCGATAAAAATGCT-AAAAGATATGAAAGAGGGAGTAA--AACAGTATGGACCCAACTCCC
CTTATATGAGGACATTATTAGATTCCATTGCTCATGGACAT---AGACTCATTCC-TTA--TGATTGGGAGA-TTCTGGC
AAAATCGTCTCT-CTCACCCTC-TCTATTTTTACAATTTAAGACTTGGTG-----------GATTGATGGG-----GTAC
AAGAACA
>5_259
A-GCACC---AAGCCCTCTTCCAGCAGGTGAGATGCCTGGAACATTACAGTCTCCAATGCAGGTTAG-------------
--------------------------------------------------------------------------------
---AGAAAATAAGACCCAACCACCAGTAGCTTAGCAATACTGGCCACTGGCCAAACTTCAGCATCAGCCATCCCC-AGAA
AGTCAGTATGGACATTCAGGAATGCTCCCAGCACCACAGGGCAG----GGAGCCATACCCTCAGCCACCCACTATGACAC
---------------------------------------------------------TTAATCCAACAGCACCACCTAGT
CAACAGGGTAGTGCGTTACATAAAATTATTGATAAT----------TCAAGAAACCAAGGAGA--TATTGAGGCATGGCA
ATTCCCAGTAACGTTAGAACCAATACCACCTGGAGAAGGG-----------GCCCAAGAGGGAGAGCCTCAAACAGTTGA
GGCCAGATATGAGTCTTTTTCTATAAAAATGCT-GAAAGATATGAAAGAGGGAGTAA--AACAGTATGGACCCAACTTCC
CTTATATGAGGACATTATTAGACTCCATTGCTCATGGTCAT---AGACTCATTCC-TTA--TGATTGGGAGA-TTTTGGC
AAAATCGTCTTC--TCACCCTC-TCAATTTTTACAATTTAAGACTTGGTG-----------GATTGATGGG-----TAAC
AAGAACA
>5_535
A-CGGCCATCAACTCCTCCTCCTGCGGTTCAGATGCCTGTGACATTACAACCTCAAATGCAGGTTAGACAAGTACAAACT
GCAAGAGAATATCAAGTAGAAAAGGATAGAGTCTCTATCCCGGCAATGCCAATCCAGACACAGTATCCACAATATCAGCT
GGTAGAAAATAAAACCCAACCACTGGTAGTTTATCAATACTGGCCGCCAGTCAAATTTCAGTATCAGCTGCTTCC-AGAG
GTTCAGTATAGATCTCAGGCGGGGTGTCCTGTCCCAAATAGCAG----GGCACTATATCAGCAACCCACAGCGATGGCGT
---------------------------------------------------------TTGATCCTACAGCACCAGCTAGT
GTACAAGATAGTACACTGCATAAAACCATT-----A----------GCCAGAAAACAGGGAGA--TCTTGAGGCATGGCA
GTACCCGGTAATTTTACAACCGATACTGGCTGGGAAAGGG-----------AGTCAAGCAGGAGCCTCTGTCCAAACTGA
GATTAGATATGAATCTTTCACCCTGAAAATGTT-AAAAGATATGAAGGAAGGAGTTA--AACAATATGGACCCAACTCTC
CTTATATGAGAACATTATTAGATTCCATTGCTCATGGAAAT---AGACTTATTCC-TTA--TGATTGGGAAA-TTTTGGC
TAAGTCTTCCCT-TTCACCCTC-TCAGTTTCTACAGTTTAAAACCTGGTG-----------GATTGATGGA-----GTAC
AAGAACA
>5_544
A-GGCAC---AAGTCCTCTTCCAGCAGGTCAGGTGCCCGTAAGATTACAACCTCAAAAGCAGGTTAA-------------
--------------------------------------------------------------------------------
---AGAAAATAAGACCCAACCGCCAGTAGCCTATCAATACTGGCCGCCGGCTGAACTTCAGTATCGGCCACCCCC-AGAA
AGTCAGTATGGATATCCAGGAATGCCCCCAGCACCACAGGGCAG----GGCGCCATACCATCAGCCGCCCACTAGGAGAC
---------------------------------------------------------TTAATCCTATGGCACCACCTAGT
AGACAGGGTAGTGAATTACATGAAATTATTGATAAA----------TCAAGAAAGGAAGGAGA--TACTGAGGCATGGCA
ATTCCCAGTAACGTTAGAACCGATGCCACCTGGAGAAGGA-----------GCCCAAGAGGGAGAGCCTCCCACAGTTGA
GGCCAGATACAAGTCTTTTTCGATAAAAATGCT-AAAAGATATGAAAGAGGGAGTAA--AACAGTATGGACCCAACTCCC
CTTATATGAGGACATTATTAGATTCCATTGCTTATGGACAT---AGACTCATTCC-TTA--TGATTGGGAGA-TTCTGGC
AAAATCGTCTCT-CTCACCCTC-TCAATTTTTACAATTTAAGACTTGGTG-----------GATTGATGGG-----GTAC
AAGAACA
>6_098
A-TGGCC---AACTCCTCTTCCAGCAGTTCAGATGCCTGTAACATTGCAACCTCAAATGCAGGTTAGACAAGTACAAACC
CCAGAAGAATATCAAATAGAAAAAGATAAAGTCTCTGCCATGGCAATGCCAATCCAGATACAGTATCCACAATATCAGCA
GGTAGAAAATAAGACCCAACTGCCAGTAGCCTATCAATACTGGCCGCCAGCCGAACTTCAGTATCGGCCGCCCCC-AGAA
AATCAGTATGGACACCCAGGAATGTTTCCAGCACCACAGGGCAG----GGCTCTAGATCCTCAGCCGTCCACTGTGAGAC
---------------------------------------------------------TTAATCCTACAGCACTACCTAGT
GGACAGCATAGTGCATTACATAAAATTATTGATAAG----------GCAAGAAAACAAGGAGA--TACTGAGGCGTGACA
ATTCCCAGTAACATTAGAACTGATACCACCTGGAGAAGGG-----------GCCCAAGAGGGAGTGCCTCCCCTAGCTGA
GGCCAGATATGAGTCCTTTTCTATAAAAATGCT-AAAAGATATGAAAGAGGGAGTAA--AACAGTATGGACCCAGCTCCC
CTTATATGAAAATATTATTAGATTCCATTGCTCATGGACAG---AGACTCATTCC-TTA--TGATTGGGAGA-TTTTGGC
CAAATCATCACT-CTCATCCTC-TCAATTTTTACAATTTAAGACTTGGTG-----------AATTGATGGG-----GCAC
AAGAACA
>6_178
A-TGGCCATCAA---CTCCTCCCGTGGTTCAGATACCTGTAACATTACAACCTCAAAGGGAGGTTAGACAAGTACAAACC
CCAAGAGAATATCAAGTAGAAAAGGATAGAGTCTCTATCCCGGCAATGCCAATTCAGATGCAGTATCCACAATATAAGCT
GGTAGAAAATAAGACCCAACCACCAGTAATTTATCAGTACTGGCCGCCAGCCGACCTTCAGTATCGGCCACCTCC-AGAG
GTTAAATACAGACCTCAAGTGGTGTGTCCTGTGCCAAATAGCAC----GGCACCATACCAGCAACCCACGGCAGTGGTGT
---------------------------------------------------------TTAATCCTACCGCACCACCTAGT
GGACAAGGTAGTACACTGCATGAAATTATTGATAAA----------GCCAGTAAACAGGGAGA--TCTTGAGGCATGGGA
GTTCCCGGTAATTTTACAACCGATACCGGCCGGGAAAGGG-----------ACTCTAGCAGGAGCGTCTGTCCAAACTGA
AGCTAGATATGAATCTTTCACCATGAAAATGTT-AAAAGATATGAAGGAAGGAGTT------AATATGGACCCAACTCCC
CTTATGTGAGAACATTATTAGATTCCATTGTTCATGGAAAT---AGACTTATTCC-TTA--TGACTGGGAAA-TTTTGGC
TAAATCTTCCCT-TTCACCCTC-TCAGTTTCTACAGTTTAAACCGTGGTG-----------GATCGATGGA-----GTAC
AAGAACA
>6_240
--------------------------------------------------------------------------------
--------------------------------------------------------------------------------
--------------------------------------------------------------------------------
```


--GGCCATT-GGGGC----TAAAACCACAATG
GCCACA--TCCTCCTCAGCCAAGTGATTGCTGGGGGAGGG-----------AGCCTGAAACTAGGCTCGCTGTGACTTGG
CTCGCAGCACTCATTATTGCCCAACCTACAGCT-----CACTACAATGAAGGAGCAA--TTCAGACTCACCCTGCAGTGT
CCTGTATGGGTCAAACAGTGG---CCGCTCTCTAAGGAAAAGTTGGGTGTGCTACATAAAATAGTTAAAAAA-CTATTTT
AAAAAGGACATGTTTCACACACT---

>6_324
A-GGCAC---AAGTCCTCTTCCAGCAGGTCAGGTGCCTGTAACATTACAACCTCAAAAGCAGGTTAA-------------
----
---AGAAAATAAGACCCAACCGCCAGTAGCCTATCAATACTGGCCTCCGGCTGAACTTCAGTATCGGCCACCCCC-AGAA
AGTCAGTATGGATATCCAGGAATGCCCCCAGCACCACAGGGCAG----GGCGCCATACCCTCAGCCGCCCACTAGGAGAC
---TTAATCCTACGGCACCACCTAGT
AGACAGGGTAGTGAATTACATGAAATTATTGATAAA----------TCAAGAAAGGAAGGAGA--TACTGAGGCATGGCA
ATTCCCAGTAACGTTAGAACCGATGCCACCTGGAGAAGGA-----------GCCCAAGAGGGAGAGCCTCCCACAGTTGA
GGCCAGATACAAGTCTTTTTCGATAAAAATACT-AAAAGATATGAAAGAGGGAGTAA--AACAGTATGGACCCAACTCCC
CTTATATGAGGACATTATTAGATTCCATTGCTCATGGACAT---AGACTCATTCC-TTA--TGATTGGGAGA-TTCTGGC
AAAATCGTCTCT-CTCACCCTC-TCAATTTTTACAATTTAAGACTTGGTG-----------GATTGATGGG-----GTAC
AAGAACA
>7_345
A-GGCAC---AAGTCCTCTTCCAGCAGGTCAGGTGCCTGTAACATTACAACCTCAAACGCAGGTTAA-------------
----
---AGAAAATAAGACCCAACCGCCAGTAGCCTATCAATACTGGCCGCCGGCTGAACTTCAGTATCGGCCACCCCC-AGAA
AGTTAGTATGGATATCCAGGAATGCCCCCAGCACCACAGGGCAG----GGCGCCATACCCTCAGCCGCCCACTAGGAGAC
---TTAATCCTACGGCACCACCTAGT
AGACAGGGTAGTGAATTACATGAAATTATTGATAAA----------TCAAGAAAGGAAGGAGA--TACTGAGGCATGGCA
ATTCCCAGTAACGTTAGAACCGATGCCACCTGGAGAAGGA-----------GCCCAAGAGGGAGAGCCTCTCACAGTTGA
GGCCAGATACAAGTCTTTTTCGATAAAAATGCT-AAAAGATATGAAAGAGAGAGTAA--AACAGTATGGACCCAACTCCC
CTTATATGAGGACATTATTAGATTCCATTGCTCATGGACAT---AGACTCATTCC-TTA--TGATTGGGAGA-TTCTGGC
AAAATCGTCTCT-CTCACCCTC-TCAATTTTTACAATTTAAGACTTGGTG-----------GATTGATGGG-----GTTC
AAGAACA
>7_450
A-GGGCC---AACTCCTCTTCCAGCAGTTCAGATGCCTGTAACATTACAACCTCAAATGCAGGTTAGGCAAGTACAAACC
CCAAGAGAATATCAAATAGAGAAAGATAGAGTCTCTGTCACGGCAATGCCAATCCAAATACAGTATCCACAATATCAGCC
GGTAGAAAATAAGACCCAACTGCCAGTAGCCTATCAATACTGGCTGCCAGCCGAACTTAAGTGTCGGCCACCCCC-GGAA
AATCAGTATGGACAGCCAGGAATGTTCCCAGCCCCACAGGACAG----GGCGCCATACCCTCAGCCACCCACTATGAGAC
---TTAATCCTACAGCACCACCTAGT
GAGCAGGGTAGTGTATTACATAAAATTATTGATGAG----------GCAAGAAAACAAAGAGA--TATTGAGGCATGGCA
ATTCCCAGTAATATTAGAACCAATACCACCTGGAGAAGGG-----------GCCCAAGAGGGAGAGCCTCCCCTAGCTGA
GGCCAGATATGAGTCTTTTTCTATAAAAATGCT-AAAAGATATGAAAGAGGGAGTAA--AACAGTATGGACCCAACTCCC
CTTATATGAGGACATTATTAGATTCCATTGCTCATGGACAT---AGACTCATTCC-TTA--TGATTGGGAGG-TTCTGGC
CAAATTGTCTCT-CTCACCCTC-TCAATTTTAACAATTTAAGACTTGGTG-----------GATTGATGGG-----GCAC
AACAACA
>8_047
A-TGGCCACCAACTCCTTCTCCCGCGGTTCAGATGCCTGTGATGTCACAATCTCAAA-----------------------
--TGCCAATCCAGGCACAGTATCCGCAATACCAGCC
AGTAGAAAATAAAACCCAACCATCGGTAGTTTATCAACACCAGCCGCCAGCCGCATTTCAGTATCCGCCGTCTCC-AGAG
GTTCAGTATGGATCTCAGGCGGTGCGTCCTGTGCCAAATAGCAA----GGCAGTATATCAACAACCCACGGCGATGGCGT
---TTGATCTTACGGTACCACCTAGT
GGACAAGATAGTGCACTGCATGAGACCATTGCTACA----------GCCAGAAAACAGGGAGA--TCTTGAGGCATGGCA
ATATCCGGTAATGTTACAACAGATGCCGGCCGGGAAAGGC-----------AGTCAAGCA--------------------
--GGAGTTA--AACAATATGGACCTAACTCTC
CTTATATGAGAACATTATTAAATTCCATTGCTCATGGAAAT---AGACTTATTTC-TTA--TGATTGGGAAA-TTCTGGC
TAAATCTTCCCT-TTCACCCTC-TCAGTATCTCCAGTTTAAAACCTGGTG-----------GATTGATGGG-----GTAC
AAGAACA
>8_074
A-TGGCCACCAACTCCTTCTCCCGCGGTTCAGATGCCTGTGATGTCACAATCTCAAA-----------------------
--TGCCAATCCAGGCACAGTATCCGCAATACCAGCC
AGTAGAAAATAAAACCCAACCATCGGTAGTTTATCAACACCAGCCGCCAGCCGCATTTCAGTATCCGCCGTCTCC-AGAG
GTTCAGTATGGATCTCAGGCGGTGCGTCCTGTGCCAAATAGCAA----GGCACTATATCAA---CCCACGGCGATGGCGT
---TTGATCTTACGGTACCACCTAGT
GGACAAGATAGTGCACTGCATGAGACCATTGCTACA----------GCCAGAAAACAGGGAGA--TCTTGAGGCATGGCA
ATATCCGGTAATGTTACAACCGATGCCGGCCGGGAAAGGG-----------AGTCAAGCA--------------------
--GGAGTTA--AACAATATGGACCTAACTCTC
CTTATACGAGAATATTATTAAATTCCATTGCTCATGGAAAT---AGACTTATTTC-TTA--TGATTGGGAAA-TTCTGGC
TATATCTTCCCT-TTCACCCTC-TCAGTATCTCCAGTTTAAAACCTGGTG-----------GATTGATGGG-----GTAC
AAGAACA
>8_094
A-TGGCCACCAACTCCTTCTCCCGCGGTTCAGATGCCTGTGATGTCACAATCTCAAA-----------------------
--TGCCAATCCAGGCACAGTATCCGCAATACCAGCC
AGTAGAAAATAAAACCCAACCATCGGTAGTTTATCAACACCAGCCGCCAGCCGCATTTCAGTATCCGCCGTCTCC-AGAG
GTTCAGTATGGATCTCAGGCGGTGCGTCCTGTGGCAAATAGCAA----GGCACTATATCAACAACCCACGGCGATGGCGT
---TTGATCTTACGGTACCACCTAGT
GGACAAGATAGTGCACTGCATGAGACCATTGCTACA----------GCCAGAAAACAGGGAGA--TCTTGAGGCATGGCA
ATATCCGGTAATGTTACAACAGATGCCGGCCGGGAAAGGC-----------AGTCAAGCA--------------------

```
--------------------------------------------------GGAGTTA--AACAATATGGACCTAACTCTC
CTTATATGAGAATATTATTAAATTCCATTGCTCATGGAAAT---AGACTTATTTC-TTA--TGATTGGGAAA-TTCTGGC
TATATCTTCCCT-TTCACCCTC-TCAGTATCTCCAGTTTAAAACCTGGTG-----------GATTGATGGG-----GTAC
AAGAACA
>8_121
--------------------------------------------------------------------------------
--------------------------------------------------------------------------------
--------------------------------------------------------------------------------
--------------------------------------------------------------------------------
--------------------------------------------------------------------------------
--------------------------------------------------------------------------------
--------------------------------------------------------------------------------
-----------------------------------------------------------GTCAGTAAGGTCCCAATTCGC
CTTCTGTCCAGTCACTATTACACTCAGTAACTCAGGACAAG---CGATTGGCCCC-GGA--CGATTGGGAGA-TTTTAGC
AAAAGCTACTCT-TTCGCCCTC-CCAATTTCTTCAATTTAAAAGTTGGTG-----------GACCGATGAG-----GCTC
AAAATCA
>8_235
--------------------------------------------------------------------------------
--------------------------------------------------------------------------------
--------------------------------------------------------------------------------
--------------------------------------------------------------------------------
--------------------------------------------------------------------------------
--------------------------------------------------GGCCTTT-GGGGC----TAAAACCACGATG
GCCACC--TCCTCCTCAGCTGAGTGAGTGCTGGGGGAGGG-----------AACCTGAAACTAGGCTCGCTGTGACTTGG
TTCACAGCACCCATTATTGCCCAACCTGCAGTT-----CACTATGGTGAAGGAGCAA--TTCAGACTCACCCTGCAGTGT
CTTGTATGGGTCAAACAGTGA---CCACTCCCTAAGGAAAAGTTGGGGGCGCTGCATAAAATAGTTTAAAAA-CTATTTT
AAAAAGGACATGTTTCACTCACT---------------------------------------------------------
-------
>8_525
A-GGCAC---AAGTCCTCTTCCAGCAGGTCAGGTGCCCGTAACATTACAACCTCAAAAGCAGGTTAA-------------
--------------------------------------------------------------------------------
---AGAAAATAAGACCCAACCACCAGTAGCCTATCAATACTGGCCGCCGGCTGAACTTCAGTATCGGCCACCCCC-AGAA
AGTCAGTATGGATATCCAGGAATGCCCCCAGCACCACAGGGCAG----GGCGCCATACCCTCAGCCGCCCACTAGGAGAC
---------------------------------------------------------TTAATCCTACGGCACCACCTAGT
AGACAGGGTAGTGAATTACATGAAATTATTGATAAA----------TCAAGAAAGGAAGGAGA--TACTGAGGCATGGCA
ATTCCCAGTAACGTTAGAACCGATGCCACCTGGAGAAGGA-----------GCCCAAGAGGGAGAGCCTCTCACAGTTGA
GGCCAGATACAAGTCTTTTTCGATAAAAATGCT-AAAAGATATGAAAGAGGGAGTAA--AACAGTATGGACCCAACTCCC
CTTATATGAGGACATTATTAGATTCCATTGCTCATGGACAT---AGACTCATTCC-TTA--CAATTGGGAGA-TTCTGGA
AAAATCGTCTCT-CTCACCCTC-TCAATTTTTACAATTTAAGACTTGGTG-----------GATTGATGGG-----GTAC
AAGAACA
>8_559
A-TGGCCACCAACTCCTTCTCCTGTGGTTCAGATGCCTGTGACATTACAACCTCAAA-----------------------
----------------------------------------------TGCCAATACAGACACAGTATCTACAATATCAGCT
GGTTGAAAATAAAACCCAACCACTGGTAGTTTATCAACACTGGCCGCCAGCCGAATTTCAGTATCGGCCATCTCC-AGAG
GTGCAGTATAGATCCCAGATGGGA--TCCTGTGCCAAATAGTAG----GGCACTATATCAACAACCCACGGCGATGGTGT
---------------------------------------------------------TTGATCCTACAGTACCAGCTAGT
GGACAAGATAGTGCACTGCATGAGACCGTTGATACA----------GCCAGAAAACAGGGAGA--TCTTGAGGCATGGCA
GTATCCGGTAATGTTACAACCGATACCGGCCGGGAAAGGGCCAGGAAAGGGAGTCAAGCAGGAGCGTCTGTCCGAACGGA
GGCTAGGTAAGAATATTTCACCATGAAAATGTT-AAAAGACATAAAGGAAGGAGCTA--AACAATATGGACCCAACTCTC
CTTATATGAGAACGTTATTAGATTCCATTGCTCATGGAAAT---AGACTTATTCC-TTA--TGATTGGGAAA-TTTTACC
TAAATCTTCCCT-TTCACCCTC-TCAGTATCTACAGTTTAAAACCTGGTG-----------GATTGATGGA-----GTAC
AAGAACA
>9_307
--------------------------------------------------------------------------------
--------------------------------------------------------------------------------
--------------------------------------------------------------------------------
--------------------------------------------------------------------------------
--------------------------------------------------------------------------------
--------------------------------------------------GGCCATT-GGGGC----TACAACCACAATG
GCCACC--TCCTCCTCAGCCGAGTGAGTGCTGGGGGAGGC-----------AGCCTGAAACTAGGCACACTGCGACTTGG
CTCGTGGCACTCATTATTGCCCAACCTACAGTT-----CACTACAGTGAAGGAGCAA--TTCAGACTCGCCCTGCAGTGT
CCTGTATGGGTCAAACAGTGG---CCACTCTCTAAGGAAAAGTTGGGGGCGCTACATAAAATAGTTAAAAAA-CTATTTA
AAAAAGGACATGTTTCACCCACT---------------------------------------------------------
-------
>9_340
A-TGGCCATCAACTCCTCCTCCCGCGGTTCAGATGCCTGTAACATTACAACCTCAAATGCAGGTTAGACAAGTACAAACC
CCAAGAGAATATCAAATAGAAAAGGAAAGAGTCTCTATCCCAGCAATGCCAATCCAGATGCAGTATCCACAATATCAGCT
GGTAGAAAATAAGACCCAACTGCCGGTAGCCTATCAATACTGGCCGCCAGCCAAACTTCAGTATCGGCCGCCCCC-AGAG
TATCAGTATGGATGGCCAGGA-TGTTTCCAGCACCGCAGGGCAG----GGCGCTATAGCCGCAGCCGCCCACTATGAGAC
---------------------------------------------------------GTAATCCTACAGCACCACCTAGT
GGACAGGGTCGTGCATTACATAAAATTATTGAAAAG----------ACA---AAACAAGGGGA--TACTGACGCGTGGCA
GACCCCGGTAATATTAGAACCCATACCACCTGGAAGAGGG-----------GCCCAAGAGGGAGCGCCTCCCCGAGCTGA
TGCCAGAAATGGGTCCTTTTCTATGAAAATGCT-AAAAGACATGAAAGAGGGAGTAA--AACAGTATGGACCCAACTCCC
CTTATATAAGAACATTATTAGATTCCACTGCTCATGGACAT---AGACTCATTCC-TTA--TGATTGGGAGA-TTTTGGC
CAAATCATCACT-CTCACC----TCAATTTTTACAATTTAAGACCTGGTG-----------GATTGATGGG-----ACAC
AAGAACA
```

>10_019
A-GGACC---AAGTCCTCTTCCAGTAGGTCAGATGCCCGTAACATTACAACCTCAAACGCAGGTTAG-------------
----
---AGAAAATAAGACCCAACTACCAGTAGCTTATCAATACTGGCCACCGGCCAAACTTCAGTATCGGCCACCCCC-AGAA
AGTCAGTATGGATATCCAGGAACGCTCCCAGCACCACAGGGCAG----GGCGCCATACCCTCAGCCACCCACTAGCAGAC
---TTAATCATACGGCACCACCTAGT
AGACAGGGTAGTGAATTACATGAAATTATTGATAAG----------TCAAGAAAGGAAGGAGA--CACTGAGGCGTGGCA
ATTCCCAGTAACGTTAGAACCGATACCACCTGGAGAAGGA-----------GCCCAAGAGGGAGAGCCTCTCACAGTTGA
GGCCAGATACAAGTCTTTTTCGATAAAAATGCT-AAAAGATATGAAAGAGGGAGTAA--AACACTATGGACCCAACTCCC
CTTATATGAGGACATTATTAGATTCCATTGCTCATGGACATCATAGACTCATTCC-TTA--TGATTGGGAGA-TTCTGGC
AAAATCGTCTCT-CTCACCCTC-TCAATTTTTACAATTTAAGACTTGGTG-----------GATTGATGGG-----GCAC
AAGAACA
>10_310
A-GGGCC---AAGTCCTCTTCCAGCAGGTCAGGTGCCCGTAACACTACAACCTCAAACGCAGGTTAA-------------
----
---AGAAAATAAGACCCAACCGCCAGTAGCTTATCAATACTGGCCGCCAGCCGAACTTCAGTATCGGCCACCCCC-AGAA
AGTCAGTATGGATATCCAGGAATGCCCCCAGCACCACAGGGCAG----GGCGCCATACCCTCAGCCGCCCACTAGGAGAC
---TTAATCCTACGGCACCACCTAGT
AGACAGGGTAGTGAATTACATGAAATTATTGATAAA----------TCAAGAAAGGAAGGAGA--TACTGAGGCGTGGCA
ATTCCCAGTAACGTTAGAACCGATGCCACCTGGAGAAGGA-----------GCCCAAGAGGGAGAGCCTCTCACAGTTGA
GGCCAGATACAAGTCTTTTTCGATAAAAATGCT-AAAAGATATGAAAGAGGGAGTAA--AACAGTATGGACCCAAATCCC
CTTATATGAGGACATTATTAGATTCCATTGCTCATGGACAT---AGACTCATTCC-TTA--TGATTGGGAGA-TTCTGGC
AAAATCGTCTCT-CTCACCCTC-TCAATTTTTACAATTTAAGACTTGGTG-----------GATTGATGGG-----GTAC
AAGAACA
>11_233

--GGCCATTTGGGGC----TAAAACCAGGATG
GCCACC--TCCTCCTCAGCCGAGTGAGTGCTGGAGGAGGG-----------AGCCTGAAACTAGGCTCACTGTGACTCA-
TTTGTGGCACCCATTATTGCCCAACCTGCAGTT-----CACTATGGTGAAGGAGCAA--TTCAGACTCACCCTGCAGCAT
CCCGTCTGGGTCAAACAGTGG---CTGCTCCCTAAGGAATAGTTGGGGGCGCTACATAAAGTAGTTTAAAAA-CTATTGT
AAAAAGGACATGTTTCACCAATG---

>11_277
A-TGGCC---AACTCCTCTTCCAGCGGTTCAGATGCCTGTAACATTACAACCTCAAATGCAGGTTAGACAAGTACAAACC
CCAAGAGAATATCAAATAGAGAG--------TCTCTGTCATGGCAACGCCAATCCAAATACAGTATCCACAATATCAGCC
GGTAGAAAATAAGACCCAACCGCCAGTAGCCTATCAATACTGGCCGCCAGCCGAACTTCAGTATCGGCCGCCCCC-AGAA
AATCAGTATGGACAGCCAGGAGTGTTTCCAGCACCACAGGGCAG----GGCGCTATAGCCTCAGCCGCCCACTATAAGAC
---TTTATCCTACAGCACCACCTAGT
GGACAAGGTAGTGCATTACATAAAATTATTGATAAG----------GCAAGGAAACAAGGAGA--TATTGAGGCGTGGCA
ATTCCCAGTA---------CCGATGCCACCTGGAGAAGGG-----------GCCCAAGAAGGAGCGCCTACCCTAGTTGG
GGCCAGATATGAGTCCTTTTCTATAAAAATGTT-AAAAGATATGAAAGAGGGAGTAA--AACAGTATGGACCCAACTCCC
CTTATATGAGAACATTATTAGGTTCCACTGCTCATGGACAT---AGACTCATTCC-TTA--TGATTGGGAGA-TTTTGGC
CAAATCATCACT-CTCACTGTC-TCAATTTTTACAATTTAAGAGTTGGTG-----------GATTGATGGG-----GCAC
AAGAACA
>11_424
A-GGCAC---AAGTCCTCTTCCAGCAGGTCAGGTGCCCGTAAGATTACAACCTCAAACGCAGGTTAA-------------
----
---AGAAAATAAGACCCAACCGCCAGTAGCTTATCAATACTGGCCGCCGGCTGAACTTCAGTATCGGCCACCCCC-AGAA
AGTCAGTATGGATATCCAGGAATGCCCCCAGCACCACAGGGCAG----GGCGCCATACCCTCAGCCACCCACTAGGAGAC
---TTAATCCTACGGCACCACCTAGT
AGACAGGGTAGTGAATTACATGAAATTATTGATAAA----------TCAAGAAAGGAAGGAGA--TACTGAGGCATGGCA
ATTCCCAGTAACGTTAGAACCGAAGCCACCTGGAGAAGGA-----------GCCCAAGAGGGAGAGCCTCCCACAGTTGA
GGCCAGATACAAGTCTTTTTCGATAAAAATGCT-AAAAGATATGAAAGAAGGAGTAA--AACAGTATGGACCCAACTCCC
CTTATATGAGGACATTATTAGATTCCATTGCTCATGGACAT---AGACTCATTCC-TTA--TGATTGGGAGA-TTCTGGC
AAAATCGTCTCT-CTCACCCTC-TCAATTTTTACAATTTAAGACTTGGTG-----------GATTGATGGG-----GTAC
AAGAACA
>11_456
A-GGGCC---AAGTCCTCTTCCAGCAGGTCAGGTGCCCGTAACATTACAACCTCAAACGCAGGTTAG-------------
----
---AGAAAATAAGAGCCAACCACCAGTAGCTTATCAATACTGGCCGCTGGCTGAACTTCAGTATCAGCCACCCCC-AGAA
AGTCAGTATGGATATCCAGGAATGCCCCCAGCACCACAGGGCAG----GGCGCCATACCCTCAGCCGCCCACTAGGAGAC
---TTAATCCTACGGCACCGCCTAGT
AGACAGGGTAGTGAATTACATGAAATTATTGAGA---------------AGAAAGGAAGGAGA--TACTGAGGCGTGGCA
ATTCCCAGTAACGTTAGAACCGATGCCACCTGGAGAAGAA-----------GCCCAAGAGGGAGAGCCTCTCACAGTTGA
GGCCAGATACAAGTCTTTTTTGATAAAAATGCT-AAAAGATATGAAAGAGGGAGTAA--AACAGTATGGACCCAACTCCC
CTTATATGAGTACATTATTAGATTCCATTGCTCATGGACAT---AGACTCATTCC-TTA--TGATTGGGAGA-TTCTGGC
AAGATCATCTCT-CTCACCCTC-TCAATTTTTACAATTTAAGACTTGGTG-----------GATTGATGGG-----GTAC
AAGAACA
>12_216
A-GGCAC---AAGTCCTCTTCCAGCAGGTCAGGTGCCCGTAACATTACAACCTCAAACGCAGGTTAA-------------
----
---AGAAAATAAGACCCAACCACCAGTAGCTTATCAATACTGGCCGCCGGCTGAACTTCAGTATCGGCCACCCCC-AGAA

```
AGTCAGTATGGATATCCAGGAATGCCCCCAGCACCACAGGGCAG----GGCGCCATACCCTCAGCCGCCCACTAGGAGAC
---------------------------------------------------------TTAATCCTACGGCACCACCTAGT
AGACAGGGTAGTGAATTACATGAAATTATTGATAAA----------TCAAGAAAGGAAGGAGA--TACTGAGGCATGGCA
ATTCCCAGTAATGTTAGAACCGATGCCACCTGGAGAAGGA-----------GCCCAAGAGGGAGAGCCTCCCACAGTTGA
GGCCAGATACAAGTCTTTTTCAATAAAAATGCT-AAAAGATATGAAAGAAGGAGTAA--AACAGTATGGACCCAACTCCC
CTTATATGAGGACATTATTAGATTCCATTGCTCATGGACAT---AGACTCATTCC-TTA--TGATTGGGAGA-TTCTGGC
AAAATCGTCTCT-CTTACCCTC-TCAATTTTTACAATTTAAGACTTGGTG-----------GATTGATGGG-----GTAC
AAGAACA
>12_419
--------------------------------------------------------------------------------
--------------------------------------------------------------------------------
--------------------------------------------------------------------------------
--------------------------------------------------------------------------------
--------------------------------------------------------------------------------
--------------------------------------------------GGCTGTT-GGGGT----TAAAAGCACGATG
GCCACC--TCCTCCTCAGCCGAGTGAGTGCTGGGGGAGGG-----------AGCCTGAAACTACGCTTGCTGCGACTTGG
CTCGTGGCACTCATTATTGCCCAACCTGCAGTT-----CACTACGGTGAAGGAGCAA--TTCAGACTTGCCCTGCAGCTT
CCTGTATGGGTCAAAGAGTGG---CCGCTCTCTAAGGAAAAGTTGGGGGTGTTACATAAAATAGTTAAAAAA-CTATTTT
AAATAGGATGTGTTCCACCCACT---------------------------------------------------------
-------
>14_023
A-TGGCCAACAACTCCTTCTCCCACGGTTCAGATGCCTGTGACATTACAACCTCAAA-----------------------
----------------------------------------------TGCCAATCCAGACACAGTATCCACAATATCAGCC
GGTAGAAAATAAAACCCAACCATCAGTAGTTTATGAACACCAGCCATCAGCCGAATTTCAGTATCGGCCGTCTCC-AGTG
GTTCAGTATAGATCCCAGGTGTTGTGTCCTGTGCCAAATAGCAG----GGCAGTATAGCAACAACCCATGGCGATGGTGT
---------------------------------------------------------TTGATCCTACACTACCACCTAGT
GGACAAGATAGTGCACTGCAAAAAATCATTGATAAA----------GCCAGAAAACAGGGAGA--TCTTGAGGCAGGGCA
GTTCCTGGTAATTTTACAACCAATACCGGCTGGAAAACGG-----------AGTCAAGCAGGAGCGTCTGTCCAAACTGA
GGCTAGATATGAATCTTTCACCATGAAAATGTT-AAAAGATATGAAGGAGGCAGTTA--AACAATATGGACCCAACTCTC
CTTATATGAGAACATTATTAGATTCCATTGCTAATGGGAAT---AGACTTATTCC-TTA--TGATTGGGAAA-TTTTGGC
TAAATCTTCTCT-TTCACCCTC-TCAGTATCTACAATTTAAAACCTGGTG-----------GATTGACGGA-----GTAC
AAGAACA
>16_010
A-TGGC---CAACTCCTCCTCCCGTGGTTCAGATACCTGTAACATTACAACCTCAAATGCAGGTTAGACAAGTACAAACC
CCAAGAGAATATCAAATAGAAAAGGATAG--TCTCTATCTCGGCAATGCCAATCCAGATACAGTATCCACAATATCAGCC
AGTAGAAAATAAAACCCAACCGCCATTAGCTTATCAATACTGGCTGCCAGCCGAGCTTCAGTATTGGCTGCCTCC-AGAG
GTCCAATACAGACCTCAAGTGGTGTGTGCCATGCCAAATTGCAC----GGCATCGTACAAGCAACCCATGGCGGTGGTGT
TTAATACGTCAGCACCACAGGGCGCGGCGCTGTGTCCTCAGCCGCCCACTATGAGACTTAATCCAACAGCACCACCTAGT
GGACAAGGTAGCACACTGCACGCGATCATTGATGAA----------GCTAGAAAACAGGGAGA--TCTTGAGGCGTGGCA
GTTCCTGGTAATTTTACAATCGGTACCGGCCGGGGAAGGG-----------GCTCCAGCAGGAGCACCTGCGGTGGCTAA
TGCTAGATATGAACGCTTCACCATGAAAATGTT-AAAAGACATGAAGGAAGGAGTTA--AACAATATGGACCCAACTCAC
CTTCTATGAGAACATTATTAGATTCCATTGCTCCAGCAGG----------------------------------------
--------------------------------------------------------------------------------
-------
>17_029
--------------------------------------------------------------------------------
--------------------------------------------------------------------------------
--------------------------------------------------------------------------------
--------------------------------------------------------------------------------
--------------------------------------------------------------------------------
--------------------------------------------------------------------------------
--------------------------------------------------------------------------------
-----------------------------------------------------------GTCAGTATGGTCCCAATTCGC
CTTTCATCCGATCGCTATTGCAATCTGTGGCTCAGAATAAG---CTATTGACTCC-GTG--TGATTGGGAGA-TTTTAAC
GAAAGTTACACT-TTCGCCCTC-CCAATTTCTTCAGTTTAAGACTTGGTG-----------GACCGACGAG-----GCTC
AAAATCA
>19_157
A-TGGCC---AACTCCTCTTCCAGCAGTTCAGATGCCTGTAACATTACAACCTCAAATGCAGGTTAGACAAGTACAAACC
TGAAGAGAATAACAAATAGAGAAAGATAGAGTATCTGTCACAGCAATGCCAATCCAAATACAGTATCCACAATATCAGCT
GGTAGAAAATAAGACCCAACTGCCAGTAGCCTATCAATACTGGCCGCCAGCCGAACTTCAGTATTGGCCGCCCCT-AGAA
AATCAGCATGGACAGCCAGGAATGTTTCCAGCACCACAGGGCAG----GGCACTATATCCGCAGCCTCCCACTATGAGAC
---------------------------------------------------------TTAATCCTACATCACCACCTAGT
AGACAGGGTGGTGCATTACATAAAATTATT---AAA----------GCAAGAAAACAAGGAGA--TATCGAGGCGTGGCA
GTTCCCAGTAATATTAGAGCCGATACCACCTGAAGAAGGG-----------GCCCAAGAGGGAGAGCCTCCCCTAGCTGA
GGCCAGATATGAGTCCTTTTCTATAAAAATGCT-AAAAGATGTGAAAGAGGGAGTAA--AA-AGTATGGACTCAACTCCC
CTTATTTGAGGACATTATTAAATTCCATTGCTCATGGACAT---AGACTCATTCC-TTT--TGATTGGGAGA-TTTTGGC
CAAATCATCACT-CTCACCCTC-T-ATCTTTTACAATTTAAGACTTTGTG-----------GATTGATGGG-----GTAC
AAGAACA
>19_238
A-GGGCC---ATGTCCTCTTCCAGCAGGTCAGATGCCCGTAATATTACAACCTCAAACGCAGTTTAG-------------
--------------------------------------------------------------------------------
---AGAAAATAAGACCCAACCGCCAGTAGCTTATCAATAATGGCCGCCAGCCAAACTTCAGTATCGGCCACCCCC-AGAA
AGTCAGTATGGATATCCAGGAACGCTCCCAGCACCACAGGGCAG----GGCGCCATACCCTCAGCCACCCACTAGGAGAC
---------------------------------------------------------TTAATCCTATGGTACCACCTAGT
AGACAGGGTAGTGAATTACCAGAAATTATTGATAAG----------TCAAGAAAGGAAGGAGA--TATTGAGGCATGGCA
ATTCCCAATAATGTTAGA------ACCACCTGGAGAAGGA-----------GCCCAAGAGGGAGAGCCTCTCACAGTTGA
```

GGCCAGATACAAGTCTTTTTCGATAAAAATGCT-AAAAGATATGAAAGAGGGAGTAA--AACAGTATGGACCCAACTCTC
CTTATATGAGGACATTATTAGATTCCATTGCTCATGGACAT---AGACTCATTCC-TTA--TGATTGGGAGA-TTCTGGC
AAAATCGTCTCT-CTCACCCTC-TCAATTTTTACAATTTAAGACTTGGTG-----------GATTGATGGG-----GCAC
AAGAACA
>19_352
A-GGCAC---AAGTCGTCTTCCAGCAGGTCAGGTGCCCGTAACATTACAACCTCAAACGCAGGTTAA------------
----
---AGAAAATAAGACCCAACCGCCAGTAGCCTATCAATACTGGCCGCCGGCTGAACTTCAGTATCGGCCACCCCT-AGAA
AGTCAGTATGGATATCCAGGAATGCCCCCAGCACCACAGGGCAG----GGCGCCATACCCTCAGCCGCCCACTAGGAGAC
---TTAATCCTACGGCACCACCTAGT
AGACGGGGTAGTGAATTACATGAAATTATTGATAAA----------TCAAGAAAGGAAGGAGA--TACTGAGGCATGGCA
ATTCCCAGTAACGTTAGAACCGATGCCACCTGGAGAAGGA-----------GCCCAAGAGGGAGAGCCTCCCACAGTTGA
GGCCAGATACAAGTCTTTTTCGATAAAAATGCT-AAAAGATATGAAAGAGGGAGTAA--AACAGTATGGACCCAACTCCC
CTTATATGAGGACATTATTAGATTCCATTGCTCATGGACAT---AGACTCATTCC-TTA--TGATTGGGAGA-TTCTGGC
AAAATCGTCTCT-CTCACCCTC-TCAATTTTTACAATTTAAGACTTGGTG-----------GATTGATGGG-----GTAC
AAGAACA
>19_407
A-TGGCC---AACTCCTCTCCCAGCAGTTCAGATGCCTGTAACATTACAACCTCAAATGCAGGTTAGACAAGTACAAACC
CCAAAAGAATATCAAATAGGGAAAGATAGAGTCTCTGTCACGGCAATGCCAATCCAAATACAGTATCCACAATATCAGCC
GGTGGAAAATAAGACCCAACCGCCAGTAACCTATCAATACTGGCCGCCAGCCGAACTTCAGGATCAGCCGCCCCC-AGAA
AATCAGTATGGACAGCCAGGAATGTTTCCAGCACCACAGGGCAG----GGCGCTATATCCTCAGCAGCCCACTATGAGAT
---TTAATCCTACACCACCACCTAGT
GGACAGGGTAGTGCATTACATAAAATTATTGATAAG----------GCAAGAAAACAAGGAGA--TACCGAGGCATGGCA
ATTCCCAGTAATATTAGAACCGATACCACCTGAAGAAGAG-----------GCCCAAGAGGGAGCGCCTCCCCTAGCTGA
GGCCAGATATGAGTCCTTTTCCATAAAAATGCT-AAAAGATATGAAAGAGGGAGTAA--AACAGTATGGACCCAACTCCC
CTTATATGAGGACATTATTAGATTCCATTGCTCATGGACAT---AGACTCATTCT-TTA--TGATTGGGAGA-TTTTGGC
CAAATGATCGCT-CTCACCCTC-TCAATGTTTACAATTTAAGACTTGGTGAAGACTTGGTGGATTGATGGG-----GCAC
AAGAACA
>19_639

--GGTCATA-GGGGC----TAAAACCACGATG
GCCACC--TCCTCCTCAGCCAAGTGAGTGCTGGGGGAGGG-----------AGCCTGAAACTAGGCTCACTGCAACTTGG
CTCGCTGTGCCCATTATTGCCCAACTTACAGTT-----CATTATGGTGAAGGAGCAA--TTCAGACTTGCCTTGCAGCAT
CCTGTCTGAGTCAAACAGTGG---CTGCTCCCTAAGGAAAATTTGGGGGCGCTACATAAAATAGTTAAAAAA-CTACTTA
AAAA-GGACACGTTTCACCCACT---

>20_078
A-TGGCC---AACTCCTCCTCCCACAGTTCAGATGCCCGTAACATTACAACCTCAAATGCAGGTTAGACAAGTACAAACC
CCAAGAGAATATCAGATAGAAAAGGATAG--TCTCTATCACAGCAATGCCAATCCAGATACAGTATTCACAATATCAGCT
GGTAGAAAGTAAGACCCAACCGCCAGTACCCTATCAATACTGACCGCCAGCCGAACTTCAGTATCAGCCGCCCCCCAGAA
AATCAGTATGGACAGCCAGGAACGTTTCCAGCACCACAGGGCACGCACGGCGATATAGCCTCAGCTGCCCACTATGAGAC
---TTAATCCTACACCACCACCTAGT
GGACAGGATGGTGCATTACATGAAATTATTGAGAAG----------GCAAGAAAACAAGGAGAGATACTGAGGCGTTTTA
ATTTCAAGTAATATTAGAGCCCATACTACCTGGAGAAGGG-----------CCC--------------TCCCCAAGCTGA
TGCCAGATATGAGTCCTTTTCTATAAAAATGCT-AAAAGACATGAAAGAGGGAGTAA--AACAGTATGGACCCAATTCTC
CTTATATGAGAACATTATTAGATTCCATTGCTCATGGAAAT---AGACTTATTCC-TTA--TGACTGGGAAA-TTTTGGC
CAAATCATCACT-CTCACCTTC-TCAATTTTTACAATTTAAGACCTGGTG-----------GATTGATGGG-----GTAC
AACAACA
>21_019
A-AGGCC---AAGTCCTCTTCCAGCAAGTCAGGTGCCTGTAACATTACAACCTCAAACGCAGGTTAA------------
----
---AGAAAATAAGACCCAACCGCCAGTAGCTTATCAATACTAGCCGCCGGCTGAACTTCAGTATCGGCCACCCCC-AGAA
AGTCAGTATGGATATCCAAGAATGCCCCCAGCACCACAGGGCAG----GGCGCCATACCCTCAGCCGCCCACTAGGAGAC
---TTAATCCTACGGCACCACCTAGT
AGACAAGGTAGTGAATTACATGAAATTATTGATAAA----------TCAAGAAAGGAAGGAGA--TACTGAGGCATGGCA
ATTCCCAGTAATGTTAGAACCGATGCCACCTGGAGAAGGA-----------GCCCAAGAGGGAGAGCCTCTCACAGTTGA
GGCCAGATACAAGTCTTTTTCGATAAAAATGCT-AAAAGATATGAAAGAAGGAGTAA--AACAGTATGGACCCAACTCCC
CTTATATGAGGACATTATTAGATTCCATTGCTCATGGACAT---AGACTCATTCC-TTA--TGATTAGGAGA-TTCTGGC
AAAATCGTCTCT-CTCACCCTC-TCAATTTTTACAATTTAAGACTTAGTA-----------GATTGATGAG-----GTAC
AAGAACG
>22_012
A-GGGAC---AAGTCCTCTTCCAGCAGGTCAGGTGCCCGTAACATTACAACCTCAAAAGCAGGTTAA------------
----
---AGAAAATAAGACCCAACCGCCAGTAGCCTATCAATACTGGCCTCCGGCTGAACTTCAGTATCGGCCACCCCC-AGAA
AGTCAGTATGGATATCCAGGAATGCCCCCAGCACCACAGGGCAG----GGCGCCATACCCTCAGCCGCCCACTAGGAGAC
---TTAATCCTACGGCACCACCTAGT
AGACAGGGTAGTGAATTACATGAAATTATTGATAAA----------TCAAGAAAGGAAGGAGA--TACTGAGGCATGGCA
ATTCCCAGTAACGTTAGAACCGATGCCACCTGGAGAAGGA-----------GCCCAAGAGGGAGAGCCTCCCACAGTTGA
GGCCAGATACAAGTCTTTTTCGATAAAAATGCT-AAAAGATATGAAAGAGGGAGTAA--AACAGTATGGACCCAACTCCC
CTTATATGAGGACATTATTAGATTCCATTGCTTATGGACAT---AGACTCATTCC-TTA--TGATTGGGAGA-TTCTGGC
AAAATCGTCTCT-CTCACCCTC-TCAATTTTTACAATTTAAGACTTGGTG-----------GATTGATGGG-----GTAC
AAGAACA

```
>22_041
A-TCGCCATCAACTCCTCCTCCCGCGGTTCAGATGCCTGTAACATTACAACCTCAAACGCAGGTTAGACAAGCACAAACC
CCAAGAGAAAATCAAGTAGAAAGGGACAGAGTCTCTATCCCGGCAATGCCAACTCAGATACAGTATCCACAATATCAGCC
GGTAGAAAATAAGACCCAACCGCTGGTAGTTTATCAATACCGGCTGCCAACCGAGCTTCAGTATCGGCCTCCTTC-AGAG
GTTCAATACAGACCTCAAGCGGTGTGTCCTGTGCCAAATAGCAC----GGCACCATACCAGCAACCCACAGCGATGGCGT
CTAATTCACCAGCAACACAGGACGCGGCGCTGTATCCTCAGCCGCCCACTGTGAGACTTAATCCTACAGCATCACGTAGT
GGACAGGGTGGTGCACTGCATGCAGTCATTGATGAA----------GCCAGAAAACAGGGCGA--TCTTGAGGCATGGCG
GTTCCTGGTAATTTTACAACTGGTACAGGCCGGGGAAGAG-----------ACTCAAGTAGGAGCGCCTGCCCGAGCTGA
GACTAGATGTGAACCTTTCACCATGAAAATGTT-AAAAGATATAAAGGAAGGAGTTA--AACAATATGGATCCAACTCCC
CTTATATAAGAACATTATTAGATTCCATTGCTCATGGAAAT---AGACTTACTCC-TTA--TGACTGGAAA-TTTTGGC
CAAATCTTCCCT-TTCATCCTC-TCAGTATCTACAGTTTAAAACCTGGTG-----------GATTGATGGA-----GTAC
AAGAACA
>X_540
--------------------------------------------------------------------------------
--------------------------------------------------------------------------------
--------------------------------------------------------------------------------
--------------------------------------------------------------------------------
--------------------------------------------------------------------------------
--------------------------------------------------GGCCGTC-AGGGC----TAAAACCACGATG
GCCACC--TCCTCCTCAGTCGAGTGAGTGCTGGGGGAGGG-----------AGCCTGAAACCAGGCTGGCTGCAACTCGG
CTCGCGGTGCCCATTATTGCCCAACCGGCAGTT-----CACTGCGGTGAAGGAGCAA--TTCAGACTCGCCCTGTAGCAT
CCTGTCTGGGTCAAACAGTGG---CCGCTCCCTAAGGAAAAGTTAGGGGCGCTACATAAAATAGTTAAAAAAACTATTTA
AAAAAGGACATGTTTCACCCACT---------------------------------------------------------
-------
>Y_026
A-TGGCCACCAACTCCTTCTCCCGCGGTTCAGATGCCTGTGACATTACAACCTCAAA-----------------------
----------------------------------------------TGCCAATCCAGACACAGTATCCACAATATCAGCT
GGTGGAAAATAAAACCCAACCACCGGCAGCTTATCAACACCAGCCGCCAGCCGAATTTCAGTATCGGCCGTCTCC-AGAG
GTTCAGTATAGAACTCAGGTGGTGCGCCCAAC-CCAAATAGCAG----GGCACTATATCAACATCCCACGGCAATGGTGT
---------------------------------------------------------TTGATCCTACAGTACCACCTAGT
GGACAAGATAGTGCACTCCATGAAATCATTGATAAA----------GCCAGAAAACAGGGAGA--TCTTGAGGCATGGCA
GTTCCCAGTAATTTTACAACCCAC-CCGGCCGGGAAAGGG-----------AGTCAAGCAGGAGCGTCTGTCCGAACTGA
GTGTAGATATGAATCTTTCACCATGAAAATGTT-AAAAGATATGAAGGAAGGAGTTA--AACAATATGGACCCAACTCTC
CTTGTATGAGAACATTATTAGATTCCATTGCTCTTGGAAAT---AGACTTATTCC-TTA--GGATTGGGAAA-TTTTGGC
TAAATCTTCCCT-TTCACCCTC-TCAATTCCTACAGTTTAAAACCTGGTA-----------GATTGATGGA-----GTAC
AAGAATA
>HML2_Rep
A-GGCAC---AAGTCCTCTTCCAGCAGGTCAGGTGCCTGTAACATTACAACCTCAAAAGCAGGTTAA-------------
--------------------------------------------------------------------------------
---AGAAAATAAGACCCAACCGCCAGTAGCCTATCAATACTGGCCTCCGGCTGAACTTCAGTATCGGCCACCCCC-AGAA
AGTCAGTATGGATATCCAGGAATGCCCCCAGCACCACAGGGCAG----GGCGCCATACCCTCAGCCGCCCACTAGGAGAC
---------------------------------------------------------TTAATCCTACGGCACCACCTAGT
AGACAGGGTAGTAAATTACATGAAATTATTGATAAA----------TCAAGAAAGGAAGGAGA--TACTGAGGCATGGCA
ATTCCCAGTAACGTTAGAACCGATGCCACCTGGAGAAGGA-----------GCCCAAGAGGGAGAGCCTCCCACAGTTGA
GGCCAGATACAAGTCTTTTTCGATAAAAAAGCT-AAAAGATATGAAAGAGGGAGTAA--AACAGTATGGACCCAACTCCC
CTTATATGAGGACATTATTAGATTCCATTGCTCATGGACAT---AGACTCATTCC-TTA--TGATTGGGAGA-TTCTGGC
AAAATCGTCTCT-CTCACCCTC-TCAATTTTTACAATTTAAGACTTGGTG-----------GATTGATGGG-----GTAC
AAGAACA
>K113
A-GGCAC---AAGTCCTCTTCCAGCAGGTCAGGTGCCCGTAACATTACAACCTCAAAAGCAGGTTAA-------------
--------------------------------------------------------------------------------
---AGAAAATAAGACCCAACCGCCAGTAGCCTACCAATACTGGCCGCCGGCTGAACTTCAGTATCAGCCACCCCC-AGAA
AGTCAGTATGGATATCCAGGAATGCCCCCAGCACCACAGGGCAG----GGCGCCATACCCTCAGCCGCCCACTAGGAGAC
---------------------------------------------------------TTAATCCTACGGCACCACCTAGT
AGACAGGGTAGTGAATTACATGAAATTATTGATAAA----------TCAAGAAAGGAAGGAGA--TACTGAGGCATGGCA
ATTCCCAGTAACGTTAGAACTGATGCCACCTGGAGAAGGA-----------GCCCAAGAGGGAGAGCCTCCCACAGTTGA
GGCCAGATACAAGTCTTTTTCGATAAAAATGCT-AAAAGATATGAAAGAGGGAGTAA--AACAGTATGGACCCAACTCCC
CTTATATGAGGACATTATTAGATTCCATTGCTCATGGACAT---AGACTCATTCC-TTA--TGATTGGGAGA-TTCTGGC
AAAATCGTCTCT-CTCACCCTC-TCAATTTTTACAATTTAAGACTTGGTG-----------GATTGATGGG-----GTAC
AAGAACA
>K115
A-GGCAC---AAGTCCTCTTCCAGCAGGTCAGGTGCCCGTAACATTACAACCTCAAAAGCAGGTTAA-------------
--------------------------------------------------------------------------------
---AGAAAATAAGACCCAACCGCCAGTAGCCTATCAATACTGGCCTCCGGCTGAACTTCAGTATCGGCCACCCCC-AGAA
AGTCAGTATGGATATCCAGGAATGCCCCCAGCACCACAGGGCAG----GGAGCCATACCCTCAGCCGCCCACTAGGAGAC
---------------------------------------------------------TTAATCCTACGGCACCACCTAGT
AGACAGGGTAGTGAATTACATGAAATTATTGATAAA----------TCAAGAAAGGAAGGAGA--TACTGAGGCATGGCA
ATTCCCAGTAACGTTAGAACCGATGCCACCTGGAGAAGGA-----------GCCCAAGAGGGAGAGCCTCCCACAGTTGA
GGCCAGATACAAGTCTTTTTCGATAAAAATGCT-AAAAGATATGAAAGAGGGAGTAA--AACAGTATGGACCCAACTCCC
CTTATATGAGGACATTATTAGATTCCATTGCTCATGGACAT---AGACTCATTCC-TTA--TGATTGGGAGA-TTCTGGC
AAAATCGTCTCT-CTCACCCTC-TCAATTTTTACAATTTAAGACTTGGTG-----------GATTGATGGG-----GTAC
AAGAACA
>622
A-GGCAC---AAGTCCTCTTCCAGCAGGTCAGGTGCCTGTAACATTACAACCTCAAAAGCAGGTTAA-------------
--------------------------------------------------------------------------------
---AGAAAATAAGACCCAACCGCCAGTAGCCTATCAATACTGGCCTCCGGCTGAACTTCAGTATCGGCCACCCCC-AGAA
```

AGTCAGTATGGATATCCAGGAATGCCCCCAGCACCACAGGGCAG----GGCGCCATACCCTCAGCCGCCCACTAGGAGAC
---TTAATCCTACAGCACCACCTAAT
AGACAGGGTAGTGCATTACATAAAATTATTGATAAG----------TCAAGAAAGCAAGGAGA--TATTGAGGCGTGGCC
ATTCCCACTAATGTTAGAACTGATACCACCTGGAGAAGGA-----------GCCCAAGAGGGAGAGCCTCCCACAGTTGA
GGCCAGATACAAGTCTTTTTCTATAAAAATGCT-AAAAGACATGAAAGAGGGAATAA--AACAGTATGGACCCAACTCCC
CTTATATGAGGACATTATTAGATTCCATTGCACACGGACAT---AGACTCATTCC-TTA--TGATTGGGAGA-TTCTGGC
AAAATCGTCTCT-CTCACCCTC-TCAATTTTTACAATTTAAGACTTGGTG-----------GATTGATAGA-----GTAC
AAGAACA
>108
A-AGGCC---AAGTCCTCTTCCAGCAAGTCAGGTGCCTGTAACATTACAACCTCAAACGCAGGTTAA-------------
----
---AGAAAATAAGACCCAACTGCCAGTAGCCTATCAATACTGGCTGCCAGCCGAACTTAAGTGTCGGCCACCCCC-GGAA
AATCAGTATGGACAGCCAGGAATGTTCCCAGCCCCACAGGACAG----GGCGCCATACCCTCAGCCACCCACTATGAGAC
---TTAATCCTACAGCACCACCTAGT
GAGCAGGGTAGTGTATTACATAAAATTATTGATGAG----------GCAAGAAAACAAAGAGA--TATTGAGGCATGGCA
ATTCCCAGTAATATTAGAACCAATACCACCTGGAGAAGGG-----------GCCCAAGAGGGAGAGCCTCCCCTAGCTGA
GGCCAGATATGAGTCTTTTTCTATAAAAATGCT-AAAAGATATGAAAGAGGGAGTAA--AACAGTATGGACCCAACTCCC
CTTATATGAGGACATTATTAGATTCCATTGCTCATGGACAT---AGACTCATTCC-TTA--TGATTGGGAGG-TTCTGGC
CAAATTGTCTCT-CTCACCCTC-TCAATTTTAACAATTTAAGACTTGGTG-----------GATTGATGGG-----GCAC
AACAACA
>94
A-GGGCC---AAGTCCTCTTCCAGCAGGTCAGGTGCCCGTAACATTACAACCTCAAACGCAGGTTAG-------------
----
---AGAAAATAAGAGCCAACCACCAGTAGCTTATCAATACTGGCCGCTGGCTGAACTTCAGTATCAGCCACCCCC-AGAA
AGTCAGTATGGATATCCAGGAATGCCCCCAGCACCACAGGGCAG----GGCGCCATACCCTCAGCCGCCCACTAGGAGAC
---TTAATCCTACGGCACCGCCTAGT
AGACAGGGTAGTGAATTACATAAAATTATTGATGAG----------GCAAGAAAACAAAGAGA--TATTGAGGCATGGCA
ATTCCCAGTAATATTAGAACCAATACCACCTGGAGAAGGG-----------GCCCAAGAGGGAGAGCCTCCCCTAGCTGA
GGCCAGATATGAGTCTTTTTCTATAAAAATGCT-AAAAGATATGAAAGAGGGAGTAA--AACAGTATGGACCCAACTCCC
CTTATATGAGTACATTATTAGATTCCATTGCTCATGGACAT---AGACTCATTCC-TTA--TGATTGGGAGA-TTCTGGC
AAGATCATCTCT-CTCACCCTC-TCAATTTTTACAATTTAAGACTTGGTG-----------GATTGATGGG-----GTAC
AAGAACA
>93
A-GGGCC---AACTCCTCTTCCAGCAGTTCAGATGCCTGTAACATTACAACCTCAAATGCAGGTTAGGCAAGTACAAACC
CCAAGAGAATATCAAATAGAGAAAGATAGAGTCTCTGTCACGGCAATGCCAATCCAAATACAGTATCCACAATATCAGCC
AGTAGAAAATAAGACCCAACTGCCAGTAGCCTATCAATACTGGCTGCCAGCCGAACTTAAGTGTCGGCCACCCCC-GGAA
AATCAGTATGGACAGCCAGGAATGTTCCCAGCCCCACAGGACAG----GGCGCCATACCCTCAGCCACCCACTATGAGAC
---TTAATCCTACAGCACCACCTAGT
GAGCAGGGTAGTGTATTACATAAAATTATTGATGAG----------GCAAGAAAACAAAGAGA--TATTGAGGCATGGCA
ATTCCCAGTAATATTAGAACCAATACCACCTGGAGAAGGG-----------GCCCAAGAGGGAGAGCCTCCCCTAGCTGA
GGCCAGATATGAGTCTTTTTCTATAAAAATGCT-AAAAGATATGAAAGAAGGAGTAA--AACAGTATGGACCCAACTCCC
CTTATATGAGGACATTATTAGATTCCATTGCTCATGGACAT---AGACTCATTCC-TTA--TGATTAGGAGA-TTCTGGC
AAAATCGTCTCT-CTCACCCTC-TCAATTTTTACAATTTAAGACTTAGTA-----------GATTGATGAG-----GTAC
AAGAACG
>B12
A-GGCAC---AAGTCCTCTTCCAGCAGGTCAGGTGCTCGTAAGATTACAACCTCAAAAGCAGGTTAA-------------
----
---AGAAAATAAGACCCAACCGCAAGTAGCCTATCAATACTGGCCGCTGGCTGAACTTCAGTATCGGCCACCCCC-AGAA
AGTCAGTATGGATATCCAGGAACGCTCCCAGCACCACAGGGCAG----GGCGCCATACCCTCAGCCACCCACTAGGAGAC
---TTAATCCTATGGCACCACCTAGT
AGACAGGGTAGTGAATTACATGAAATTATTGATAAA----------TCAAGAAAGGAAGGAGA--TACTGAGGCATGGCA
ATTCCCAGTAACGTTAGAACCGATGCCACCTGGAGAAGGA-----------GCCCAAGAGGGAGAGCCTCCCACAGTTGA
GGCCAGATACAAGTCTTTTTCGATAAAAATGCT-AAAAGATATGAAAGAGGGAGTAA--AACAGTATGGACCCAACTCCC
CTTATATGAGGACATTATTAGATTCCATTGCTCATGGACAT---AGACTCATTCC-TTA--TGATTGGGAGG-TTCTGGC
CAAATTGTCTCT-CTCACCCTC-TCAATTTTAACAATTTAAGACTTGGTG-----------GATTGATGGG-----GCAC
AACAACA
>B362
A-GGCAC---AAGTCCTCTTCCAGCAGGTCAGGTGCCCGTAAGATTACAACCTCAAACGCAGGTTAA-------------
----
---AGAAAATAAGACCCAACCGCCAGTAGCTTATCAATACTGGCCGCTGGCTGAACTTCAGTATCGGCCACCCCC-AGAA
AGTCAGTATGGATATCCAGGAATGCCCCCAGCACCACAGGGCAG----GGCGCCATACCCTCAGCCACCCACTAGGAGAC
---TTAATCCTATGGCACCACCTAGT
GAGCAGGGTAGTGTATTACATAAAATTATTGATGAG----------GCAAGAAAACAAAGAGA--TATTGAGGCATGGCA
ATTCCCAGTAATATTAGAACCAATACCACCTGGAGAAGGG-----------GCCCAAGAGGGAGAGCCTCCCCTAGCTGA
GGCCAGATATGAGTCTTTTTCTATAAAAATGCT-AAAAGATATGAAAGAGGGAGTAA--AACACTATGGACCCAACTCCC
CTTATATGAGGACATTATTAGATTCCATTGCTCATGGACATCATAGACTCATTCC-TTA--TGATTGGGAGA-TTCTGGC
AAAATCGTCTCT-CTCACCCTC-TCAATTTTTACAATTTAAGACTTGGTG-----------GATTGATGGG-----GCAC
AAGAACA
>B276
A-GGCAC---AAGTCATCTTCCAGCAGGTCAGGTGCCCGTAACATTACAACCTCAAAAGCAGGTTAA-------------
----
---AGAAAATAAGACCCAACCGCCAGTAGCCTATCAATACTGGCCGCCGGCTGAACTTCAGTATCGGCCACCCCC-AGAA
AGTCAGTATGGATATCCAGGAATGCCCCCAGCACCACAGGGCAG----GGCGCCATACCCTCAGCTGCCCACTAGGAGAC
---TTAATCCTATGGCACCACCTAGT
AGACAGGGTAGTGAATTACATGAAATTATTGATAAA----------TCAAGAAAGGAAGGAGA--TACTGAGGCGTGGCA
ATTCCCAGTAACATTAGAACCGATGCCAACTGGAGAAGGA-----------GCCCAAGAGGGAGAGCCTCCCCTAGCTGA

GGCCAGATATGAGTCTTTTTCTATAAAAATGCT-AAAAGATATGAAAGAGGGAGTAA--AACAGTATGGACCCAACTCCC
CTTATATGAGGACATTATTAGATTCCATTGCTCATGGACAT---AGACTCATTCC-TTA--TGATTGGGAGG-TTCTGGC
CAAATTGTCTCT-CTCACCCTC-TCAATTTTAACAATTTAAGACTTGGTG-----------GATTGATGGG-----GCAC
AACAACA
>B282
A-GGCAC---AAGTCATCTTCCAGCAGGTCAGGTGCCCGTAACATTACAACCTCAAAAGCAGGTTAA-------------
----
---AGAAAATAAGACCCAACCGCCAGTAGCCTATCAATACTGGCCACCGGCTGAACTTCAGTATCGGCCACCCCC-AGAA
AATCAGTATGGACAGCCAGGAATGTTCCCAGCCCCACAGGACAG----GGCGCCATACCCTCAGCCACCCACTATGAGAC
---TTAATCCTACAGCACCACCTAGT
GAGCAGGGTAGTGTATTACATAAAATTATTGATGAG----------GCAAGAAAACAAAGAGA--TATTGAGGCATGGCA
ATTCCCAGTAATATTAGAACCAATACCACCTGGAGAAGGG-----------GCCCAAGAGGGAGAGCCTCCCCTAGCTGA
GGCCAGATATGAGTCTTTTTCTATAAAAATGCT-AAAAGATATGAAAGAGGGAGTAA--AACAGTATGGACCCAACTCCC
CTTATATGAGGACATTATTAGATTCCATTGCTCATGGACAT---AGACTCATTCC-TTA--TGATTGGGAGA-TTCTGGC
AAAATCGTCTCT-CTCACCCTC-TCAATTTTTACAATTTAAGACTTGGTG-----------GATTGATAGA-----GTAC
AAGAACA
>B188
A-GGCAC---AAGTCCTCTTCCAGCAGGTCAGGTGCCCGTAAGATTACAACCTCAAACGCAGGTTAA-------------
----
---AGAAAATAAGACCCAACCGCCAGTAGCTTATCAATACTGGCCGCCGGCTGAACTTCAGTATCGGCCACCCCC-AGAA
AGTCAGTATGGATATCCAGGAATGTTCCCAGCCCCACAGGACAG----GGCGCCATACCCTCAGCCACCCACTATGAGAC
---TTAATCCTACAGCACCACCTAGT
GAGCAGGGTAGTGAATTACATGAAATTATTGATAAA----------TCAAGAAAGGAAGGAGA--TACTGAGGCATGGCA
ATTCCCAGTAACGTTAGAACCGATGCCACCTGGAGAAGGA-----------GCCCAAGAGGGAGAGCCTCCCACAGTTGA
GGCCAGATACAAGTCTTTTTCGATGAAAATGCT-AAAAGATATGAAAGAGGGAGTAA--AACAGTATGGACCCAACTCCC
CTTATATGAGGACATTATTAGATTCCATTGCTCATGGACAT---AGACTCATTCC-TTA--TGATTGGGAGG-TTCTGGC
CAAATTGTCTCT-CTCACCCTC-TCAATTTTAACAATTTAAGACTTGGTG-----------GATTGATGGG-----GCAC
AACAACA
>B262
A-GAGCC---AAGTCCTTTTCTAGCAGGTCAGATGCCCATAACATTACAACCTCAAACGCAGGTTAG-------------
----
---AGAAAATAAGACCCAACCACCAGTAGCTTATCAATACTGGCCACTGGCTGAACTTCAGTATCGGCCACCCCC-AGAA
AGTCAGTATGGATATCCAGGAATGCCCCCAGCACCACAGGGCAG----GGCGCCATACCATCAGCCGCCCACTAGGAGAC
---TTAATCCTATGGCACCACCTAGT
AGACAGGGTAGTGAATTACATGAAATTATTGATAAA----------TCAAGAAAGGAAGGAGA--TACTGAGGCATGGCA
ATTCCCAGTAACGTTAGAACCGATGCCACCTGGAGAAGGA-----------GCCCAAGAGGGAGAGCCTCCCACAGTTGA
GGCCAGATACAAGTCTTTTTCGATAAAAATGCT-AAAAGATATGAAAGAGGGAGTAA--AACAGTATGGACCCAACTCCC
CTTATATGAGGACATTATTAGATTCCATTGCTCATGGACAT---AGACTCATTCC-TTA--TGATTGGGAGA-TTCTGGC
AAAATCGTCTCT-CTCGCCCTC-TCAATTTTTACAATTTAAGACTTGGTG-----------GATTGATAGA-----GTAC
AAGAACA
>B358
A-GGCAC---AAGCCCTCTTCCAGCAGGTCAGGTGCTCGTAAGATTACAACCTCAAAAGCAGGTTAA-------------
----
---AGAAAATAAGACCCAACTGCCAGTAGCCTATCAATACTGGCTGCCAGCCGAACTTAAGTGTCGGCCACCCCC-GGAA
AATCAGTATGGACAGCCAGGAATGTTCCCAGCCCCACAGGACAG----GGCGCCATACCCTCAGCCACCCACTATGAGAC
---TTAATCCTACAGCACCACCTAGT
GAGCAGGGTAGTGTATTACATAAAATTATTGATGAG----------GCAAGAAAACAAAGAGA--TATTGAGGCATGGCA
ATTCCCAGTAATATTAGAACCAATACCACCTGGAGAAGGG-----------GCCCAAGAGGGAGAGCCTCCCCTAGCTGA
GGCCAGATATGAGTCTTTTTCTATAAAAATGCT-AAAAGATATGAAAGAGGGAGTAA--AACAGTATGGACCCAACTCCC
CTTATATGAGGACATTATTAGATTCCATTGCTCATGGACAT---AGACTCATTCC-TTA--TGATTGGGAGG-TTCTGGC
CAAATTGTCTCT-CTCACCCTC-TCAATTTTTACAATTTAAGACTTGGTG-----------GATTGATAGA-----GTAC
AAGAACA
>B98a
A-GGACC---AAGTCCTCTTCCAGTAGGTCAGATGCCCGTAACATTACAACCTCAAACGCAGGTTAG-------------
----
---AGGAAATAAGACCCAACTACCAGTAGCTTATCAATACTGGCCGCCGGCCAAACTTCAGTATCGGCCACCCCC-AGAA
AGTTAGTATGGATATCCAGGAATGCCCCCAGCACCACAGGGCAG----GGCGCCATACCCTCAGCCGCCCACTAGGAGAC
---TTAATCCTACGGCACCACCTAGT
AGACAGGGTAGTGAATTACATGAAATTATTGATAAA----------TCAAGAAAGGAAGGAGA--TACTGAGGCATGGCA
ATTCCCAGTAACGTTAGAACCGATGCCAACTGGAGAAGGA-----------GCCCAAGAGGGAGAGCCTCCCCTAGCTGA
GGCCAGATATGAGTCTTTTTCGATAAAAATGCT-AAAAGATATGAAAGAGAGAGTAA--AACAGTATGGACCCAACTCCC
CTTATATGAGGACATTATTAGATTCCATTGCTCATGGACAT---AGACTCATTCC-TTA--TGATTGGGAGA-TTCTGGC
AAAATCGTCTCT-CTCACCCTC-TCAATTTTTACAATTTAAGACTTGGTG-----------GATTGATGGG-----GTAC
AAGAACA
>B269
A-GGCAC---AAGTCCTCTTCCAGCAGGTCAGGTGCCTGTAACATTACAACCTCAAACGCAGGTTAA-------------
----
---AGAAAATAAGACCCAACTGCCAGTAGCCTATCAATACTGGCTGCCAGCCGAACTTAAGTGTCGGCCACCCCC-GGAA
AATCAGTATGGACAGCCAGGAATGTTCCCAGCCCCACAGGACAG----GGCGCCATACCCTCAGCCACCCACTATGAGAC
---TTAATCCTACAGCACCACCTAGT
GAGCAGGGTAGTGTATTACATAAAATTATTGATGAG----------GCAAGAAAACAAAGAGA--TATTGAGGCATGGCA
ATTCCCAGTAATATTAGAACCAATACCACCTGGAGAAGGG-----------GCCCAAGAGGGAGAGCCTCCCCTAGCTGA
GGCCAGATATGAGTCTTTTTCTATAAAAATGCT-AAAAGATATGAAAGAGGGAGTAA--AACAGTATGGACCCAACTCCC
CTTATATGAGGACATTATTAGATTCCATTGCTCATGGACAT---AGACTCATTCC-TTA--TGATTGGGAGG-TTCTGGC
CAAATTGTCTCT-CTCACCCTC-TCAATTTTAACAATTTAAGACTTGGTG-----------GATTGATGGG-----GCAC
AACAACA

>B251
A-GGCAC---AAGTCCTCTTCCAGCAGGTCAGGTGCCTGTAACATTACAACCTCAAACGCAGGTTAGGCAAGTACAAACC
CCAAGAGAATATCAAATAGAGAAAGATAGAGTCTCTGTCACGGCAATGCCAATCCAAATACAGTATCCACAATATCAGCC
GGTAGAAAATAAGACCCAACTGCCAGTAGCTTATCAATACTGGCCGCCGGCCAAACTTCAGTATCGGCCACCCCC-AGAA
AGTCAGTATGGATATCCAGGAACGCTCCCAGCACCACAGGGCAG----GGTGCCATACCCTCAGCCACCCACTAGCAGAC
---TTAATCATACGGCACCACCTAGT
AGACAGGGTGGTGAATTACATGAAATTATTGATAAA----------TCAAGAAAGGAAGGAGA--TACTGAGGCATGGCA
ATTCCCAGTAACGTTAGAACCGATGCCACCTGGAGAAGGA-----------GCCCAAGAGGGAGAGCCTCCCACAGTTGA
GGCCAGATACAAGTCTTTTTCGATAAAAATGCT-AAAAGATATGAAAGAGGGAGTAA--AACAGTATGGACCCAACTCCC
CTTATATGAGGACATTATTAGATTCCATTGCTCATGGACAT---AGACTCATTCC-TTA--TGATTGGGAGA-TTCTGGC
AAAATCGTCTCT-CACACCCTC-TCAATTTTTACAATTTAAGACTTGGTG-----------GATTGATGGG-----GTAC
AAGAACA
>B100
A-GGGCC---AACTCCTCTTCCAGCAGTTCAGATGCCTGTAACATTACAACCTCAAATGCAGGTTAGGCAAGTACAAACC
CCAAGAGAATATCAAATAGAGAAAGATAGAGTCTCTGTCACGGCAATGCCAATCCAAATACAGTATCCACAATATCAGCC
GGTAGAAAATAAGACCCAACCGCCAGAAGCTTATCAATACTGGCCGCCGGCTGAACTTCAGTATCGGCCACCCCC-AGAA
AGTCAGTATGGATATCCAGGAATGCCCCCAGCACCACAGGGCAG----GGCGCCATACCCTCAGCTGCCCACTAGGAGAC
---TTAATCCTATGGCACCACCTAGT
AGACAGGGTAGTGAATTACATGAAATTATTGATAAA----------TCAAGAAAGGAAGGAGA--TACTGAGGCGTGGCA
ATTCCCAGTAACATTAGAACCGATGCCAACTGGAGAAGGA-----------GCCCAAGAGGGAGAGCCTCTCACAGTTGA
GGCCAGATACAAGTCTTTTTCGATAAAAATGCT-AAAAGATATGAAAGAGAGAGTAA--AACAGTATGGACCCAACTCCC
CTTATATGAGGACATTATTAGATTCCATTGCTCATGGACAT---AGACTCATTCC-TTA--TGATTGGGAGA-TTCTGGC
AAAATCGTCTCT-CTCACCCTC-TCAATTTTTACAATTTAAGACTTGGTG-----------GATTGATGGG-----GTTC
AAGAACA
>B81
A-GGGCC---AACTCCTCTTCCAGCAGTTCAGATGCCTGTAACATTACAACCTCAAATGCAGGTTAGGCAAGTACAAACC
CCAAGAGAATATCAAATAGAGAAAGATAGAGTCTCTGTCACGGCAATGCCAATCCAAATACAGTATCCACAATATCAGCC
AGTAGAAAATAAGACCCAACTGCCAGTAGCCTATCAATACTGGCCGCCGGCTGAACTTCAGTATCGGCCACCCCC-AGAA
AGTCAGTATGGATATCCAGGAATGCCCCCAGCACCACAGGGCAG----GGCGCCATACCCTCAGCTGCCCACTAGGAGAC
---TTAATCCTATGGCACCACCTAGT
AGACAGGGTAGTGAATTACATGAAATTATTGATAAA----------TCAAGAAAGGAAGGGGA--CACTGAGGCGTGGCA
ATTCCCAGTAACGTTAGAACCGATACCACCTGGAGAAGGA-----------GCCCAAGAGGGAGAGCCTCTCACAGTTGA
GGCCAGATACAAGTCTTTTTCGATAAAAATGCT-AAAAGATATGAAAGAGGGAGTAA--AACAGTATGGACCCAACTCTC
CTTATATGAGGACATTATTAGATTCCATTGCTCATGGACAT---AGACTCATTCC-TTA--TGATTGGGAGA-TTCTGGC
AAAATCGTCTCT-CTCACCCTC-TCAATTTTTACAATTTAAGACTTGGTG-----------GATTGATGGG-----GTAC
AAGAACA
>B260
A-GGGCC---AACTCCTCTTCCAGCAGTTCAGATGCCTGTAACATTACAACCTCAAATGCAGGTTAGGCAAGTACAAACC
CCAAGAGAATATCAAATAGAGAAAGATAGAGTCTCTGTCACGGCAATGCCAATCCAAATACAGTATCCACAATATCAGCC
GGTAGAAAATAAGACCCAACCGCAAGTAGCCTATCAATACTGGCCGCTGGCTGAACTTCAGTATCGGCCACCCCC-AGAA
AGTCAGTATGGATATCCAGGAATGCCCCCAGCACCACAGGGCAG----GGCGCCATACCATCAGCCGCCCACTAGGAGAC
---TTAATCCTATGGCACCACCTAGT
AGACAGGGTAGTGAATTACATGAAATTATTGATAAA----------TCAAGAAAGGAAGGAGA--TACTGAGGCATGGCA
ATTCCCAGTAACGTTAGAACCGATGCCACCTGGAGAAGGA-----------GCCCAAGAGGGAGAGCCTCCCACAGTTGA
GGCCAGATACAAGTCTTTTTCGATAAAAATGCT-AAAAGATATGAAAGAGGGAGTAA--AACAGTATGGACCCAACTCCC
CTTATATGAGGACATTATTAGATTCCATTGCTTATGGACAT---AGACTCATTCC-TTA--TGATTGGGAGA-TTCTGGC
AAAATCGTCTCT-CTCACCCTC-TCAATTTTTACAATTTAAGACTTGGTG-----------GATTGATAGA-----GTAC
AAGAACA
>B83
A-GGGCC---AACTCCTCTTCCAGCAGTTCAGATGCCTGTAACATTACAACCTCAAATGCAGGTTAGGCAAGTACAAACC
CCAAGAGAATATCAAATAGAGAAAGATAGAGTCTCTGTCACGGCAATGCCAATCCAAATACAGTATCCACAATATCAGCC
AGTAGAAAATAAGACCCAACCGCCAGTAGCCTATCAATACTGGCCGCCGGCCAAACTTCAGTATCGGCCACCCCC-AGAA
AGTCAGTATGGATATCCAGGAACGCTCCCAGCACCACAGGGCAG----GGTGCCATACCCTCAGCCACCCACTAGCAGAC
---TTAATCATACGGCACCACCTAGT
AGACAGGGTGGTGAATTACATGAAATTATTGATAAG----------TCAAGAAAGGAAGGAGA--TACTGAGGCATGGCA
ATTCCCAGTAACGTTAGAACCGATGCCACCTGGAGAAGGA-----------GCCCAAGAGGGAGAGCCTCCCACAGTTGA
GGCCAGATACAAGTCTTTTTCGATAAAAATGCT-AAAAGATATGAAAGAGGGAGTAA--AACAGTATGGACCCAACTCCC
CTTATATGAGGACATTATTAGATTCCATTGCTCATGGACAT---AGACTCATTCC-TTA--TGATTGGGAGA-TTCTGGC
AAAATCGTCTCT-CTCACCCTC-TCAATTTTTACAATTTAAGACTTGGTG-----------GATTGATAGA-----GTAC
AAGAACA
>B281
A-GGGCC---AACTCCTCTTCCAGCAGTTCAGATGCCTGTAACATTACAACCTCAAATGCAGGTTAGGCAAGTACAAACC
CCAAGAGAATATCAAATAGAGAAAGATAGAGTCTCTGTCACGGCAATGCCAATCCAAATACAGTATCCACAATATCAGCC
GGTAGAAAATAAGACCCAACTGCCAGTAGCCTATCAATACTGGCTGCCAGCCGAACTTAAGTGTCGGCCACCCCC-GGAA
AGTCAGTATGGATATCCAGGAATGCCCCCAGCACCACAGGGCAG----GGCGCCATACCCTCAGCTGCCCACTAGGAGAC
---TTAATCCTATGGCACCACCTAGT
AGACAGGGTAGTGAATTACATGAAATTATTGATAAA----------TCAAGAAAGGAAGGAGA--TACTGAGGCATGGCA
ATTCCCAGTAACGTTAGAACCGATGCCACCTGGAGAAGGA-----------GCCCAAGAGGGAGAGCCTCCCACAGTTGA
GGCCAGATACAAGTCTTTTTCGATAAAAATGCT-AAAAGATATGAAAGAGGGAGTAA--AACAGCATGGACCCAACTCCC
CTCATATGAGGACATTATTAGATTCCATTGCTCATGGACAT---AGACTCATTCC-TTA--TGATTGGGAGA-TTCTGGC
AAAATCGTCTCT-CTCACCCTC-TCAATTTTTACAATTTAAGACTTGGTG-----------GATTGATGGG-----GTAC
AAGAACA
>B270
A-GGGCC---AACTCCTCTTCCAGCAGTTCAGATGCCTGTAACATTACAACCTCAAATGCAGGTTAGGCAAGTACAAACC
CCAAGAGAATATCAAATAGAGAAAGATAGAGTCTCTGTCACGGCAATGCCAATCCAAATACAGTATCCACAATATCAGCC
GGTAGAAAATAAGACCCAACTGCCAGTAGCCTATCAATACTGGCTGCCAGCCGAACTTAAGTGTCGGCCACCCCC-GGAA

```
AATCAGTATGGACAGCCAGGAATGTTCCCAGCACCACAGGGCAG----GGCGCCATACCCTCAGCTGCCCACTAGGAGAC
---------------------------------------------------------TTAATCCTATGGCACCACCTAGT
AGACAGGGTAGTGAATTACATGAAATTATTGATAAA----------TCAAGAAAGGAAGGAGA--TACTGAGGCGTGGCA
ATTCCCAGTAACATTAGAACCGATACCACCTGGAGAAGGA-----------GCCCAAGAGGGAGAGCCTCCCACAGTTGA
GGCCAGATACAAGTCTTTTTCTATAAAAATGCT-AAAAGACATGAAAGAGGGAATAA--AACAGTATGGACCCAACTCCC
CTTATATGAGGACATTATTAGATTCCATTGCACACGGACAT---AGACTCATTCC-TTA--TGATTGGGAGA-TTCTGGC
AAAATCGTCTCT-CTCACCCTC-TCAATTTTTACAATTTAAGATTTGGTG-----------GATTAATGGG-----GCAC
AAGAACA
>B268
A-GGGCC---AACTCCTCTTCCAGCAGTTCAGATGCCTGTAACATTACAACCTCAAATGCAGGTTAGGCAAGTACAAACC
CCAAGAGAATATCAAATAGAGAAAGATAGAGTCTCTGTCACGGCAATGCCAATCCAAATACAGTATCCACAATATCAGCC
GGTAGAAAATAAGACCCAACCGCCAGTAGCCTATCAATACTGGCCTCCGGCTGAACTTCAGTATCGGCCACCCCC-AGAA
AGTCAGTATGGATATCCAGGAATGCCCCCAGCACCACAGGGCAG----GGCGCCATACCCTCAGCCGCCCACTAGGAGAC
---------------------------------------------------------TTAATCCTACGGCACCACCTAGT
AGACAGGGTAGTGAATTACATGAAATTATTGATAAA----------TCAAGAAAGGAAGGAGA--TACTGAGGCATGGCA
ATTCCCAGTAACGTTAGAACCGATGCCACCTGGAGAAGGA-----------GCCCAAGAGGGAGAGCCTCCCACAGTTGA
GGCCAGATACAAGTCTTTTTCGATAAAAATGCT-AAAAGATATGAAAGAGGGAGTAA--AACAGTATGGACCCAACTCCC
CTTATATGAGGACATTATTAGATTCCATTGCTCATGGACAT---AGACTCATTCC-TTA--TGATTGGGAGG-TTCTGGC
CAAATTGTCTCT-CTCACCCTC-TCAATTTTAACAATTTAAGACTTGGTG-----------GATTGATGGG-----GCAC
AACAACA
>B44
A-GGCAC---AAGTCCTCTTCCAGCAGGTCAGGTGCCCGTAAGATTACAACCTCAAACGCAGGTTAA-------------
--------------------------------------------------------------------------------
---AGAAAATAAGACCCAACCGCCAGTAGCTTATCAATACTGGCCGCCGGCTGAACTTCAGTATCGGCCACCCCC-AGAA
AGTCAGTATGGATATCCAGGAATGCCCCCAGCACCACAGGGCAG----GGCGCCATACCCTCAGCTGCCCACTAGGAGAC
---------------------------------------------------------TTAATCCTATGGCACCACCTAGT
AGACAGGGTAGTGAATTACATGAAATTATTGATAAA----------TCAAGAAAGGAAGGAGA--TACTGAGGCATGGCA
ATTCCCAGTAACGTTAGAACCAATGCCACCTGGAGAAGGA-----------GCCCAAGAGGGAGAGCCTCCCACAGTTGA
GGCCAGATACAAGTCTTTTTCGATAAAAATGCT-AAAAGATATGAAAGAGGGAGTAA--AACAGTATGGACCCAACTCTC
CTTATATGAGGACATTATTAGATTCCATTGCTCATGGACAT---AGACTCATTCC-TTA--TGATTGGGAGA-TTCTGGC
AAAATCGTCTCT-CTCACCCTC-TCAATTTTTACAATTTAAGACTTGGTG-----------GATTGATGGG-----GTAC
AAGAACA
>B148
A-GGCAC---AAGTCATCTTCCAGCAGGTCAGGTGCCCGTAACATTACAACCTCAAAAGCAGGTTAA-------------
--------------------------------------------------------------------------------
---AGAAAATAAGACCCAACCGCCAGTAGCCTATCAATACTGGCCGCCGGCTGAACTTCAGTATCGGCCACCCCC-AGAA
AGTCAGTATGGATATCCAGGAATGCCCCCAGCACCACAGGGCAG----GGCGCCATACCCTCAGCCACCCACTAGGAGAC
---------------------------------------------------------TTAATCCTACGGCACCACCTAGT
AGACGGGGTAGTGAATTACATGAAATTATTGATAAA----------TCAAGAAAGGAAGGAGA--TACTGAGGCATGGCA
ATTCCCAGTAACGTTAGAACCGATGCCACCTGGAGAAGGA-----------GCCCAAGAGGGAGAGCCTCCCACAGTTGA
GGCCAGATACAAGTCTTTTTCGATAAAAATGCT-AAAAGATATGAAAGAGGGAGTAA--AACAGTATGGACCCAACTCCC
CTTATATGAGGACATTATTAGATTCCATTGCTCATGGACAT---AAACTCATTCC-TTA--TGATTGGGAGA-TTCTGGC
AAAATCGTCTCT-CTCACCCTC-TCAATTTTTACAATTTAAGACTTGGTG-----------GATTGATGGG-----GTAC
AAGAACA
>B187
A-GGCAC---AAGTCCTCTTCCAGCAGGTCAGGTGCCCGTAAGATTACAACCTCAAAAGCAGGTTAA-------------
--------------------------------------------------------------------------------
---AGAAAATAAGACCCAACCGCCAGTAGCCTATCAATACTGGCCGCCGGCTGAACTTCAGTATCGGCCACCCCC-AGAA
AGTCAGTATGGATATCCAGGAATGCCCCCAGCACCACAGGGCAG----GGCGCCATACCATCAGCCGCCCACTAGGAGAC
---------------------------------------------------------TTAATCCTATGGCACCACCTAGT
AGACAGGGTAGTGAATTACATGAAATTATTGATAAA----------TCAAGAAAGGAAGGAGA--TACTGAGGCATGGCA
ATTCCCAGTAACGTTAGAACCGATGCCACCTGGAGAAGGA-----------GCCCAAGAGGGAGAGCCTCCCACAGTTGA
GGCCAGATACAAGTCTTTTTCGATAAAAATGCT-AAAAGATATGAAAGAGGGAGTAA--AACAGTATGGACCCAACTCCC
CTTATATGAGGACATTATTAGATTCCATTGCTTATGGACAT---AGACTCATTCC-TTA--TGATTGGGAGA-TTCTGGC
AAAATCGTCTCT-CTCACCCTC-TCAATTTTTACAATTTAAGACTTGGTG-----------GATTGATGGG-----GTAC
AAGAACA
>B45
A-GGCAC---AAGTCCTCTTCCAGCAGGTCAGGTGCCTGTAACATTACAACCTCAAACGCAGGTTAA-------------
--------------------------------------------------------------------------------
---AGAAAATAAGACCCAACCGCCAGTAGCCTATCAATACTGGCCGCCGGCTGAACTTCAGTATCGGCCACCCCC-AGAA
AGTTAGTATGGATATCCAGGAATGCCCCCAGCACCACAGGGCAG----GGCGCCATACCCTCAGCCGCCCACTAGGAGAC
---------------------------------------------------------TTAATCCTACGGCACCACCTAGT
AGACAGGGTAGTGAATTACATGAAATTATTGATAAA----------TCAAGAAAGGAAGGAGA--TACTGAGGCATGGCA
ATTCCCAGTAACGTTAGAACCGATGCCACCTGGAGAAGGA-----------GCCCAAGAGGGAGAGCCTCTCACAGTTGA
GGCCAGATACAAGTCTTTTTCGATAAAAATGCT-AAAAGATATGAAAGAGAGAGTAA--AACAGTATGGACCCAACTCCC
CTTATATGAGGACATTATTAGATTCCATTGCTCATGGACAT---AGACTCATTCC-TTA--TGATTGGGAGA-TTCTGGC
AAAATCGTCTCT-CTCACCCTC-TCAATTTTTACAATTTAAGACTTGGTG-----------GATTGATGGG-----GTTC
AAGAACA
>B2
A-GAGCC---AAGTCCTTTTCTAGCAGGTCAGATGCCCATAACATTACAACCTCAAACGCAGGTTAG-------------
--------------------------------------------------------------------------------
---AGAAAATAAGACCCAACCACCAGTAGCTTATCAATACTGGCCACTGGCTGAACTTCAGTATCAGCCACCCCC-AGAA
AGTCAGTATGGACATCCAGGAATGCTGCCAGCACTACAGAATAG----GGTGCCACACCCTCAGCCGCCCACTTTGAGAC
---------------------------------------------------------TTAATCCTATGGCACCACCTAGT
AGACAGGGTAGTGCATTACATAAAATTATTGTCAAGAAAATTATAGTCAAGAAAGCAAGGAGA--TACTGAGGTGTGGCA
ATTCCCAGTAATGTTGGAACTGATGCCACCTGGAGAAGGG-----------GCCCAAGAGGGAGAGCCTCCCACAGTTGA
```

GGCCAGATACAAGTCTTTTTCTATAAAAATGCT-AAAAGATAGGAAAGACGGAGTCA--AACAGTATGGACCCAACTCCC
CTTACATGAGGACATTATTAGATTCCATTGCTCATGGACAT---AGACTCATTCC-TTA--TGATTGGGAGA-TTCTGGC
AAAATCGTCTTT-CTCACCCTC-TCAATTTGTACAATTTAAGACTTGGTG-----------GATTGATAGG-----ACAC
AAGAACA
>B131
A-GGCAC---AAGTCCTCTTCCAGCAGGTCAGGTGCCTGTAACATTACAACCTCAAAAGCAGGTTAA-------------
----
---AGAAAATAAGACCCAACCGCCAGTAGCCTATCAATACTGGCCTCCGGCTGAACTTCAGTATCGGCCACCCCC-AGAA
AGTCAGTATGGATATCCAGGAATGCCCCCAGCACCACAGGGCAG----GGCGCAATACCCTCAGCCGCCCACTAGGAGAC
---TTAATCCTACGGCACCACCTAGT
AGACAGGGTAGTGAATTACATGAAATTATTGATAAA----------TCAAGAAAGGAAGGAGA--TACTGAGGCATGGCA
ATTCCCAGTAACGTTAGAACCGATGCCACCTGGAGAAGGA-----------GCCCAAGAGGGAGAGCCTCCCACAGTTGA
GGCCAGATACAAGTCTTTTTCGATAAAAATACT-AAAAGATATGAAAGAGGGAGTAA--AACAGTATGGACCCAACTCCC
CTTATATGAGGACATTATTAGATTCCATTGCTCATGGACAT---AGACTCATTCC-TTA--TGATTGGGAGA-TTCTGGC
AAAATCGTCTCT-CTCACCCTC-TCAATTTTTACAATTTAAGACTTGGTG-----------GATTGATGGG-----GTAC
AAGAACA
>B107
A-GGGCC---AACTCCTCTTCCAGCAGTTCAGATGCCTGTAACATTACAACCTCAAATGCAGGTTAGGCAAGTACAAACC
CCAAGAGAATATCAAATAGAGAAAGATAGAGTCTCTGTCACGGCAATGCCAATCCAAATACAGTATCCACAATATCAGCC
GGTAGAAAATAAGACCCAACTGCCAGTAGCCTATCAATACTGGCTGCCAGCCGAACTTAAGTGTCGGCCACCCCC-GGAA
AATCAGTATGGACAGCCAGGAATGTTCCCAGCCCCACAGGACAG----GGCGCCATACCCTCAGCCACCCACTATGAGAC
---TTAATCCTACAGCACCACCTAGT
GAGCAGGGTAGTGTATTACATAAAATTATTGATGAG----------GCAAGAAAACAAAGAGA--TATTGAGGCATGGCA
ATTCCCAGTAATATTAGAACCAATACCACCTGGAGAAGGG-----------GCCCAAGAGGGAGAGCCTCCCCTAGCTGA
GGCCAGATATGAGTCTTTTTCTATAAAAATGCT-AAAAGATATGAAAGAGGGAGTAA--AACAGTATGGACCCAACTCCC
CTTATATGAGGACATTATTAGATTCCATTGCTCATGGACAT---AGACTCATTCC-TTA--TGATTGGGAGG-TTCTGGC
CAAATTGTCTCT-CTCACCCTC-TCAATTTTAACAATTTAAGACTTGGTG-----------GATTGATGGG-----GCAC
AACAACA
>B112
A-GGGCC---AACTCCTCTTCCAGCAGTTCAGATGCCTGTAACATTACAACCTCAAATGCAGGTTAGGCAAGTACAAACC
CCAAGAGAATATCAAATAGAGAAAGATAGAGTCTCTGTCACGGCAATGCCAATCCAAATACAGTATCCACAATATCAGCC
GGTAGAAAATAAGACCCAACTGCCAGTAGCCTATCAATACTGGCTGCCAGCCGAACTTAAGTGTCGGCCACCCCC-GGAA
AATCAGTATGGACAGCCAGGAATGTTCCCAGCCCCACAGGACAG----GGCGCCATACCCTCAGCCACCCACTATGAGAC
---TTAATCCTACAGCACCACCTAGT
GAGCAGGGTAGTGTATTACATAAAATTATTGATGAG----------GCAAGAAAACAAAGAGA--TATTGAGGCATGGCA
ATTCCCAGTAATATTAGAACCAATACCACCTGGAGAAGGG-----------GCCCAAGAGGGAGAGCCTCCCCTAGCTGA
GGCCAGATATGAGTCTTTTTCTATAAAAATGCT-AAAAGATATGAAAGAGGGAGTAA--AACAGTATGGACCCAACTCCC
CTTATATGAGGACATTATTAGATTCCATTGCTCATGGACAT---AGACTCATTCC-TTA--TGATTGGGAGG-TTCTGGC
CAAATTGTCTCT-CTCACCCTC-TCAATTTTAACAATTTAAGACTTGGTG-----------GATTGATGGG-----GCAC
AACAACA
>B15
A-GGACC---AAGTCCTCTTCCAGTAGGTCAGATGCCCGTAACATTACAACCTCAAACGCAGGTTAG-------------
----
---AGAAAATAAGACCCAACTACCAGTAGCTTATCAATACTGGCCGCCGGCCAAACTTCAGTATCGGCCACCCCC-AGAA
AGTCAGTATGGATATCCAGGAACGCTCCCAGCACCACAGGGCAG----GGCGCCATACCCTCAGCCACCCACTAGCAGAC
---TTAATCATACGGCACCACCTAGT
AGACAGGGTAGTGAATTACATGAAATTATTGATAAG----------TCAAGAAAGGAAGGAGA--CACTGAGGCGTGGCA
ATTCCCAGTAACGTTAGAACCGATACCACCTGGAGAAGGA-----------GCCCAAGAGGGAGAGCCTCTCACAGTTGA
GGCCAGATACAAGTCTTTTTCGATAAAAATGCT-AAAAGATATGAAAGAGGGAGTAA--AACACTATGGACCCAACTCCC
CTTATATGAGGACATTATTAGATTCCATTGCTCATGGACATCATAGACTCATTCC-TTA--TGATTGGGAGA-TTCTGGC
AAAATCGTCTCT-CTCACCCTC-TCAATTTTTACAATTTAAGACTTGGTG-----------GATTGATGGG-----GCAC
AAGAACA
>B86
A-GGGCC---AAGTCCTCTTCCAGCAGGTCAGGTGCCCGTAACACTACAACCTCAAACGCAGGTTAA-------------
----
---AGAAAATAAGACCCAACCGCCAGTAGCCTATCAATACTGGCCTCCGGCTGAACTTCAGTATCGGCCACCCCC-AGAA
AGTCAGTACGGATATCCAGGAATGCCCCCAGCACCACAGGGCAG----GGCGCCATACCCTCAGCTGCCCACTAGGAGAC
---TTAATCCTACGGCACCACCTAGT
AGACAGGGTAGTGAATTACATGAAATTATTGATAAA----------TCAAGAAAGGAAGGAGA--TACTGAGGCATGGCA
ACTCCCAGTAACGTTAGAACCGATGCCACCTGGAGAAGGA-----------GCCCAAGAGGGAGAGCCTCCCACAGTTGA
GGCCAGATACAAGTCTTTTTCGATAAAAATGCT-AAAAGATATGAAAGAGGGAGTAA--AACAGTATGGACCCAACTCCC
CTTATAGGAGGACATTATTAGATTCCATTGCTCATGGACAT---AGACTCATTCCCTTA--TGATTGGGAGA-TTCTGGC
AAAATCGTCTCT-CTCACCCTCCTCAATTTTTACA-TTTAAGACTTGGTG-----------GATTGATAGA-----GTAC
AGGAACA
>B86_B107
A-GGGCC---AAGTCCTCTTCCAGCAGGTCAGGTGCCCGTAACACTACAACCTCAAACGCAGGTTAA-------------
----
---AGAAAATAAGACCCAACCGCCAGTAGCCTATCAATACTGGCCTCCGGCTGAACTTCAGTATCGGCCACCCCC-AGAA
AGTCAGTACGGATATCCAGGAATGCCCCCAGCACCACAGGGCAG----GGCGCCATACCCTCAGCCACCCACTATGAGAC
---TTAATCCTACAGCACCACCTAGT
GAGCAGGGTAGTGTATTACATAAAATTATTGATGAG----------GCAAGAAAACAAAGAGA--TATTGAGGCATGGCA
ATTCCCAGTAATATTAGAACCAATACCACCTGGAGAAGGG-----------GCCCAAGAGGGAGAGCCTCCCCTAGCTGA
GGCCAGATATGAGTCTTTTTCTATAAAAATGCT-AAAAGATATGAAAGAGGGAGTAA--AACAGTATGGACCCAACTCCC
CTTATATGAGGACATTATTAGATTCCATTGCTCATGGACAT---AGACTCATTCC-TTA--TGATTGGGAGG-TTCTGGC
CAAATTGTCTCT-CTCACCCTC-TCAATTTTAACAATTTAAGACTTGGTG-----------GATTGATGGG-----GCAC
AACAACA

>B86_B44
A-GGGCC---AAGTCCTCTTCCAGCAGGTCAGGTGCCCGTAACACTACAACCTCAAACGCAGGTTAA-------------
----
---AGAAAATAAGACCCAACCGCCAGTAGCCTATCAATACTGGCCTCCGGCTGAACTTCAGTATCGGCCACCCCC-AGAA
AGTCAGTACGGATATCCAGGAATGCCCCCAGCACCACAGGGCAG----GGCGCCATACCCTCAGCTGCCCACTAGGAGAC
---TTAATCCTACGGCACCACCTAGT
AGACAGGGTAGTGAATTACATGAAATTATTGATAAA----------TCAAGAAAGGAAGGAGA--TACTGAGGCATGGCA
ACTCCCAGTAACGTTAGAACCGATGCCACCTGGAGAAGGA-----------GCCCAAGAGGGAGAGCCTCCCACAGTTGA
GGCCAGATACAAGTCTTTTTCGATAAAAATGCT-AAAAGATATGAAAGAGGGAGTAA--AACAGTATGGACCCAACTCTC
CTTATATGAGGACATTATTAGATTCCATTGCTCATGGACAT---AGACTCATTCC-TTA--TGATTGGGAGA-TTCTGGC
AAAATCGTCTCT-CTCACCCTC-TCAATTTTTACAATTTAAGACTTGGTG-----------GATTGATGGG-----GTAC
AAGAACA
>B44_B45_B187
A-GGCAC---AAGTCCTCTTCCAGCAGGTCAGGTGCCCGTAAGATTACAACCTCAAACGCAGGTTAA-------------
----
---AGAAAATAAGACCCAACCGCCAGTAGCTTATCAATACTGGCCGCCGGCTGAACTTCAGTATCGGCCACCCCC-AGAA
AGTTAGTATGGATATCCAGGAATGCCCCCAGCACCACAGGGCAG----GGCGCCATACCCTCAGCCGCCCACTAGGAGAC
---TTAATCCTACGGCACCACCTAGT
AGACAGGGTAGTGAATTACATGAAATTATTGATAAA----------TCAAGAAAGGAAGGAGA--TACTGAGGCATGGCA
ATTCCCAGTAACGTTAGAACCGATGCCACCTGGAGAAGGA-----------GCCCAAGAGGGAGAGCCTCTCACAGTTGA
GGCCAGATACAAGTCTTTTTCGATAAAAATGCT-AAAAGATATGAAAGAGGGAGTAA--AACAGTATGGACCCAACTCCC
CTTATATGAGGACATTATTAGATTCCATTGCTTATGGACAT---AGACTCATTCC-TTA--TGATTGGGAGA-TTCTGGC
AAAATCGTCTCT-CTCACCCTC-TCAATTTTTACAATTTAAGACTTGGTG-----------GATTGATGGG-----GTAC
AAGAACA
>B2_B15_B2
A-GAGCC---AAGTCCTTTTCTAGCAGGTCAGATGCCCATAACATTACAACCTCAAACGCAGGTTAG-------------
----
---AGAAAATAAGACCCAACCACCAGTAGCTTATCAATACTGGCCACTGGCTGAACTTCAGTATCAGCCACCCCC-AGAA
AGTCAGTATGGACATCCAGGAATGCTGCCAGCACTACAGAATAG----GGTGCCACACCCTCAGCCGCCCACTTTGAGAC
---TTAATCATACGGCACCACCTAGT
AGACAGGGTAGTGAATTACATGAAATTATTGATAAG----------TCAAGAAAGGAAGGAGA--CACTGAGGCGTGGCA
ATTCCCAGTAACGTTAGAACCGATACCACCTGGAGAAGGA-----------GCCCAAGAGGGAGAGCCTCTCACAGTTGA
GGCCAGATACAAGTCTTTTTCGATAAAAATGCT-AAAAGATAGGAAAGACGGAGTCA--AACAGTATGGACCCAACTCCC
CTTACATGAGGACATTATTAGATTCCATTGCTCATGGACAT---AGACTCATTCC-TTA--TGATTGGGAGA-TTCTGGC
AAAATCGTCTTT-CTCACCCTC-TCAATTTGTACAATTTAAGACTTGGTG-----------GATTGATAGG-----ACAC
AAGAACA
>B112_B131
A-GGGCC---AACTCCTCTTCCAGCAGTTCAGATGCCTGTAACATTACAACCTCAAATGCAGGTTAGGCAAGTACAAACC
CCAAGAGAATATCAAATAGAGAAAGATAGAGTCTCTGTCACGGCAATGCCAATCCAAATACAGTATCCACAATATCAGCC
GGTAGAAAATAAGACCCAACTGCCAGTAGCCTATCAATACTGGCTGCCAGCCGAACTTAAGTGTCGGCCACCCCC-GGAA
AATCAGTATGGACAGCCAGGAATGTTCCCAGCCCCACAGGACAG----GGCGCAATACCCTCAGCCGCCCACTAGGAGAC
---TTAATCCTACGGCACCACCTAGT
AGACAGGGTAGTGAATTACATGAAATTATTGATAAA----------TCAAGAAAGGAAGGAGA--TACTGAGGCATGGCA
ATTCCCAGTAACGTTAGAACCGATGCCACCTGGAGAAGGA-----------GCCCAAGAGGGAGAGCCTCCCACAGTTGA
GGCCAGATACAAGTCTTTTTCGATAAAAATACT-AAAAGATATGAAAGAGGGAGTAA--AACAGTATGGACCCAACTCCC
CTTATATGAGGACATTATTAGATTCCATTGCTCATGGACAT---AGACTCATTCC-TTA--TGATTGGGAGA-TTCTGGC
AAAATCGTCTCT-CTCACCCTC-TCAATTTTTACAATTTAAGACTTGGTG-----------GATTGATGGG-----GTAC
AAGAACA
>B443
A-GGACC---AAGTCCTCTTCCAGTAGGTCAGATGCCCGTAACATTACAACCTCAAACGCAGGTTAG-------------
----
---AGAAAATAAGACCCAACTACCAGTAGCTTATCAATACTGGCCGCCGGCCAAACTTCAGTATCGGCCACCCCC-AGAA
AGTCAGTATGGATATCCAGGAACGCTCCCAGCACCACAGGGCAG----GGTGCCATACCCTCAGCCACCCACTAGCAGAC
---TTAGTCATACGGCACCACCTAGT
AGACAGGGTGGTGAATTACATGAAATTATTGATAAG----------TCAAGAAAGGAAGGAGA--CACTGAGGCGTGGCA
ATTCCCAGTAACGTTAGAACCGATACCACCTGGAGAAGGA-----------GCCCAAGAGGGAGAGCCTCTCACAGTTGA
GGCCAGATACAAGTCTTTTTCGATAAAAATGCT-AAAAGATATGAAAGAGGGAGTAA--AACACTATGGACCCAACTCCC
CTTATATGAGGACATTATTAGATTCCATTGCTCATGGACATCATAGACTCATTCC-TTA--TGATTGGGAGA-TTCTGGC
AAAATCGTCTCT-CTCACCCTC-TCAATTTTTACAATTTAAGACTTGGTG-----------GATTGATGGG-----GCAC
AAGAACA
>B474
A-GGCAC---AAGTCCTCTTCCAGCAGGTCAGGTGCCTGTAACATTACAACCTCAAACGCAGGTTAA-------------
----
---AGAAAATAAGACCCAACCGCCAGAAGCTTATCAATACTGGCCGCCGGCTGAACTTCAGTATCAGCCACCCCC-AGAA
AGTCAGTATGGATATCCAGGAATACCCCCAGCACCACAGGGCAG----GGCGCCATACCCTCAGCTGCCCACTAGGAGAC
---TTAATCCTACGGCACCACCTAGT
AGACAGGGTAGTGAATTACATGAAATTATTGATAAA----------CCAAGAAAGGAAGGAGA--TACTGAGGCGTGGCA
ATTCCCAGTAACATTAGAACCGATGCCAACTGGAGAAGGA-----------GCCCAAGAGGGAGAGCCTCCCACAGTTGA
GGCCAGATACAAGTCTTTTTCGATAAAAATGCT-AAAAGATATGAAAGAGGGAGTAA--AACAGTATGGACCCAACTCCC
CTTATATGAGGACATTATTAGATTCCATTGCTCATGGACAT---AGACTCATTCC-TTA--TGATTGGGAGA-TTCTGGC
AAAATCGTCTCT-CTCACCCTC-TCAATTTTTACAATTTAAGACTTGGCG-----------GATTGATAGA-----GTAC
AAGAACA
>B443_B474
A-GGACC---AAGTCCTCTTCCAGTAGGTCAGATGCCCGTAACATTACAACCTCAAACGCAGGTTAG-------------
----
---AGAAAATAAGACCCAACTACCAGTAGCTTATCAATACTGGCCGCCGGCCAAACTTCAGTATCGGCCACCCCC-AGAA

```
AGTCAGTATGGATATCCAGGAACGCTCCCAGCACCACAGGGCAG----GGTGCCATACCCTCAGCCACCCACTAGCAGAC
---------------------------------------------------------TTAGTCATACGGCACCACCTAGT
AGACAGGGTAGTGAATTACATGAAATTATTGATAAA----------CCAAGAAAGGAAGGAGA--TACTGAGGCGTGGCA
ATTCCCAGTAACATTAGAACCGATGCCAACTGGAGAAGGA-----------GCCCAAGAGGGAGAGCCTCCCACAGTTGA
GGCCAGATACAAGTCTTTTTCGATAAAAATGCT-AAAAGATATGAAAGAGGGAGTAA--AACAGTATGGACCCAACTCCC
CTTATATGAGGACATTATTAGATTCCATTGCTCATGGACAT---AGACTCATTCC-TTA--TGATTGGGAGA-TTCTGGC
AAAATCGTCTCT-CTCACCCTC-TCAATTTTTACAATTTAAGACTTGGCG-----------GATTGATAGA-----GTAC
AAGAACA
```

<u>Reproduction of HERV-K(HML-2) *gag* sequence recombinants</u>

We aimed to reproduce recomination events between HML-2 *gag* sequences in an *in vitro/ex vivo* approach. We utilized different HML-2 *gag* sequences that we had cloned in the course of the experiment (see manuscript text). The *gag* sequences differed from each other by presence of unique restriction enzyme sites at the 5' or the 3' end of the sequence. Specifically, *gag* sequence B280 uniquely displayed an HindIII site at the 5' end. Sequence B255 uniquely displayed an RsrII site at the 3' end. Sequence B15 uniquely displayed a PflFI site at the 5' end (Suppl. Fig. A).

We produced RNA from those cloned *gag* sequences using the SP6/T7 Transcription Kit (Roche). We purified the RNA using the RNeasy Mini Kit (Qiagen) columns. We eliminated plasmid template DNA by DNAse I treatment using Turbo DNA-free (Ambion Inc.) and following recommendations for rigorous DNAse treatment. We generated cDNA from mixed equal amounts of RNA from clones B255 and B15 and from clones B255 and B280. RNA's were diluted at 1:1000 and about 1.5 ng of each diluted RNA were included in mixed cDNA synthesis. We used the Omniscript RT Kit (Qiagen Inc.). An RT negative control was also included at that point. Kits were used according to the manufacturers' recommendations. We subjected cDNA and RT(–) control to a standard PCR employing *gag* primers (see manuscript text). We proceeded further only when the negative control displayed no product. We cloned PCR products into pGEM T-Easy (Promega) and prepared plasmid DNA from 200 (100 per mixed RNA's) clones using a standard miniprep procedure. We double-digested plasmid DNAs with appropriate restriction enzymes, followed by agarose gelelectrophoresis of restriction fragments.

Out of 100 analysed plasmid clones for mixture B255/B15, we identified 6 clones producing DNA fragments characteristic of recombination of two restriction enzyme sites in one sequence. We identified 8 clones that could not be digested at all, indicating recombination-mediated elimination of restriction enzyme sites (Suppl. Fig. B). Out of 100 analysed plasmid clones for mixture B255/B280, we identified 3 clones that indicated recombination of restriction enzyme sites, and 4 clones that indicated elimininination of sites.

Taken together, 21 out of 200 plasmid clones (=10.5%) showed clear signs of recombination events. Thus, recombination events observed in the HERV-K(HML-2) expression analysis, and resulting in HERV-KX sequences, could be reproduced in an *in vitro/ex vivo* approach.

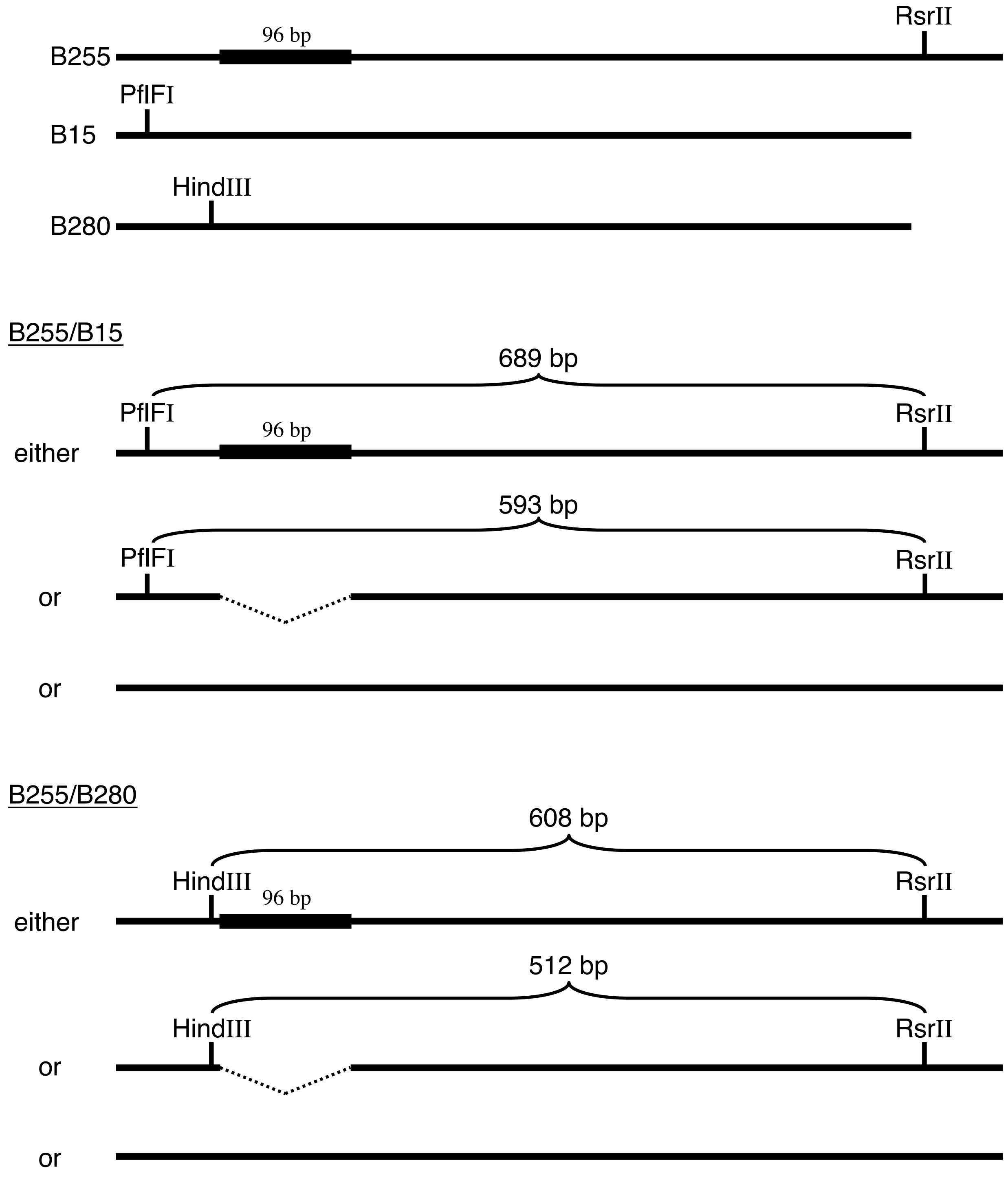

Suppl. Fig. A: Depiction of different HML-2 *gag* sequences employed to reproduce/screen recombinations *in vitro/ex vivo*. B255, B15 and B280 represent different *gag* sequences cloned into the pGEM T-Easy vector (Promega). B255 stems from locus 7_450 that is an HERV-K(OLD) provirus and that harbors a 96 bp insertion in its 5' region compared to B15 and B280 that stem from modern HML-2 proviruses (see manuscript text). The different *gag* sequences harbor unique restriction enzyme sites near their 5' or 3' ends. In the course of the *in vitro/ex vivo* experiment, recombinations result in combination of those sites within a clone, or in removal of those sites, depending on the direction of chimera formation. All three restriction enzymes do not cut the plasmid vector backbone. Indicated lengths of restriction fragments depend on presence/absence of the 96 bp sequence, and on direction and location of the recombination. We assayed RNA mixtures of clones B255/B15 and B255/B280. *Gag* sequences and location of restriction sites are not drawn to scale.

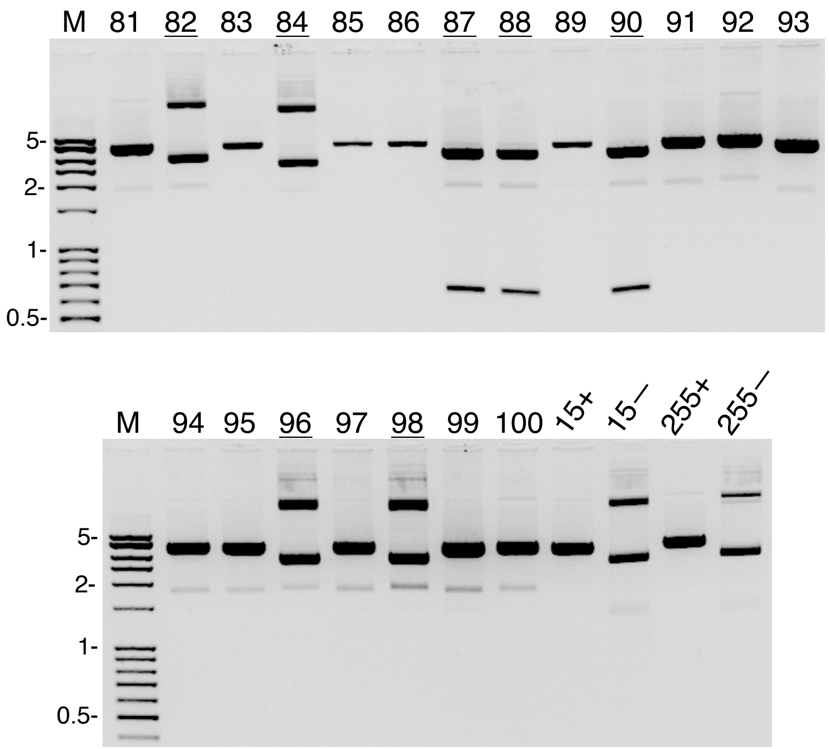

Suppl. Fig. B: Results of restriction enzyme-mediated screening for *in vitro/ex vivo* recombinants. Exemplarily shown are results for digestions of plasmid clones with PflFI and RsrII. As depicted in Suppl. Fig A, a restriction fragment of 689 bp or 593 bp is characteristic of recombination of both restriction sites in one plasmid clone (87, 88, 90), while undigestable plasmids indicate recombination-mediated elimination of both restriction sites from the plasmid clone (82, 84, 96, 98). Controls for properly digested plasmid B15+, B255+) and for undigested plasmid (15–, 255–) were included.
